# Identification of Novel miRNAs and miRNA Expression Profiling in Wheat Hybrid Necrosis

**DOI:** 10.1371/journal.pone.0117507

**Published:** 2015-02-23

**Authors:** Jianping Zhou, Yan Cheng, Meiqi Yin, Ennian Yang, Wenping Gong, Cheng Liu, Xuelian Zheng, Kejun Deng, Zhenglong Ren, Yong Zhang

**Affiliations:** 1 School of Life Science and Technology, University of Electronic Science and Technology of China, Chengdu 610054, China; 2 Crop Research Institute, Sichuan Academy of Agricultural Sciences, Chengdu 610066, China; 3 State Key Laboratory of Plant Breeding and Genetics, Sichuan Agricultural University, Wenjiang, Chengdu 611130, China; Nazarbayev University, KAZAKHSTAN

## Abstract

MicroRNAs (miRNAs) play essential roles in a vast array of biological processes, including growth and development, defense against viral infection, and responses to environmental changes in plant. Wheat hybrid necrosis is an interesting genetic phenomenon observed frequency and it is lethal or semi lethal, resulting in gradual death or loss of productivity. However, the molecular basis and mechanisms associated with hybrid necrosis in wheat are still not well understood. Here, we report the population and expression profiles of miRNAs in wheat hybrid necrosis. We identified a total of 57 conserved miRNA families as well as 182 putative novel miRNAs. Expression profiling revealed that expression of 49 known miRNAs and 165 novel miRNAs was changed in hybrid necrosis. And the expression levels of some miRNAs and their predicated targets have been confirmed by qRT-PCR. These results indicate that these miRNAs, especially miR159, miR166, miR167 and miR5072 could be involved in the extensive regulation of gene expression in response to hybrid necrosis.

## Introduction

MicroRNAs (miRNAs) are endogenous single-stranded noncoding RNAs (~21 nucleotides in length) that are key posttranscriptional regulator in eukaryotic gene expression by targeting mRNAs for cleavage or translational repression. More and more evidence show that miRNAs play central roles in a vast array of biological processes, including growth and development, defense against viral infection, and responses to environmental changes in eukaryotes [1–4]. In plants, the biogenesis of plant miRNAs is a complex multi-step enzymatic process [5–7]. miRNAs are transcribed by RNA polymerase II to primary miRNAs (pri-miRNAs) which are partially self-complementary and possess the fold-back hairpin structure, The pri-miRNAs are then processed to generate precursor miRNAs (pre-miRNAs) by the protein complex consisting of the Dicer-like 1 (DCL1), the C2H2-zinc finger protein SERRATE 11 (SE), and the double-stranded RNA-binding protein HYPONAS-TIC LEAVES1 (HYL1) in the cell nucleus[8,9]. The Pre-miRNAs or mature miRNAs (miRNA/miRNA* duplexes) are then exported to the cytoplasm mediated by the protein HASTY. After methyl groups are added to the ends of the duplexes catalyzed by the protein HEN1, one strand of the duplexes is selectively incorporated into the RNA-induced silencing complex (RISC) to form the mature miRNAs, whereas the other strand, designated miRNA*, is typically degraded[8, 9]. The miRNA strand is ultimately loaded into the Argonaute (AGO) protein of RNA-induced silencing complex (RISC) to carry out its function [8, 9]. Since the identification of the first miRNA from *Caenorhabditis elegans* through genetic screens for aberrant development [10, 11], Recently, thousands of miRNAs have been identified in various multicellular eukaryotes and are released in the miRBase database (http://www.mirbase.org/, Release 21, June 2014) [12–14]. Although hundreds of plant miRNAs and their targets have been identified, the majority of studies are still focused on model plant species, such as *Arabidopsis thaliana*, *Oryza sativa*, *Zea mays* and *Nicotiana tabacum* [12, 13, 15, 16]. To further understand the function of plant miRNAs, more efforts should be made to include plant species with specific developmental and genetic features, which might contain miRNAs that are specific to these features [17]. With the rapid development of high throughput DNA deep sequencing technology, non-conserved miRNAs from divergent plant species, including wheat [18, 19], rose [20], peanut [21], grape [22], barley [23], cucumber [24], olive [25], tomato [26], apple [27], and peach [28] have been identified.

Wheat (*Triticum aestivum*, AABBDD, 2n = 42) is a globally important crop, occupying 17% of all the cultivated land and accounting for 20 percent of the calories consumed by humans [29, 30], and is, therefore, of great economic importance. Hybrid necrosis has been frequently observed in F_1_ hybrids between genotypes of common wheat [31]. Hybrid necrosis is usually lethal or semi-lethal, resulting in gradual death or loss of productivity [32–34]. Therefore, hybrid necrosis is a serious barrier either for combining desirable traits from different genotypes of common wheat or for transferring genes from related species to commercial cultivars [32, 35]. The literature presented the hypothesis that hybrid necrosis can result from autoimmunity, perhaps as a pleiotropic effect of evolution of genes that are involved in pathogen response [36]. However, details regarding the molecular basis and mechanisms associated with hybrid necrosis in wheat are still not well understood. Recently, some miRNAs have been isolated and identified from wheat[18], [19], [37–39], but, compared with the number of miRNAs that have been identified in model plants such as *Arabidopsis thaliana*, *Oryza sativa* and *Nicotiana tabacum*, more miRNAs should be identified from wheat to understand the development, genetic phenomenon and stress response in plants. In the present research, we report small RNA profiling in wheat hybrid necrosis to identify new miRNAs and discover hybrid necrosis regulated miRNAs through high-throughput sequencing approach.

## Results

### Construction and Sequencing of Small RNA Libraries

The F_1_ hybrids between common wheat Neimai8 (N8) and II469 show hybrid dwarfness belonging to necrosis (S1 Fig.). To obtain a comprehensive survey of miRNAs in hybrid necrosis wheat, we constructed and sequenced two small RNA libraries from wheat F_1_ hybrids and its parents (mixed tissues of N8 and II469), respectively.

**Fig 1:**
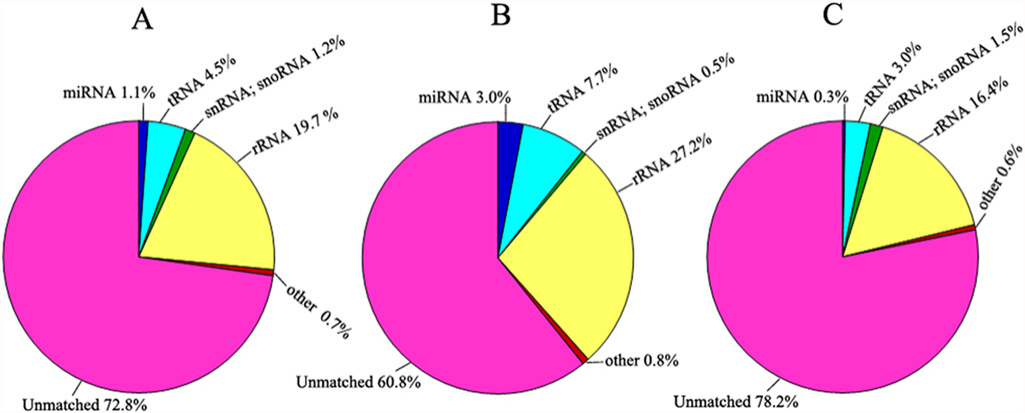
Distribution of small RNAs among different categories. (A) Total small RNAs from the libraries of F_1_ hybrids and their parents (N8 and II469). (B) Small RNAs from F_1_ hybrids. (C) Small RNAs from N8 and II469.

In all, 9,901,074 and 20,872,856 reads were respective obtained from the Illumina Hiseq2000 sequencing machine for the small RNA libraries of the F_1_ hybrids and its parents. After removing the adaptor/acceptor sequences, filtering the low-quality tags and cleaning up the contamination formed by the adaptor-adaptor ligation, total 15,344,192 clean reads were obtained, representing 1,125,608 unique sequences. Among the total reads, 166,482 were found to be similar to miRNAs. The rest of the sequences were found to be other types of RNA, including non-coding RNA, tRNA, rRNA, snRNA or snoRNA. The proportions of different categories of small RNAs are given in Fig. 1A–C.

In addition, total 11,336,796 clean reads were mapped to genome (S1 Table). The majority of the reads were distributed on contig214090, contig87233 and contig5021661, and accounted for 5958705 reads (52% of the total reads), 3130665 reads (27.56% of the total reads) and 104166 reads (0.92% of the total reads), respectively. 519199 unique sequences from F_1_ sample and 470596 unique sequences from parents sample were respective mapped to genome (S1 Table).

**Fig 2:**
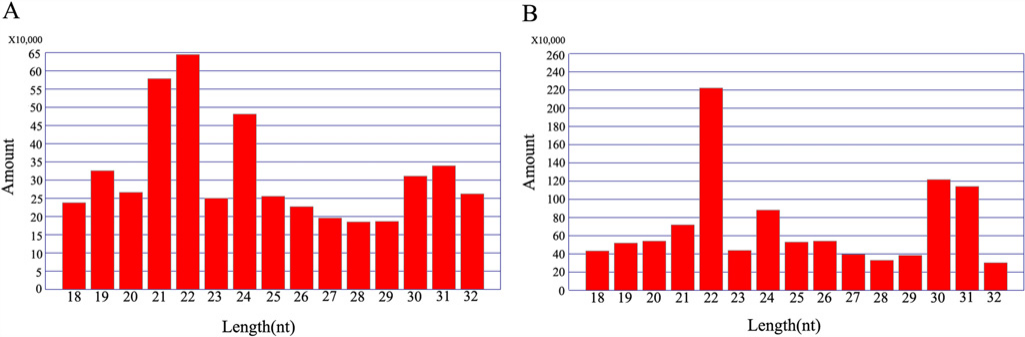
Size distribution of small RNA (sRNA) sequences from wheat. (A) Small RNAs from F_1_ hybrids. (B) Small RNAs from N8 and II469.

The composition of different categories of small RNAs often reflects the roles in a particular tissue or species and associated biogenetic machines. The length distribution of the small RNAs ranged from 18 to 32 nt was examined and shown in Fig. 2A–B. In F_1_ sample, 22-nt, 21-nt and 24-nt small RNAs were the major population, while 22-nt, 24-nt, 30-nt and 31-nt small RNAs were the major population in its parents sample. All the small RNA sequences have been deposited into NCBI SRA database under accession number: SRX500281.

### Identification of Conserved miRNAs

There are 24,521 hairpin precursor miRNAs and 30,424 mature miRNAs including thousands of plant miRNAs from more than 200 species deposited in the miRBase database (http://www.mirbase.org/, Release 21, June 2014)[12–14]. Here, we explore the miRNAs in our sequencing data to systematically identify both conserved and species-specific miRNAs in wheat. To identify conserved miRNAs in wheat, we aligned the small RNA sequences against the known plant mature miRNAs registered in the miRBase (Release 21: June 2014), and their corresponding precursor sequences were checked to insure the miRNAs have their expected secondary structures. On the basis of sequence similarity, our analysis revealed that a total of 57 known miRNA families were identified and listed in S2 Table. Among them, 29 families were known and well-conserved, including miR156, miR158, miR159, miR160, miR164, miR165, miR166, miR167, miR168, miR169, miR170, miR171, miR172, miR319, miR393, miR394, miR396, miR397, miR398, miR399, miR444, miR530, miR827, miR1120, miR1128, miR1432, miR1436, miR1511, miR2911. Twenty-eight families were known but not well-conserved, including miR1125, miR1127, miR1136, miR5139, miR6478, miR894, miR1318, miR5071, miR5072, miR5073, miR5077, miR5082, miR5083, miR5538, miR818, miR5049, miR5048, miR6191, miR6203, miR3630, miR4995, miR5368, miR6300, miR5054, miR5062, miR5064, miR5200, miR5203.

Interestingly, wheat has several less-conserved miRNAs which were reported previously only in monocots, such as miR5054, miR5062, miR5064, miR5200, miR5203 in *Brachypodium distachyon* [40], miR5049, miR5048, miR6191, miR6203 in *Hordeum vulgare* [23, 41], miR1318, miR5071, miR5072, miR5073, miR5077, miR5082, miR5083, miR5538, miR818 in *Oryza sativa* [42], miR1125, miR1127, miR1136 in wheat [18].

### Identification of Novel miRNAs in wheat

The genome sequences (http://mips.helmholtz-muenchen.de/plant/wheat/uk454survey/index.jsp) [29] and EST sequences (http://wheat.pw.usda.gov/GG2/) of wheat were also used to predict the potential novel miRNAs in wheat. A total of 182 novel miRNAs were obtained and named tae-miR2131a to tae-miR2256 (S3 Table). Those miRNAs which were located different contigs and have the consensus mature sequence were characterized the same family. So these novel miRNAs belong to 126 miRNA families. 31 out of the 126 miRNA families have more than one member. For example, tae-2132 family has seven members.

In addition, the corresponding miRNA* and precursor sequence were identified for these novel miRNAs, further supporting their existence as miRNAs (S3 Table). Furthermore, the stem-loop structures of these miRNAs were also predicted (S3 Table; S2 Fig.).

### miRNAs expression patterns in wheat hybrid necrosis

**Fig 3:**
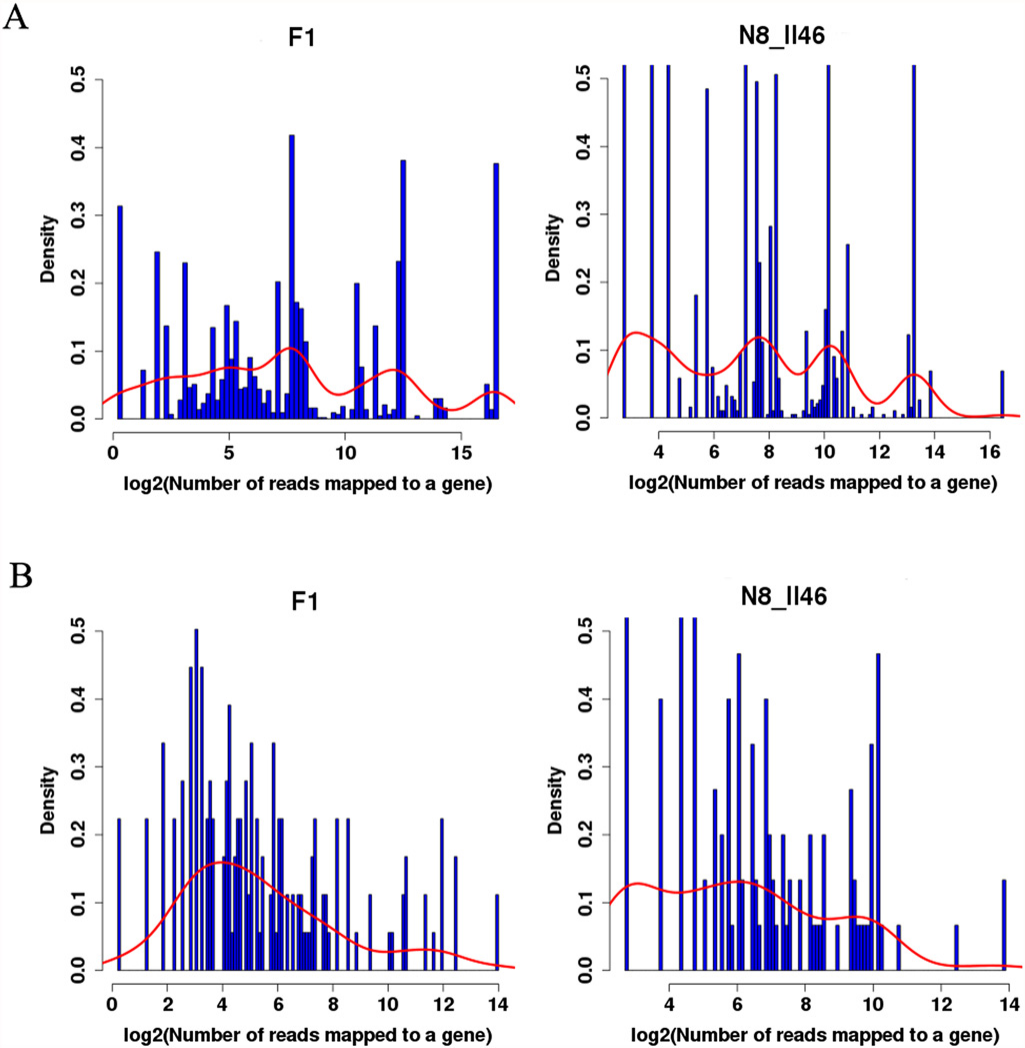
Distribution of miRNAs expression. Different expression of known miRNAs (A) and novel miRNAs (B) from F_1_ hybrids and their parents (N8 and II469).

Knowledge of the expression patterns of miRNAs could provide clues about their functions. It has been reported that high-throughput sequencing can be used as a tool for miRNA expression profiling [39, 43]. Therefore, in this study expression profiling of some known and new identified miRNAs was also determined based on illumina sequencing data (Fig. 3A–B; S2 and S3 Tables). Compared to parents (N8 and II469), the expression profiling of both known and new miRNAs in F_1_ hybrids was different (Fig. 3A–B). In addition, the most normalization abundant three, miR166, miR165, miR168 reached to 98607, 79571, and 30573, respectively (S2 Table). The consensus results were also obtained from rice and wheat in previous study [39, 44, 45].

Compared with the conserved miRNAs, most of the novel miRNAs were relatively in low abundance (S3 Table). Among the 182 novel miRNAs, only three (Tae-2131a, Tae-2131b and Tae-2131c) had 18190 transcripts. Moreover, in order to determine the response or cause of wheat miRNAs to hybrid necrosis, we looked for the miRNAs that were up- or down- regulated in F_1_ hybrids compared to its parents (S3 Table). We can observed that 49 conserved miRNAs and 165 novel miRNAs were identified to be significantly relative to hybrid necrosis from S2 and S3 Tables, indicating that miRNAs could be involved in the extensive regulation of gene expression in response to hybrid necrosis.

### Target predictions for wheat miRNAs

In order to better understand possible biological functions of the newly identified miRNAs as well as the known miRNAs in wheat hybrid necrosis, we searched for putative target genes using the Miranda program (http://www.miRNA.org/miRNA/home.do) with default parameters according to the wheat EST sequences database (http://wheat.pw.usda.gov/GG2/ and http://www.ncbi.nlm.nih.gov/) and the Triticeae Full-Length CDS Database, TriFLDB (http://trifldb.psc.riken.jp/v3/index.pl). Putative targets of 57 known miRNA families and 182 novel miRNAs were predicted (S2, S3, S4, S5 Tables). Among the known miRNAs, total 523 ESTs and 18 full-length cDNAs as target genes were found for miR164 while only one full-length cDNA (RFL_Contig3586) as target was predicted for miR5077 (S2 and S4 Tables). And more than 500 ESTs as target genes were respectively obtained for miR-tae2159 families, miR-tae2192 families and miR-tae2203 families while there was no EST as target for miR-tae2253 (S3 and S5 Tables). There were no more ideal or deep analysis results but the above preliminary target predictions for these miRNAs were obtained, which could be due to the limited of wheat EST sequences available in the databases and the absent of wheat genome annotation and more detailed gene annotation of the full length cDNA library.

### miRNA and predicated targets validation

**Fig 4:**
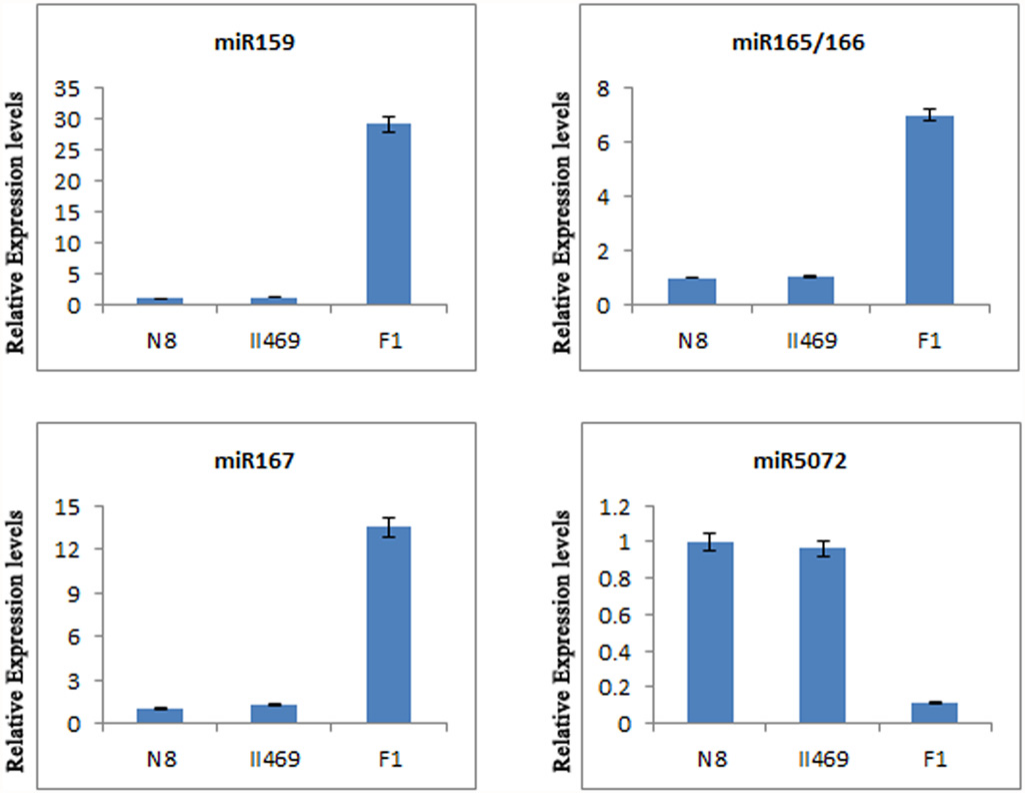
The expression levels of miR159, miR165/166, miR167 and miR5072. The expression levels were assayed by qRT-PCR. Bars show standard error.

To verify the existence and expression change of the identified wheat miRNAs and predicated targets, RNA preparation subjected to quantitative RT-PCR (qRT-PCR) was the same as those used in the Illumina sequencing assay. In this study, 5 miRNAs (tae-miR159, tae-miR165/166, tae-miR167 and tae-miR5072) were validated and measured using qRT-PCR (Fig. 4). As shown in the Fig. 4, the expression changes of these miRNAs in F_1_ and its parents are similar to the results of Illumina sequencing. These results suggest that miRNAs had been successfully and accurately discovered from wheat hybrid necrosis with Illumina sequencing.

Moreover, 7 predicated targets (AK248335 and AK249930 for miR165/166, AJ748348_1 and AK248211 for miR159, AK248413 and RFL_Contig2908 for miR167, AK253010 for miR5072) were chosen randomly in TriFLDB (S2 Table) and validated using qRT-PCR (S3 Fig.). As shown in S3 Fig., except AK249930, there is not obvious different for the expression levels of the predicated targets in F_1_ compared to its parents, indicating that the expression levels of miR159, miR167, miR5072 and their predicated respective targets did not show an obvious negative correlation. There are two reasons for this situation, one is many miRNAs regulate their target(s) by translation inhibition but not slicing activity [9], and another is these predicated targets are not real targets for the miRNA.

## Discussion

Hybrid necrosis was belonging to postzygotic hybrid incompatibilities which involve epistatic interactions as predicted by the Bateson-Dobzhansky-Muller (BDM) model [46]. And a further study showed hybrid incompatibility depended on activation of the salicylic acid (SA) stress signaling pathway [47]. Moreover, the hypothesis that hybrid necrosis can result from autoimmunity was also presented [36]. However, Dalal and Khanna-Chopra et al. reported that hybrid necrosis in wheat leaves was associated with oxidative stress without a well-coordinated antioxidant defense system [48, 49]. Although hybrid necrosis in wheat was first reported in the 1940s [50] and a series of classical researches revealed that this phenomenon was genetically controlled by two complementary dominant genes *Ne1* and *Ne2* located on chromosome arms 5BL and 2BS, respectively [34, 51–54], the molecular basis and mechanisms associated with hybrid necrosis in wheat are still not well understood.

MiRNAs have fundamental functions in regulating almost all aspects of plant development and in response to stress. Although miRNAs have been studied extensively in the past several years, only a few documents have been reported for wheat, one of the most important crops cultivated worldwide. Recently, some miRNAs have been isolated and identified from wheat [18, 19, 37–39], but the identity and function of most wheat miRNAs are still largely unknown. In the present study, we performed small RNA sequencing and determined the expression profiles of miRNAs in wheat hybrid necrosis. We identified 57 known miRNA families and 182 novel miRNAs, as well as their corresponding precursors and prediction targets. Among of them, 49 conserved miRNAs and 165 novel miRNAs were appeared to be differentially expressed during hybrid necrosis (S2 and S3 Tables), indicating that miRNAs could be involved in the extensive regulation of gene expression in response to hybrid necrosis. For conserved miRNAs, except for 15 miRNAs (highlighted in red letter in S2 Table) have been reported in wheat [18, 19, 37–39], 34 miRNAs were identified in wheat by us.

The most interesting and striking miRNAs with high changes during hybrid necrosis were miR165, miR166, miR159 and miR167 (S2 Table). Previous work showed that miR165 and miR166 were different in sequence by only a single nucleotide [55] and their same targets were thought to be the class III homeodomain leucine-zipper (HD-ZIP III) genes [56]. Over expressions of miR165 caused organ polarity alternations and defects in development of vascular tissues and inter fascicular fibers [57]. And a majority of miR166-overexpressing transformants also demonstrated a diverse array of phenotypic alternations such as downward curled leaves and stunted growth; some eventually died after the appearance of a few pairs of rosette leaves [58]. In the present study, the dramatic increase of expressions of miR165 and miR166 in F_1_ hybrids were observed (S2 Table, Fig. 4), suggesting that miR165 and miR166 should be responsible for wheat hybrid necrosis. Moreover, previously reports showed and confirmed that miR159 regulated the expression of a family of seven transcription factors that includes the two redundant GAMYB-like genes, MYB33and MYB65 as positive regulators of ABA responses though overexpression of miR159 [59, 60] and the loss-of-function mutations (T-DNA insertional mutants) in miR159 [61]. Overexpression of miR159a delayed the flowering of short day grown plants, as well as male sterility due to disruption of anther development [59, 60]. MiR159 over-expression also rendered plants hyposensitive to ABA [62]. And the mir159ab double mutant has pleiotropic morphological defects, including altered growth habit, curled leaves, small siliques, and small seeds [61]. Compared to parents, miR159 presented over expression in F_1_ hybrids (S2 Table, Fig. 4) in this study. it was reasonable for us to believe that miR159 was relative to hybrid necrosis. In addition, Yang et al. [63] and Wu et al. [64] reported that miR167 controlled patterns of the auxin responsive factor 6 (ARF6) and auxin responsive factor 8 (ARF8), and regulated both female and male reproduction in Arabidopsis and rice. Overexpressing miR167 mimicked the double mutant *arf6arf8* phenotypes, such as short hypocotyls, short internode, reduced stem elongation, plant height dwarf [64]. In our study, the same phenotypes and the high increase of miR167 expression were observed in wheat F_1_ hybrids (S2 Table, Fig. 4), indicating that miR167 maybe play important role in wheat hybrid necrosis. Furthermore, the expression patters of many abiotic/biotic stress relative miRNAs have been sharply changed in this paper (S2 Table), such as miR169 [65], miR444 [66], miR1511 [67], miR5139 [20] and miR5368 [68] which were related to nitrogen-starvation responses, dehydration stress response, microbes response, ethylene, water deficit and rust-stress responses, respectively. Considering those above, we can propose that F_1_ hybrids between some different genotypes of common wheat give rise to hybrid incompatibility which triggered autoimmunity. In order to response to autoimmunity, many miRNAs expression patterns were changed to adjust their targets. Of these miRNAs were ones that regulated the expression genes which were relative to hormones, development, growth and abiotic/biotic stress. Especially, the markedly increase of expressions of miR165/miR166, miR159 and miR167 were the major cause of hybrid necrosis.

## Materials and Methods

### Plant materials

Hexaploid wheat (*Triticum aestivum* L.) cultivar Neimai8 (N8), line II469 and their (cross and reciprocal cross) F_1_ hybrids were grown in a growth chamber at a relative humidity of 75% and 26/20°C day and night temperature. Seven days later, these seedling plants were transferred into the growth chamber with 4°C temperature because of F_1_ hybrids presented much more obvious dwarfness in low temperature (4°C) than in higher temperature (above 20°C). After seven days, the whole seedling plants including leaves and roots were frozen immediately in the liquid nitrogen, and stored at -80°C for further use. The same treatment was replicated twice.

### Small RNA library development and sequencing

Total RNA was isolated using TRIzol (Invitrogen, USA) and then was purified. Small RNA libraries were prepared and sequenced according to the manufacturer’s instructions and sequenced on an Illumina HiSeq2000 system (Majorbio BioTech, China).

### Small RNA analysis and miRNAs Prediction

The bioinformatics analysis of small RNAs was performed as described previously with minor modification [20]. Briefly, the raw sequencing data were processed to trim the adapter sequences and remove low quality sequences, and rRNA, tRNA, snRNA, snoRNA and degradation fragments of mRNAs sequences were also removed. The cleaned small RNA sequences were aligned to the wheat genome http://mips.helmholtz-muenchen.de/plant/wheat/uk454survey/index.jsp) [29] and the wheat transcriptome database sequences (http://wheat.pw.usda.gov/GG2/) using Bowtie (http://bowtie-bio.sourceforge.net/index.shtml) with perfect matches. Only sRNAs with no more than 20 hits were kept and their flanking sequences on the genome or transcriptome (200 bp on each side) were extracted and then folded in silico using the RNAfold program RNAfold (http://www.tbi.univie.ac.at/RNA/). Resulting folded structures were checked with miRdeep2 (http://www.mdc-berlin.de/en/research/research_teams/systems_biology_of_gene_regulatory_elements/projects/miRDeep/index.html) with default parameters. Candidate miRNAs whose precursors passed miRdeep2 were then aligned to the miRNA database, miRBase 18.0, using Bowtie. The miRNAs shared homology with known miRNAs were identified as conserved miRNA candidates. Then, they were further confirmed by checking their corresponding precursor structures. Only the candidates with expected structures were identified as conserved miRNAs.

After identifying all candidate miRNAs, those which did not share homology to all known sequences in miRBase were regarded as novel miRNA candidates. And the novel miRNAs’ precursor structures were further analyzed by miRdeep2. Potential miRNA star sequences were identified from the sRNA data set to provide additional evidence supporting miRNA predictions.

### miRNAs expression analysis

The different expression patters of the conserved miRNAs and novel miRNAs in sample F_1_ hybrids and its parents was carried out by DEGseq program (http://www.bioconductor.org/packages/release/bioc/html/DEGseq.html).

### Prediction of miRNA targets

All of the conserved and novel rose miRNAs were aligned against wheat transcriptome dataset (http://wheat.pw.usda.gov/GG2/) using Miranda program with default parameters (http://www.miRNA.org/miRNA/home.do) and the Triticeae Full-Length CDS Database, TriFLDB(http://trifldb.psc.riken.jp/v3/index.pl).

### miRNA and predicated targets validation

The identified wheat miRNAs were validated by using quantitative real time PCR (qRT-PCR). In this study, 5 miRNAs (tae-miR159, tae-miR165/166, tae-miR167 and tae-miR5072) and 7 predicated targets (AK248335, AK249930, AJ748348_1, AK248211, AK248413, RFL_Contig2908, and AK253010) were validated. The primers were listed in S6 Table.

Total RNA was extracted using TRIzol (Invitrogen, USA). Purified RNA was first treated with DNase I to remove any potential genomic DNA contamination, and then used for cDNA Synthesis.

For examination of the levels of miRNA, miRNA cDNA Synthesis was carried out using miRcute miRNA cDNA kit (Tiangen, China) according to the manufacturer’s instructions. Quantitative real time PCR was performed using the miRcute miRNA qPCR detection kit (Tiangen, China). U6 RNA was used as an internal control.

For examination of the levels of predicated target genes, reverse transcription reaction (RT) was carried out with a RevertAid First Strand cDNA Synthesis Kit (Thermo Scientific, USA), and qRT-PCR was performed with an SuperReal PreMix Plus (SYBR Green) PCR master mix kit (Tiangen, China) according to the manufacturer’s instructions. Actin mRNA was used as an internal control.

Values were obtained by normalizing to U6 or Actin and then comparing the normalized values to those of control plants. The relative levels of gene expression were calculated using the 2-△△ cycle threshold method. Three biological replicates were examined to ensure reproducibility.

## Supporting Information

S1 Fig The phenotype of wheat F1 hybrids derived from Neimai8 (N8) and II469. (DOCX)

S2 Fig Examples of the predicated secondary structures of miRNAs. Red colored letter: mature miRNA sequence; yellow colored letter: loop sequence; blue colored letter: miRNA* sequence.
(ZIP)

S3 Fig qRT-PCR analysis of some predicated targets expression levels. (DOCX)

S1 Table Small RNAs genome mapped. (ZIP)

S2 Table Known miRNAs identified from wheat and their expression patterns. (DOCX)

S3 Table Prediction of novel miRNAs and their precursors and expression targets. (DOCX)

S4 Table Identified targets of conserved miRNAs targets. (XLSX)

S5 Table Identified targets of new miRNAs in wheat. (XLSX)

S6 Table Real-time PCR primers used in this study. (XLSX)
