# Supplementary figures and images for: Identification of Novel miRNAs and miRNA Expression Profiling in Wheat Hybrid Necrosis
Source: PLoS One. 2015 Feb 23;10(2):e0117507. doi: 10.1371/journal.pone.0117507 (PMC4338152; doi:10.1371/journal.pone.0117507)

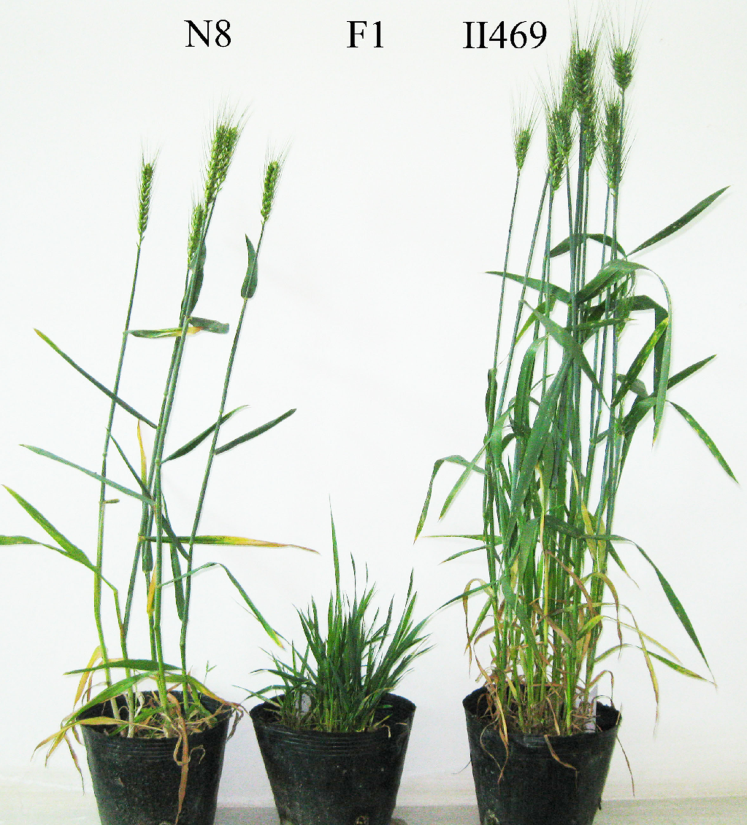


**Figure S1.** the phenotype ofwheatF1 hybrids derived from Neimai8 (N8) and II469

Supplement: S1 Fig — (DOCX) [file pone.0117507.s001.docx]

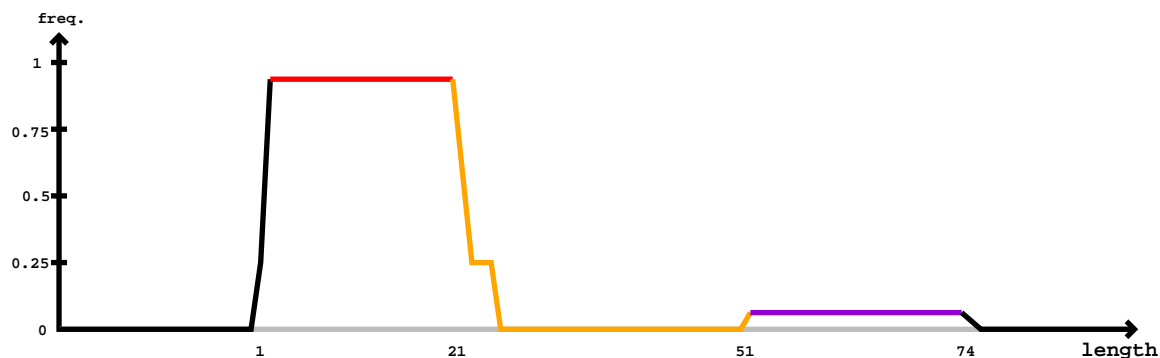

Star

[illegible]

Supplement: S2 Fig — Red colored letter: mature miRNA sequence; yellow colored letter: loop sequence; blue colored letter: miRNA* sequence. (ZIP) [file pone.0117507.s002.zip › Figures s1/contig102619_2645.pdf]

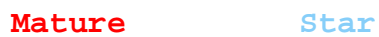[illegible]

Supplement: S2 Fig — Red colored letter: mature miRNA sequence; yellow colored letter: loop sequence; blue colored letter: miRNA* sequence. (ZIP) [file pone.0117507.s002.zip › Figures s1/contig1047394_9933.pdf]

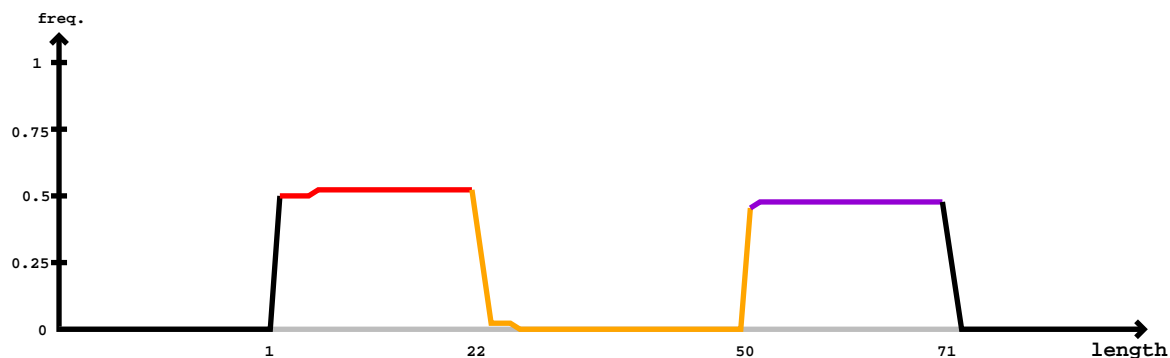

Star

[illegible]

Supplement: S2 Fig — Red colored letter: mature miRNA sequence; yellow colored letter: loop sequence; blue colored letter: miRNA* sequence. (ZIP) [file pone.0117507.s002.zip › Figures s1/contig1047595_9935.pdf]

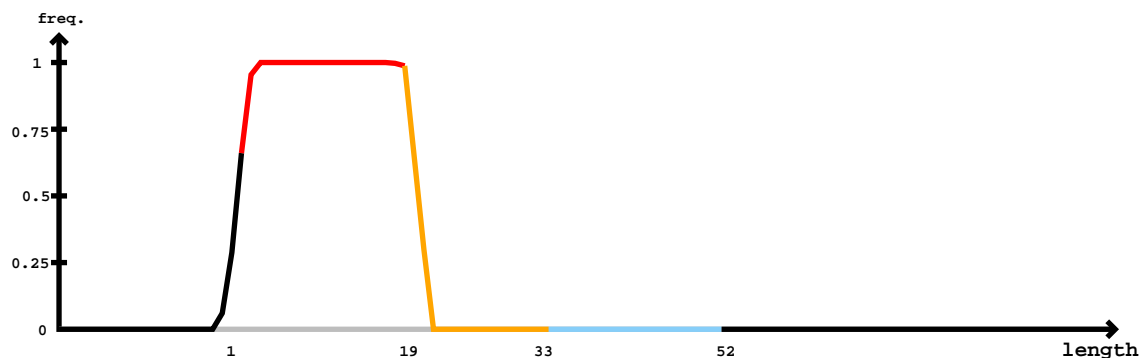

Star

[illegible]

Supplement: S2 Fig — Red colored letter: mature miRNA sequence; yellow colored letter: loop sequence; blue colored letter: miRNA* sequence. (ZIP) [file pone.0117507.s002.zip › Figures s1/contig106441_2711.pdf]

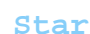[illegible]

Supplement: S2 Fig — Red colored letter: mature miRNA sequence; yellow colored letter: loop sequence; blue colored letter: miRNA* sequence. (ZIP) [file pone.0117507.s002.zip › Figures s1/contig1067062_10017.pdf]

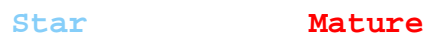[illegible]

Supplement: S2 Fig — Red colored letter: mature miRNA sequence; yellow colored letter: loop sequence; blue colored letter: miRNA* sequence. (ZIP) [file pone.0117507.s002.zip › Figures s1/contig112667_2845.pdf]

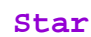[illegible]

Supplement: S2 Fig — Red colored letter: mature miRNA sequence; yellow colored letter: loop sequence; blue colored letter: miRNA* sequence. (ZIP) [file pone.0117507.s002.zip › Figures s1/contig1160517_10417.pdf]

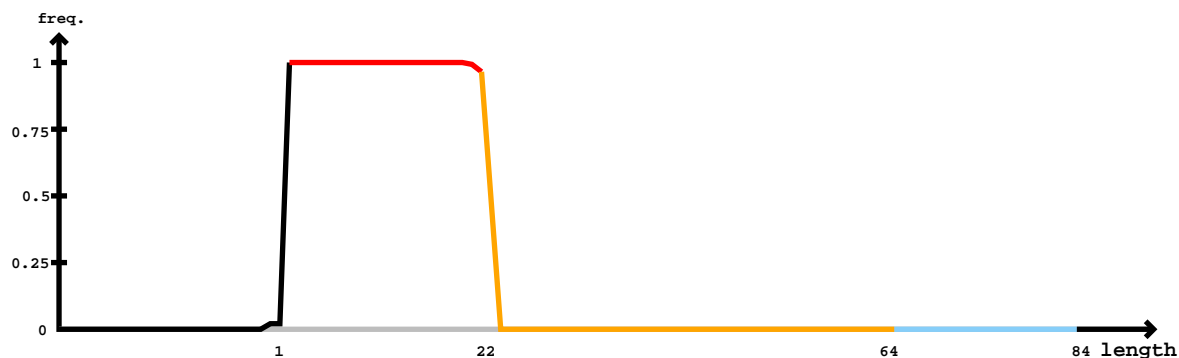

Star

[illegible]

Supplement: S2 Fig — Red colored letter: mature miRNA sequence; yellow colored letter: loop sequence; blue colored letter: miRNA* sequence. (ZIP) [file pone.0117507.s002.zip › Figures s1/contig1169132_10464.pdf]

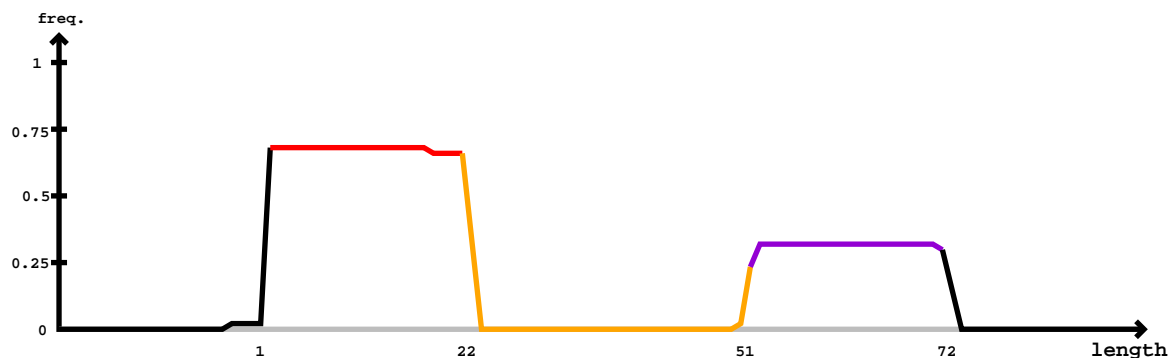

Star

[illegible]

Supplement: S2 Fig — Red colored letter: mature miRNA sequence; yellow colored letter: loop sequence; blue colored letter: miRNA* sequence. (ZIP) [file pone.0117507.s002.zip › Figures s1/contig1173624_10491.pdf]

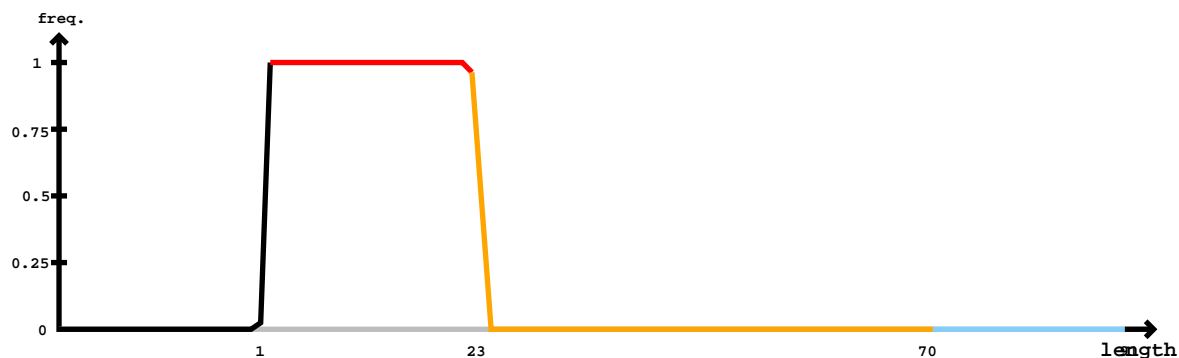

Star

[illegible]

Supplement: S2 Fig — Red colored letter: mature miRNA sequence; yellow colored letter: loop sequence; blue colored letter: miRNA* sequence. (ZIP) [file pone.0117507.s002.zip › Figures s1/contig1239786_10728.pdf]

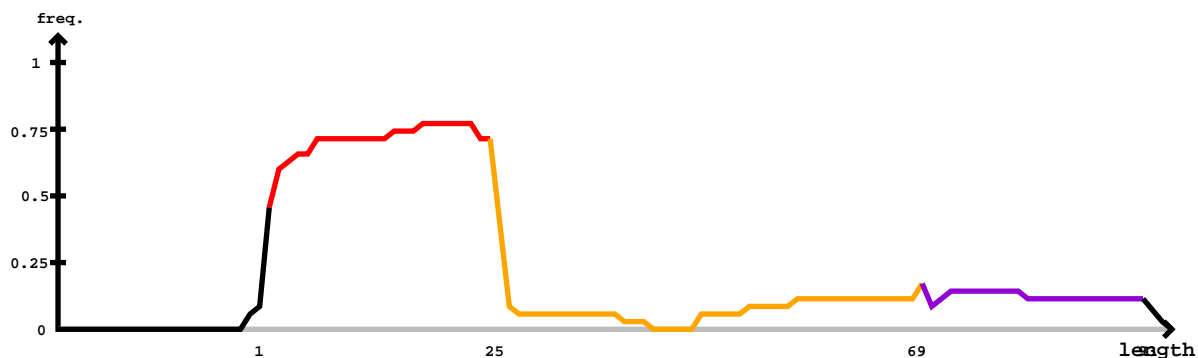

Star

[illegible]

Supplement: S2 Fig — Red colored letter: mature miRNA sequence; yellow colored letter: loop sequence; blue colored letter: miRNA* sequence. (ZIP) [file pone.0117507.s002.zip › Figures s1/contig1364498_11207.pdf]

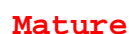

Supplement: S2 Fig — Red colored letter: mature miRNA sequence; yellow colored letter: loop sequence; blue colored letter: miRNA* sequence. (ZIP) [file pone.0117507.s002.zip › Figures s1/contig154805_3489.pdf]

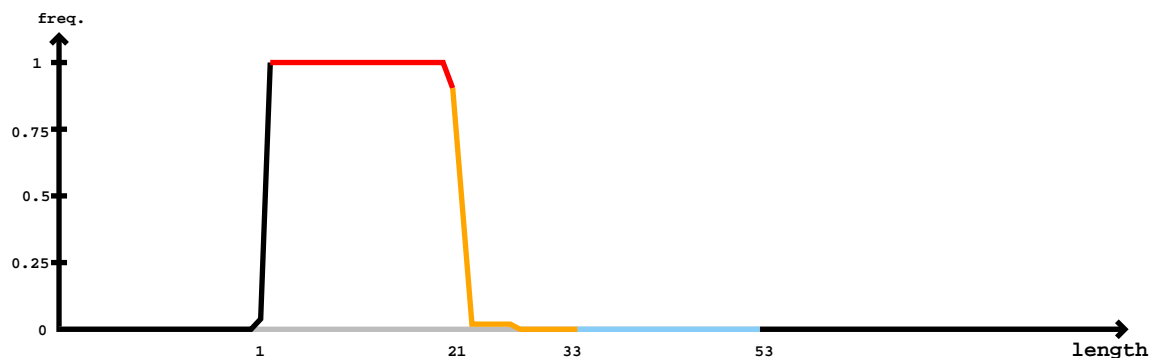

Star

[illegible]

Supplement: S2 Fig — Red colored letter: mature miRNA sequence; yellow colored letter: loop sequence; blue colored letter: miRNA* sequence. (ZIP) [file pone.0117507.s002.zip › Figures s1/contig1552523_11876.pdf]

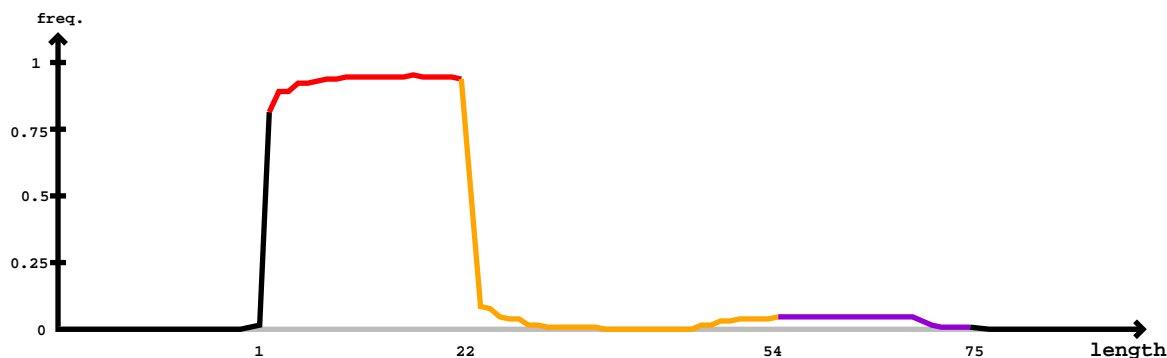

Star

[illegible]

Supplement: S2 Fig — Red colored letter: mature miRNA sequence; yellow colored letter: loop sequence; blue colored letter: miRNA* sequence. (ZIP) [file pone.0117507.s002.zip › Figures s1/contig157376_3524.pdf]

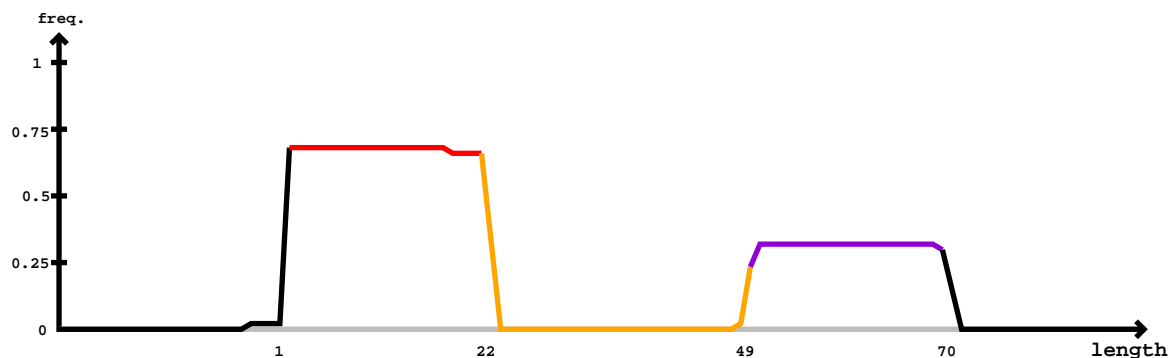

Star

[illegible]

Supplement: S2 Fig — Red colored letter: mature miRNA sequence; yellow colored letter: loop sequence; blue colored letter: miRNA* sequence. (ZIP) [file pone.0117507.s002.zip › Figures s1/contig169127_3668.pdf]

[illegible]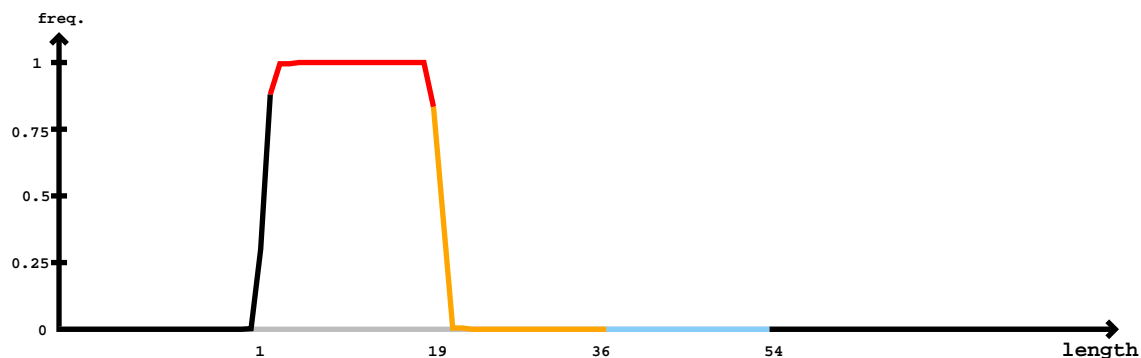

Star

[illegible]

Supplement: S2 Fig — Red colored letter: mature miRNA sequence; yellow colored letter: loop sequence; blue colored letter: miRNA* sequence. (ZIP) [file pone.0117507.s002.zip › Figures s1/contig172691_3710.pdf]

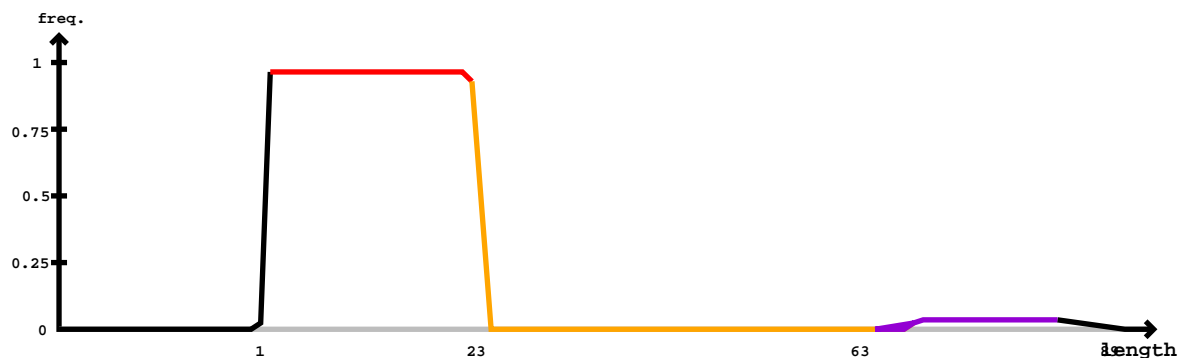

Star

[illegible]

Supplement: S2 Fig — Red colored letter: mature miRNA sequence; yellow colored letter: loop sequence; blue colored letter: miRNA* sequence. (ZIP) [file pone.0117507.s002.zip › Figures s1/contig176099_3752.pdf]

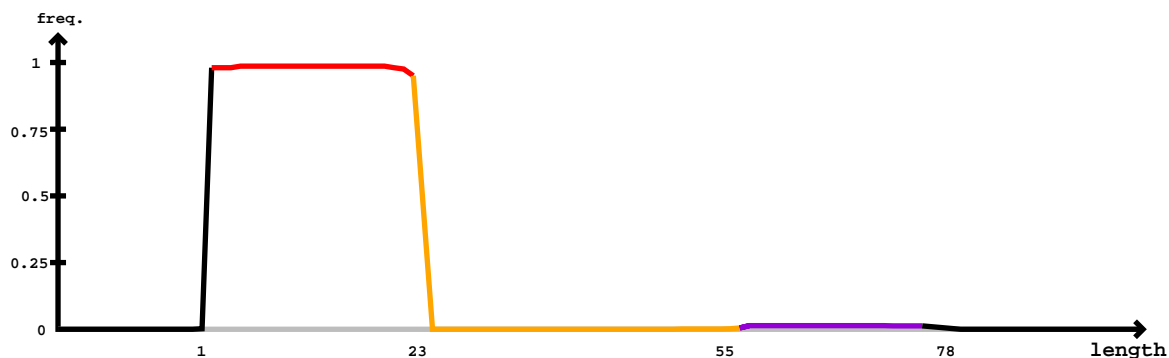

Star

[illegible]

Supplement: S2 Fig — Red colored letter: mature miRNA sequence; yellow colored letter: loop sequence; blue colored letter: miRNA* sequence. (ZIP) [file pone.0117507.s002.zip › Figures s1/contig1803535_12570.pdf]

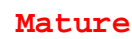[illegible]

Supplement: S2 Fig — Red colored letter: mature miRNA sequence; yellow colored letter: loop sequence; blue colored letter: miRNA* sequence. (ZIP) [file pone.0117507.s002.zip › Figures s1/contig1860742_12747.pdf]

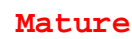[illegible]

Supplement: S2 Fig — Red colored letter: mature miRNA sequence; yellow colored letter: loop sequence; blue colored letter: miRNA* sequence. (ZIP) [file pone.0117507.s002.zip › Figures s1/contig187007_3905.pdf]

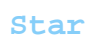

Supplement: S2 Fig — Red colored letter: mature miRNA sequence; yellow colored letter: loop sequence; blue colored letter: miRNA* sequence. (ZIP) [file pone.0117507.s002.zip › Figures s1/contig1885144_12835.pdf]

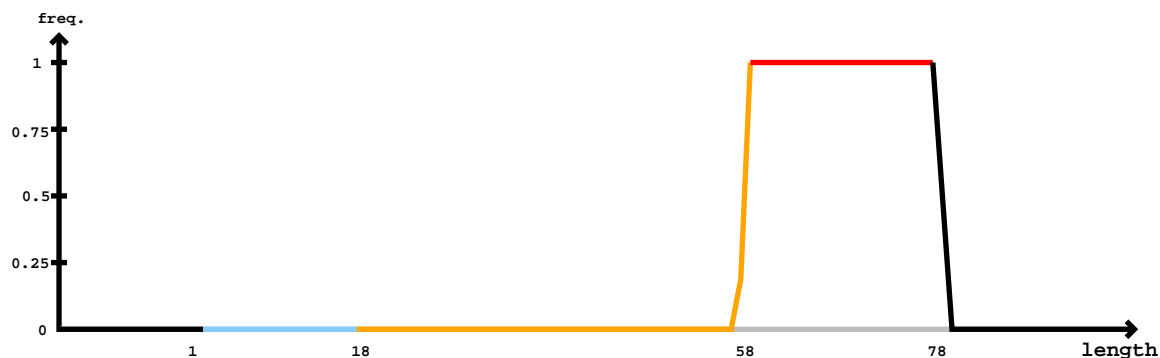

## Mature

Supplement: S2 Fig — Red colored letter: mature miRNA sequence; yellow colored letter: loop sequence; blue colored letter: miRNA* sequence. (ZIP) [file pone.0117507.s002.zip › Figures s1/contig1885144_12836.pdf]

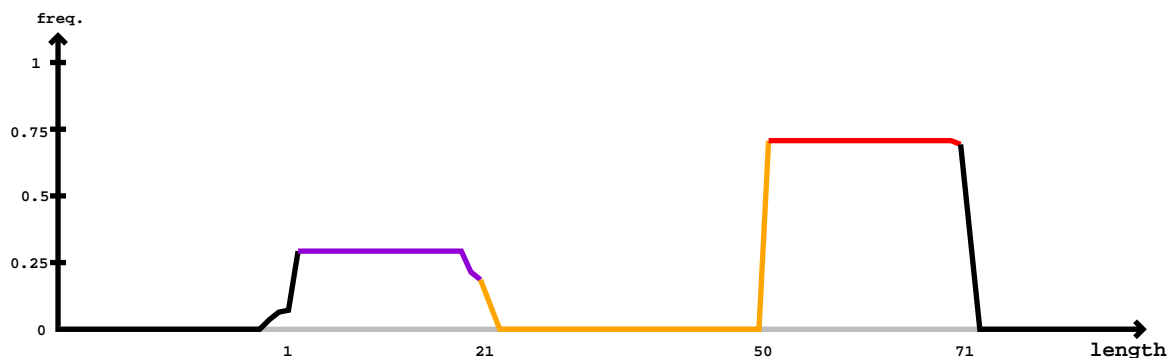

## Mature

[illegible]

Supplement: S2 Fig — Red colored letter: mature miRNA sequence; yellow colored letter: loop sequence; blue colored letter: miRNA* sequence. (ZIP) [file pone.0117507.s002.zip › Figures s1/contig1941326_12979.pdf]

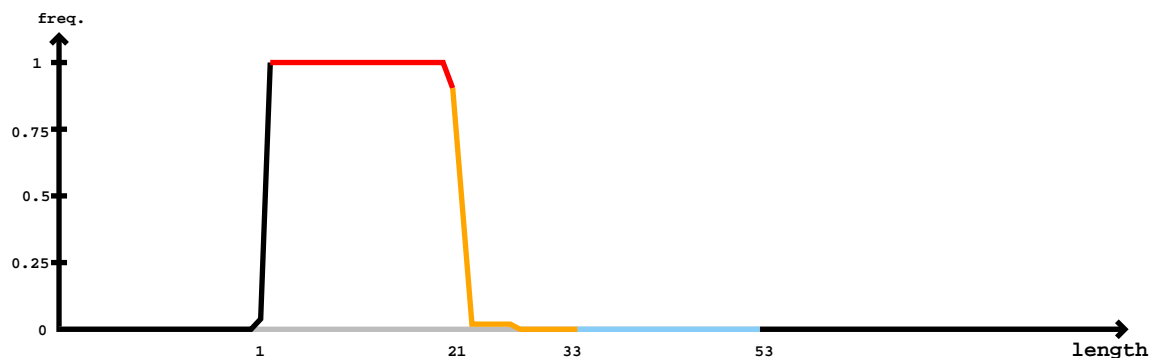

Star

[illegible]

Supplement: S2 Fig — Red colored letter: mature miRNA sequence; yellow colored letter: loop sequence; blue colored letter: miRNA* sequence. (ZIP) [file pone.0117507.s002.zip › Figures s1/contig205503_4134.pdf]

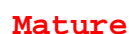[illegible]

Supplement: S2 Fig — Red colored letter: mature miRNA sequence; yellow colored letter: loop sequence; blue colored letter: miRNA* sequence. (ZIP) [file pone.0117507.s002.zip › Figures s1/contig2071105_13361.pdf]

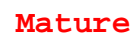[illegible]

Supplement: S2 Fig — Red colored letter: mature miRNA sequence; yellow colored letter: loop sequence; blue colored letter: miRNA* sequence. (ZIP) [file pone.0117507.s002.zip › Figures s1/contig2088896_13400.pdf]

[illegible]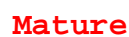[illegible]

Supplement: S2 Fig — Red colored letter: mature miRNA sequence; yellow colored letter: loop sequence; blue colored letter: miRNA* sequence. (ZIP) [file pone.0117507.s002.zip › Figures s1/contig2287307_13867.pdf]

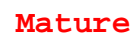[illegible]

Supplement: S2 Fig — Red colored letter: mature miRNA sequence; yellow colored letter: loop sequence; blue colored letter: miRNA* sequence. (ZIP) [file pone.0117507.s002.zip › Figures s1/contig2312229_13918.pdf]

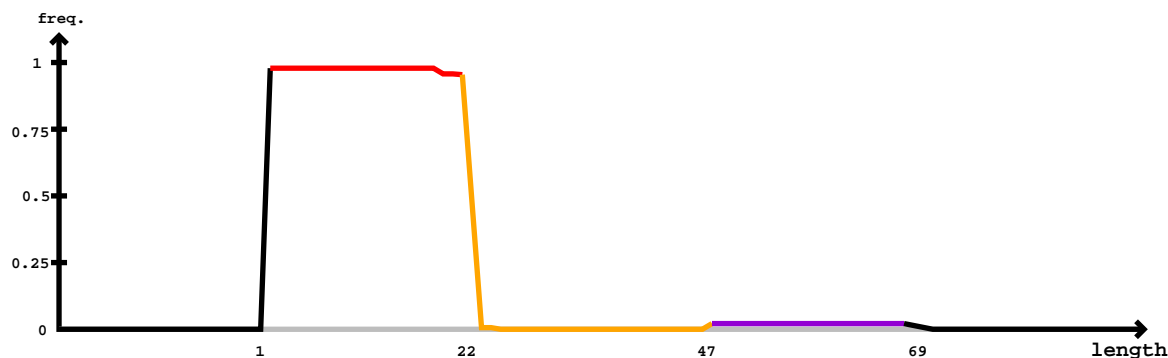

Star

[illegible]

Supplement: S2 Fig — Red colored letter: mature miRNA sequence; yellow colored letter: loop sequence; blue colored letter: miRNA* sequence. (ZIP) [file pone.0117507.s002.zip › Figures s1/contig254772_4721.pdf]

[illegible]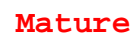[illegible]

Supplement: S2 Fig — Red colored letter: mature miRNA sequence; yellow colored letter: loop sequence; blue colored letter: miRNA* sequence. (ZIP) [file pone.0117507.s002.zip › Figures s1/contig2566467_14516.pdf]

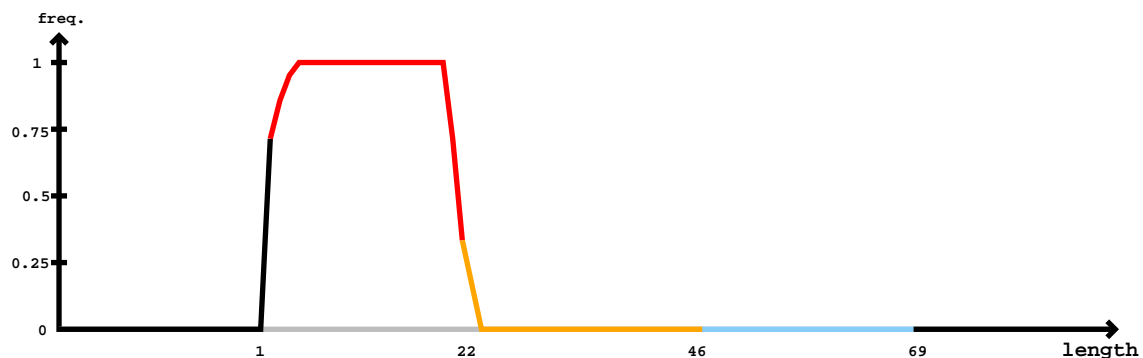

Star

[illegible]

Supplement: S2 Fig — Red colored letter: mature miRNA sequence; yellow colored letter: loop sequence; blue colored letter: miRNA* sequence. (ZIP) [file pone.0117507.s002.zip › Figures s1/contig2716339_14848.pdf]

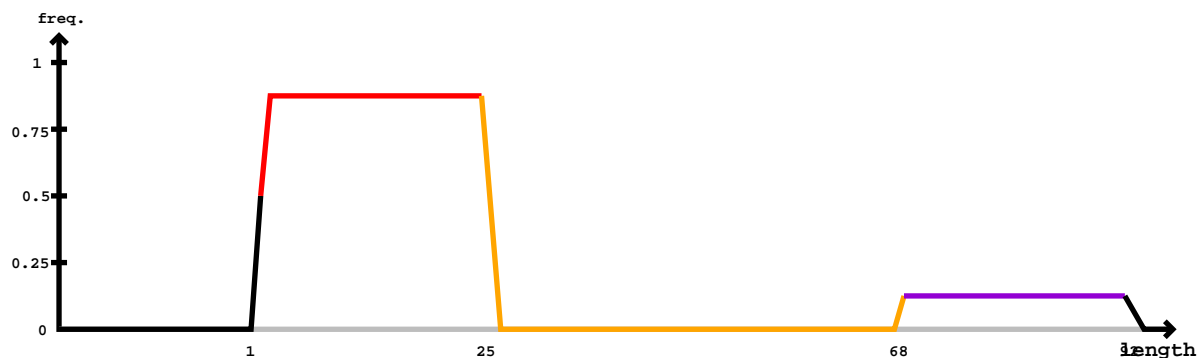

Star

[illegible]

Supplement: S2 Fig — Red colored letter: mature miRNA sequence; yellow colored letter: loop sequence; blue colored letter: miRNA* sequence. (ZIP) [file pone.0117507.s002.zip › Figures s1/contig2766119_14941.pdf]

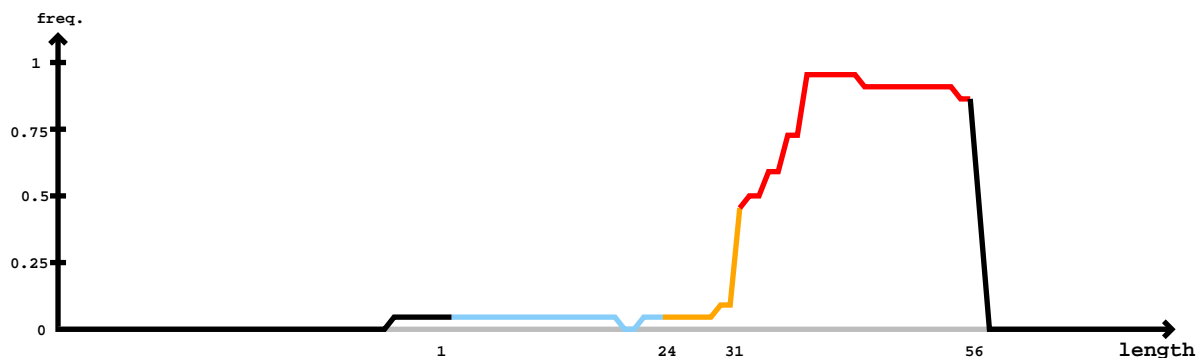

## Mature

[illegible]

Supplement: S2 Fig — Red colored letter: mature miRNA sequence; yellow colored letter: loop sequence; blue colored letter: miRNA* sequence. (ZIP) [file pone.0117507.s002.zip › Figures s1/contig2851415_15106.pdf]

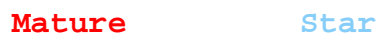[illegible]

Supplement: S2 Fig — Red colored letter: mature miRNA sequence; yellow colored letter: loop sequence; blue colored letter: miRNA* sequence. (ZIP) [file pone.0117507.s002.zip › Figures s1/contig293089_5094.pdf]

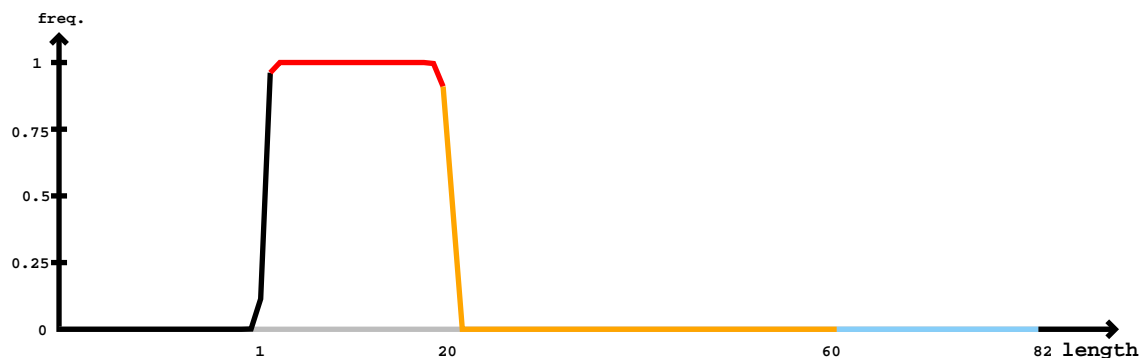

Star

[illegible]

Supplement: S2 Fig — Red colored letter: mature miRNA sequence; yellow colored letter: loop sequence; blue colored letter: miRNA* sequence. (ZIP) [file pone.0117507.s002.zip › Figures s1/contig2955072_15264.pdf]

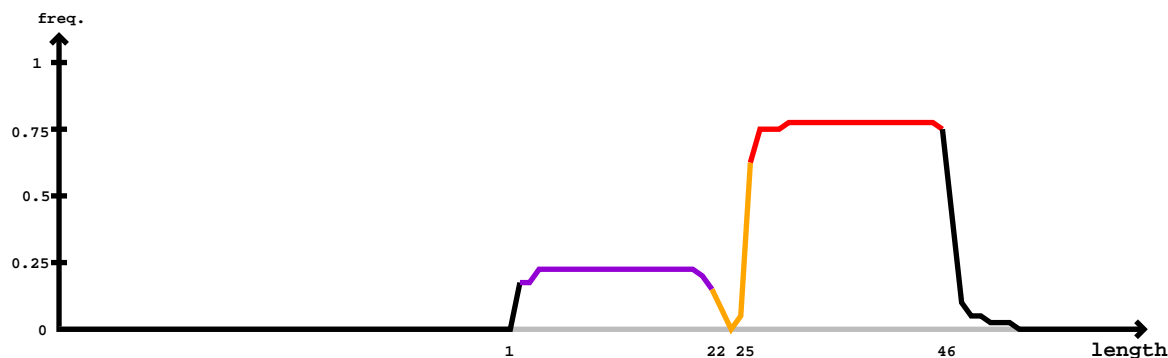[illegible]

Supplement: S2 Fig — Red colored letter: mature miRNA sequence; yellow colored letter: loop sequence; blue colored letter: miRNA* sequence. (ZIP) [file pone.0117507.s002.zip › Figures s1/contig2984958_15313.pdf]

[illegible]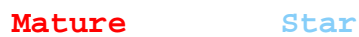[illegible]

Supplement: S2 Fig — Red colored letter: mature miRNA sequence; yellow colored letter: loop sequence; blue colored letter: miRNA* sequence. (ZIP) [file pone.0117507.s002.zip › Figures s1/contig2996054_15340.pdf]

[illegible]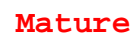[illegible]

Supplement: S2 Fig — Red colored letter: mature miRNA sequence; yellow colored letter: loop sequence; blue colored letter: miRNA* sequence. (ZIP) [file pone.0117507.s002.zip › Figures s1/contig3001237_15345.pdf]

[illegible]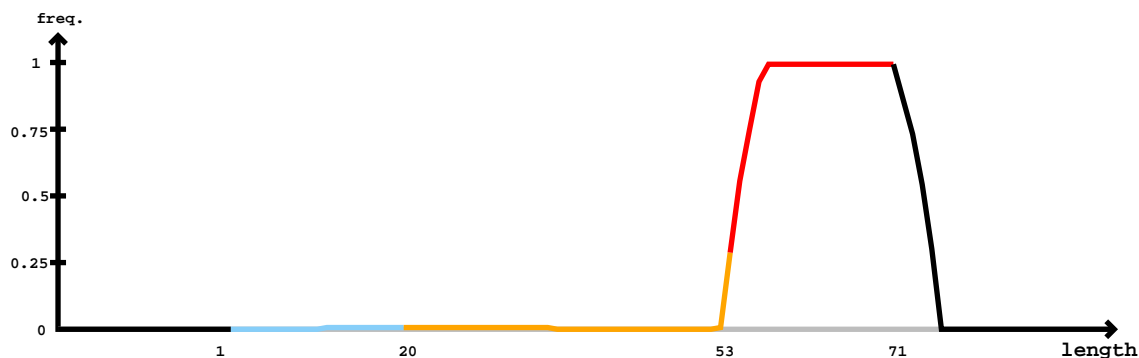[illegible]

Supplement: S2 Fig — Red colored letter: mature miRNA sequence; yellow colored letter: loop sequence; blue colored letter: miRNA* sequence. (ZIP) [file pone.0117507.s002.zip › Figures s1/contig3013472_15368.pdf]

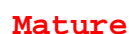[illegible]

Supplement: S2 Fig — Red colored letter: mature miRNA sequence; yellow colored letter: loop sequence; blue colored letter: miRNA* sequence. (ZIP) [file pone.0117507.s002.zip › Figures s1/contig3048700_15424.pdf]

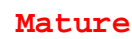[illegible]

Supplement: S2 Fig — Red colored letter: mature miRNA sequence; yellow colored letter: loop sequence; blue colored letter: miRNA* sequence. (ZIP) [file pone.0117507.s002.zip › Figures s1/contig3128711_15582.pdf]

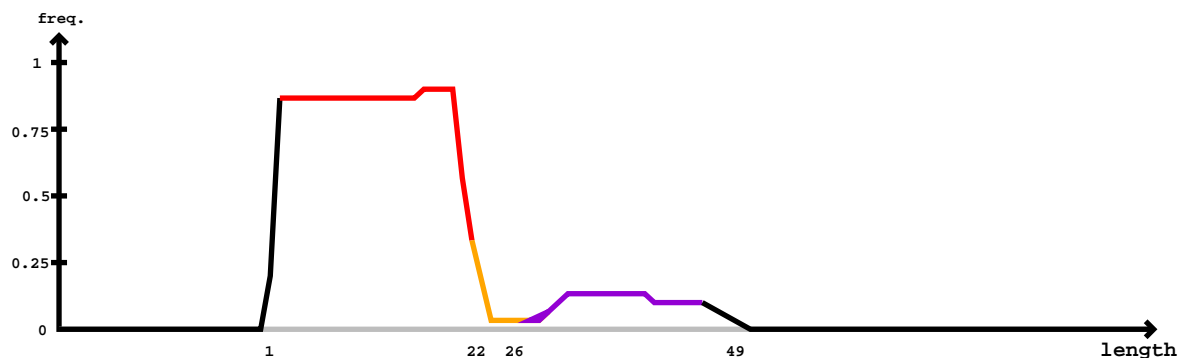[illegible]

Supplement: S2 Fig — Red colored letter: mature miRNA sequence; yellow colored letter: loop sequence; blue colored letter: miRNA* sequence. (ZIP) [file pone.0117507.s002.zip › Figures s1/contig3141743_15597.pdf]

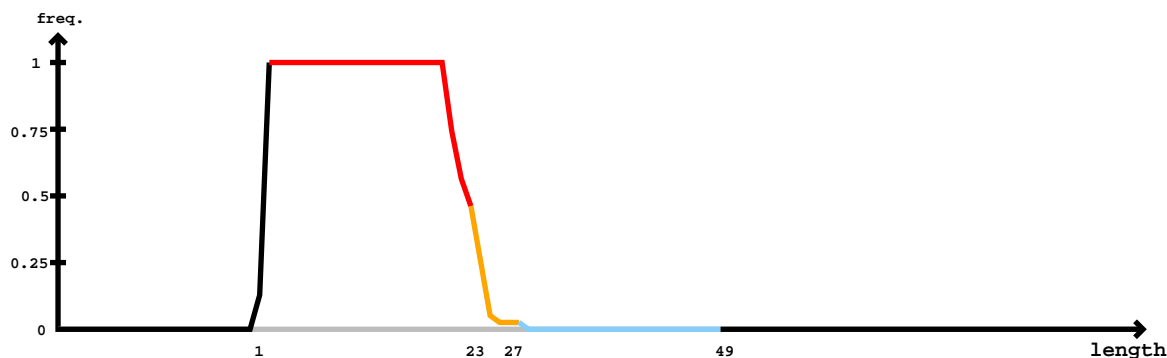[illegible]

Supplement: S2 Fig — Red colored letter: mature miRNA sequence; yellow colored letter: loop sequence; blue colored letter: miRNA* sequence. (ZIP) [file pone.0117507.s002.zip › Figures s1/contig3150844_15617.pdf]

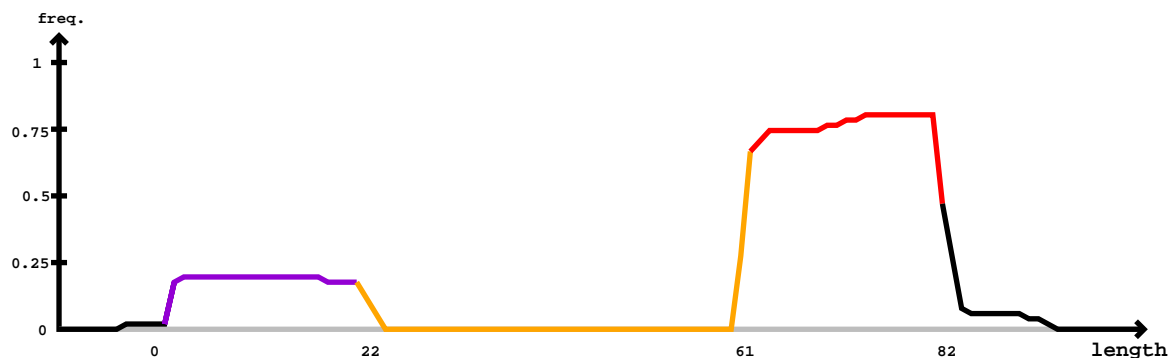

## Mature

[illegible]

Supplement: S2 Fig — Red colored letter: mature miRNA sequence; yellow colored letter: loop sequence; blue colored letter: miRNA* sequence. (ZIP) [file pone.0117507.s002.zip › Figures s1/contig3273262_15798.pdf]

[illegible]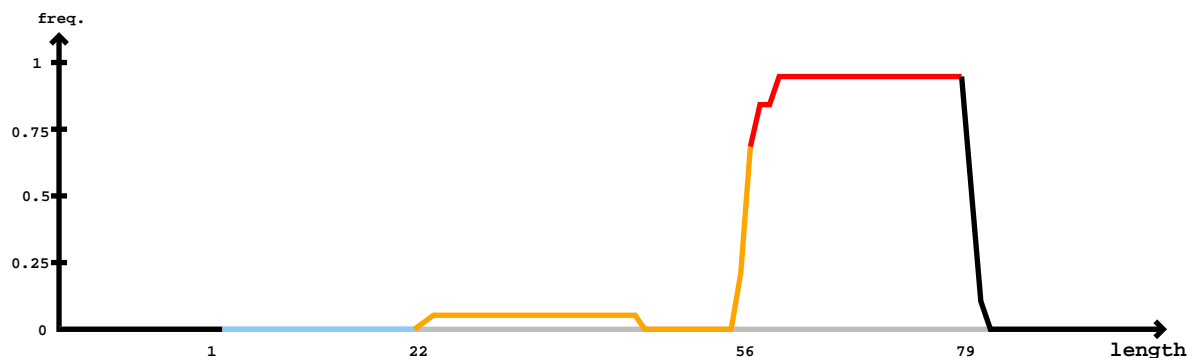

## Mature

[illegible]

Supplement: S2 Fig — Red colored letter: mature miRNA sequence; yellow colored letter: loop sequence; blue colored letter: miRNA* sequence. (ZIP) [file pone.0117507.s002.zip › Figures s1/contig3530906_16194.pdf]

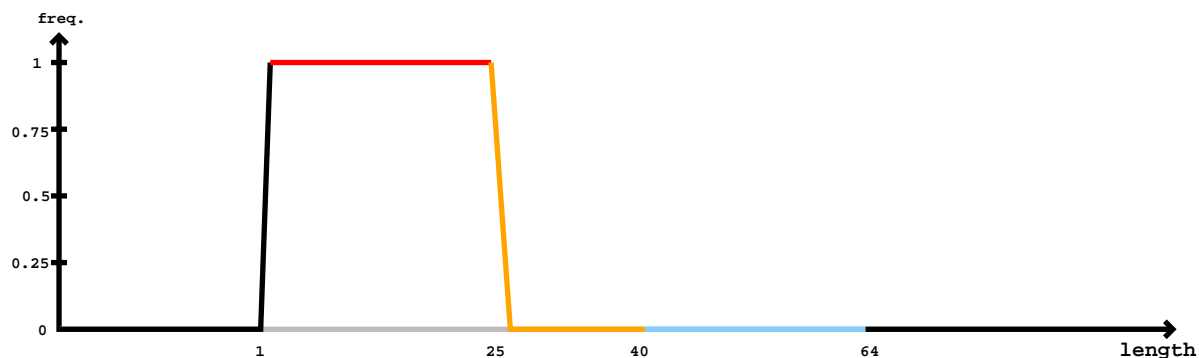

Star

[illegible]

Supplement: S2 Fig — Red colored letter: mature miRNA sequence; yellow colored letter: loop sequence; blue colored letter: miRNA* sequence. (ZIP) [file pone.0117507.s002.zip › Figures s1/contig3576723_16244.pdf]

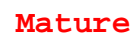[illegible]

Supplement: S2 Fig — Red colored letter: mature miRNA sequence; yellow colored letter: loop sequence; blue colored letter: miRNA* sequence. (ZIP) [file pone.0117507.s002.zip › Figures s1/contig3619587_16325.pdf]

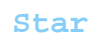[illegible]

Supplement: S2 Fig — Red colored letter: mature miRNA sequence; yellow colored letter: loop sequence; blue colored letter: miRNA* sequence. (ZIP) [file pone.0117507.s002.zip › Figures s1/contig3874161_16862.pdf]

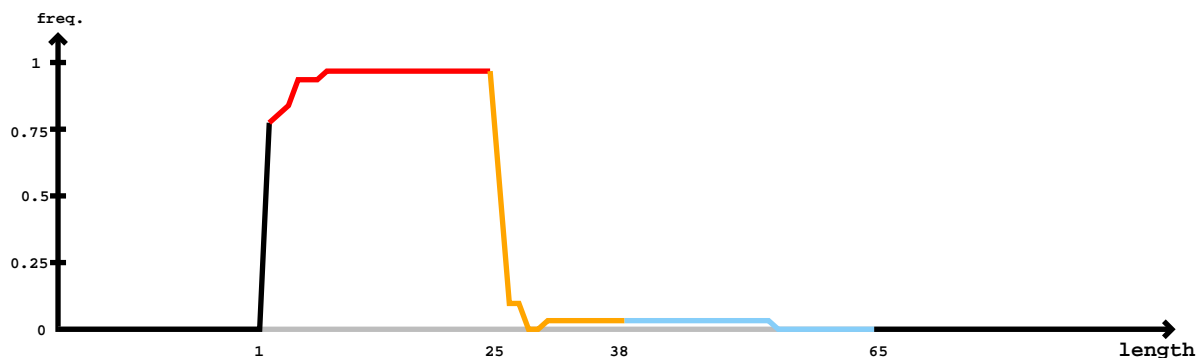

Star

Supplement: S2 Fig — Red colored letter: mature miRNA sequence; yellow colored letter: loop sequence; blue colored letter: miRNA* sequence. (ZIP) [file pone.0117507.s002.zip › Figures s1/contig389713_5984.pdf]

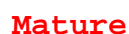[illegible]

Supplement: S2 Fig — Red colored letter: mature miRNA sequence; yellow colored letter: loop sequence; blue colored letter: miRNA* sequence. (ZIP) [file pone.0117507.s002.zip › Figures s1/contig4020558_17163.pdf]

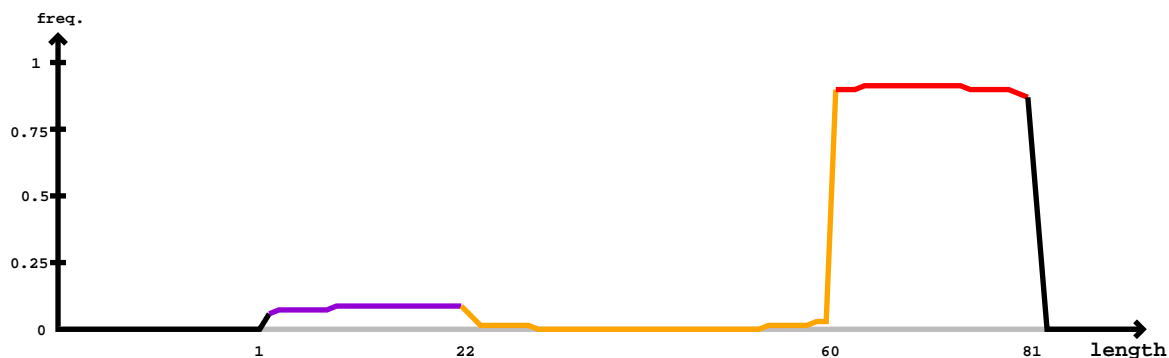

**Mature**

[illegible]

Supplement: S2 Fig — Red colored letter: mature miRNA sequence; yellow colored letter: loop sequence; blue colored letter: miRNA* sequence. (ZIP) [file pone.0117507.s002.zip › Figures s1/contig4055796_17218.pdf]

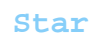[illegible]

Supplement: S2 Fig — Red colored letter: mature miRNA sequence; yellow colored letter: loop sequence; blue colored letter: miRNA* sequence. (ZIP) [file pone.0117507.s002.zip › Figures s1/contig412239_6168.pdf]

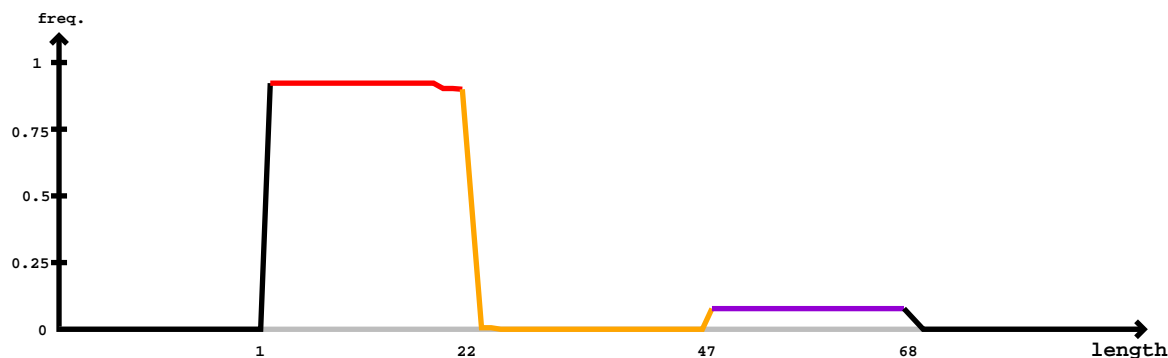

Star

[illegible]

Supplement: S2 Fig — Red colored letter: mature miRNA sequence; yellow colored letter: loop sequence; blue colored letter: miRNA* sequence. (ZIP) [file pone.0117507.s002.zip › Figures s1/contig4164108_17456.pdf]

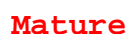[illegible]

Supplement: S2 Fig — Red colored letter: mature miRNA sequence; yellow colored letter: loop sequence; blue colored letter: miRNA* sequence. (ZIP) [file pone.0117507.s002.zip › Figures s1/contig422991_6267.pdf]

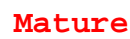[illegible]

Supplement: S2 Fig — Red colored letter: mature miRNA sequence; yellow colored letter: loop sequence; blue colored letter: miRNA* sequence. (ZIP) [file pone.0117507.s002.zip › Figures s1/contig423007_6272.pdf]

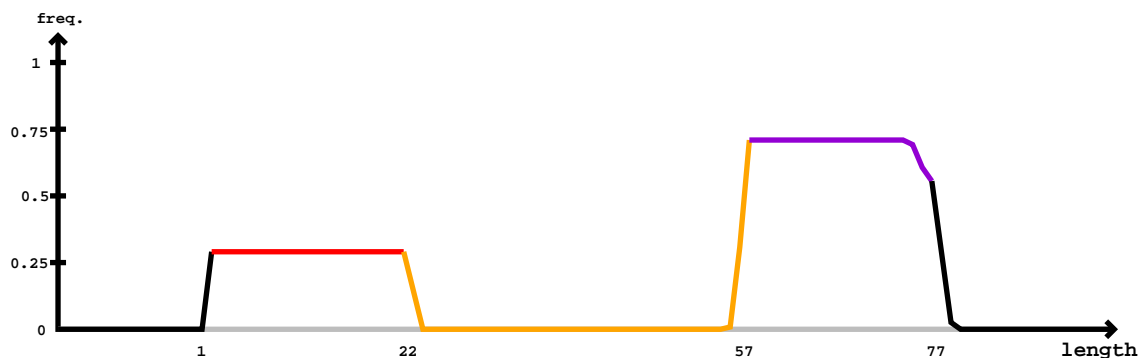

Star

[illegible]

Supplement: S2 Fig — Red colored letter: mature miRNA sequence; yellow colored letter: loop sequence; blue colored letter: miRNA* sequence. (ZIP) [file pone.0117507.s002.zip › Figures s1/contig4244526_17663.pdf]

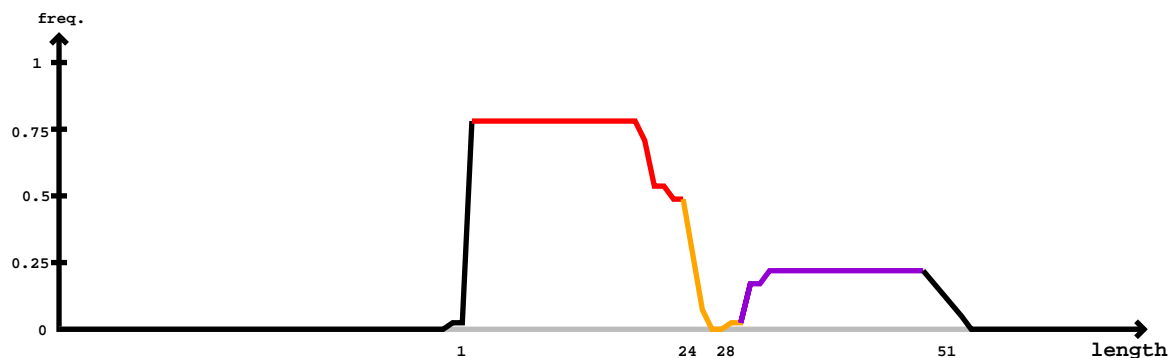

Mature Star

[illegible]

Supplement: S2 Fig — Red colored letter: mature miRNA sequence; yellow colored letter: loop sequence; blue colored letter: miRNA* sequence. (ZIP) [file pone.0117507.s002.zip › Figures s1/contig426428_6298.pdf]

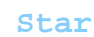[illegible]

Supplement: S2 Fig — Red colored letter: mature miRNA sequence; yellow colored letter: loop sequence; blue colored letter: miRNA* sequence. (ZIP) [file pone.0117507.s002.zip › Figures s1/contig4578333_18289.pdf]

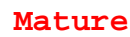[illegible]

Supplement: S2 Fig — Red colored letter: mature miRNA sequence; yellow colored letter: loop sequence; blue colored letter: miRNA* sequence. (ZIP) [file pone.0117507.s002.zip › Figures s1/contig4603203_18346.pdf]

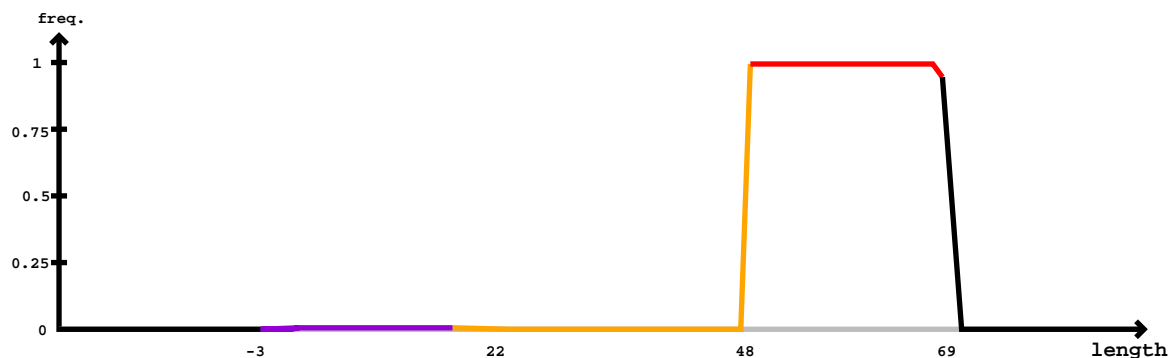

## Mature

[illegible]

Supplement: S2 Fig — Red colored letter: mature miRNA sequence; yellow colored letter: loop sequence; blue colored letter: miRNA* sequence. (ZIP) [file pone.0117507.s002.zip › Figures s1/contig4606075_18348.pdf]

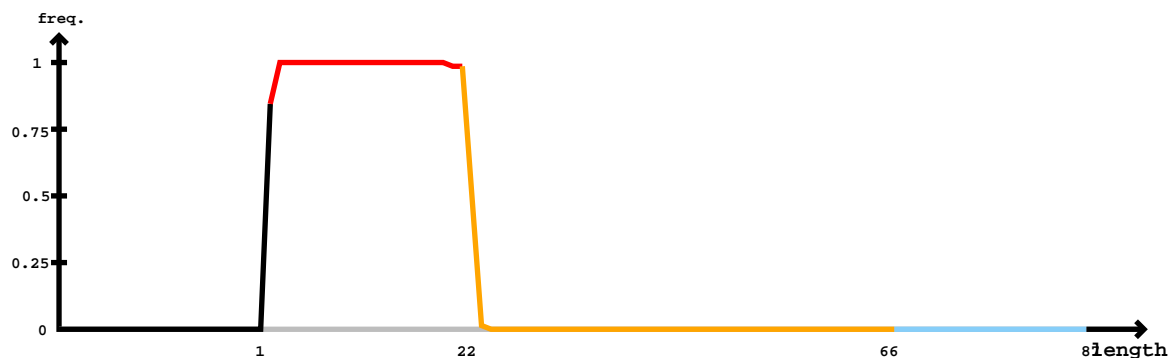

Star

[illegible]

Supplement: S2 Fig — Red colored letter: mature miRNA sequence; yellow colored letter: loop sequence; blue colored letter: miRNA* sequence. (ZIP) [file pone.0117507.s002.zip › Figures s1/contig4624450_18391.pdf]

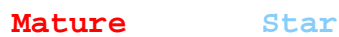[illegible]

Supplement: S2 Fig — Red colored letter: mature miRNA sequence; yellow colored letter: loop sequence; blue colored letter: miRNA* sequence. (ZIP) [file pone.0117507.s002.zip › Figures s1/contig4857495_18798.pdf]

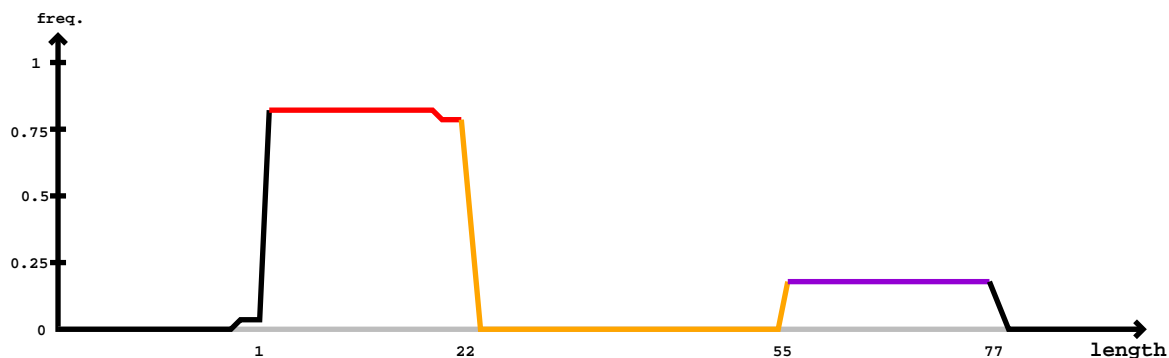

Star

[illegible]

Supplement: S2 Fig — Red colored letter: mature miRNA sequence; yellow colored letter: loop sequence; blue colored letter: miRNA* sequence. (ZIP) [file pone.0117507.s002.zip › Figures s1/contig4877233_18823.pdf]

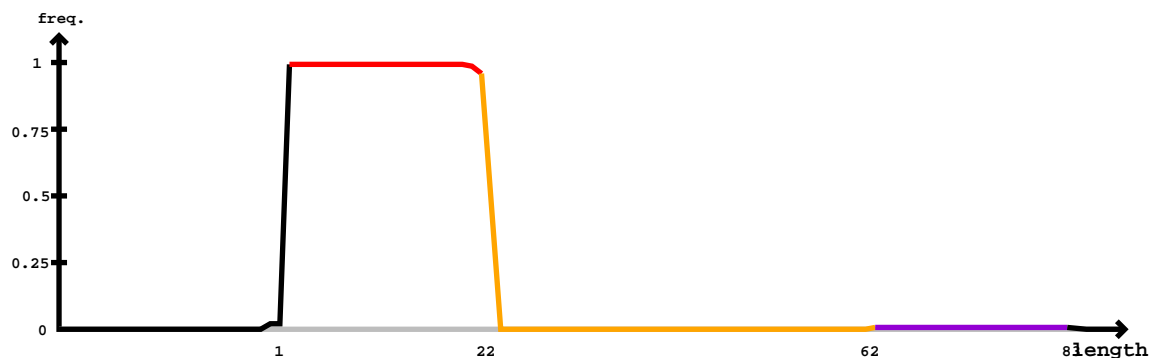

Star

[illegible]

Supplement: S2 Fig — Red colored letter: mature miRNA sequence; yellow colored letter: loop sequence; blue colored letter: miRNA* sequence. (ZIP) [file pone.0117507.s002.zip › Figures s1/contig515251_7046.pdf]

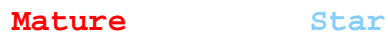[illegible]

Supplement: S2 Fig — Red colored letter: mature miRNA sequence; yellow colored letter: loop sequence; blue colored letter: miRNA* sequence. (ZIP) [file pone.0117507.s002.zip › Figures s1/contig537825_7191.pdf]

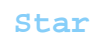[illegible]

Supplement: S2 Fig — Red colored letter: mature miRNA sequence; yellow colored letter: loop sequence; blue colored letter: miRNA* sequence. (ZIP) [file pone.0117507.s002.zip › Figures s1/contig542503_7224.pdf]

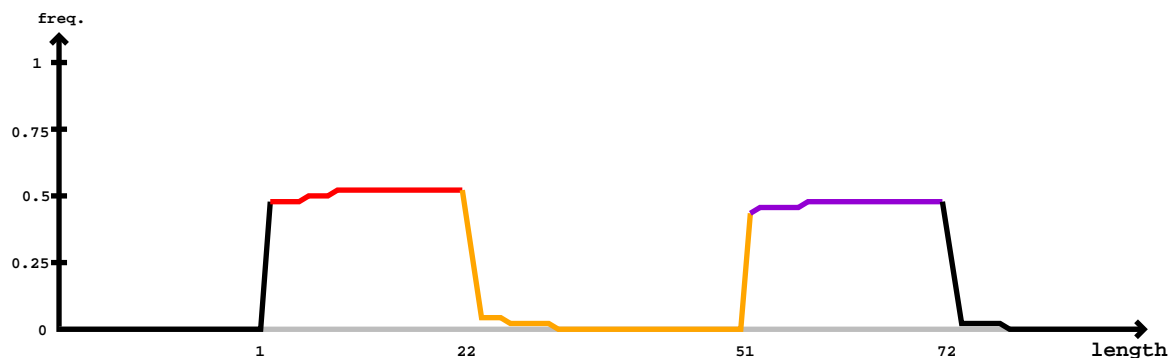

Star

[illegible]

Supplement: S2 Fig — Red colored letter: mature miRNA sequence; yellow colored letter: loop sequence; blue colored letter: miRNA* sequence. (ZIP) [file pone.0117507.s002.zip › Figures s1/contig59907_1840.pdf]

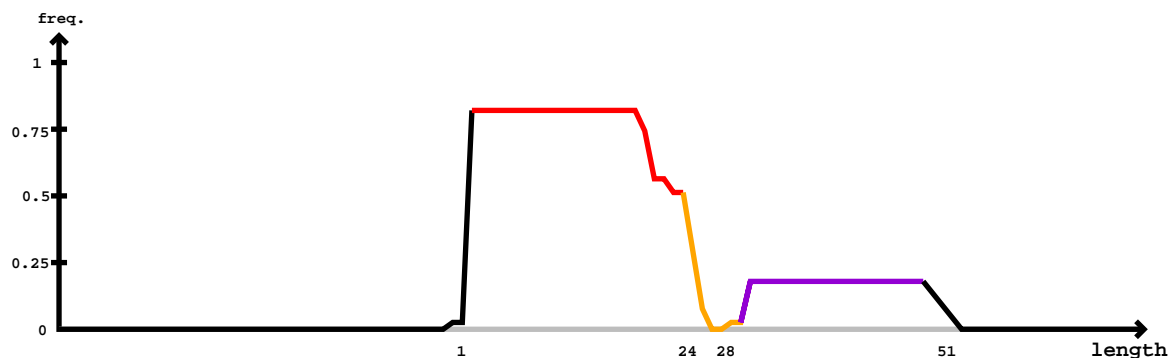[illegible]

Supplement: S2 Fig — Red colored letter: mature miRNA sequence; yellow colored letter: loop sequence; blue colored letter: miRNA* sequence. (ZIP) [file pone.0117507.s002.zip › Figures s1/contig599141_7583.pdf]

[illegible]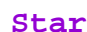[illegible]

Supplement: S2 Fig — Red colored letter: mature miRNA sequence; yellow colored letter: loop sequence; blue colored letter: miRNA* sequence. (ZIP) [file pone.0117507.s002.zip › Figures s1/contig600599_7592.pdf]

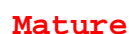[illegible]

Supplement: S2 Fig — Red colored letter: mature miRNA sequence; yellow colored letter: loop sequence; blue colored letter: miRNA* sequence. (ZIP) [file pone.0117507.s002.zip › Figures s1/contig608257_7658.pdf]

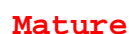[illegible]

Supplement: S2 Fig — Red colored letter: mature miRNA sequence; yellow colored letter: loop sequence; blue colored letter: miRNA* sequence. (ZIP) [file pone.0117507.s002.zip › Figures s1/contig632315_7817.pdf]

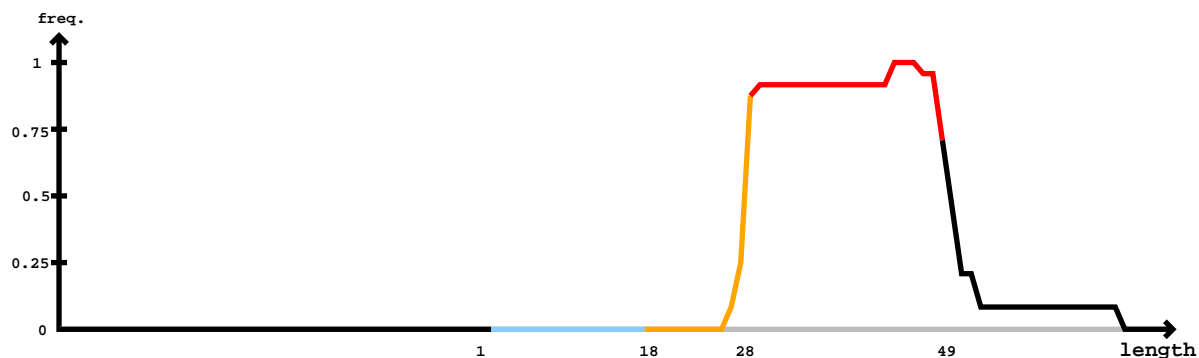

## Mature

[illegible]

Supplement: S2 Fig — Red colored letter: mature miRNA sequence; yellow colored letter: loop sequence; blue colored letter: miRNA* sequence. (ZIP) [file pone.0117507.s002.zip › Figures s1/contig63933_1966.pdf]

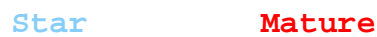[illegible]

Supplement: S2 Fig — Red colored letter: mature miRNA sequence; yellow colored letter: loop sequence; blue colored letter: miRNA* sequence. (ZIP) [file pone.0117507.s002.zip › Figures s1/contig66765_2007.pdf]

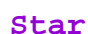[illegible]

Supplement: S2 Fig — Red colored letter: mature miRNA sequence; yellow colored letter: loop sequence; blue colored letter: miRNA* sequence. (ZIP) [file pone.0117507.s002.zip › Figures s1/contig700267_8204.pdf]

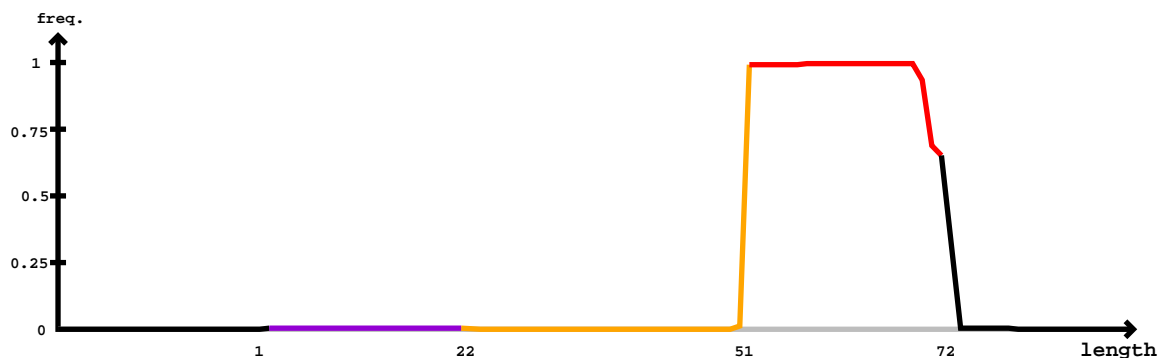

## Mature

[illegible]

Supplement: S2 Fig — Red colored letter: mature miRNA sequence; yellow colored letter: loop sequence; blue colored letter: miRNA* sequence. (ZIP) [file pone.0117507.s002.zip › Figures s1/contig70790_2116.pdf]

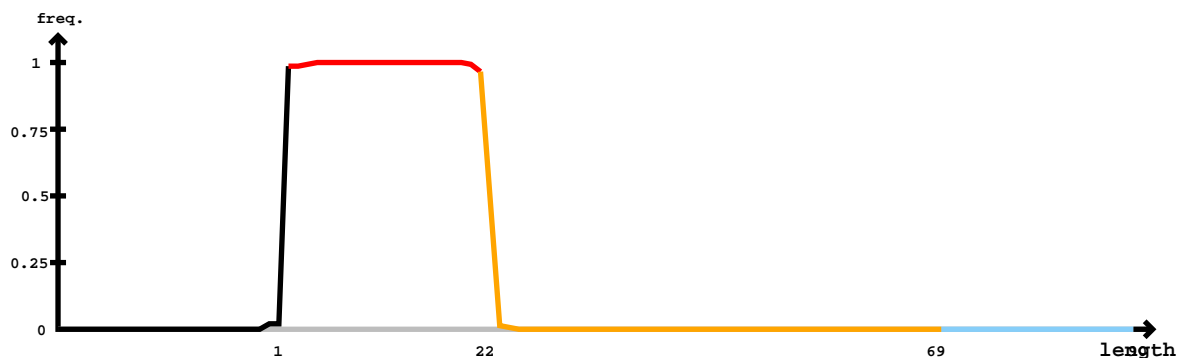

Star

[illegible]

Supplement: S2 Fig — Red colored letter: mature miRNA sequence; yellow colored letter: loop sequence; blue colored letter: miRNA* sequence. (ZIP) [file pone.0117507.s002.zip › Figures s1/contig767379_8575.pdf]

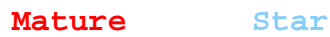[illegible]

Supplement: S2 Fig — Red colored letter: mature miRNA sequence; yellow colored letter: loop sequence; blue colored letter: miRNA* sequence. (ZIP) [file pone.0117507.s002.zip › Figures s1/contig768112_8578.pdf]

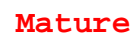[illegible]

Supplement: S2 Fig — Red colored letter: mature miRNA sequence; yellow colored letter: loop sequence; blue colored letter: miRNA* sequence. (ZIP) [file pone.0117507.s002.zip › Figures s1/contig773303_8615.pdf]

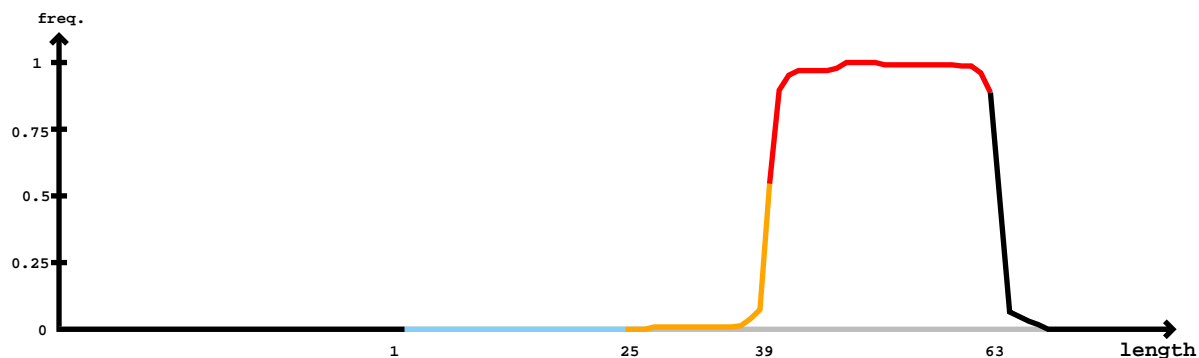

## Mature

[illegible]

Supplement: S2 Fig — Red colored letter: mature miRNA sequence; yellow colored letter: loop sequence; blue colored letter: miRNA* sequence. (ZIP) [file pone.0117507.s002.zip › Figures s1/contig814930_8846.pdf]

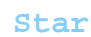[illegible]

Supplement: S2 Fig — Red colored letter: mature miRNA sequence; yellow colored letter: loop sequence; blue colored letter: miRNA* sequence. (ZIP) [file pone.0117507.s002.zip › Figures s1/contig94531_2504.pdf]

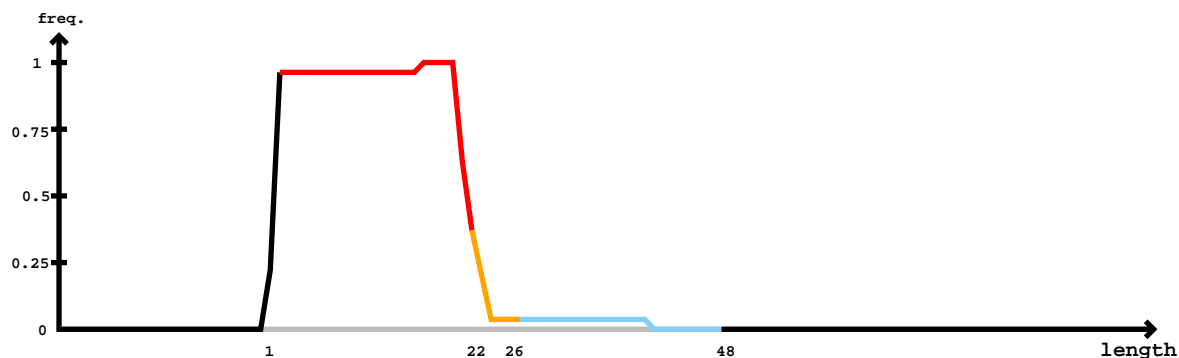[illegible]

Supplement: S2 Fig — Red colored letter: mature miRNA sequence; yellow colored letter: loop sequence; blue colored letter: miRNA* sequence. (ZIP) [file pone.0117507.s002.zip › Figures s1/contig95434_2517.pdf]

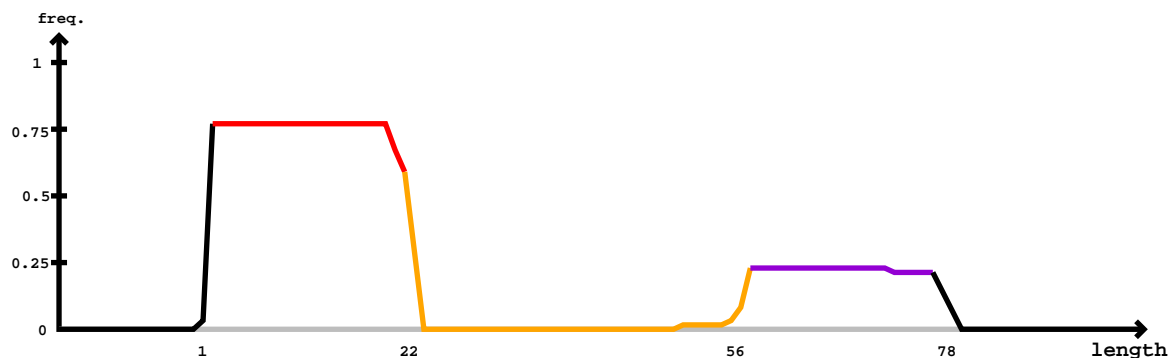

Star

[illegible]

Supplement: S2 Fig — Red colored letter: mature miRNA sequence; yellow colored letter: loop sequence; blue colored letter: miRNA* sequence. (ZIP) [file pone.0117507.s002.zip › Figures s1/contig958892_9544.pdf]

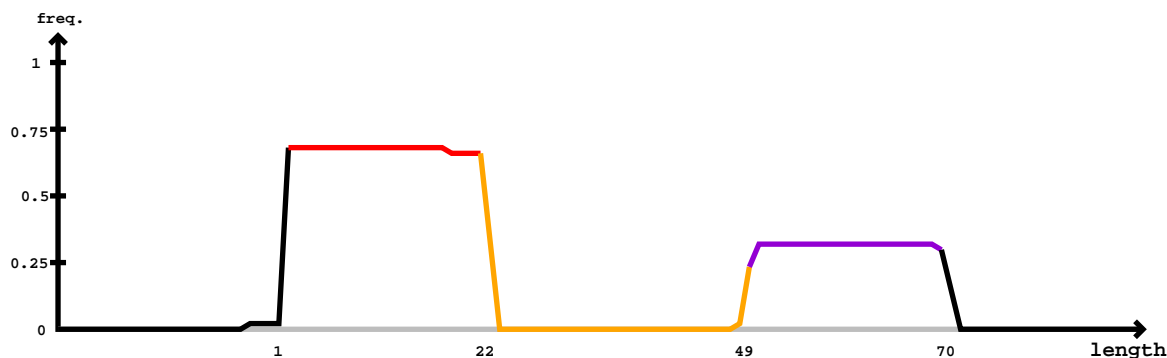

Star

[illegible]

Supplement: S2 Fig — Red colored letter: mature miRNA sequence; yellow colored letter: loop sequence; blue colored letter: miRNA* sequence. (ZIP) [file pone.0117507.s002.zip › Figures s1/contig98625_2582.pdf]
