# Supplementary material for: Identification of Novel miRNAs and miRNA Expression Profiling in Wheat Hybrid Necrosis
Source: PLoS One. 2015 Feb 23;10(2):e0117507. doi: 10.1371/journal.pone.0117507 (PMC4338152; doi:10.1371/journal.pone.0117507)
Supplement: S2 Fig — Red colored letter: mature miRNA sequence; yellow colored letter: loop sequence; blue colored letter: miRNA* sequence. (ZIP) [file pone.0117507.s002.zip › Figures s1/contig00619_67.pdf]

Provisional ID	:	contig00619_67
Score total	:	2.3
Score for star read(s)	:	-1.3
Score for read counts	:	0
Score for mfe	:	2.6
Score for randfold	:	1.6
Score for cons. seed	:	-0.6
Total read count	:	38
Mature read count	:	18
Loop read count	:	0
Star read count	:	20

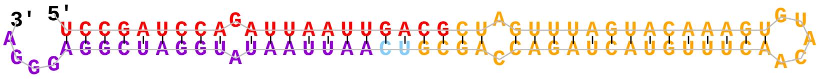

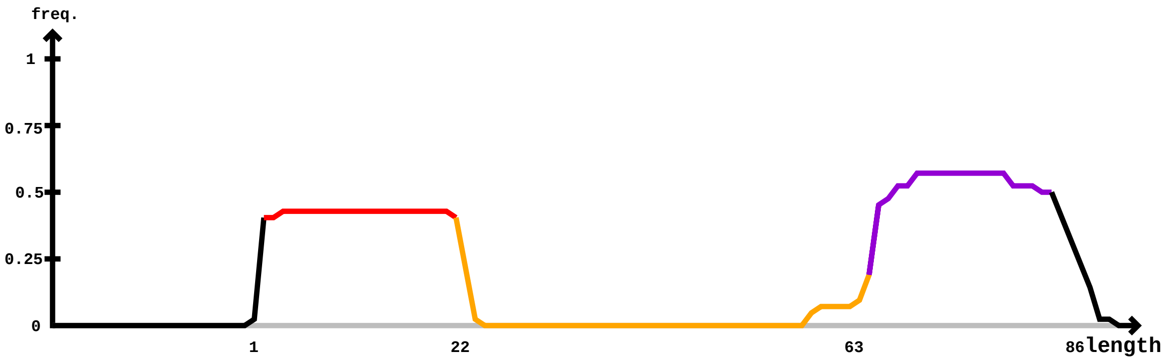


```
                         Mature                                                              Star
5'- gaagaaaaaacuuuacucacuccgauccagauuaauugacgcuaguuuaguacaaaguguacaacuuuguacuagaccagcgucaauuaauauggaucggagggaguaugu  -3'    obs
    gaagaaaaaacuuuacucacuccgauccagauuaauugacgcuaguuuaguacaaaguguacaacuuuguacuagaccagcgucaauuaauauggaucggagggaguaugu         exp
    .............(((((.((((((((((.(((((((((((((.((((((((((((((.....)))))))))))))).))))))))))))).)))))))))).)))))...   reads    mm     sample
    ....................uccgauccagauuaauugacg......................................................................   2        0      NN8
    ....................uccgauccaAauuaauugacg......................................................................   1        1      NN8
    ...................................................................................caauuaauauggaucggagggGg.....   1        1      NN8

    ...................cuccgauccaUauuaauugac.......................................................................   1        1      FF1
    ....................uccgauccaAauuaauugacg......................................................................   1        1      FF1
    ....................uccgauccGgauuaauugacg......................................................................   1        1      FF1
    ....................uccgauccagauuaauugacg......................................................................   10       0      FF1
    ....................uccgauccaUauuaauugacg......................................................................   1        1      FF1
    ......................cgGuccagauuaauugacgcu....................................................................   1        1      FF1
    .............................................................................cagcgucaauuaauauggauc.............   1        0      FF1
    .............................................................................Uagcgucaauuaauauggaucgga..........   1        1      FF1
    ..............................................................................agcgucaauuaauauggauc.............   1        0      FF1
    ..................................................................................ucaauuaauUuggaucggagggag.....   1        1      FF1
    ...................................................................................caauuaauUuggaucggaggg.......   1        1      FF1
    ...................................................................................caauuaauauggaucggagggag.....   1        0      FF1
    ...................................................................................caauuaauUuggaucggagggagu....   1        1      FF1
    ....................................................................................aauuaauauggaucggaggga......   6        0      FF1
    ....................................................................................aauuaauUuggaucggaggga......   1        1      FF1
    ....................................................................................aaGuaauauggaucggaggga......   1        1      FF1
    ....................................................................................aauuaauauggaucggagggaA.....   2        1      FF1
    ....................................................................................aauuaauUuggaucggagggagu....   1        1      FF1
    .....................................................................................auuaauauggaucgAaggga......   1        1      FF1
    ......................................................................................uuaauauggaucUgagggagu....   1        1      FF1
    ......................................................................................uuaauauggaucggagggagu....   1        0      FF1
    ........................................................................................aauUuggaucggagggagu....   1        1      FF1
    ........................................................................................aauauggaucggagggaguaG..   1        1      FF1
```
