# Supplementary material for: Identification of Novel miRNAs and miRNA Expression Profiling in Wheat Hybrid Necrosis
Source: PLoS One. 2015 Feb 23;10(2):e0117507. doi: 10.1371/journal.pone.0117507 (PMC4338152; doi:10.1371/journal.pone.0117507)
Supplement: S2 Fig — Red colored letter: mature miRNA sequence; yellow colored letter: loop sequence; blue colored letter: miRNA* sequence. (ZIP) [file pone.0117507.s002.zip › Figures s1/contig07156_419.pdf]

Provisional ID	:	contig07156_419
Score total	:	41
Score for star read(s)	:	3.9
Score for read counts	:	33.8
Score for mfe	:	2.3
Score for randfold	:	1.6
Score for cons. seed	:	-0.6
Total read count	:	78
Mature read count	:	52
Loop read count	:	0
Star read count	:	26

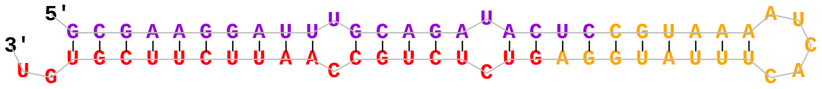

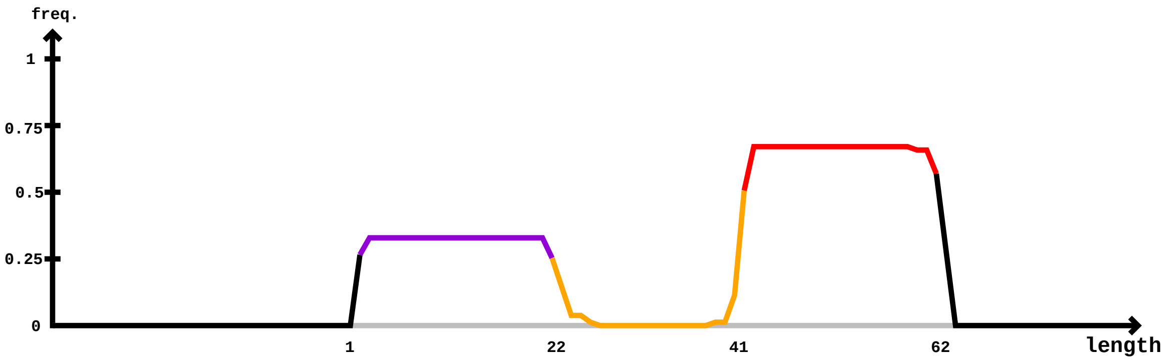

Star Mature

```
5'- aaguggcucaaagcaaagauugcgaagagcgcgaaggauuugcagauacuccguaaaaucacuuuauggagucucugccaauucuucguguucuuaacgucuuugcaguuu -3'   obs
    aaguggcucaaagcaaagauugcgaagagcgcgaaggauuugcagauacuccguaaaaucacuuuauggagucucugccaauucuucguguucuuaacgucuuugcaguuu        exp
    ............((((((((....((((((((((((((((.(((((.((((((((((.....)))))))))).))))).))))))))))))))))...)))))))).....   reads   mm   sample
    ..............................gcgaaggauuugcagauacu.............................................................   3       0    NN8
    ..............................gcgaaggauuugcagauacuc............................................................   3       0    NN8
    ..............................gcgaaggauuugcagauacuccgu.........................................................   1       0    NN8
    ..............................gcgaaggauuugcagauacuccgua........................................................   1       0    NN8
    ......................................................................gucucugccaauucuucgugu....................   1       0    NN8

    ..............................gcgaaggauuugcagauacu.............................................................   3       0    FF1
    ..............................gcgaaggauuugcagauacuU............................................................   1       1    FF1
    ..............................gcgaaggauuugcagauacuc............................................................   7       0    FF1
    ..............................gcgaaggauuugAagauacuc............................................................   1       1    FF1
    ..............................gcgaaggauuugcagauacuccgu.........................................................   1       0    FF1
    ...............................cgaaggauuugcagauacuc............................................................   1       0    FF1
    ...............................cgaaggauuugcagauacucc...........................................................   4       0    FF1
    ...................................................................ggagucucugccGauucuucgugu....................   1       1    FF1
    .....................................................................agucucugccaauucuucg.......................   1       0    FF1
    .....................................................................agucucugccaauucuucgug.....................   7       0    FF1
    ......................................................................gucucugccaauucuucgugu....................   18      0    FF1
    ......................................................................gucucugccaauucuucgugG....................   3       1    FF1
    ......................................................................gucucugccaauucuucguguA...................   1       1    FF1
    ......................................................................gucucugccaauucuucguguu...................   8       0    FF1
    .......................................................................ucucugccaauucuucgugu....................   1       0    FF1
    .......................................................................ucucugccaauucuucgGguu...................   1       1    FF1
    .......................................................................ucucugccaauucuucguguu...................   11      0    FF1
```
