# Supplementary material for: Identification of Novel miRNAs and miRNA Expression Profiling in Wheat Hybrid Necrosis
Source: PLoS One. 2015 Feb 23;10(2):e0117507. doi: 10.1371/journal.pone.0117507 (PMC4338152; doi:10.1371/journal.pone.0117507)
Supplement: S2 Fig — Red colored letter: mature miRNA sequence; yellow colored letter: loop sequence; blue colored letter: miRNA* sequence. (ZIP) [file pone.0117507.s002.zip › Figures s1/contig106305_2702.pdf]

Provisional ID	:	contig106305_2702
Score total	:	1196.9
Score for star read(s)	:	3.9
Score for read counts	:	1186
Score for mfe	:	2.4
Score for randfold	:	1.6
Score for cons. seed	:	3
Total read count	:	2338
Mature read count	:	2265
Loop read count	:	0
Star read count	:	73

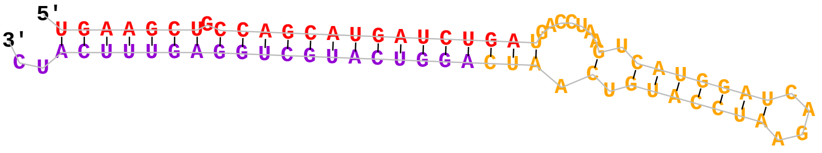

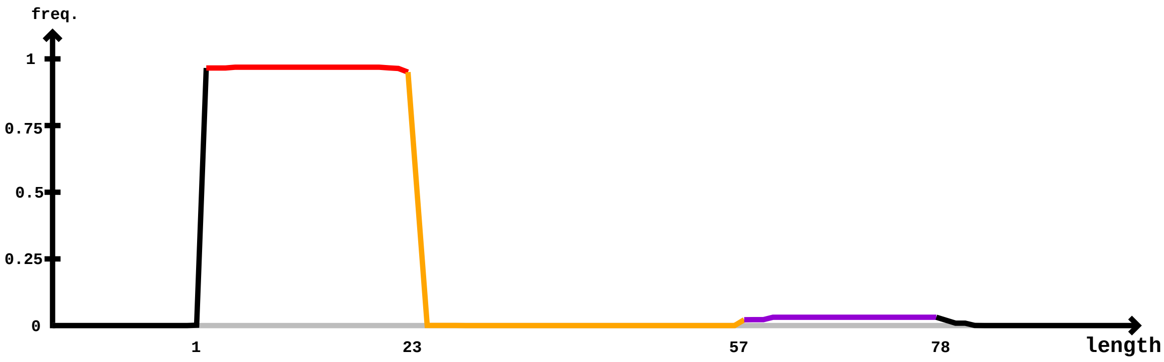

Mature **Star**

```
5'- gcaccacaagcuggugaagcugccagcaugaucugaugaccuaaagucauggaucagaauccaugucaaucaggucaugcuggaguuucaucugcuggucggagcacaacga   -3'   obs
    gcaccacaagcuggugaagcugccagcaugaucugaugaccuaaagucauggaucagaauccaugucaaucaggucaugcuggaguuucaucugcuggucggagcacaacga         exp
    ((.((((.(((.(((((((((.(((((((((((((((........(.(((((((....))))))).).)))))))))))))))))))))))).))).)).)).))....... reads   mm   sample
    ..............ugaagcugccagcaugaucugC............................................................................ 20      1    NN8
    ..............ugaagcugccagcaugaucuga............................................................................ 15      0    NN8
    .................agcugccagcaugaucuga............................................................................ 1       0    NN8
    .................agcugccagcaugaucugC............................................................................ 6       1    NN8
    ........................................................................ggucaugcuggaguuucauc.................... 1       0    NN8

    .............Cugaagcugccagcaugauc............................................................................... 2       1    FF1
    .............Cugaagcugccagcaugaucug............................................................................. 1       1    FF1
    ..............ugaagcugccagcaugauc............................................................................... 4       0    FF1
    ..............ugaagcugccagcaugaucu.............................................................................. 4       0    FF1
    ..............ugaagcugccagcaugaucA.............................................................................. 1       1    FF1
    ..............ugaagcugccagcaugaucuC............................................................................. 1       1    FF1
    ..............ugaagcugccagcauAaucug............................................................................. 1       1    FF1
    ..............ugaagcugccagcaugaucug............................................................................. 28      0    FF1
    ..............ugaagcugccagcaugaucuU............................................................................. 1       1    FF1
    ..............uUaagcugccagcaugaucuga............................................................................ 1       1    FF1
    ..............ugaagcugccGgcaugaucuga............................................................................ 1       1    FF1
    ..............Ggaagcugccagcaugaucuga............................................................................ 4       1    FF1
    ..............ugaagcugccagcaugaGcuga............................................................................ 1       1    FF1
    ..............ugaagcugAcagcaugaucuga............................................................................ 1       1    FF1
    ..............ugaagcugccagcaugaucugU............................................................................ 10      1    FF1
    ..............ugaagcugccagAaugaucuga............................................................................ 3       1    FF1
    ..............ugaagcGgccagcaugaucuga............................................................................ 2       1    FF1
    ..............ugaagcugccagcaugaucugG............................................................................ 5       1    FF1
    ..............ugaagcugccagcaugaucuga............................................................................ 1179    0    FF1
    ..............ugaagcugccagcauUaucuga............................................................................ 3       1    FF1
    ..............ugaagcugccagUaugaucuga............................................................................ 1       1    FF1
    ..............ugaagcugccaUcaugaucuga............................................................................ 2       1    FF1
    ..............ugaagcugccagcauAaucuga............................................................................ 1       1    FF1
    ..............ugaagcugccagcaugaucugC............................................................................ 949     1    FF1
    ..............ugaagcugccagcaugaAcuga............................................................................ 1       1    FF1
    ..............Cgaagcugccagcaugaucuga............................................................................ 1       1    FF1
    ..............ugaagcugccagcaugUucuga............................................................................ 1       1    FF1
```

Mature   Star

```
gcaccacaagcuggugaagcugccagcaugaucugaugaccuaagucauggaucagaauccaugucaaucaggucaugcuggaguuucaucugcuggucggagcacaacga
..............ugaagcugccaAcaugaucuga...........................................................................	1	1	FF1
..............ugaUgcugccagcaugaucuga...........................................................................	1	1	FF1
..............uAaagcugccagcaugaucuga...........................................................................	1	1	FF1
..............ugaagcugccagcaCgaucuga...........................................................................	1	1	FF1
..............ugaagcugccagGaugaucuga...........................................................................	3	1	FF1
..............ugaagcugccagcGugaucuga...........................................................................	1	1	FF1
..............ugaagcugccagcaugaucugaA..........................................................................	5	1	FF1
..............ugaagcugccagcaugaucugaugacc......................................................................	1	0	FF1
......................................................................aggucaugcuggaguuucauc....................	48	0	FF1
......................................................................aggucaugcuggGguuucauc....................	1	1	FF1
......................................................................aggucaugcugAaguuucauc....................	2	1	FF1
.........................................................................ucaugcuggaguuucaucugc.................	19	0	FF1
.........................................................................ucaugcuggaguGucaucugc.................	1	1	FF1
.........................................................................ucaugcuggaguuucaucugcA................	1	1	FF1
```
