# Supplementary material for: Identification of Novel miRNAs and miRNA Expression Profiling in Wheat Hybrid Necrosis
Source: PLoS One. 2015 Feb 23;10(2):e0117507. doi: 10.1371/journal.pone.0117507 (PMC4338152; doi:10.1371/journal.pone.0117507)
Supplement: S2 Fig — Red colored letter: mature miRNA sequence; yellow colored letter: loop sequence; blue colored letter: miRNA* sequence. (ZIP) [file pone.0117507.s002.zip › Figures s1/contig106724_2720.pdf]

Provisional ID	:	contig106724_2720
Score total	:	3884.7
Score for star read(s)	:	3.9
Score for read counts	:	3873.8
Score for mfe	:	2.4
Score for randfold	:	1.6
Score for cons. seed	:	3
Total read count	:	7610
Mature read count	:	4810
Loop read count	:	0
Star read count	:	2800

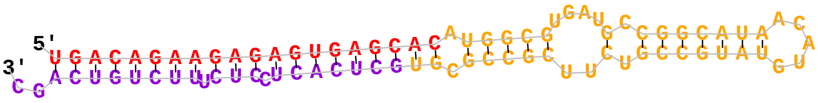

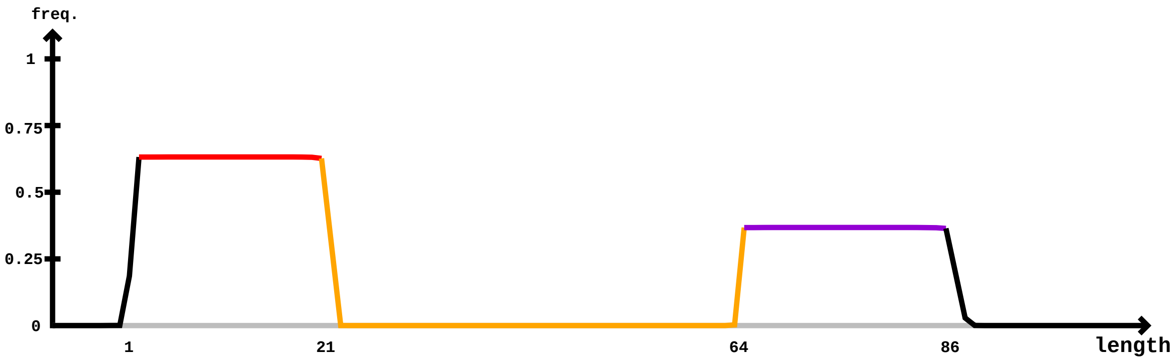

Mature **Star**

```
5'- gagagauugacagaagagagugagcacauggcgugaugccggcauaacauguaugccgucuucgccgcgugcucacuccucuuucugucagccucuuucucucaggaguuua  -3'   obs
    gagagauugacagaagagagugagcacauggcgugaugccggcauaacauguaugccgucuucgccgcgugcucacuccucuuucugucagccucuuucucucaggaguuua        exp
    ((((..((((((((((((((((((.(((((....(.(((((((.....))))))).)..))))).))))))))).))).))))))))..))))).(((....)))....   reads   mm   sample
    ......uugacagaagagagugaUcac.....................................................................................   1       1    NN8
    ......uugacagaagagagugagcac.....................................................................................   12      0    NN8
    ......uugacagaagagagugagcacU....................................................................................   1       1    NN8
    .......ugacagaagagagugagc.......................................................................................   1       0    NN8
    .......ugacagaagagagugagca......................................................................................   2       0    NN8
    .......ugacagaagagagugUgcac.....................................................................................   1       1    NN8
    .......ugacagaagagagugagcac.....................................................................................   135     0    NN8
    .......ugacagaagagGgugagcac.....................................................................................   1       1    NN8
    .......Agacagaagagagugagcac.....................................................................................   1       1    NN8
    .......ugacagaagagagugagcaU.....................................................................................   1       1    NN8
    .......ugacagaagagagugagcacU....................................................................................   11      1    NN8
    .........acagaagagagugagcac.....................................................................................   1       0    NN8
    ......................................................................gcucacuccucuuucuguc.......................   1       0    NN8
    ......................................................................gcucacuccucuuucuguca......................   3       0    NN8
    ......................................................................gcucacuccucuuucugucag.....................   7       0    NN8
    ......................................................................gcucacuccucuuuGugucag.....................   1       1    NN8
    ......................................................................gcucacuccucuuucuguGagc....................   1       1    NN8
    ......................................................................gcucacGccucuuucugucagc....................   1       1    NN8
    ......................................................................gcucacuccucuuucuguUagc....................   2       1    NN8
    ......................................................................gcucacuccucuuGcugucagc....................   2       1    NN8
    ......................................................................gcucacuccucuCucugucagc....................   3       1    NN8
    ......................................................................gcuGacuccucuuucugucagc....................   1       1    NN8
    ......................................................................gAucacuccucuuucugucagc....................   1       1    NN8
    ......................................................................gcucacuccuUuuucugucagc....................   1       1    NN8
    ......................................................................gcucCcuccucuuucugucagc....................   1       1    NN8
    ......................................................................gcucacuccucuuuAugucagc....................   1       1    NN8
    ......................................................................gcucacuccuAuuucugucagc....................   2       1    NN8
    ......................................................................gcucacuccucuuucAgucagc....................   1       1    NN8
    ......................................................................gcCcacuccucuuucugucagc....................   1       1    NN8
    ......................................................................gcucacuccucuuucugucagG....................   1       1    NN8
    ......................................................................gcucacuccuGuuucugucagc....................   1       1    NN8
    ......................................................................gcucaUuccucuuucugucagc....................   2       1    NN8
    ......................................................................gcucacuccucuAucugucagc....................   1       1    NN8
```

Mature **Star**

```
gagagauugacagaagagagugagcacauggcgugaugccggcauaacauguaugccgucuucgccgcgugcucacuccucuuucugucagccucuuucucucaggaguuua
......................................................................gcucacuccucuuucuguAagc....................   1     1   NN8
......................................................................gcucacuccucuuucugucagc....................   837   0   NN8
......................................................................gcucUcuccucuuucugucagc....................   2     1   NN8
......................................................................gcucacuccucuuucugucagGc...................   1     1   NN8
......................................................................gcucacuccucuuucugAcagcc...................   2     1   NN8
......................................................................gcucacuccucuuucugucUgcc...................   1     1   NN8
......................................................................gAucacuccucuuucugucagcc...................   1     1   NN8
......................................................................gcucacuccucGuucugucagcc...................   1     1   NN8
......................................................................Ucucacuccucuuucugucagcc...................   1     1   NN8
......................................................................gcucacuccucuuucGgucagcc...................   1     1   NN8
......................................................................Acucacuccucuuucugucagcc...................   1     1   NN8
......................................................................gcucGcuccucuuucugucagcc...................   3     1   NN8
......................................................................gcucacuccucuuuGugucagcc...................   1     1   NN8
......................................................................gcucacuccucuuucugucagcU...................   2     1   NN8
......................................................................gcucacuccucuuucuguAagcc...................   2     1   NN8
......................................................................gcucacuccuAuuucugucagcc...................   1     1   NN8
......................................................................gcucacuccucuuucuguGagcc...................   2     1   NN8
......................................................................gcucacuccucuuucugucagcA...................   3     1   NN8
......................................................................gcucacuccGcuuucugucagcc...................   1     1   NN8
......................................................................gcucacuccucuuucugCcagcc...................   1     1   NN8
......................................................................gcucacuccuGuuucugucagcc...................   1     1   NN8
......................................................................gcucacucUucuuucugucagcc...................   1     1   NN8
......................................................................gcucacuccucuuucugGcagcc...................   2     1   NN8
......................................................................gcucacuccucuuucAgucagcc...................   1     1   NN8
......................................................................gcucacuccucuuucugucagUc...................   1     1   NN8
......................................................................gcucacuccucuuucugucagcc...................   589   0   NN8
......................................................................gcucacuccucuuucuguUagcc...................   1     1   NN8
......................................................................gcucacuccucuuuUugucagccu..................   1     1   NN8
......................................................................gcucaGuccucuuucugucagccu..................   1     1   NN8
......................................................................gcucacuccucuuucugucagccA..................   8     1   NN8
......................................................................gGucacuccucuuucugucagccu..................   1     1   NN8
......................................................................gcucacuccucuuucAgucagccu..................   1     1   NN8
......................................................................gcucacuccucuuucugucagccu..................   89    0   NN8
......................................................................gcucacuccucuuucugucagccC..................   1     1   NN8
......................................................................gcucacuccucuuucugucagccuc.................   2     0   NN8
.........................................................................cacuccucuuucugucagc....................   1     0   NN8

....gauugacagaagagagugagcac.....................................................................................   2     0   FF1
.....auugacagaagagagugagcac.....................................................................................   1     0   FF1
......uugacagaagagagugag........................................................................................   2     0   FF1
......uugacagaagagagugagc.......................................................................................   2     0   FF1
......uugacagaagagagugagca......................................................................................   5     0   FF1
......uugacagaagagGgugagca......................................................................................   1     1   FF1
......uugacagaagagagugagAac.....................................................................................   2     1   FF1
......uugGcagaagagagugagcac.....................................................................................   1     1   FF1
......uugacagaagagagGgagcac.....................................................................................   12    1   FF1
......uugacagaagagagugaCcac.....................................................................................   2     1   FF1
......uugacagaagagagugaAcac.....................................................................................   4     1   FF1
......Augacagaagagagugagcac.....................................................................................   2     1   FF1
......uugacaAaagagagugagcac.....................................................................................   2     1   FF1
......uugacagaagagagAgagcac.....................................................................................   1     1   FF1
......uugaGagaagagagugagcac.....................................................................................   1     1   FF1
......uugacagaagagagugagcac.....................................................................................   1307  0   FF1
......uugacagaagagaguUagcac.....................................................................................   1     1   FF1
......uuAacagaagagagugagcac.....................................................................................   1     1   FF1
......uugacagaagagagCgagcac.....................................................................................   3     1   FF1
......uugacagaagagagugagcaU.....................................................................................   3     1   FF1
......uugacagaagagaguAagcac.....................................................................................   3     1   FF1
......uugacagaagagGgugagcac.....................................................................................   2     1   FF1
......uugaUagaagagagugagcac.....................................................................................   3     1   FF1
......uugacagaagagagugaUcac.....................................................................................   1     1   FF1
......uugacagaagagUgugagcac.....................................................................................   4     1   FF1
......uugCcagaagagagugagcac.....................................................................................   1     1   FF1
......uugacagaagagagugagcaA.....................................................................................   1     1   FF1
......uugacUgaagagagugagcac.....................................................................................   1     1   FF1
......uugacagaagagagugagGac.....................................................................................   1     1   FF1
......uuCacagaagagagugagcac.....................................................................................   1     1   FF1
......uugacagaagagagugGgcac.....................................................................................   1     1   FF1
......uugacagaagagCgugagcac.....................................................................................   3     1   FF1
......uugacagaCgagagugagcac.....................................................................................   1     1   FF1
```

Mature **Star**

```
gagagauugacagaagagagugagcacauggcgugaugccggcauaacauguaugccgucuucgccgcgugcucacuccucuuucugucagccucuuucucucaggaguuua
......uugacagaagagagugagcacU....................................................................................    20     1    FF1
......uugacagaagagagugagcaca....................................................................................    3      0    FF1
.......ugacagaagagagugagc.......................................................................................    3      0    FF1
.......ugCcagaagagagugagca......................................................................................    1      1    FF1
.......ugacagaagagagugagca......................................................................................    22     0    FF1
.......Ggacagaagagagugagca......................................................................................    1      1    FF1
.......ugacagaagagaCugagca......................................................................................    1      1    FF1
.......ugacagaagagCgugagcac.....................................................................................    1      1    FF1
.......ugacagaagagagugagcCc.....................................................................................    2      1    FF1
.......ugacagaagagagugaUcac.....................................................................................    1      1    FF1
.......ugacagaaAagagugagcac.....................................................................................    3      1    FF1
.......uAacagaagagagugagcac.....................................................................................    1      1    FF1
.......ugacagaagaAagugagcac.....................................................................................    1      1    FF1
.......ugacagCagagagugagcac.....................................................................................    1      1    FF1
.......ugacagaagagGgugagcac.....................................................................................    2      1    FF1
.......ugacagaaCagagugagcac.....................................................................................    1      1    FF1
.......ugacagaagagaCugagcac.....................................................................................    2      1    FF1
.......ugacagaagagaAugagcac.....................................................................................    3      1    FF1
.......ugacagaGgagagugagcac.....................................................................................    1      1    FF1
.......ugaUagaagagagugagcac.....................................................................................    1      1    FF1
.......Ggacagaagagagugagcac.....................................................................................    3      1    FF1
.......Agacagaagagagugagcac.....................................................................................    1      1    FF1
.......ugacagaagagagugagcac.....................................................................................    2949   0    FF1
.......ugacagaagagagugagcGc.....................................................................................    1      1    FF1
.......ugaGagaagagagugagcac.....................................................................................    1      1    FF1
.......ugacagaagagagugagcaU.....................................................................................    11     1    FF1
.......ugacagaagagagugagcaA.....................................................................................    2      1    FF1
.......ugGcagaagagagugagcac.....................................................................................    2      1    FF1
.......ugacagaagagagugagGac.....................................................................................    6      1    FF1
.......ugacagaagagagAgagcac.....................................................................................    2      1    FF1
.......ugacagaagagagGgagcac.....................................................................................    8      1    FF1
.......ugacagaagagagugagAac.....................................................................................    5      1    FF1
.......ugacagaagagaUugagcac.....................................................................................    7      1    FF1
.......ugacagaagagagugCgcac.....................................................................................    2      1    FF1
.......ugaAagaagagagugagcac.....................................................................................    4      1    FF1
.......ugacagGagagagugagcac.....................................................................................    2      1    FF1
.......ugacagaagagagugaCcac.....................................................................................    1      1    FF1
.......ugacagaagagagugaAcac.....................................................................................    1      1    FF1
.......uUacagaagagagugagcac.....................................................................................    2      1    FF1
.......ugacagaagagagugGgcac.....................................................................................    11     1    FF1
.......ugacagaagagagugagcacU....................................................................................    151    1    FF1
.......ugacagaagagagugagcaca....................................................................................    21     0    FF1
.......ugacagaagagagugagcacCu...................................................................................    1      1    FF1
.........cagaagagagugagcaca.....................................................................................    1      0    FF1
.....................................................................ugcucacuccucuuucuguca......................    1      0    FF1
.....................................................................ugcucacuccucuuucugucag.....................    5      0    FF1
.....................................................................ugcucacuccucuuucuguAagc....................    1      1    FF1
.....................................................................ugcucacuccucuuucugucagc....................    12     0    FF1
......................................................................gcucacuccucuuucugu........................    1      0    FF1
......................................................................gcucacuccucuuucuguc.......................    2      0    FF1
......................................................................gcucacuccucuuucugucag.....................    6      0    FF1
......................................................................gcucacuccucuCucugucagc....................    2      1    FF1
......................................................................gcucacuccucAuucugucagc....................    1      1    FF1
......................................................................gcucGcuccucuuucugucagc....................    1      1    FF1
......................................................................gcucacuccucGuucugucagc....................    1      1    FF1
......................................................................gcucacuccucuuucuCucagc....................    1      1    FF1
......................................................................gcucacuccucuAucugucagc....................    1      1    FF1
......................................................................gcucacuccucuuucugucagc....................    574    0    FF1
......................................................................gcucacuccucuuucugucagU....................    1      1    FF1
......................................................................gcucacucAucuuucugucagc....................    2      1    FF1
......................................................................gUucacuccucuuucugucagc....................    1      1    FF1
......................................................................gcucacuccucuuucugGcagc....................    3      1    FF1
......................................................................gcucacuAcucuuucugucagc....................    1      1    FF1
......................................................................gGucacuccucuuucugucagc....................    1      1    FF1
......................................................................gcuAacuccucuuucugucagc....................    1      1    FF1
......................................................................gcucacAccucuuucugucagc....................    1      1    FF1
......................................................................gcucacuccucuuucuguUagc....................    1      1    FF1
......................................................................gcucacucGucuuucugucagc....................    1      1    FF1
......................................................................gcucacuccucuuucGgucagc....................    2      1    FF1
......................................................................gcucacuccucuuucAgucagc....................    1      1    FF1
```

Mature **Star**

```
gagagauugacagaagagagugagcacauggcgugaugccggcauaacauguaugccgucuucgccgcgugcucacuccucuuucugucagccucuuucucucaggaguuua
......................................................................gcucacuccucuGucugucagc....................  1    1  FF1
......................................................................gcucacGccucuuucugucagc....................  2    1  FF1
......................................................................gcucacuccucuuucCgucagc....................  1    1  FF1
......................................................................gcucacuccucuuucugCcagc....................  1    1  FF1
......................................................................gcucacuccucuuucuguGagc....................  1    1  FF1
......................................................................gcucacuccucuuucugucagcc...................  441  0  FF1
......................................................................gcucUcuccucuuucugucagcc...................  1    1  FF1
......................................................................gcucacuccucuuucAgucagcc...................  1    1  FF1
......................................................................gcucacuccucuuucugGcagcc...................  1    1  FF1
......................................................................gcucacucGucuuucugucagcc...................  1    1  FF1
......................................................................gGucacuccucuuucugucagcc...................  1    1  FF1
......................................................................gcuGacuccucuuucugucagcc...................  1    1  FF1
......................................................................gcucacAccucuuucugucagcc...................  1    1  FF1
......................................................................gcucacuccucuuucugCcagcc...................  3    1  FF1
......................................................................gcucacucAucuuucugucagcc...................  1    1  FF1
......................................................................gcucacuccucuCucugucagcc...................  2    1  FF1
......................................................................gcucUcuccucuuucugucagccu..................  1    1  FF1
......................................................................gcucacuccucuuucugucagccC..................  5    1  FF1
......................................................................gcucacuccucuuucugucagccG..................  1    1  FF1
......................................................................gcucacuccucuuucugucagccA..................  1    1  FF1
......................................................................gcucacuccucuuucugucagccu..................  100  0  FF1
......................................................................gcucacuccucuuucugGcagccu..................  1    1  FF1
......................................................................gcucacuccucuuucuCucagccu..................  1    1  FF1
......................................................................gcucacuccucGuucugucagccu..................  1    1  FF1
......................................................................gcucacuccucuuucugucagccuc.................  1    0  FF1
........................................................................ucacuccucuuucugucagccu..................  1    0  FF1
........................................................................uAacuccucuuucugucagccu..................  1    1  FF1
```
