# Supplementary material for: Identification of Novel miRNAs and miRNA Expression Profiling in Wheat Hybrid Necrosis
Source: PLoS One. 2015 Feb 23;10(2):e0117507. doi: 10.1371/journal.pone.0117507 (PMC4338152; doi:10.1371/journal.pone.0117507)
Supplement: S2 Fig — Red colored letter: mature miRNA sequence; yellow colored letter: loop sequence; blue colored letter: miRNA* sequence. (ZIP) [file pone.0117507.s002.zip › Figures s1/contig1095979_10124.pdf]

Provisional ID	:	contig1095979_10124
Score total	:	2
Score for star read(s)	:	-1.3
Score for read counts	:	0
Score for mfe	:	2.3
Score for randfold	:	1.6
Score for cons. seed	:	-0.6
Total read count	:	14
Mature read count	:	5
Loop read count	:	9
Star read count	:	0

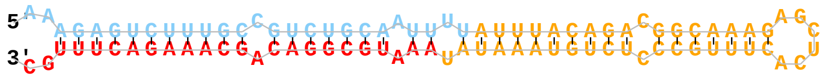

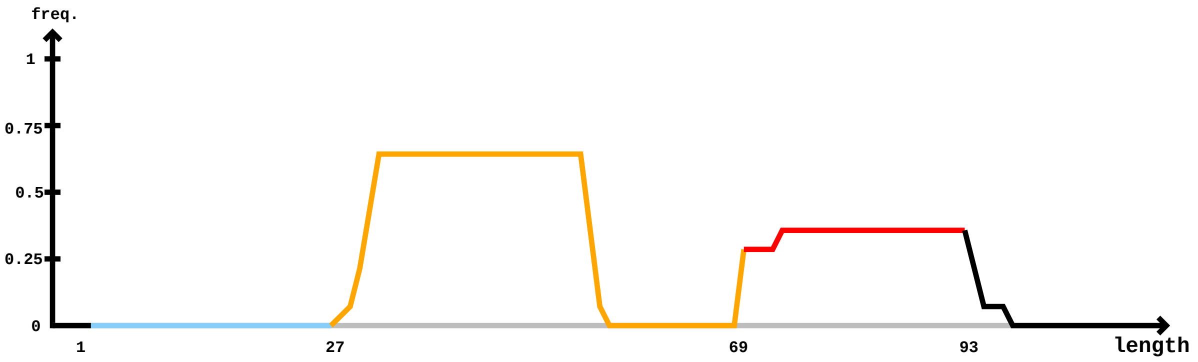

Star Mature

```
5'- gcaaagagucuuugccgucugcaauuuuauuuacagacggcaaagagcucacuuugcccucuguaaauauaaaugcggacagcaaagacuuugccaucuguguuuuuuguuaca   -3'    exp
    (((..((((((((((.(((((((.((.((((((((((.(((((((......))))))).)))))))))).)).))))))).))))))))))))).....(((........))).   reads      mm         sample
    ..............................uuacagacgAcaaagagcucacuu............................................................   2          1          NN8
    ................................acagacgAcaaagagcucacuuu...........................................................   2          1          NN8

    .............................uuuacagacgAcaaagagcucacuuu...........................................................   1          1          FF1
    ...............................uacagacggcaaagaAcucacuu............................................................   1          1          FF1
    ...............................uacaAacggcaaagagcucacuu............................................................   1          1          FF1
    ...............................uaAagacggcaaagagcucacuuu...........................................................   1          1          FF1
    ................................acagacggcaaagagcucacuuug..........................................................   1          0          FF1
    ......................................................................aaaugcggacagcaaagacuuugc....................   4          0          FF1
    ..........................................................................gcggacagcaaagacuuugccauc................   1          0          FF1
```
