# Supplementary material for: Identification of Novel miRNAs and miRNA Expression Profiling in Wheat Hybrid Necrosis
Source: PLoS One. 2015 Feb 23;10(2):e0117507. doi: 10.1371/journal.pone.0117507 (PMC4338152; doi:10.1371/journal.pone.0117507)
Supplement: S2 Fig — Red colored letter: mature miRNA sequence; yellow colored letter: loop sequence; blue colored letter: miRNA* sequence. (ZIP) [file pone.0117507.s002.zip › Figures s1/contig1109357_10188.pdf]

Provisional ID	:	contig1109357_10188
Score total	:	2030.1
Score for star read(s)	:	3.9
Score for read counts	:	2019
Score for mfe	:	2.5
Score for randfold	:	1.6
Score for cons. seed	:	3
Total read count	:	3972
Mature read count	:	3406
Loop read count	:	0
Star read count	:	566

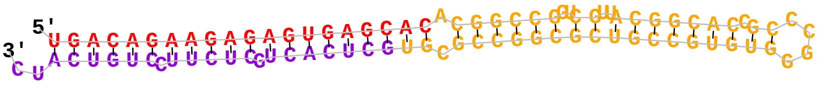

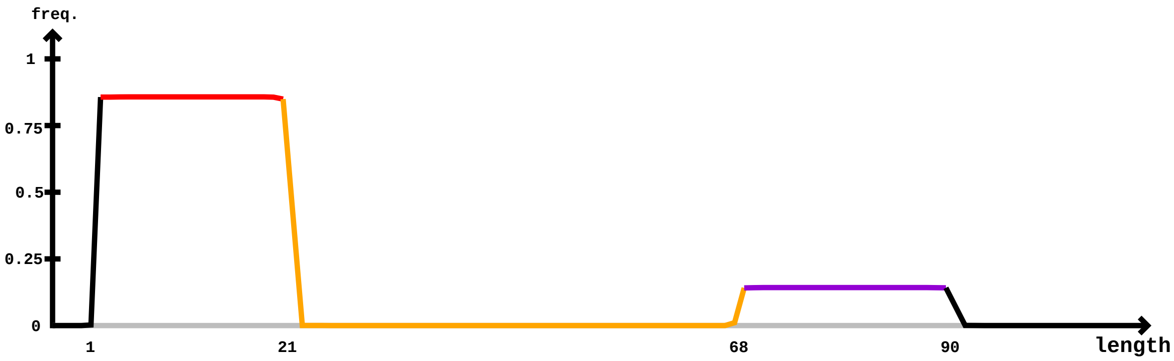

Mature **Star**

```
5'- cggugacagaagagagugagcacacggccggucguuacggcaccgcccgggugugccgucgcggccgcgugcucacugcucuuccugucauccacucucccgcuucccuucc  -3'  obs
    cggugacagaagagagugagcacacggccggucguuacggcaccgcccgggugugccgucgcggccgcgugcucacugcucuuccugucauccacucucccgcuucccuucc       exp
    .((((((((((((((((((((((.((((((..((..(((((((.((....))))))))))))))))).)))))))))).))))).))))))))....................   reads   mm   sample
    ...ugacagaagagagugagc...........................................................................................   1       0    NN8
    ...ugacagaagagagugagca..........................................................................................   2       0    NN8
    ...ugacagaagagagugagcac.........................................................................................   135     0    NN8
    ...ugacagaagagagugUgcac.........................................................................................   1       1    NN8
    ...ugacagaagagagugagcaU.........................................................................................   1       1    NN8
    ...Agacagaagagagugagcac.........................................................................................   1       1    NN8
    ...ugacagaagagGgugagcac.........................................................................................   1       1    NN8
    ...ugacagaagagagugagcacU........................................................................................   11      1    NN8
    .....acagaagagagugagcac.........................................................................................   1       0    NN8
    ......................................................................gcAcacugcucuuccuguca......................   1       1    NN8
    ......................................................................gcucacugcucuuccuguca......................   1       0    NN8
    ......................................................................gcucacuAcucuuccugucauc....................   1       1    NN8
    ......................................................................gcucacugcucuuccugucUuc....................   1       1    NN8
    ......................................................................gcucaAugcucuuccugucauc....................   1       1    NN8
    ......................................................................gcucacugcucuuccuguAauc....................   1       1    NN8
    ......................................................................gcucacugcucuuccugAcauc....................   1       1    NN8
    ......................................................................gcucacugcGcuuccugucauc....................   1       1    NN8
    ......................................................................gcucacugcucuuccugucauc....................   263     0    NN8
    ......................................................................gcucacugcucuuccuguUauc....................   1       1    NN8
    ......................................................................gcucacugcucuuccuguGauc....................   1       1    NN8
    ......................................................................gcucacugcucuuccuAucauc....................   1       1    NN8
    ......................................................................gcucacugcucuuccugucaucc...................   38      0    NN8
    ......................................................................gcGcacugcucuuccugucaucc...................   1       1    NN8
    ......................................................................gcucacugcucuuccugucauccU..................   1       1    NN8
    ........................................................................cucacugcucuuccugucauc....................   1       0    NN8

    ..gugacagaagagagugagca..........................................................................................   2       0    FF1
    ..Augacagaagagagugagcac.........................................................................................   2       1    FF1
    ..gugacagaagagagugagcac.........................................................................................   5       0    FF1
    ...ugacagaagagagugagc...........................................................................................   3       0    FF1
    ...Ggacagaagagagugagca..........................................................................................   1       1    FF1
    ...ugacagaagagagugagca..........................................................................................   22      0    FF1
    ...ugacagaagagaCugagca..........................................................................................   1       1    FF1
```

Mature **Star**

cggugacagaagagagugagcacacggccggucguuacggcaccgcccgggugugccgucgcggccgcgugcucacugcucuuccugucauccacucucccgcuucccuucc			
...ugCcagaagagagugagca..	1	1	FF1
...ugacagaagagaCugagcac...	2	1	FF1
...ugacagaagagagugagGac...	6	1	FF1
...ugacagaaAagagugagcac...	3	1	FF1
...ugacagaagagagugagAac...	5	1	FF1
...ugacagaagagagugaCcac...	1	1	FF1
...ugacagaagagaUugagcac...	7	1	FF1
...ugacagaagagagugagcaA...	2	1	FF1
...ugacagaagagagGgagcac...	8	1	FF1
...ugacagaGgagagugagcac...	1	1	FF1
...ugacagaagagagugaAcac...	1	1	FF1
...ugacagaagagagAgagcac...	2	1	FF1
...Agacagaagagagugagcac...	1	1	FF1
...ugacagGagagagugagcac...	2	1	FF1
...ugacagCagagagugagcac...	1	1	FF1
...ugacagaagagagugaUcac...	1	1	FF1
...ugacagaagagagugagcCc...	2	1	FF1
...ugacagaagaAagugagcac...	1	1	FF1
...ugGcagaagagagugagcac...	2	1	FF1
...ugaAagaagagagugagcac...	4	1	FF1
...ugacagaagagCgugagcac...	1	1	FF1
...ugacagaagagagugagcGc...	1	1	FF1
...Ggacagaagagagugagcac...	3	1	FF1
...uUacagaagagagugagcac...	2	1	FF1
...ugacagaagagagugagcac...	2949	0	FF1
...ugaUagaagagagugagcac...	1	1	FF1
...ugacagaagagagugGgcac...	11	1	FF1
...ugacagaaCagagugagcac...	1	1	FF1
...ugacagaagagagugagcaU...	11	1	FF1
...ugacagaagagagugCgcac...	2	1	FF1
...uAacagaagagagugagcac...	1	1	FF1
...ugaGagaagagagugagcac...	1	1	FF1
...ugacagaagagGgugagcac...	2	1	FF1
...ugacagaagagaAugagcac...	3	1	FF1
...ugacagaagagagugagcacU..	151	1	FF1
...ugacagaagagagugagcaca..	21	0	FF1
.....acagaagagagugagcacacgU...	1	1	FF1
......cagaagagagugagcaca...	1	0	FF1
....ugcucacugcucuuccugucauc....................	42	0	FF1
....ugcucacugcucuuccugucaucG...................	1	1	FF1
....gcucacugcucuuccugucau.....................	2	0	FF1
....gcucacugcucuuccGgucauc....................	1	1	FF1
....gcucacuAcucuuccugucauc....................	1	1	FF1
....gcucacugcucuuccugucauc....................	170	0	FF1
....gcucacugcucAuccugucaucc...................	1	1	FF1
....gcucacugcucuuccugCcaucc...................	1	1	FF1
....gcucacugcucuuccGgucaucc...................	1	1	FF1
....gcucacugcucuuccugucaucc...................	27	0	FF1
....cucacugcucuuccugucauc....................	1	0	FF1
....cucacugcucuuccugucaucc...................	1	0	FF1
....ucacugcucuuccugucaucc...................	1	0	FF1
....ucacugcucuuccugucauccac.................	1	0	FF1
