# Supplementary material for: Identification of Novel miRNAs and miRNA Expression Profiling in Wheat Hybrid Necrosis
Source: PLoS One. 2015 Feb 23;10(2):e0117507. doi: 10.1371/journal.pone.0117507 (PMC4338152; doi:10.1371/journal.pone.0117507)
Supplement: S2 Fig — Red colored letter: mature miRNA sequence; yellow colored letter: loop sequence; blue colored letter: miRNA* sequence. (ZIP) [file pone.0117507.s002.zip › Figures s1/contig1109643_10191.pdf]

| | | |
|---|---|---|
| Provisional ID | : | contig1109643_10191 |
| Score total | : | 9.9 |
| Score for star read(s) | : | 3.9 |
| Score for read counts | : | 3.2 |
| Score for mfe | : | 1.8 |
| Score for randfold | : | 1.6 |
| Score for cons. seed | : | -0.6 |
| Total read count | : | 18 |
| Mature read count | : | 15 |
| Loop read count | : | 0 |
| Star read count | : | 3 |

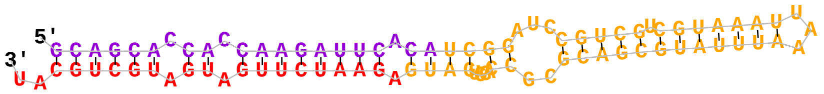

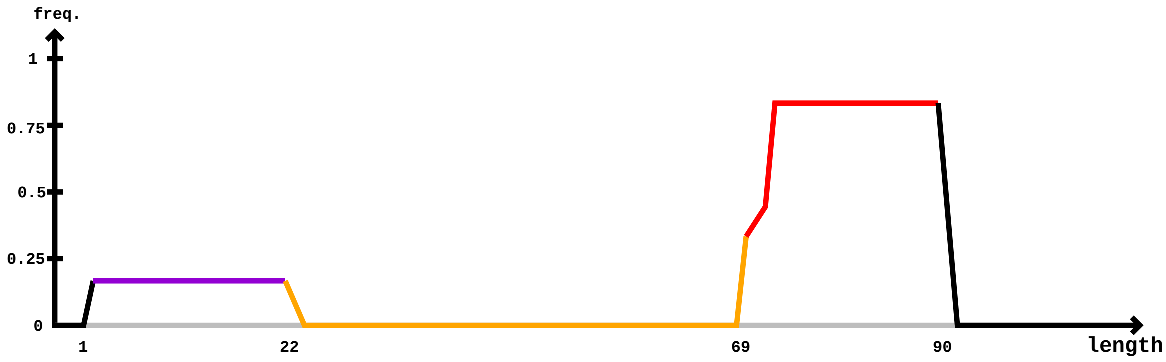


```
    Star                                                                 Mature
5'- gugcagcaccaccaagauucacaucggauccgucgucguaaauuaaauuuaugcgacgcgccaggugaugagaaucuugaugaugcugcaucagcaggcacucgacucgac   -3'    obs
    gugcagcaccaccaagauucacaucggauccgucgucguaaauuaaauuuaugcgacgcgccaggugaugagaaucuugaugaugcugcaucagcaggcacucgacucgac          exp
    ((((((((.((.((((((((.((((((...(((((.(((((((...))))))))))))..))....)))).)))))))).)).))))))))....................    reads    mm    sample
    ..gcagcaccaccaagauucaca........................................................................................    3        0     NN8
    .......................................................................gaaucuugaugaugcugcGuc...................    1        1     NN8
    ........................................................................aaucuugaugaugcugcau....................    1        0     NN8
    .........................................................................aucuugaugaugcugcGu....................    1        1     NN8
    .........................................................................aucuugaugaugcugcau....................    5        0     NN8

    ......................................................................agaaucuugaugaugcugcau....................    6        0     FF1
    .........................................................................aucuugaugaugcugcau....................    1        0     FF1
```
