# Supplementary material for: Identification of Novel miRNAs and miRNA Expression Profiling in Wheat Hybrid Necrosis
Source: PLoS One. 2015 Feb 23;10(2):e0117507. doi: 10.1371/journal.pone.0117507 (PMC4338152; doi:10.1371/journal.pone.0117507)
Supplement: S2 Fig — Red colored letter: mature miRNA sequence; yellow colored letter: loop sequence; blue colored letter: miRNA* sequence. (ZIP) [file pone.0117507.s002.zip › Figures s1/contig112667_2846.pdf]

Provisional ID	: contig112667_2846	
Score total	:	5.9
Score for star read(s)	:	-1.3
Score for read counts	:	0
Score for mfe	:	2.6
Score for randfold	:	1.6
Score for cons. seed	:	3
Total read count	:	16
Mature read count	:	16
Loop read count	:	0
Star read count	:	0

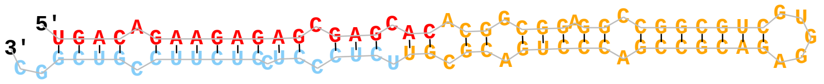

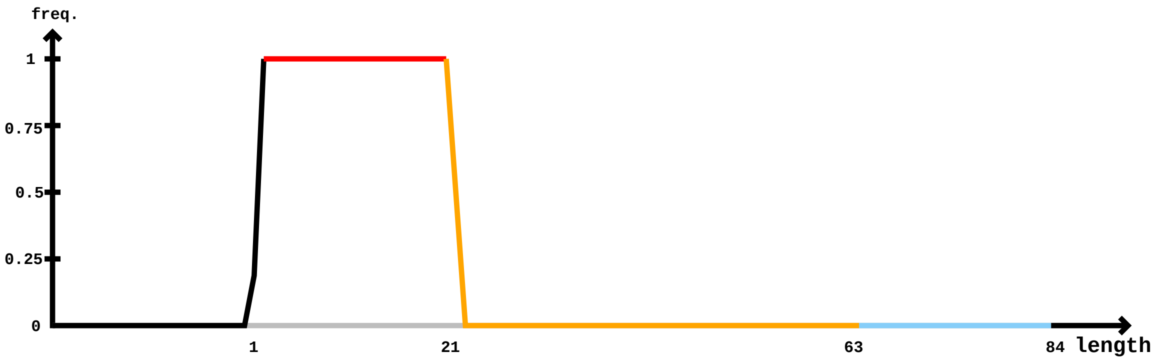

Mature   Star

```
5'- ccgccgugcccgcgggaggcugacagaagagagcgagcacacggcggaggccggcgucguggagacgccgacccugacgcguucucccuccucuuccgucggccuccggc  -3'   exp
    ...........((.((((((((((.((((((((.(((.((.((.(((.((.(((((((.....))))))).))))).)).)).))).)).)))))).)))))))))).))   reads    mm    sample
    ...................Uugacagaagagagcgagcac......................................................................   3        1     FF1
    ....................ugacagaagagagGgagcac......................................................................   8        1     FF1
    ....................ugacagaagagagAgagcac......................................................................   2        1     FF1
    ....................ugacagaagagagcgagcac......................................................................   3        0     FF1
```
