# Supplementary material for: Identification of Novel miRNAs and miRNA Expression Profiling in Wheat Hybrid Necrosis
Source: PLoS One. 2015 Feb 23;10(2):e0117507. doi: 10.1371/journal.pone.0117507 (PMC4338152; doi:10.1371/journal.pone.0117507)
Supplement: S2 Fig — Red colored letter: mature miRNA sequence; yellow colored letter: loop sequence; blue colored letter: miRNA* sequence. (ZIP) [file pone.0117507.s002.zip › Figures s1/contig1236006_10709.pdf]

| | | |
|---|---|---|
| Provisional ID | : | contig1236006_10709 |
| Score total | : | 2.4 |
| Score for star read(s) | : | -1.3 |
| Score for read counts | : | 0 |
| Score for mfe | : | 2.7 |
| Score for randfold | : | 1.6 |
| Score for cons. seed | : | -0.6 |
| Total read count | : | 41 |
| Mature read count | : | 32 |
| Loop read count | : | 0 |
| Star read count | : | 9 |

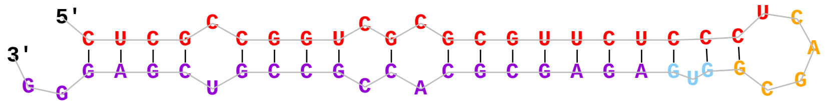


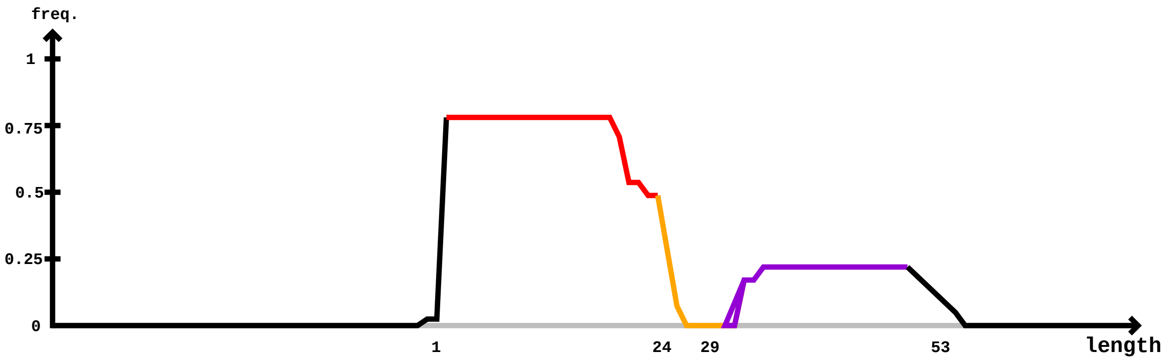


Mature Star

```
5'- guaagaggagcgcggccagauugcgccguggccauccuccucgccggucgcgcguucucccucagcggugagagcgcaccgccgucgagggacuuggccgcggugcuggcc  -3'   obs
    guaagaggagcgcggccagauugcgccguggccauccuccucgccggucgcgcguucucccucagcggugagagcgcaccgccgucgagggacuuggccgcggugcuggcc         exp
    ............(((((....((((((((((((...((((((.((((.(.((((((((((.....)).)))))))).).)))).))))))...))))))))))))))))    reads   mm   sample
    .......................................Uucgccggucgcgcguucu.....................................................    1       1    NN8
    .......................................cucgccggucgcgcguCcu.....................................................    1       1    NN8
    .......................................cucgccggucgcgcguucucc...................................................    2       0    NN8
    .......................................cucgccggucgcgcguucucccu.................................................    3       0    NN8
    .......................................cucgccggucgcgcguCcucccu.................................................    9       1    NN8
    .......................................cucgccggucgcgcguucucccuca...............................................    2       0    NN8
    ......................................................................agagcgcaccgccgucgaggg....................    1       0    NN8

    .....................................uccucgccggucgcgcguucu.....................................................    1       0    FF1
    .......................................cucgccggucgcgcguCc......................................................    3       1    FF1
    .......................................Uucgccggucgcgcguucu.....................................................    4       1    FF1
    .......................................cucgccggucgcgcguCcucccu.................................................    2       1    FF1
    .......................................cucgccggucgcgcguCcucccuc................................................    2       1    FF1
    .......................................cucgccggucgcgcguucucccuc................................................    1       0    FF1
    .......................................cucgccggucgcgcguucucccuca...............................................    1       0    FF1
    ......................................................................agagcgcaccgccgucgaggg....................    6       0    FF1
    ........................................................................agcgcaccgccgucgagggac..................    2       0    FF1
```
