# Supplementary material for: Identification of Novel miRNAs and miRNA Expression Profiling in Wheat Hybrid Necrosis
Source: PLoS One. 2015 Feb 23;10(2):e0117507. doi: 10.1371/journal.pone.0117507 (PMC4338152; doi:10.1371/journal.pone.0117507)
Supplement: S2 Fig — Red colored letter: mature miRNA sequence; yellow colored letter: loop sequence; blue colored letter: miRNA* sequence. (ZIP) [file pone.0117507.s002.zip › Figures s1/contig1282657_10869.pdf]

Provisional ID	:	contig1282657_10869
Score total	:	2.2
Score for star read(s)	:	-1.3
Score for read counts	:	0
Score for mfe	:	2.5
Score for randfold	:	1.6
Score for cons. seed	:	-0.6
Total read count	:	26
Mature read count	:	26
Loop read count	:	0
Star read count	:	0

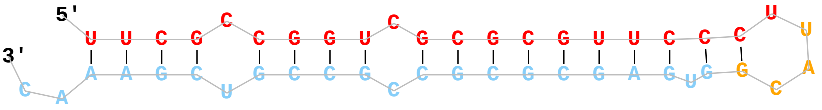

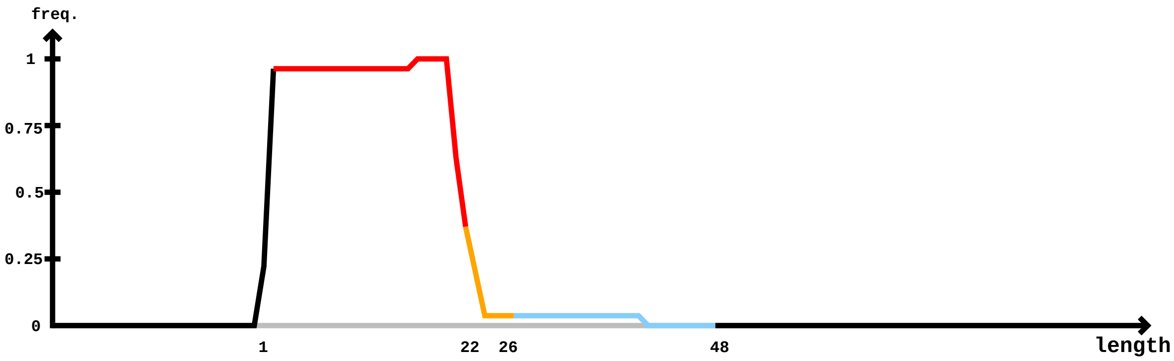

Mature **Star**

```
5'- gguggcgucguugccauuccuuucgccggucgcgcguucccuuacggugagcgcgccgccgucgaaacguuuggccgcggugcccuuugccccucucggggugguucuucgu   -3'    exp
    ((.(((..(((.((((..(.(((((.((((.((((((((((....)).)))))))).)))).))))).)..)))).)))..)))))(..((((....))))..)........    reads     mm        sample
    .....................uucgccggucgcgcguucU........................................................................    1         1         NN8

    ....................uuucgccggucgcgcguucU........................................................................    1         1         FF1
    ....................uuucgccggucgcgcguuccc.......................................................................    1         0         FF1
    ....................Cuucgccggucgcgcguuccc.......................................................................    1         1         FF1
    ....................uuucgccggucgcgcguucccu......................................................................    3         0         FF1
    .....................uucgcUggucgcgcguucc........................................................................    1         1         FF1
    .....................uucgccggucgcgcguucU........................................................................    4         1         FF1
    .....................uucgccggucgcgcguucc........................................................................    3         0         FF1
    .....................uucgccggucgcgcguuccc.......................................................................    2         0         FF1
    .....................uucgccggucgcgcguuccU.......................................................................    3         1         FF1
    .....................uucgccggucgcgcguucccu......................................................................    5         0         FF1
    .....................uucgccggucgcgcguucccuu.....................................................................    1         0         FF1
    ....................................uucccuuacggugagcgcgccgcc....................................................    1         0         FF1
```
