# Supplementary material for: Identification of Novel miRNAs and miRNA Expression Profiling in Wheat Hybrid Necrosis
Source: PLoS One. 2015 Feb 23;10(2):e0117507. doi: 10.1371/journal.pone.0117507 (PMC4338152; doi:10.1371/journal.pone.0117507)
Supplement: S2 Fig — Red colored letter: mature miRNA sequence; yellow colored letter: loop sequence; blue colored letter: miRNA* sequence. (ZIP) [file pone.0117507.s002.zip › Figures s1/contig1299632_10941.pdf]

Provisional ID	:	contig1299632_10941
Score total	:	2030.1
Score for star read(s)	:	3.9
Score for read counts	:	2019
Score for mfe	:	2.5
Score for randfold	:	1.6
Score for cons. seed	:	3
Total read count	:	3972
Mature read count	:	3406
Loop read count	:	0
Star read count	:	566

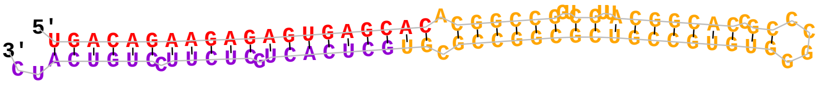

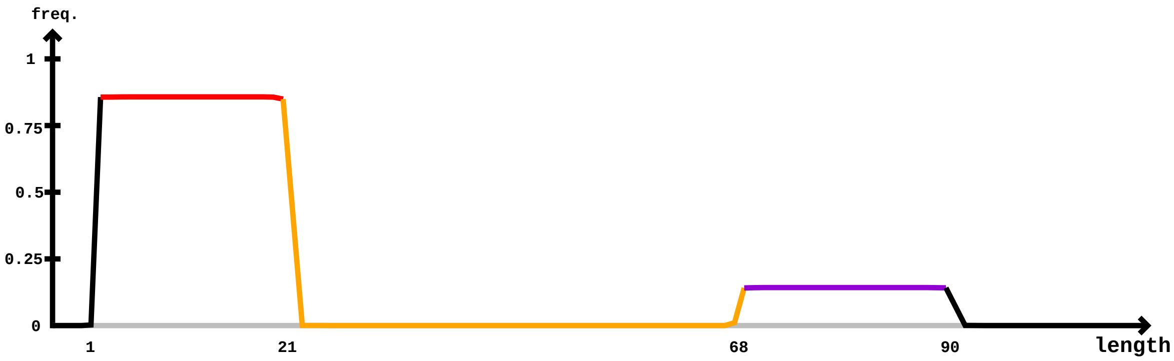

Mature **Star**

```
5'- cggugacagaagagagugagcacacggccggucguuacggcaccgcccgggugugccgucgcggccgcgugcucacugcucuuccugucauccacucucccgcuucccuucc   -3'   obs
    cggugacagaagagagugagcacacggccggucguuacggcaccgcccgggugugccgucgcggccgcgugcucacugcucuuccugucauccacucucccgcuucccuucc         exp
    .((((((((((((((((((((((.((((((..((..(((((((.((....))))))))))))))))).))))))))).))))).))))))))....................   reads   mm   sample
    ...ugacagaagagagugagc...........................................................................................   1       0    NN8
    ...ugacagaagagagugagca..........................................................................................   2       0    NN8
    ...ugacagaagagagugagcac.........................................................................................   135     0    NN8
    ...ugacagaagagagugUgcac.........................................................................................   1       1    NN8
    ...ugacagaagagagugagcaU.........................................................................................   1       1    NN8
    ...Agacagaagagagugagcac.........................................................................................   1       1    NN8
    ...ugacagaagagGgugagcac.........................................................................................   1       1    NN8
    ...ugacagaagagagugagcacU........................................................................................   11      1    NN8
    .....acagaagagagugagcac.........................................................................................   1       0    NN8
    ......................................................................gcAcacugcucuuccuguca......................   1       1    NN8
    ......................................................................gcucacugcucuuccuguca......................   1       0    NN8
    ......................................................................gcucacuAcucuuccugucauc....................   1       1    NN8
    ......................................................................gcucacugcucuuccugucUuc....................   1       1    NN8
    ......................................................................gcucaAugcucuuccugucauc....................   1       1    NN8
    ......................................................................gcucacugcucuuccuguAauc....................   1       1    NN8
    ......................................................................gcucacugcucuuccugAcauc....................   1       1    NN8
    ......................................................................gcucacugcGcuuccugucauc....................   1       1    NN8
    ......................................................................gcucacugcucuuccugucauc....................   263     0    NN8
    ......................................................................gcucacugcucuuccuguUauc....................   1       1    NN8
    ......................................................................gcucacugcucuuccuguGauc....................   1       1    NN8
    ......................................................................gcucacugcucuuccuAucauc....................   1       1    NN8
    ......................................................................gcucacugcucuuccugucaucc...................   38      0    NN8
    ......................................................................gcGcacugcucuuccugucaucc...................   1       1    NN8
    ......................................................................gcucacugcucuuccugucauccU..................   1       1    NN8
    .......................................................................cucacugcucuuccugucauc....................   1       0    NN8

    ..gugacagaagagagugagca..........................................................................................   2       0    FF1
    ..Augacagaagagagugagcac.........................................................................................   2       1    FF1
    ..gugacagaagagagugagcac.........................................................................................   5       0    FF1
    ...ugacagaagagagugagc...........................................................................................   3       0    FF1
    ...Ggacagaagagagugagca..........................................................................................   1       1    FF1
    ...ugacagaagagagugagca..........................................................................................   22      0    FF1
    ...ugacagaagagaCugagca..........................................................................................   1       1    FF1
```

```
   Mature                                                             Star
cggugacagaagagagugagcacacggccggucguuacggcaccgcccgggugugccgucgcggccgcgugcucacugcucuuccugucauccacucucccgcuucccuucc
...ugCcagaagagagugagca..........................................................................................    1      1    FF1
...ugacagaagagaCugagcac.........................................................................................    2      1    FF1
...ugacagaagagagugagGac.........................................................................................    6      1    FF1
...ugacagaaAagagugagcac.........................................................................................    3      1    FF1
...ugacagaagagagugagAac.........................................................................................    5      1    FF1
...ugacagaagagagugaCcac.........................................................................................    1      1    FF1
...ugacagaagagaUugagcac.........................................................................................    7      1    FF1
...ugacagaagagagugagcaA.........................................................................................    2      1    FF1
...ugacagaagagagGgagcac.........................................................................................    8      1    FF1
...ugacagaGgagagugagcac.........................................................................................    1      1    FF1
...ugacagaagagagugaAcac.........................................................................................    1      1    FF1
...ugacagaagagagAgagcac.........................................................................................    2      1    FF1
...Agacagaagagagugagcac.........................................................................................    1      1    FF1
...ugacagGagagagugagcac.........................................................................................    2      1    FF1
...ugacagCagagagugagcac.........................................................................................    1      1    FF1
...ugacagaagagagugaUcac.........................................................................................    1      1    FF1
...ugacagaagagagugagcCc.........................................................................................    2      1    FF1
...ugacagaagaAagugagcac.........................................................................................    1      1    FF1
...ugGcagaagagagugagcac.........................................................................................    2      1    FF1
...ugaAagaagagagugagcac.........................................................................................    4      1    FF1
...ugacagaagagCgugagcac.........................................................................................    1      1    FF1
...ugacagaagagagugagcGc.........................................................................................    1      1    FF1
...Ggacagaagagagugagcac.........................................................................................    3      1    FF1
...uUacagaagagagugagcac.........................................................................................    2      1    FF1
...ugacagaagagagugagcac.........................................................................................    2949   0    FF1
...ugaUagaagagagugagcac.........................................................................................    1      1    FF1
...ugacagaagagagugGgcac.........................................................................................    11     1    FF1
...ugacagaaCagagugagcac.........................................................................................    1      1    FF1
...ugacagaagagagugagcaU.........................................................................................    11     1    FF1
...ugacagaagagagugCgcac.........................................................................................    2      1    FF1
...uAacagaagagagugagcac.........................................................................................    1      1    FF1
...ugaGagaagagagugagcac.........................................................................................    1      1    FF1
...ugacagaagagGgugagcac.........................................................................................    2      1    FF1
...ugacagaagagaAugagcac.........................................................................................    3      1    FF1
...ugacagaagagagugagcacU........................................................................................    151    1    FF1
...ugacagaagagagugagcaca........................................................................................    21     0    FF1
.....acagaagagagugagcacacgU.....................................................................................    1      1    FF1
......cagaagagagugagcaca........................................................................................    1      0    FF1
.....................................................................ugcucacugcucuuccugucauc....................    42     0    FF1
.....................................................................ugcucacugcucuuccugucaucG...................    1      1    FF1
......................................................................gcucacugcucuuccugucau.....................    2      0    FF1
......................................................................gcucacugcucuuccGgucauc....................    1      1    FF1
......................................................................gcucacuAcucuuccugucauc....................    1      1    FF1
......................................................................gcucacugcucuuccugucauc....................    170    0    FF1
......................................................................gcucacugcucAuccugucaucc...................    1      1    FF1
......................................................................gcucacugcucuuccugCcaucc...................    1      1    FF1
......................................................................gcucacugcucuuccGgucaucc...................    1      1    FF1
......................................................................gcucacugcucuuccugucaucc...................    27     0    FF1
.......................................................................cucacugcucuuccugucauc....................    1      0    FF1
.......................................................................cucacugcucuuccugucaucc...................    1      0    FF1
........................................................................ucacugcucuuccugucaucc...................    1      0    FF1
........................................................................ucacugcucuuccugucauccac.................    1      0    FF1
```
