# Supplementary material for: Identification of Novel miRNAs and miRNA Expression Profiling in Wheat Hybrid Necrosis
Source: PLoS One. 2015 Feb 23;10(2):e0117507. doi: 10.1371/journal.pone.0117507 (PMC4338152; doi:10.1371/journal.pone.0117507)
Supplement: S2 Fig — Red colored letter: mature miRNA sequence; yellow colored letter: loop sequence; blue colored letter: miRNA* sequence. (ZIP) [file pone.0117507.s002.zip › Figures s1/contig1379187_11263.pdf]

Provisional ID	:	contig1379187_11263
Score total	:	0.6
Score for star read(s)	:	-1.3
Score for read counts	:	0
Score for mfe	:	0.9
Score for randfold	:	1.6
Score for cons. seed	:	-0.6
Total read count	:	23
Mature read count	:	23
Loop read count	:	0
Star read count	:	0

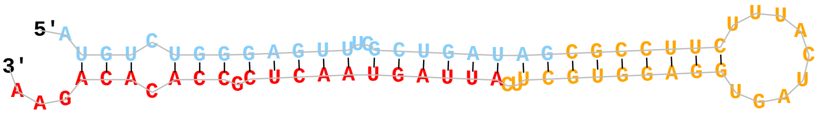

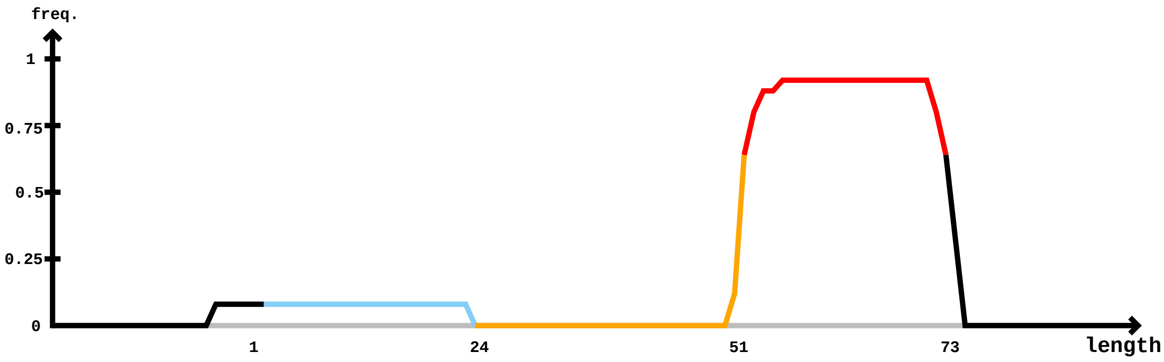

Star Mature

```
5'- uucaugggaggccaagaccgaugucugggaguuucgcugauagcgccuucuuuacuaguggaggugcuucauuaguaacucgccacacagaaaaaaugauagauuuucuu -3'    exp
    .((.(((....))).))....(((.((((((((..(((((((((((((((.........)))))))))..))))))))))).))).)))......................   reads    mm    sample
    ...............gaAcgaugucugggaguuucgcugaua.....................................................................   2        1     NN8
    .....................................................................cauuaguaacucgccacacagaa...................   3        0     NN8
    ......................................................................auuaguaacucgccacacag.....................   2        0     NN8
    ......................................................................auuaguaacucgccacacaga....................   1        0     NN8
    ......................................................................auuaguaacucgccacacagaa...................   6        0     NN8
    .......................................................................uuaguaacucgccacacaga....................   1        0     NN8
    .......................................................................uuaguaacucgccacacagaa...................   3        0     NN8
    ........................................................................uaguaacucgccacacag.....................   1        0     NN8
    ........................................................................uaguaacucgccacacagaa...................   1        0     NN8
    ..........................................................................guaacucgccacacagaa...................   1        0     NN8

    ......................................................................auuaguaacucgccacacaga....................   2        0     FF1
    ......................................................................auuaguaacucgccacacagaa...................   2        0     FF1
```
