# Supplementary material for: Identification of Novel miRNAs and miRNA Expression Profiling in Wheat Hybrid Necrosis
Source: PLoS One. 2015 Feb 23;10(2):e0117507. doi: 10.1371/journal.pone.0117507 (PMC4338152; doi:10.1371/journal.pone.0117507)
Supplement: S2 Fig — Red colored letter: mature miRNA sequence; yellow colored letter: loop sequence; blue colored letter: miRNA* sequence. (ZIP) [file pone.0117507.s002.zip › Figures s1/contig1423883_11407.pdf]

Provisional ID	:	contig1423883_11407
Score total	:	44.3
Score for star read(s)	:	3.9
Score for read counts	:	33.8
Score for mfe	:	2
Score for randfold	:	1.6
Score for cons. seed	:	3
Total read count	:	78
Mature read count	:	59
Loop read count	:	2
Star read count	:	17

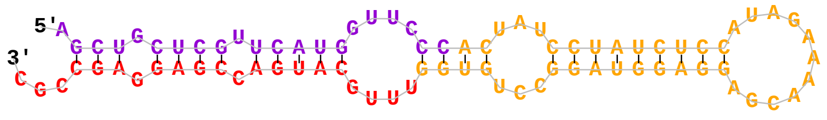

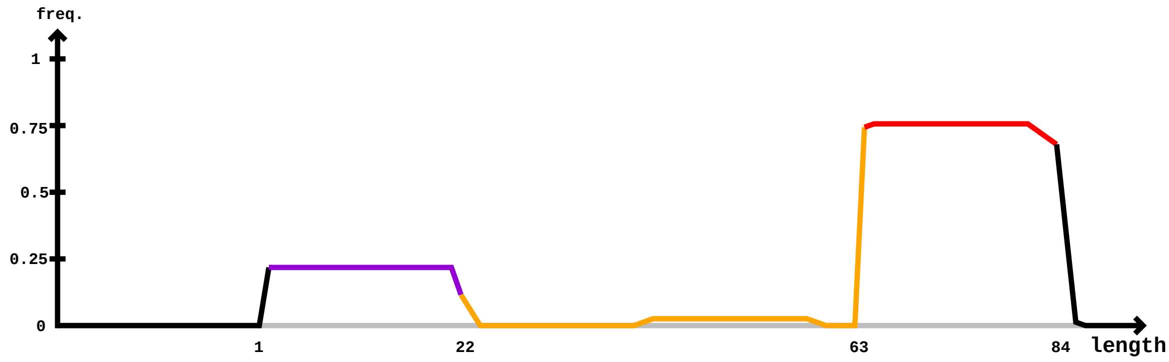

Star Mature

```
5'- ucuaccggaaggguuugugcagcugcucguucaugguucccacuauccuaucuccauagaaaacgaggagguaggccugugguuugcaugaccgaggagccgcuucgaucc   -3'    obs
    ucuaccggaaggguuugugcagcugcucguucaugguucccacuauccuaucuccauagaaaacgaggagguaggccugugguuugcaugaccgaggagccgcuucgaucc          exp
    ....((....))..(((.((.(((.((((.(((((....((((...(((((((((...........)))))))))...))))....))))).)))).))).))..)))...   reads   mm   sample
    ....................agcugcucguucaugguucc.......................................................................   3       0    NN8
    ..................................................................................uuugcaugaccgaggagccgcu.......   1       0    NN8

    ....................agcugcucguucaugguucc.......................................................................   4       0    FF1
    ....................agcugcuUguucaugguucc.......................................................................   1       1    FF1
    ....................agcugcuUguucaugguuccc......................................................................   2       1    FF1
    ....................agcugcucguucaugguuccc......................................................................   7       0    FF1
    ...........................................................aaaacgaggagguaggcc..................................   1       0    FF1
    ............................................................aaacgaggagAuaggccu.................................   1       1    FF1
    ..................................................................................uuugcaugaccgaggagc...........   2       0    FF1
    ..................................................................................uuugcaugaccgaggagcc..........   2       0    FF1
    ..................................................................................uuugcaugaccgaggagccg.........   1       0    FF1
    ..................................................................................uuugcaugacUgaggagccg.........   1       1    FF1
    ..................................................................................uuugcaugaccgaggagccgU........   1       1    FF1
    ..................................................................................Guugcaugaccgaggagccgc........   1       1    FF1
    ..................................................................................uuugcaugaccgaggagccgc........   35      0    FF1
    ..................................................................................Guugcaugaccgaggagccgcu.......   1       1    FF1
    ..................................................................................uuugcaugaccgaggagccgcG.......   1       1    FF1
    ..................................................................................Auugcaugaccgaggagccgcu.......   1       1    FF1
    ..................................................................................uuugcaugaccgaggagccgcu.......   10      0    FF1
    ..................................................................................uuugcaugaccgaggagccgcuu......   1       0    FF1
    ...................................................................................uugcaugaccgaggagccgcu.......   1       0    FF1
```
