# Supplementary material for: Identification of Novel miRNAs and miRNA Expression Profiling in Wheat Hybrid Necrosis
Source: PLoS One. 2015 Feb 23;10(2):e0117507. doi: 10.1371/journal.pone.0117507 (PMC4338152; doi:10.1371/journal.pone.0117507)
Supplement: S2 Fig — Red colored letter: mature miRNA sequence; yellow colored letter: loop sequence; blue colored letter: miRNA* sequence. (ZIP) [file pone.0117507.s002.zip › Figures s1/contig143877_3348.pdf]

Provisional ID	:	contig143877_3348
Score total	:	5
Score for star read(s)	:	-1.3
Score for read counts	:	0
Score for mfe	:	1.7
Score for randfold	:	1.6
Score for cons. seed	:	3
Total read count	:	34
Mature read count	:	34
Loop read count	:	0
Star read count	:	0

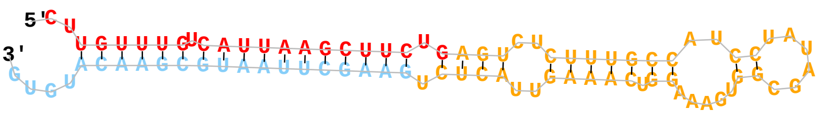

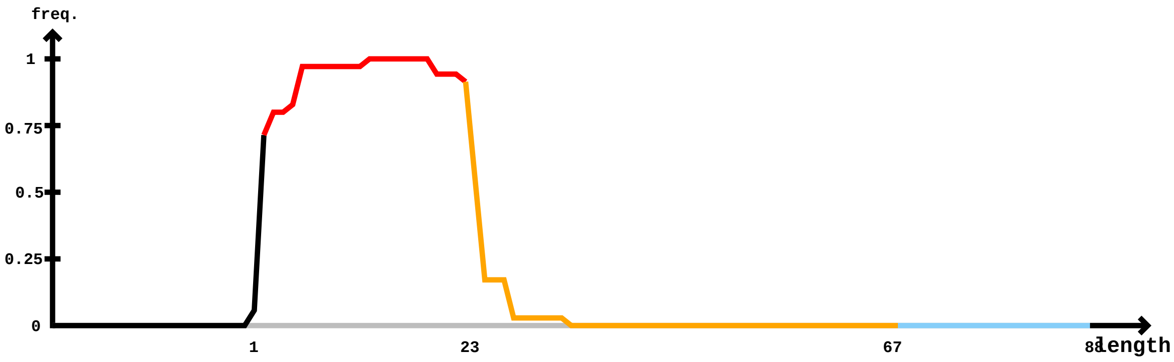

Mature Star

```
5'- cuaucaagauuuguuugccacuuguuugucauuaagcuucugagucucuuugccauccuauagcggugaaaggucaaaguuacucugaagcuuaaugcgaacaugugaccaa  -3'   exp
    .................(((.((((((.((((((((((.((((..(((((((..((......)).....)).)))))..)))).))))))))))))))))).))).....   reads   mm   sample
    ....................cuuguuugucauuaagcuucug......................................................................   1       0    NN8
    .......................guuugucauuaagcuucug......................................................................   1       0    NN8

    ...................acuuguuugucauuaagcu..........................................................................   1       0    FF1
    ...................acuuguuugucauuaagcuucu.......................................................................   1       0    FF1
    ....................cuuguuugucauuaagcu..........................................................................   1       0    FF1
    ....................cuuguuugucauuaagcuucug......................................................................   20      0    FF1
    ....................Guuguuugucauuaagcuucug......................................................................   1       1    FF1
    .....................uuguuugucauuaagcuucug......................................................................   3       0    FF1
    ........................uuugucauuaagcuucugaguc..................................................................   5       0    FF1
    ...............................uuaagcuucugagucucuuug............................................................   1       0    FF1
```
