# Supplementary material for: Identification of Novel miRNAs and miRNA Expression Profiling in Wheat Hybrid Necrosis
Source: PLoS One. 2015 Feb 23;10(2):e0117507. doi: 10.1371/journal.pone.0117507 (PMC4338152; doi:10.1371/journal.pone.0117507)
Supplement: S2 Fig — Red colored letter: mature miRNA sequence; yellow colored letter: loop sequence; blue colored letter: miRNA* sequence. (ZIP) [file pone.0117507.s002.zip › Figures s1/contig1461569_11549.pdf]

Provisional ID	:	contig1461569_11549
Score total	:	2030.1
Score for star read(s)	:	3.9
Score for read counts	:	2019
Score for mfe	:	2.5
Score for randfold	:	1.6
Score for cons. seed	:	3
Total read count	:	3972
Mature read count	:	3406
Loop read count	:	0
Star read count	:	566

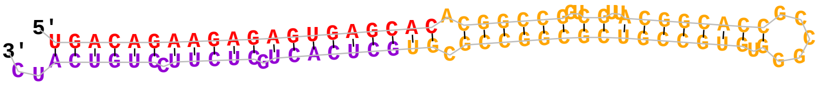

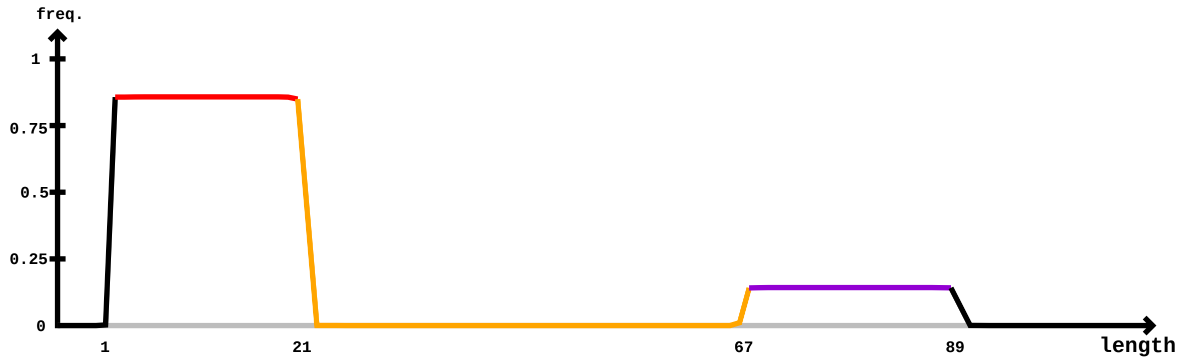

Mature   Star

```
5'- gcggugacagaagagagugagcacacggccggucguuacggcaccgccgggugugccgucgcggccgcgugcucacugcucuuccugucauccacucucccgcuuccuuccg  -3'  obs
    gcggugacagaagagagugagcacacggccggucguuacggcaccgccgggugugccgucgcggccgcgugcucacugcucuuccugucauccacucucccgcuuccuuccg       exp
    ((((((((((((((((((((((((.((((((..((..((((((((.....).))))))))))))))).)))))))))).))))).)))))).........)))).........  reads  mm  sample
    ....ugacagaagagagugagc..........................................................................................  1      0   NN8
    ....ugacagaagagagugagca.........................................................................................  2      0   NN8
    ....ugacagaagagagugagcac........................................................................................  135    0   NN8
    ....ugacagaagagagugUgcac........................................................................................  1      1   NN8
    ....ugacagaagagagugagcaU........................................................................................  1      1   NN8
    ....Agacagaagagagugagcac........................................................................................  1      1   NN8
    ....ugacagaagagGgugagcac........................................................................................  1      1   NN8
    ....ugacagaagagagugagcacU.......................................................................................  11     1   NN8
    ......acagaagagagugagcac........................................................................................  1      0   NN8
    ......................................................................gcAcacugcucuuccuguca......................  1      1   NN8
    ......................................................................gcucacugcucuuccuguca......................  1      0   NN8
    ......................................................................gcucacuAcucuuccugucauc....................  1      1   NN8
    ......................................................................gcucacugcucuuccugucUuc....................  1      1   NN8
    ......................................................................gcucaAugcucuuccugucauc....................  1      1   NN8
    ......................................................................gcucacugcucuuccuguAauc....................  1      1   NN8
    ......................................................................gcucacugcucuuccugAcauc....................  1      1   NN8
    ......................................................................gcucacugcGcuuccugucauc....................  1      1   NN8
    ......................................................................gcucacugcucuuccugucauc....................  263    0   NN8
    ......................................................................gcucacugcucuuccuguUauc....................  1      1   NN8
    ......................................................................gcucacugcucuuccuguGauc....................  1      1   NN8
    ......................................................................gcucacugcucuuccuAucauc....................  1      1   NN8
    ......................................................................gcucacugcucuuccugucaucc...................  38     0   NN8
    ......................................................................gcGcacugcucuuccugucaucc...................  1      1   NN8
    ......................................................................gcucacugcucuuccugucauccU..................  1      1   NN8
    ........................................................................cucacugcucuuccugucauc....................  1      0   NN8

    ...gugacagaagagagugagca.........................................................................................  2      0   FF1
    ...Augacagaagagagugagcac........................................................................................  2      1   FF1
    ...gugacagaagagagugagcac........................................................................................  5      0   FF1
    ....ugacagaagagagugagc..........................................................................................  3      0   FF1
    ....Ggacagaagagagugagca.........................................................................................  1      1   FF1
    ....ugacagaagagagugagca.........................................................................................  22     0   FF1
    ....ugacagaagagaCugagca.........................................................................................  1      1   FF1
```

Mature **Star**

```
gcggugacagaagagagugagcacacggccggucguuacggcaccgccgggugugccgucgcggccgcgugcucacugcucuuccugucauccacucucccgcuuccuuccg
....ugCcagaagagagugagca.........................................................................................   1     1   FF1
....ugacagaagagaCugagcac........................................................................................   2     1   FF1
....ugacagaagagagugagGac........................................................................................   6     1   FF1
....ugacagaaAagagugagcac........................................................................................   3     1   FF1
....ugacagaagagagugagAac........................................................................................   5     1   FF1
....ugacagaagagagugaCcac........................................................................................   1     1   FF1
....ugacagaagagaUugagcac........................................................................................   7     1   FF1
....ugacagaagagagugagcaA........................................................................................   2     1   FF1
....ugacagaagagagGgagcac........................................................................................   8     1   FF1
....ugacagaGgagagugagcac........................................................................................   1     1   FF1
....ugacagaagagagugaAcac........................................................................................   1     1   FF1
....ugacagaagagagAgagcac........................................................................................   2     1   FF1
....Agacagaagagagugagcac........................................................................................   1     1   FF1
....ugacagGagagagugagcac........................................................................................   2     1   FF1
....ugacagCagagagugagcac........................................................................................   1     1   FF1
....ugacagaagagagugaUcac........................................................................................   1     1   FF1
....ugacagaagagagugagcCc........................................................................................   2     1   FF1
....ugacagaagaAagugagcac........................................................................................   1     1   FF1
....ugGcagaagagagugagcac........................................................................................   2     1   FF1
....ugaAagaagagagugagcac........................................................................................   4     1   FF1
....ugacagaagagCgugagcac........................................................................................   1     1   FF1
....ugacagaagagagugagcGc........................................................................................   1     1   FF1
....Ggacagaagagagugagcac........................................................................................   3     1   FF1
....uUacagaagagagugagcac........................................................................................   2     1   FF1
....ugacagaagagagugagcac........................................................................................   2949  0   FF1
....ugaUagaagagagugagcac........................................................................................   1     1   FF1
....ugacagaagagagugGgcac........................................................................................   11    1   FF1
....ugacagaaCagagugagcac........................................................................................   1     1   FF1
....ugacagaagagagugagcaU........................................................................................   11    1   FF1
....ugacagaagagagugCgcac........................................................................................   2     1   FF1
....uAacagaagagagugagcac........................................................................................   1     1   FF1
....ugaGagaagagagugagcac........................................................................................   1     1   FF1
....ugacagaagagGgugagcac........................................................................................   2     1   FF1
....ugacagaagagaAugagcac........................................................................................   3     1   FF1
....ugacagaagagagugagcacU.......................................................................................   151   1   FF1
....ugacagaagagagugagcaca.......................................................................................   21    0   FF1
......acagaagagagugagcacacgU....................................................................................   1     1   FF1
.......cagaagagagugagcaca.......................................................................................   1     0   FF1
.....................................................................ugcucacugcucuuccugucauc....................   42    0   FF1
.....................................................................ugcucacugcucuuccugucaucG...................   1     1   FF1
......................................................................gcucacugcucuuccugucau.....................   2     0   FF1
......................................................................gcucacugcucuuccGgucauc....................   1     1   FF1
......................................................................gcucacuAcucuuccugucauc....................   1     1   FF1
......................................................................gcucacugcucuuccugucauc....................   170   0   FF1
......................................................................gcucacugcucAuccugucaucc...................   1     1   FF1
......................................................................gcucacugcucuuccugCcaucc...................   1     1   FF1
......................................................................gcucacugcucuuccGgucaucc...................   1     1   FF1
......................................................................gcucacugcucuuccugucaucc...................   27    0   FF1
.......................................................................cucacugcucuuccugucauc....................   1     0   FF1
.......................................................................cucacugcucuuccugucaucc...................   1     0   FF1
........................................................................ucacugcucuuccugucaucc...................   1     0   FF1
........................................................................ucacugcucuuccugucauccac.................   1     0   FF1
```
