# Supplementary material for: Identification of Novel miRNAs and miRNA Expression Profiling in Wheat Hybrid Necrosis
Source: PLoS One. 2015 Feb 23;10(2):e0117507. doi: 10.1371/journal.pone.0117507 (PMC4338152; doi:10.1371/journal.pone.0117507)
Supplement: S2 Fig — Red colored letter: mature miRNA sequence; yellow colored letter: loop sequence; blue colored letter: miRNA* sequence. (ZIP) [file pone.0117507.s002.zip › Figures s1/contig1491442_11671.pdf]

Provisional ID	:	contig1491442_11671
Score total	:	0.2
Score for star read(s)	:	-1.3
Score for read counts	:	0
Score for mfe	:	0.5
Score for randfold	:	1.6
Score for cons. seed	:	-0.6
Total read count	:	211
Mature read count	:	211
Loop read count	:	0
Star read count	:	0

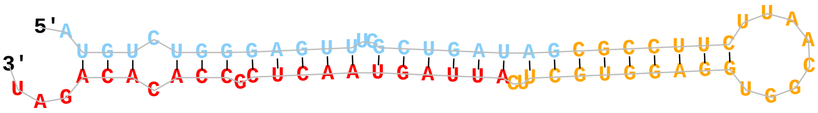

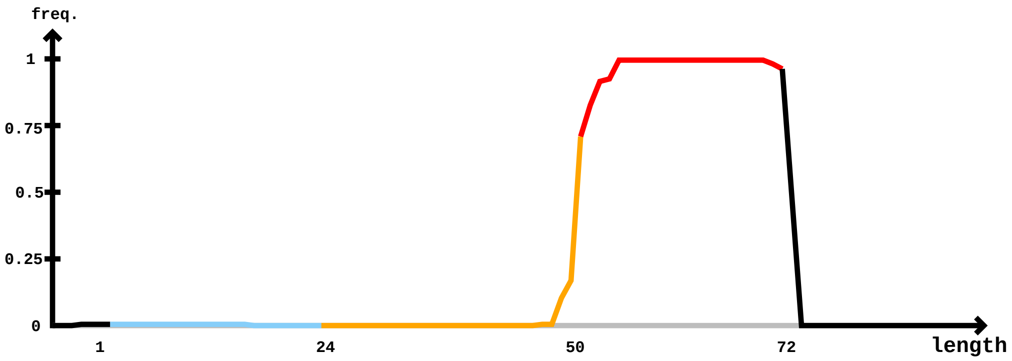

Star Mature

```
5'- aacgaugucugggaguuucgcugauagcgccuucuuaacgguggaggugcuucauuaguaacucgccacacagauacaccgcucgauuuuucuuu   -3'    exp
    .....(((.((((((((..(((((((((((((((........)))))))))..))))))))))).))).)))).......................   reads   mm    sample
    .acgaugucugggaguuuc............................................................................   1       0     NN8
    .................................................cuucauuaguaacucgcAacagau....................   1       1     NN8
    ...................................................ucauuaguaacucgccacacagau....................   21      0     NN8
    ....................................................cauuaguaacucgccacacagau....................   12      0     NN8
    .....................................................auuaguaacucgccacacag......................   2       0     NN8
    .....................................................auuaguaacucgccacacaga.....................   1       0     NN8
    .....................................................auuaguaacucgccacacagau....................   93      0     NN8
    .....................................................aGuaguaacucgccacacagau....................   1       1     NN8
    ......................................................uuaguaacucgccacacaga.....................   1       0     NN8
    ......................................................uuaguaacucgccacacagau....................   23      0     NN8
    .......................................................uaguaacucgccacacag......................   1       0     NN8
    .......................................................uaguaacucgccacacagau....................   12      0     NN8
    .......................................................uagGaacucgccacacagau....................   1       1     NN8
    ........................................................aguaacucgccacacagau....................   2       0     NN8
    .........................................................guaacucgccacacagau....................   14      0     NN8

    ....................................................cauuaguaacucgccacacagau....................   2       0     FF1
    .....................................................auuaguaacucgccacacaga.....................   2       0     FF1
    .....................................................auuaguaacucgccaGacagau....................   1       1     FF1
    .....................................................aAuaguaacucgccacacagau....................   1       1     FF1
    .....................................................auuaguaacucgccacacagau....................   13      0     FF1
    .....................................................auuagCaacucgccacacagau....................   1       1     FF1
    ......................................................uuaguaacucgccacacagau....................   1       0     FF1
    .......................................................uaguaacucgccacacagau....................   5       0     FF1
    .........................................................guaacucgccacacagau....................   1       0     FF1
```
