# Supplementary material for: Identification of Novel miRNAs and miRNA Expression Profiling in Wheat Hybrid Necrosis
Source: PLoS One. 2015 Feb 23;10(2):e0117507. doi: 10.1371/journal.pone.0117507 (PMC4338152; doi:10.1371/journal.pone.0117507)
Supplement: S2 Fig — Red colored letter: mature miRNA sequence; yellow colored letter: loop sequence; blue colored letter: miRNA* sequence. (ZIP) [file pone.0117507.s002.zip › Figures s1/contig1563490_11906.pdf]

Provisional ID	: contig1563490_11906	
Score total	:	2
Score for star read(s)	:	-1.3
Score for read counts	:	0
Score for mfe	:	2.3
Score for randfold	:	1.6
Score for cons. seed	:	-0.6
Total read count	:	21
Mature read count	:	21
Loop read count	:	0
Star read count	:	0

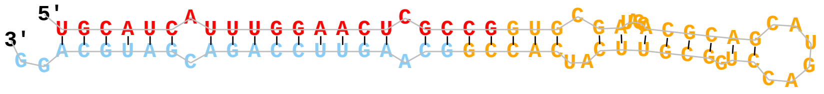

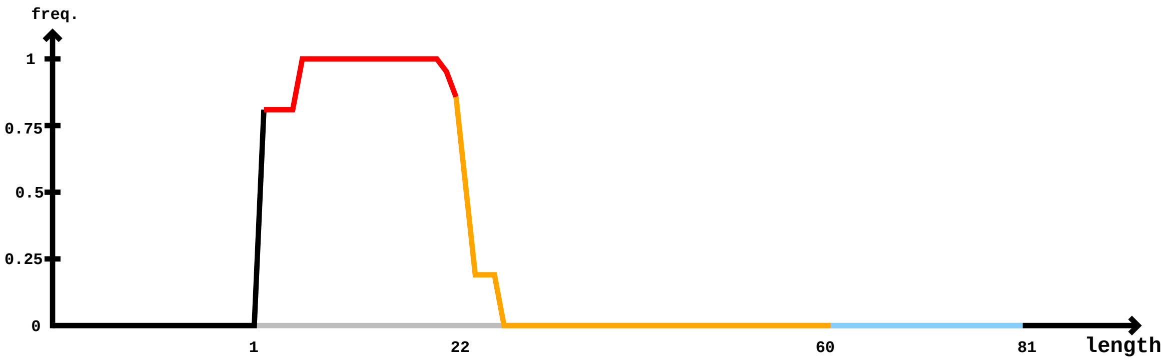

Mature Star

```
5'- uggccaccucgaggaagcccugcaucauuuggaacucgccggugcgauagcacgcagcaugaccuggcguucaucaccggcaaguuccagacgaugcaggccuucaucaag   -3'    exp
    ..........((.((((.((((((((.(((((((((.(((((((.((....((((((......)).))))))..))))))).))))))))).)))))))).)))).))...    reads    mm    sample
    ....................ugcaucauuuggaacucgc........................................................................    1        0     FF1
    ....................ugcaucauuuggaacucgcc.......................................................................    2        0     FF1
    ....................Cgcaucauuuggaacucgccg......................................................................    1        1     FF1
    ....................ugcaucauuuggaacucgccg......................................................................    11       0     FF1
    ....................ugcaucauuuggaacucgccgg.....................................................................    2        0     FF1
    .......................ucauuuggaacucgccggugc...................................................................    4        0     FF1
```
