# Supplementary material for: Identification of Novel miRNAs and miRNA Expression Profiling in Wheat Hybrid Necrosis
Source: PLoS One. 2015 Feb 23;10(2):e0117507. doi: 10.1371/journal.pone.0117507 (PMC4338152; doi:10.1371/journal.pone.0117507)
Supplement: S2 Fig — Red colored letter: mature miRNA sequence; yellow colored letter: loop sequence; blue colored letter: miRNA* sequence. (ZIP) [file pone.0117507.s002.zip › Figures s1/contig1569657_11932.pdf]

Provisional ID	:	contig1569657_11932
Score total	:	1.9
Score for star read(s)	:	-1.3
Score for read counts	:	0
Score for mfe	:	2.2
Score for randfold	:	1.6
Score for cons. seed	:	-0.6
Total read count	:	2761
Mature read count	:	2761
Loop read count	:	0
Star read count	:	0

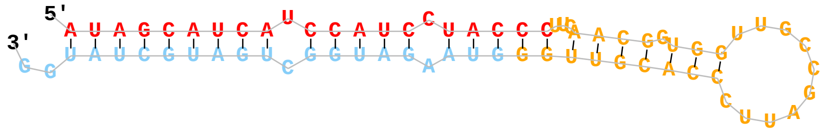

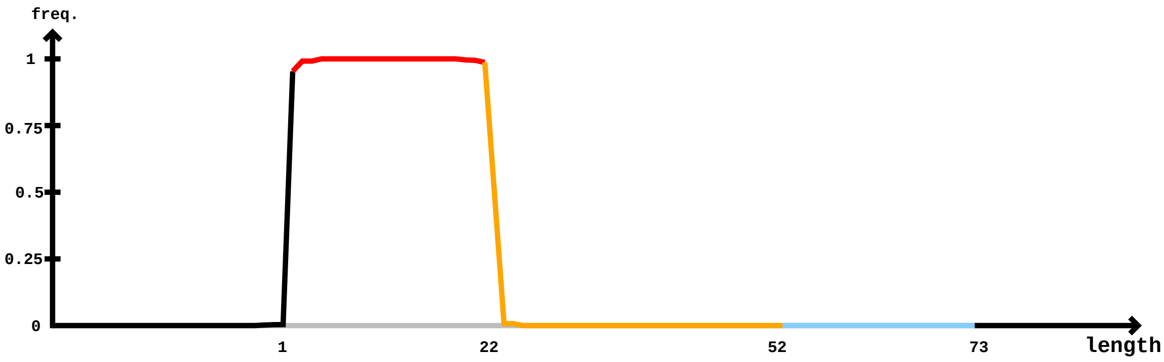

Mature Star

```
5'- agguccucugguugaacuugcccauagcaucauccauccuacccuucaacgguggguugccgauucccacguugg guaagauggcugaugcuaugg auagcuucaaaccagg   -3'    exp
```

```
5'- agguccucugguugaacuugcccauagcaucauccauccuacccuucaacgguggguugccgauucccacguuggguaagauggcugaugcuauggauagcuucaaaccagg  -3'   exp
    .......(((((((((.(((.(((((((((((.(((((.(((((...((((.(((..........))))))))))))).))))).))))))))))).))).))).)))))).   reads       mm          sample
    .....................ccauagcaucauccauccua......................................................................   4           0           NN8
    ......................cauagcaucauccauccuacc....................................................................   1           0           NN8
    .......................auagcaucauccauccuaccc...................................................................   29          0           NN8
    .......................auagcaucauccauccuacccu..................................................................   1           0           NN8
    ........................uagcaucauccauccuacccu..................................................................   1           0           NN8
    ..........................gcaucauccauccuaccc...................................................................   3           0           NN8
    ..........................gcaucauccauccuacccuuc................................................................   5           0           NN8

    ....................cccauagcaucauccauccua......................................................................   5           0           FF1
    .......................auagcaucauccauccuC......................................................................   1           1           FF1
    .......................auagcaucauccauccua......................................................................   1           0           FF1
    .......................auagcaucGuccauccuac.....................................................................   1           1           FF1
    .......................auagcaucauccauccuac.....................................................................   2           0           FF1
    .......................auagGaucauccauccuac.....................................................................   1           1           FF1
    .......................auagcaucauccauccuacc....................................................................   21          0           FF1
    .......................aAagcaucauccauccuaccc...................................................................   1           1           FF1
    .......................auagcaucauccauccuUccc...................................................................   1           1           FF1
    .......................Uuagcaucauccauccuaccc...................................................................   3           1           FF1
    .......................auagcaucaAccauccuaccc...................................................................   1           1           FF1
    .......................auagcaucauccaGccuaccc...................................................................   1           1           FF1
    .......................auagUaucauccauccuaccc...................................................................   1           1           FF1
    .......................auagcGucauccauccuaccc...................................................................   1           1           FF1
    .......................aGagcaucauccauccuaccc...................................................................   5           1           FF1
    .......................Cuagcaucauccauccuaccc...................................................................   1           1           FF1
    .......................auagcauGauccauccuaccc...................................................................   1           1           FF1
    .......................auagcaucUuccauccuaccc...................................................................   1           1           FF1
    .......................auagcaucauccauccuaUcc...................................................................   1           1           FF1
    .......................auagcaucauccGuccuaccc...................................................................   3           1           FF1
    .......................auagcaucauccauGcuaccc...................................................................   1           1           FF1
    .......................auagcaucauccaucAuaccc...................................................................   3           1           FF1
    .......................auagcaucauUcauccuaccc...................................................................   1           1           FF1
    .......................auagcaucGuccauccuaccc...................................................................   1           1           FF1
    .......................auagcaucauccauccAaccc...................................................................   2           1           FF1
    .......................auagcaucauccauccGaccc...................................................................   3           1           FF1
```

```
                       Mature                                          Star
agguccucugguugaacuugcccauagcaucauccauccuacccuucaacgguggcugccgauucccacguuggguaagauggcugaugcuauggauagcuucaaaccagg
.......................aCagcaucauccauccuaccc...................................................................   2     1   FF1
.......................auagcaucaucGauccuaccc...................................................................   3     1   FF1
.......................auagcaucauccauccuaAcc...................................................................   1     1   FF1
.......................auagcaucaucUauccuaccc...................................................................   1     1   FF1
.......................Guagcaucauccauccuaccc...................................................................   5     1   FF1
.......................auagGaucauccauccuaccc...................................................................   1     1   FF1
.......................auagcaucauccaucGuaccc...................................................................   6     1   FF1
.......................auagcaucauccauccCaccc...................................................................   1     1   FF1
.......................auagcaucauccUuccuaccc...................................................................   3     1   FF1
.......................auagcaucauccCuccuaccc...................................................................   5     1   FF1
.......................auagcaucauGcauccuaccc...................................................................   1     1   FF1
.......................auagcaGcauccauccuaccc...................................................................   1     1   FF1
.......................auagcaucauccaCccuaccc...................................................................   1     1   FF1
.......................auagcaucaucAauccuaccc...................................................................   3     1   FF1
.......................auagcaucauccauccuaccc...................................................................   2494  0   FF1
.......................auagcaucauccauccuaccG...................................................................   2     1   FF1
.......................auagcaucauccauccuacccu..................................................................   8     0   FF1
........................uagcaucauccauccuaccc...................................................................   4     0   FF1
........................uagcaucauccaCccuacccu..................................................................   1     1   FF1
........................uagcaucauccauccuacccC..................................................................   1     1   FF1
........................uagcaucauccauccuaGccu..................................................................   1     1   FF1
........................uagcaucauccauccuacccu..................................................................   96    0   FF1
........................uagcaucauccauccuacccA..................................................................   1     1   FF1
........................Gagcaucauccauccuacccu..................................................................   1     1   FF1
..........................gcaucauccauccuacccuu.................................................................   1     0   FF1
..........................gcaucauccauccuacccuuc................................................................   14    0   FF1
```
