# Supplementary material for: Identification of Novel miRNAs and miRNA Expression Profiling in Wheat Hybrid Necrosis
Source: PLoS One. 2015 Feb 23;10(2):e0117507. doi: 10.1371/journal.pone.0117507 (PMC4338152; doi:10.1371/journal.pone.0117507)
Supplement: S2 Fig — Red colored letter: mature miRNA sequence; yellow colored letter: loop sequence; blue colored letter: miRNA* sequence. (ZIP) [file pone.0117507.s002.zip › Figures s1/contig1597271_12023.pdf]

Provisional ID	:	contig1597271_12023
Score total	:	189.2
Score for star read(s)	:	3.9
Score for read counts	:	178.6
Score for mfe	:	2.2
Score for randfold	:	1.6
Score for cons. seed	:	3
Total read count	:	362
Mature read count	:	361
Loop read count	:	0
Star read count	:	1

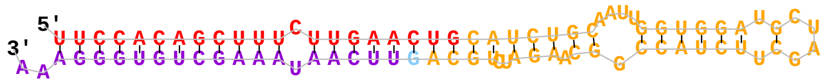

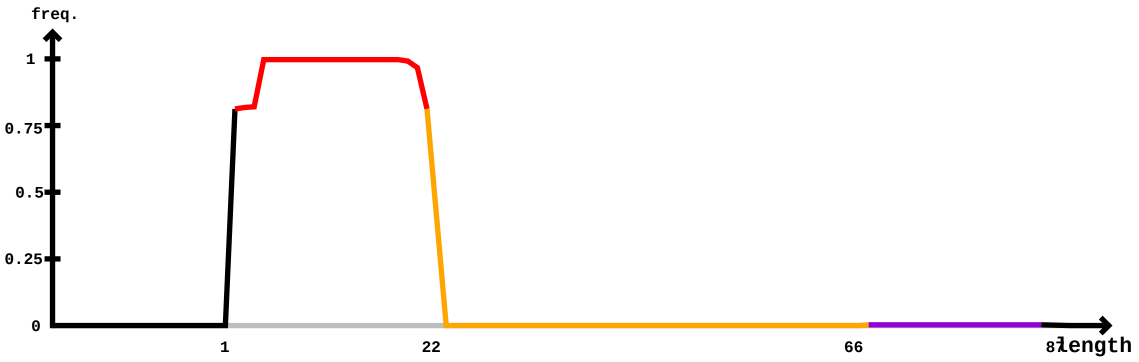


```
                                 Mature                                                              Star
5'- uggcccucuuugcugucuuccacagcuuucuugaacugcaucugcaauugguggaugcuagcuucuaccggcaagaucugcaguucaauaaagcugugggaaauugca   -3'    obs
    uggcccucuuugcugucuuccacagcuuucuugaacugcaucugcaauugguggaugcuagcuucuaccggcaagaucugcaguucaauaaagcugugggaaauugca          exp
    ..........(((.((.((((((((((((.(((((((((((((((....((((((.(....).)))))).)).)))..)))))))))).)))))))))))).)).)))   reads   mm   sample
    .................uuccacagcuuucuugaacu.......................................................................   3       0    NN8
    .................uuccacagcuuucuugaacug......................................................................   4       0    NN8
    ...................ccacagcuuAcuugaacug......................................................................   1       1    NN8
    ....................Gacagcuuucuugaacug......................................................................   1       1    NN8
    ....................cacagcuuucuugaacug......................................................................   39      0    NN8

    .................uuccacagcuuucuugaU.........................................................................   1       1    FF1
    .................uuccacagcuuucuugaa.........................................................................   1       0    FF1
    .................uuccacagcuuucuugaac........................................................................   9       0    FF1
    .................uuccacagcuuucuugaacu.......................................................................   51      0    FF1
    .................uuGcacagcuuucuugaacu.......................................................................   1       1    FF1
    .................uuccacagcCuucuugaacu.......................................................................   1       1    FF1
    .................Auccacagcuuucuugaacug......................................................................   1       1    FF1
    .................uucAacagcuuucuugaacug......................................................................   1       1    FF1
    .................uuccacagcuuucuuAaacug......................................................................   1       1    FF1
    .................uuccacagcuuucuugaacuC......................................................................   1       1    FF1
    .................uuAcacagcuuucuugaacug......................................................................   1       1    FF1
    .................Guccacagcuuucuugaacug......................................................................   1       1    FF1
    .................uuccacagcuuucuugaacug......................................................................   209     0    FF1
    .................uuccacagcuuuAuugaacug......................................................................   1       1    FF1
    .................uucUacagcuuucuugaacug......................................................................   1       1    FF1
    .................uuccacagGuuucuugaacug......................................................................   1       1    FF1
    .................uuccacagcuuucuugaaGug......................................................................   1       1    FF1
    .................uuGcacagcuuucuugaacug......................................................................   1       1    FF1
    .................uuccacagcuuucuugaacugU.....................................................................   3       1    FF1
    ..................uccacagcuuucuugaacug......................................................................   2       0    FF1
    ....................cacagcuuucuugaaGug......................................................................   1       1    FF1
    ....................cacagcuuucuugaacug......................................................................   23      0    FF1
    ...................................................................................uucaauaaagcugugggaaa.....   1       0    FF1
```
