# Supplementary material for: Identification of Novel miRNAs and miRNA Expression Profiling in Wheat Hybrid Necrosis
Source: PLoS One. 2015 Feb 23;10(2):e0117507. doi: 10.1371/journal.pone.0117507 (PMC4338152; doi:10.1371/journal.pone.0117507)
Supplement: S2 Fig — Red colored letter: mature miRNA sequence; yellow colored letter: loop sequence; blue colored letter: miRNA* sequence. (ZIP) [file pone.0117507.s002.zip › Figures s1/contig1618727_12094.pdf]

Provisional ID	:	contig1618727_12094
Score total	:	0
Score for star read(s)	:	-1.3
Score for read counts	:	-1.3
Score for mfe	:	1.7
Score for randfold	:	1.6
Score for cons. seed	:	-0.6
Total read count	:	9
Mature read count	:	9
Loop read count	:	0
Star read count	:	0

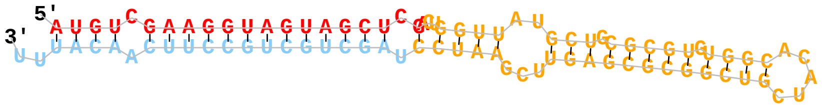

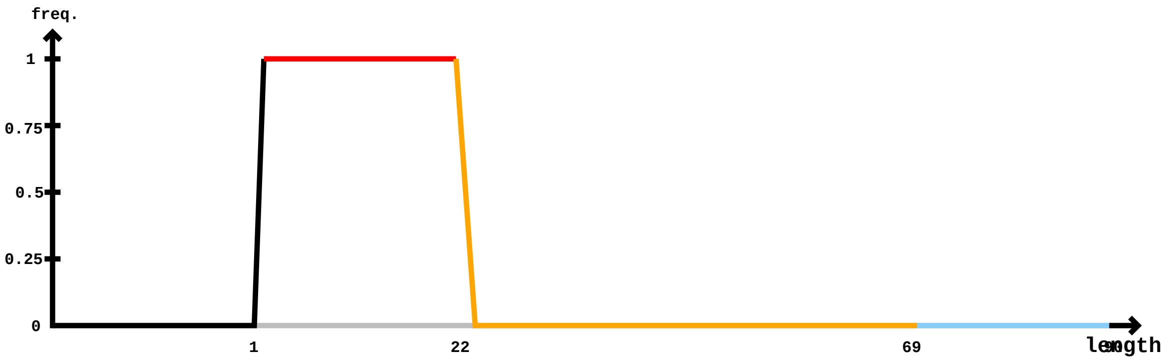

Mature   Star

```
5'- aguuucacgguaaggucugcaugucgaagguaguagcucgacugguuaugcugcgcguguggcacaucgucggcgcgaguucgaauccuagcugcugccuucaacauuuuu   -3'    exp
    .......(((......))).((((.(((((((((((((.(...((((..(((.(((((.((((.....))))))))))))...)))).)))))))))))))).))))....    reads   mm   sample
    ....................augucgaagguaguagcucga......................................................................    1       0    NN8

    ....................augucgaagguaguagcucga......................................................................    8       0    FF1
```
