# Supplementary material for: Identification of Novel miRNAs and miRNA Expression Profiling in Wheat Hybrid Necrosis
Source: PLoS One. 2015 Feb 23;10(2):e0117507. doi: 10.1371/journal.pone.0117507 (PMC4338152; doi:10.1371/journal.pone.0117507)
Supplement: S2 Fig — Red colored letter: mature miRNA sequence; yellow colored letter: loop sequence; blue colored letter: miRNA* sequence. (ZIP) [file pone.0117507.s002.zip › Figures s1/contig1644676_12166.pdf]

Provisional ID	:	contig1644676_12166
Score total	:	1.2
Score for star read(s)	:	-1.3
Score for read counts	:	0
Score for mfe	:	1.5
Score for randfold	:	1.6
Score for cons. seed	:	-0.6
Total read count	:	134
Mature read count	:	134
Loop read count	:	0
Star read count	:	0

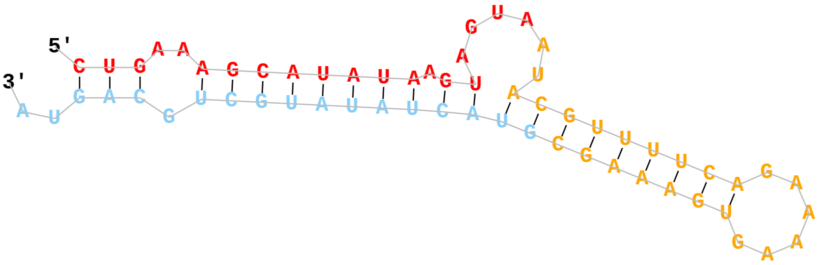

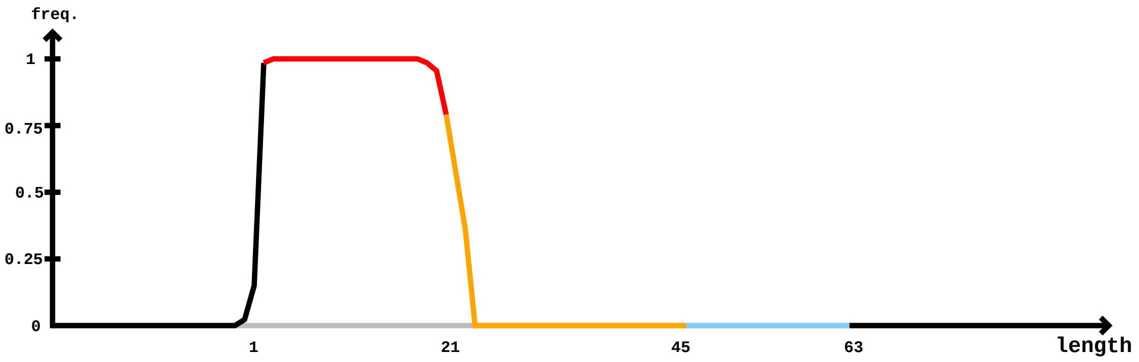

Mature Star

```
5'- caggccccugggaggagaagcugaaagcauauaaguaguaauacguuuucagaaaagugaaagcguacuauaugcugcaguagagaggaauacgcuuuccagagggaa   -3'   exp
    ....(((((((((((....((((..((((((((.((......(((((((((......))))))))))))))))))).)))).....(.....).)))))))).)))..    reads   mm    sample
    ..................Ugcugaaagcauauaaguagu....................................................................    1       1     NN8
    ...................gcugaaagcauauaagua.......................................................................    2       0     NN8
    ...................gcugaaagcauauaaguag......................................................................    1       0     NN8
    ...................gcugaaagcauauaaAuagu.....................................................................    1       1     NN8
    ...................gcugaaagcauauaaguagu.....................................................................    6       0     NN8
    ...................gcugaaagcauauaaguagua....................................................................    5       0     NN8
    ...................gcugaaagcauauaaguaguaaG..................................................................    1       1     NN8
    ....................cugaaagcauauaaguag......................................................................    3       0     NN8
    ....................cugaaagcauauaaguagu.....................................................................    9       0     NN8
    ....................cugaaagcauauaaguagua....................................................................    51      0     NN8
    ....................cugaaagcauauaaguaguaaG..................................................................    45      1     NN8
    .....................ugaaagcauauaaguagu.....................................................................    1       0     NN8

    ..................Ugcugaaagcauauaaguagu....................................................................    2       1     FF1
    ...................gcugaaagcauauaaguagu.....................................................................    1       0     FF1
    ....................cugaaagcauauaaguagua....................................................................    2       0     FF1
    ....................cugaaagcauauaaguaguaaG..................................................................    2       1     FF1
    .....................ugaaagcauauaaguagu.....................................................................    1       0     FF1
```
