# Supplementary material for: Identification of Novel miRNAs and miRNA Expression Profiling in Wheat Hybrid Necrosis
Source: PLoS One. 2015 Feb 23;10(2):e0117507. doi: 10.1371/journal.pone.0117507 (PMC4338152; doi:10.1371/journal.pone.0117507)
Supplement: S2 Fig — Red colored letter: mature miRNA sequence; yellow colored letter: loop sequence; blue colored letter: miRNA* sequence. (ZIP) [file pone.0117507.s002.zip › Figures s1/contig172799_3713.pdf]

Provisional ID	:	contig172799_3713
Score total	:	1.1
Score for star read(s)	:	-1.3
Score for read counts	:	0
Score for mfe	:	1.4
Score for randfold	:	1.6
Score for cons. seed	:	-0.6
Total read count	:	199
Mature read count	:	199
Loop read count	:	0
Star read count	:	0

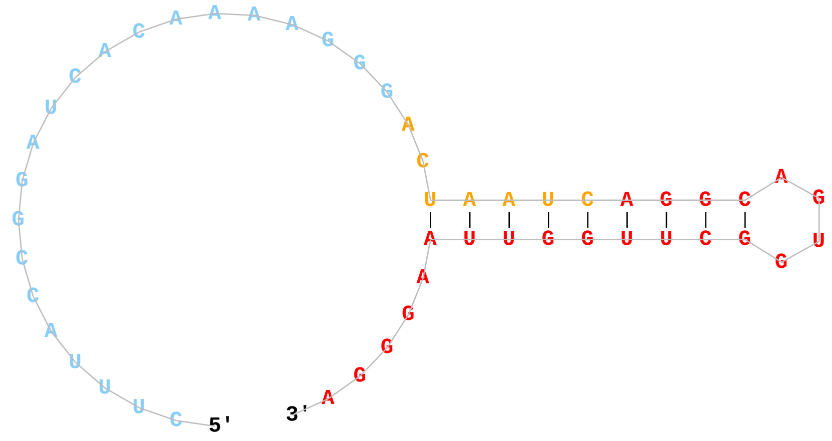

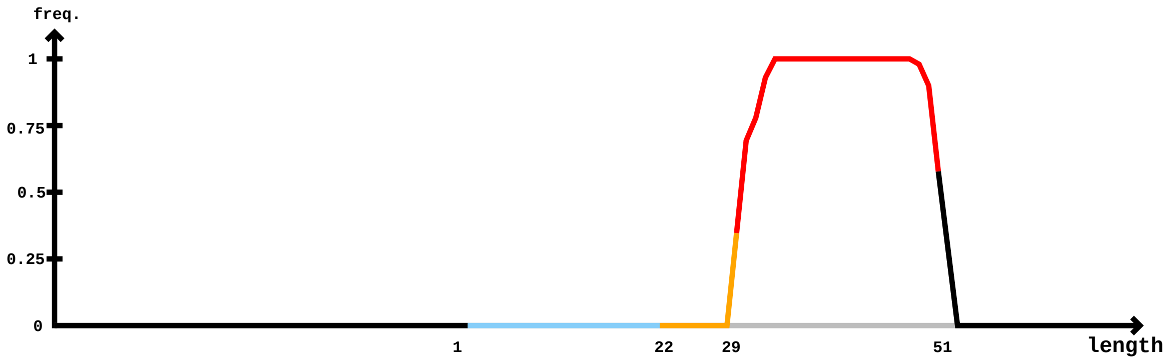

Star Mature

```
5'- agguaaacucugggaacaaaagaugaggauccuuucugcuccuuuaccggaucacaaaagggacuaaucaggcaguggcuugguuaagggaguagaacgcaccagagccgc  -3'   exp
    .((....((((((........(((....)))..(((((((((((((((((..(((......((....)).....)))..)))).)))))))))))))....))))))))..   reads      mm      sample
    .....................................................................aggcaguggcuugguuaagg......................   4          0       NN8
    .....................................................................aggcaguggcuugguuaaggg.....................   17         0       NN8
    .....................................................................aggcGguggcuugguuaaggga....................   1          1       NN8
    .....................................................................aggcaguggAuugguuaaggga....................   1          1       NN8
    .....................................................................aggcaguggcuugguuaaggga....................   39         0       NN8
    ......................................................................ggcaguggcuugguuaag.......................   3          0       NN8
    ......................................................................ggcaguggcuugguuaagg......................   6          0       NN8
    ......................................................................ggcaguggUuugguuaagg......................   1          1       NN8
    ......................................................................ggcaguggcuugguuaaggg.....................   19         0       NN8
    ......................................................................Cgcaguggcuugguuaaggg.....................   1          1       NN8
    ......................................................................ggcaguggcuugguuaaggga....................   30         0       NN8
    .......................................................................gcaguggcuugguuaagg......................   4          0       NN8
    .......................................................................gcaguggcuugguuaaggg.....................   6          0       NN8
    .......................................................................gcaguggcuugguuaaggga....................   5          0       NN8
    ........................................................................caguggcuugguuaaggg.....................   13         0       NN8
    ........................................................................caguggcuugguuaaggga....................   12         0       NN8
    .........................................................................aguggcuugguuaaggga....................   13         0       NN8

    .....................................................................aggcaguggcuugguuaagg......................   1          0       FF1
    .....................................................................aggcaguggcuugguuaaggga....................   6          0       FF1
    ......................................................................ggcaguggcuugguuaag.......................   1          0       FF1
    ......................................................................ggcaguggcuugguuaaggg.....................   7          0       FF1
    ......................................................................ggcaguggcuugguuaaggga....................   1          0       FF1
    .......................................................................gcaguggcuugguuaaggga....................   2          0       FF1
    ........................................................................caguggcuugguuaaggg.....................   1          0       FF1
    ........................................................................caguggcuugguuaaggga....................   4          0       FF1
    .........................................................................aguggcuugguuaaggga....................   1          0       FF1
```
