# Supplementary material for: Identification of Novel miRNAs and miRNA Expression Profiling in Wheat Hybrid Necrosis
Source: PLoS One. 2015 Feb 23;10(2):e0117507. doi: 10.1371/journal.pone.0117507 (PMC4338152; doi:10.1371/journal.pone.0117507)
Supplement: S2 Fig — Red colored letter: mature miRNA sequence; yellow colored letter: loop sequence; blue colored letter: miRNA* sequence. (ZIP) [file pone.0117507.s002.zip › Figures s1/contig172807_3715.pdf]

| | | |
|---|---|---|
| Provisional ID | : | contig172807_3715 |
| Score total | : | 1.9 |
| Score for star read(s) | : | -1.3 |
| Score for read counts | : | 0 |
| Score for mfe | : | 2.2 |
| Score for randfold | : | 1.6 |
| Score for cons. seed | : | -0.6 |
| Total read count | : | 177 |
| Mature read count | : | 177 |
| Loop read count | : | 0 |
| Star read count | : | 0 |

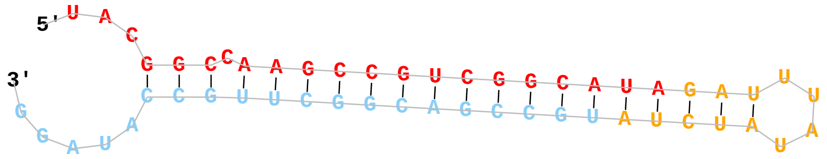


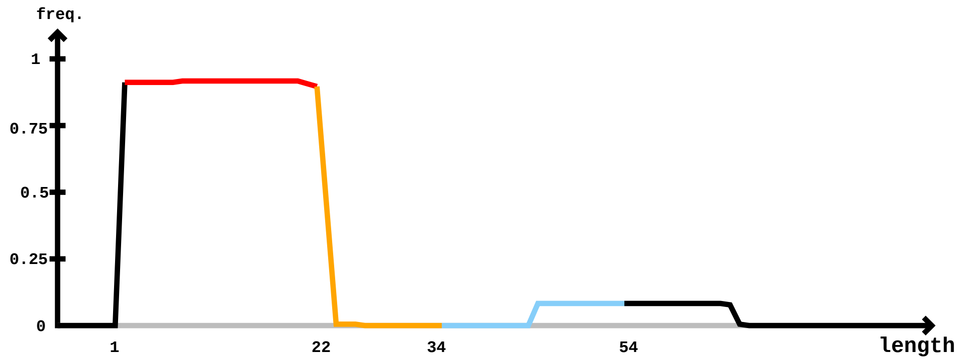


**Mature** **Star**

```
5'- gugccuacggccaagccgucggcauagauuuauaucuaugccgacggcuugccauaggcauagcccugccacgugucgccuccugaccc   -3'    exp
    (((((((.(((.(((((((((((((((((....)))))))))))))))))))).))))))).............((((.....))))..   reads      mm         sample
    .....uacggcAaagccgucggcaua...............................................................   1          1          NN8
    ................................................uugccauaggcauagcccug.....................   1          0          NN8
    ................................................uugccGuaggcauagcccugc....................   2          1          NN8

    .....uacggcAaagccgucggca.................................................................   2          1          FF1
    .....uacggcAaagccgucggcau................................................................   2          1          FF1
    .....uacggcUaagccgucggcaua...............................................................   1          1          FF1
    .....uacggcAaagccgucggcaua...............................................................   170        1          FF1
    ..........Aaagccgucggcauagauu............................................................   1          1          FF1
    ................................................uugccGuaggcauagcccugc....................   6          1          FF1
    ................................................uugccauaggcauagcccugc....................   6          0          FF1
    ................................................uugccauaggcaAagcccugcc...................   1          1          FF1
```
