# Supplementary material for: Identification of Novel miRNAs and miRNA Expression Profiling in Wheat Hybrid Necrosis
Source: PLoS One. 2015 Feb 23;10(2):e0117507. doi: 10.1371/journal.pone.0117507 (PMC4338152; doi:10.1371/journal.pone.0117507)
Supplement: S2 Fig — Red colored letter: mature miRNA sequence; yellow colored letter: loop sequence; blue colored letter: miRNA* sequence. (ZIP) [file pone.0117507.s002.zip › Figures s1/contig1769557_12487.pdf]

Provisional ID	:	contig1769557_12487
Score total	:	14
Score for star read(s)	:	3.9
Score for read counts	:	7.3
Score for mfe	:	1.9
Score for randfold	:	1.6
Score for cons. seed	:	-0.6
Total read count	:	26
Mature read count	:	19
Loop read count	:	0
Star read count	:	7

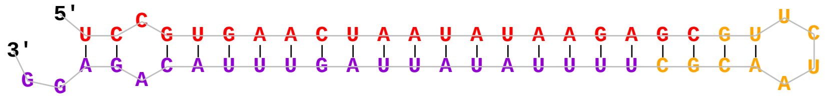

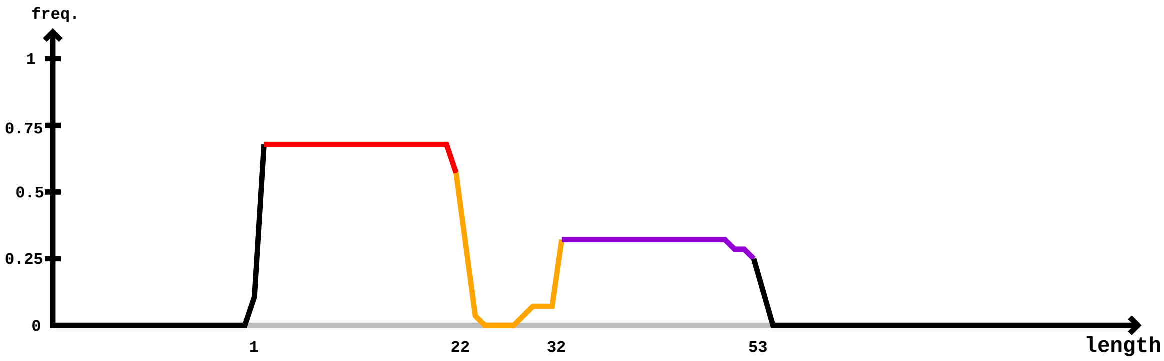

Mature Star

```
5'- ugaauauuaccuaguacuccuccgugaacuaauauaagagcguucuaacgcuuuuauauuaguuuacagaggaguaugucuuucuccuuuagcaauuauacucuggucgau   -3'    obs
    ugaauauuaccuaguacuccuccgugaacuaauauaagagcguucuaacgcuuuuauauuaguuuacagaggaguaugucuuucuccuuuagcaauuauacucuggucgau          exp
    ............((((((((.((((((((((((((((((((....)))))))))))))))))))).))))))))).....((..((..((........))..))..)).   reads     mm        sample
    ...................cuccguAaacuaauauaagag.......................................................................   1         1         NN8
    ...................cuccgugaacuaauauaagagcgu....................................................................   1         0         NN8
    ....................uccguAaacuaauauaagagc......................................................................   1         1         NN8
    ...................................................uCuuauauuaguuuacagagg.......................................   6         1         NN8

    ...................cuccguAaacuaauauaagag.......................................................................   1         1         FF1
    ....................uccgugaacuaauauaagag.......................................................................   1         0         FF1
    ....................uccguAaacuaauauaagagc......................................................................   10        1         FF1
    ....................uccgugaacuaauauaagagc......................................................................   4         0         FF1
    ...............................................acgcuuuuauauuaguuuacGgag........................................   1         1         FF1
    ................................................cgcuCuuauauuaguuuacag..........................................   1         1         FF1
    ...................................................uCuuauauuaguuuacagagg.......................................   1         1         FF1
```
