# Supplementary material for: Identification of Novel miRNAs and miRNA Expression Profiling in Wheat Hybrid Necrosis
Source: PLoS One. 2015 Feb 23;10(2):e0117507. doi: 10.1371/journal.pone.0117507 (PMC4338152; doi:10.1371/journal.pone.0117507)
Supplement: S2 Fig — Red colored letter: mature miRNA sequence; yellow colored letter: loop sequence; blue colored letter: miRNA* sequence. (ZIP) [file pone.0117507.s002.zip › Figures s1/contig17780_764.pdf]

| | | |
|---|---|---|
| Provisional ID | : | contig17780_764 |
| Score total | : | 51.5 |
| Score for star read(s) | : | 3.9 |
| Score for read counts | : | 44 |
| Score for mfe | : | 2.6 |
| Score for randfold | : | 1.6 |
| Score for cons. seed | : | -0.6 |
| Total read count | : | 98 |
| Mature read count | : | 97 |
| Loop read count | : | 0 |
| Star read count | : | 1 |

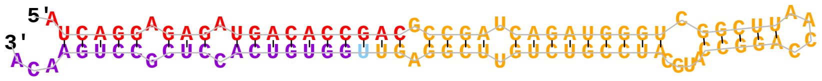


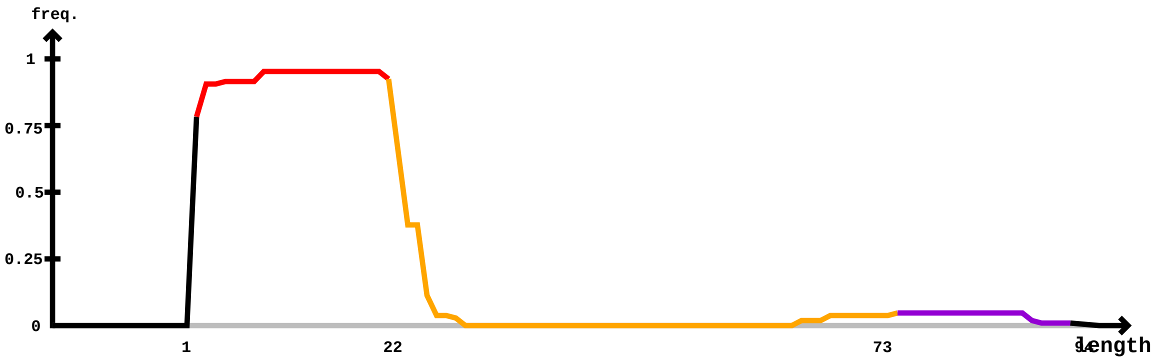


**Mature** **Star**

```
5'- uuaggguccugugaucaggagagaugacaccgacgccgaucagaugggucggcuuaaccaggccaugcauccgucuguucggaguuggugucaccucgccugaacauagg  -3'   obs
    uuaggguccugugaucaggagagaugacaccgacgccgaucagaugggucggcuuaaccaggccaugcauccgucuguucggaguuggugucaccucgccugaacauagg        exp
    .......((((((.(((((.(((.((((((((((.((((.(((((((((.(((((....)))))....))))))))).)))).))))))))))).))).))))).))))))   reads     mm        sample
    .............aucaggagagaugacaccgaA............................................................................   1         1         NN8
    .............aucaggagagaugacaccgac............................................................................   2         0         NN8
    .............aucaggagagaugacaccgacgcc.........................................................................   1         0         NN8
    ....................gagaugacaccgacgccgauU.....................................................................   1         1         NN8

    .............aucaggagagaugacaccgU.............................................................................   1         1         FF1
    .............aucaggagagaugacaccga.............................................................................   2         0         FF1
    .............aucaggagagaugacaccCac............................................................................   1         1         FF1
    .............aucaggagagaugacaccgac............................................................................   48        0         FF1
    .............aucaggagagaugacaccgacg...........................................................................   1         0         FF1
    .............aucaggagagaugacaccgacU...........................................................................   1         1         FF1
    .............aucaggagagaugacaccgacgcc.........................................................................   25        0         FF1
    ..............ucaggagagaugacaccgac............................................................................   1         0         FF1
    ..............ucaggagagaugacaccgacg...........................................................................   2         0         FF1
    ..............ucaggagagaugacaccgacgcc.........................................................................   2         0         FF1
    ..............ucaggagagaugacaccgacgccU........................................................................   7         1         FF1
    ..............ucaggagagaugacaccgacgccg........................................................................   1         0         FF1
    ................aggagagaugacaccgac............................................................................   1         0         FF1
    ....................gagaugacaccgacgccgau......................................................................   1         0         FF1
    ....................gagaugacaccgacgccgauU.....................................................................   1         1         FF1
    ....................gagaugacaccgacgccgauc.....................................................................   1         0         FF1
    ............................................................................gGucggaguuggugucaccucgcc..........   1         1         FF1
    ............................................................................guucggaguuggugucaccucgcc..........   1         0         FF1
    ...............................................................................cggaguuggugucaccucgcc..........   1         0         FF1
    ...............................................................................cggaguuggugucaccucgccu.........   1         0         FF1
    ......................................................................................ggugucaccucgccugaaca....   1         0         FF1
```
