# Supplementary material for: Identification of Novel miRNAs and miRNA Expression Profiling in Wheat Hybrid Necrosis
Source: PLoS One. 2015 Feb 23;10(2):e0117507. doi: 10.1371/journal.pone.0117507 (PMC4338152; doi:10.1371/journal.pone.0117507)
Supplement: S2 Fig — Red colored letter: mature miRNA sequence; yellow colored letter: loop sequence; blue colored letter: miRNA* sequence. (ZIP) [file pone.0117507.s002.zip › Figures s1/contig1816694_12607.pdf]

Provisional ID	: contig1816694_12607	
Score total	:	1.2
Score for star read(s)	:	-1.3
Score for read counts	:	0
Score for mfe	:	1.7
Score for randfold	:	-2.2
Score for cons. seed	:	3
Total read count	:	33
Mature read count	:	33
Loop read count	:	0
Star read count	:	0

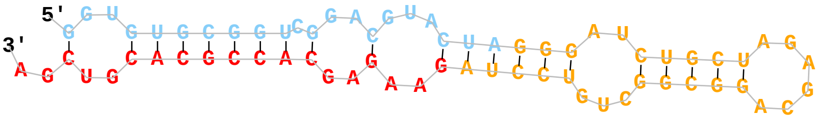

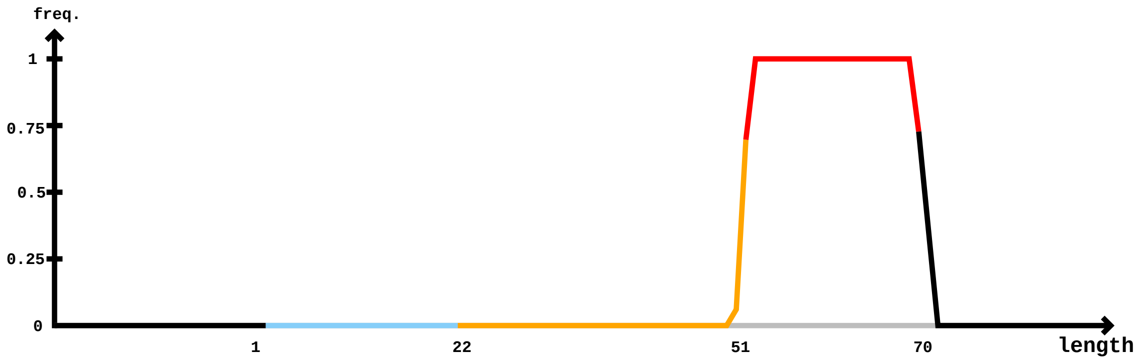


```
                      Star                                             Mature
5'- cgaagaguccggcaccgucuggugugcggucggacguacuagggaucugcuagagcaggcggcuguccuagaagagcaccgcacgucgauggcuacggugauugaaag   -3'    exp
    ........(((...(((((.(..(((((((.(..(...((((((..(((((......)))))...))))))..)..))))))))..))))))...)))..........    reads       mm          sample
    ......................................................................gaagagcaccgcacgucg....................    7           0           NN8
    ......................................................................gaagagcaccgcacgucgC...................    10          1           NN8
    .......................................................................aagagcaccgcacgucgC...................    7           1           NN8

    .....................................................................Ggaagagcaccgcacgucg....................    2           1           FF1
    ......................................................................gaagagcaccgcacgucgC...................    4           1           FF1
    .......................................................................aagagcaccgcacgucgC...................    3           1           FF1
```
