# Supplementary material for: Identification of Novel miRNAs and miRNA Expression Profiling in Wheat Hybrid Necrosis
Source: PLoS One. 2015 Feb 23;10(2):e0117507. doi: 10.1371/journal.pone.0117507 (PMC4338152; doi:10.1371/journal.pone.0117507)
Supplement: S2 Fig — Red colored letter: mature miRNA sequence; yellow colored letter: loop sequence; blue colored letter: miRNA* sequence. (ZIP) [file pone.0117507.s002.zip › Figures s1/contig1844859_12689.pdf]

Provisional ID	:	contig1844859_12689
Score total	:	37.3
Score for star read(s)	:	3.9
Score for read counts	:	30.2
Score for mfe	:	2.2
Score for randfold	:	1.6
Score for cons. seed	:	-0.6
Total read count	:	71
Mature read count	:	69
Loop read count	:	0
Star read count	:	2

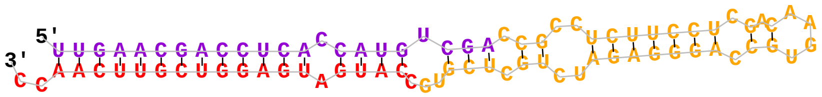

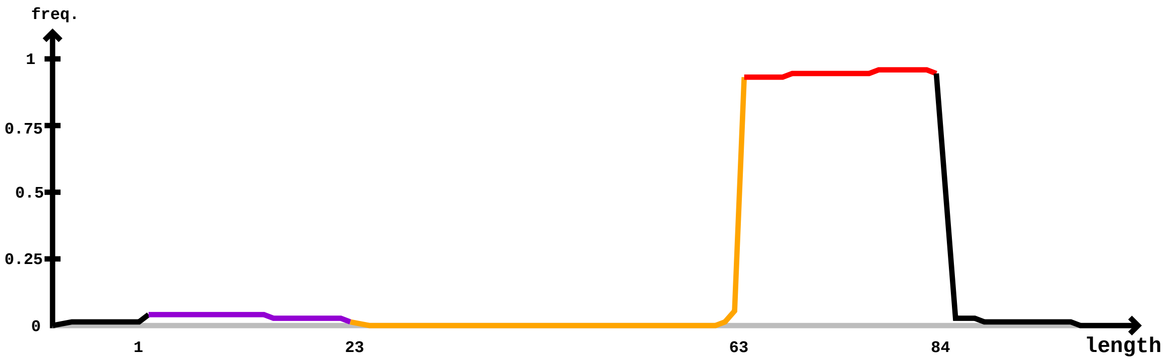

Star **Mature**

```
5'- uuugccgguugaacgaccucaccaugucgaccgccucuuccucgacaagugccagggagaucugcucgugccaugaugaggucguucaaccagcaaaacggcggcgguugc -3'    obs
    uuugccgguugaacgaccucaccaugucgaccgccucuuccucgacaagugccagggagaucugcucgugccaugaugaggucguucaaccagcaaaacggcggcgguugc         exp
    (((((.(((((((((((((((.((((.(((.((..(((((((.(.(....)).)))))))..)).)))...)))).))))))))))))))).))))).((((....)))).   reads   mm   sample
    ....................................................................ugccaCgaugaggucguucaac.....................   1       1    NN8
    .....................................................................gccaCgaugaggucguucaacc....................   3       1    NN8
    ......................................................................ccaCgaugaggucguucaacc....................   1       1    NN8
    ......................................................................ccaugaugaggucguucaacc....................   8       0    NN8

    uuugccgguugaacgaccuca..........................................................................................   1       0    FF1
    ........uugaacgaccucaccaugucg..................................................................................   1       0    FF1
    ........uugaacgaccucaccauguUga.................................................................................   1       1    FF1
    ......................................................................ccaCgaugaggucguucaacc....................   3       1    FF1
    ......................................................................ccUugaugaggucguucaacc....................   1       1    FF1
    ......................................................................ccaugaugaggucguucaacc....................   50      0    FF1
    ......................................................................ccaugaGgaggucguucaacc....................   1       1    FF1
    ...........................................................................augaggucguucaaccagca................   1       0    FF1
    ....................................................................................uucaaccagcaaaacggcggc......   1       0    FF1
```
