# Supplementary material for: Identification of Novel miRNAs and miRNA Expression Profiling in Wheat Hybrid Necrosis
Source: PLoS One. 2015 Feb 23;10(2):e0117507. doi: 10.1371/journal.pone.0117507 (PMC4338152; doi:10.1371/journal.pone.0117507)
Supplement: S2 Fig — Red colored letter: mature miRNA sequence; yellow colored letter: loop sequence; blue colored letter: miRNA* sequence. (ZIP) [file pone.0117507.s002.zip › Figures s1/contig1855197_12728.pdf]

Provisional ID	:	contig1855197_12728
Score total	:	0.1
Score for star read(s)	:	-1.3
Score for read counts	:	0
Score for mfe	:	0.4
Score for randfold	:	1.6
Score for cons. seed	:	-0.6
Total read count	:	17
Mature read count	:	16
Loop read count	:	0
Star read count	:	1

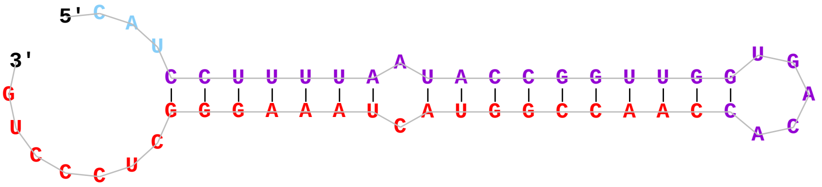

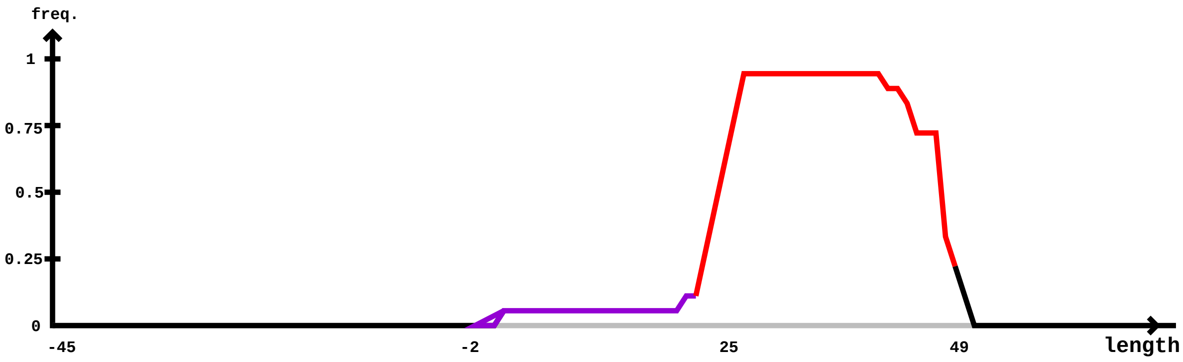

Star Mature

```
5'- cacaaauuaaaugaaaaagaaauuauaaaccaaccccccccccauccuuuuaauaccgguuggugacaccaaccgguacuaaagggcucccugcccccgaagcuggcucgugc   -3'    obs
    cacaaauuaaaugaaaaagaaauuauaaaccaacccccccccccauccuuuuaauaccgguuggugacaccaaccgguacuaaagggcucccugcccccgaagcuggcucgugc          exp
    (((..........................................(((((((.((((((((((.....)))))))))).)))))))......(((.........)))..))).   reads       mm          sample
    ..................................................................caccaaccgguacuaaagg............................   1           0           NN8
    ...................................................................accaaUcgguacuaaagggcuccc......................   1           1           NN8
    ....................................................................ccaaccgAuacuaaagggcucccu.....................   1           1           NN8
    .....................................................................caaccgguacuaaagggcucccuU....................   1           1           NN8
    ......................................................................aaccgguacuaaagggcu.........................   1           0           NN8
    ......................................................................aaUcgguacuaaagggcuccc......................   1           1           NN8

    .............................................ccuuuuaauaccgguugAugacac............................................   1           1           FF1
    ................................................................gaUaccaaccgguacuaaagggcu.........................   1           1           FF1
    ..................................................................caccaaccgguacuaaUgggc..........................   1           1           FF1
    ...................................................................accaaccgguacuaaagggcuccc......................   3           0           FF1
    ...................................................................acUaaccgguacuaaagggcuccc......................   1           1           FF1
    ....................................................................ccaaccgguacuaaagggcucccu.....................   1           0           FF1
    .....................................................................caaccgguaUuaaagggcuccc......................   1           1           FF1
    .....................................................................caaccgguacuaaagggcucccuU....................   2           1           FF1
    ......................................................................aaccgguacuaaaAggcucccugc...................   1           1           FF1
```
