# Supplementary material for: Identification of Novel miRNAs and miRNA Expression Profiling in Wheat Hybrid Necrosis
Source: PLoS One. 2015 Feb 23;10(2):e0117507. doi: 10.1371/journal.pone.0117507 (PMC4338152; doi:10.1371/journal.pone.0117507)
Supplement: S2 Fig — Red colored letter: mature miRNA sequence; yellow colored letter: loop sequence; blue colored letter: miRNA* sequence. (ZIP) [file pone.0117507.s002.zip › Figures s1/contig1884136_12833.pdf]

Provisional ID	:	contig1884136_12833
Score total	:	2
Score for star read(s)	:	-1.3
Score for read counts	:	0
Score for mfe	:	2.3
Score for randfold	:	1.6
Score for cons. seed	:	-0.6
Total read count	:	71
Mature read count	:	71
Loop read count	:	0
Star read count	:	0

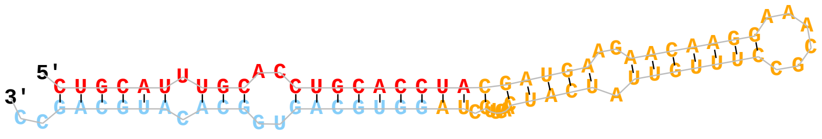

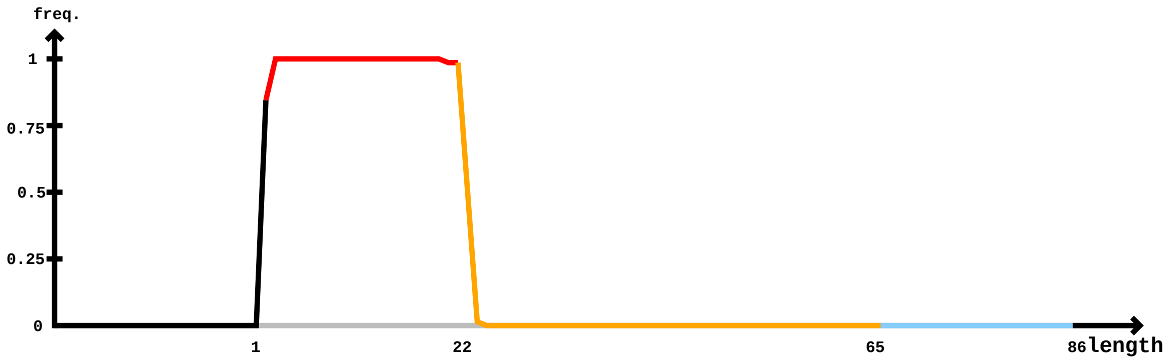

Mature **Star**

```
5'- agaagaauggcgugauauggcugcauuugcaccugcaccuacgaugaagaacaaggaaacgccuuuguuaucaucaaguugcuaggugcaguggcacaugcagccguauca   -3'    exp
    ............((((((((((((((.(((..(((((((((((((((..(((((((......))))))).))))).....).)))))))))..))).))))))))))))))     reads      mm        sample
    ....................cugcauuugcaccugcacc........................................................................     1          0         FF1
    ....................cugcauuugcaccugcaccua......................................................................     54         0         FF1
    ....................Augcauuugcaccugcaccua......................................................................     1          1         FF1
    ....................cGgcauuugcaccugcaccua......................................................................     1          1         FF1
    ....................cCgcauuugcaccugcaccua......................................................................     1          1         FF1
    ....................cugcauuugcaccugcaAcua......................................................................     1          1         FF1
    ....................cugcauuugcacAugcaccua......................................................................     1          1         FF1
    .....................ugcauuugcaccugcaccua......................................................................     1          0         FF1
    .....................ugcauuugcaccugcaccuaG.....................................................................     7          1         FF1
    .....................ugcauuugcaccugcaccuac.....................................................................     2          0         FF1
    .....................ugcauuugcaccugcaccuacU....................................................................     1          1         FF1
```
