# Supplementary material for: Identification of Novel miRNAs and miRNA Expression Profiling in Wheat Hybrid Necrosis
Source: PLoS One. 2015 Feb 23;10(2):e0117507. doi: 10.1371/journal.pone.0117507 (PMC4338152; doi:10.1371/journal.pone.0117507)
Supplement: S2 Fig — Red colored letter: mature miRNA sequence; yellow colored letter: loop sequence; blue colored letter: miRNA* sequence. (ZIP) [file pone.0117507.s002.zip › Figures s1/contig1941326_12977.pdf]

Provisional ID	:	contig1941326_12977
Score total	:	31.7
Score for star read(s)	:	3.9
Score for read counts	:	24.6
Score for mfe	:	2.2
Score for randfold	:	1.6
Score for cons. seed	:	-0.6
Total read count	:	60
Mature read count	:	57
Loop read count	:	0
Star read count	:	3

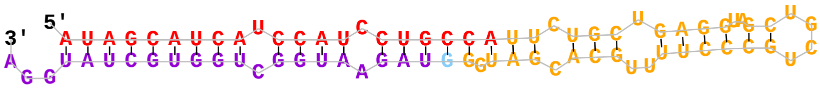

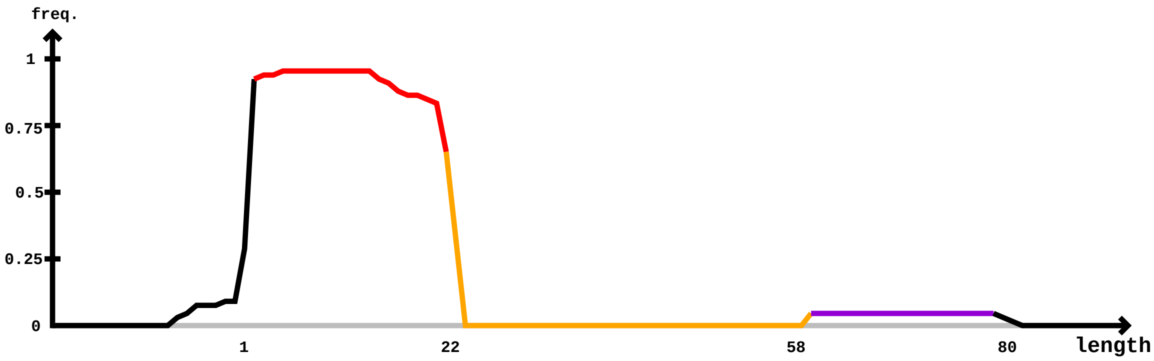

Mature   Star

```
5'- ccucugguuugacuuguccauagcaucauccauccugccauucugcugagguagcugcugcccuuuugcacgauggguagaauggcuggugcuauggaugaauucgacca  -3'   obs
    ccucugguuugacuuguccauagcaucauccauccugccauucugcugagguagcugcugcccuuuugcacgauggguagaauggcuggugcuauggaugaauucgacca         exp
    .....((((.((.(..((((((((((((.((((.((((((((.(((.((((..((....))))))..))).))).))))).)))).))))))))))))..).)).)))).   reads   mm   sample
    ..................cauagcaucauccauccugcc.......................................................................   7       0    NN8
    ..................cauagcaucauccauccugcca......................................................................   1       0    NN8
    ...................auagcaucauccauccugcca......................................................................   1       0    NN8
    ......................gcaucauccauccugcca......................................................................   1       0    NN8

    ...........acuuguccauagcaucaucca..............................................................................   2       0    FF1
    ............cuuguccauagcaucauccau.............................................................................   1       0    FF1
    .............uuguccauagcaucauccauc............................................................................   2       0    FF1
    ................uccauagcaucauccaucc...........................................................................   1       0    FF1
    ..................cauagcaucauccauccugcc.......................................................................   3       0    FF1
    ..................caAagcaucauccauccugcc.......................................................................   1       1    FF1
    ..................cauagcaucauccauGcugcc.......................................................................   1       1    FF1
    ...................auagcaucauccauccuC.........................................................................   1       1    FF1
    ...................aCagcaucauccauccugcca......................................................................   1       1    FF1
    ...................auagcaucauccauccugcca......................................................................   38      0    FF1
    ...................auagcaucauccCuccugcca......................................................................   1       1    FF1
    ....................uagcaucauccauccugc........................................................................   1       0    FF1
    .............................................................................uagaauggcuggugcuaugga............   2       0    FF1
    .............................................................................uagaauggcuggugcuauggUu...........   1       1    FF1
```
