# Supplementary material for: Identification of Novel miRNAs and miRNA Expression Profiling in Wheat Hybrid Necrosis
Source: PLoS One. 2015 Feb 23;10(2):e0117507. doi: 10.1371/journal.pone.0117507 (PMC4338152; doi:10.1371/journal.pone.0117507)
Supplement: S2 Fig — Red colored letter: mature miRNA sequence; yellow colored letter: loop sequence; blue colored letter: miRNA* sequence. (ZIP) [file pone.0117507.s002.zip › Figures s1/contig1984885_13090.pdf]

Provisional ID	:	contig1984885_13090
Score total	:	76.5
Score for star read(s)	:	3.9
Score for read counts	:	65.9
Score for mfe	:	2.1
Score for randfold	:	1.6
Score for cons. seed	:	3
Total read count	:	141
Mature read count	:	120
Loop read count	:	0
Star read count	:	21

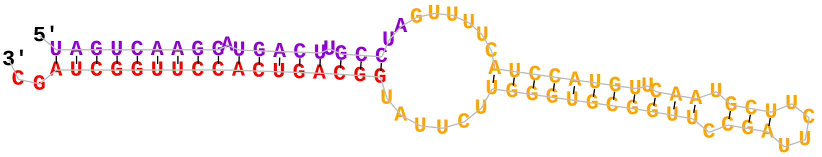

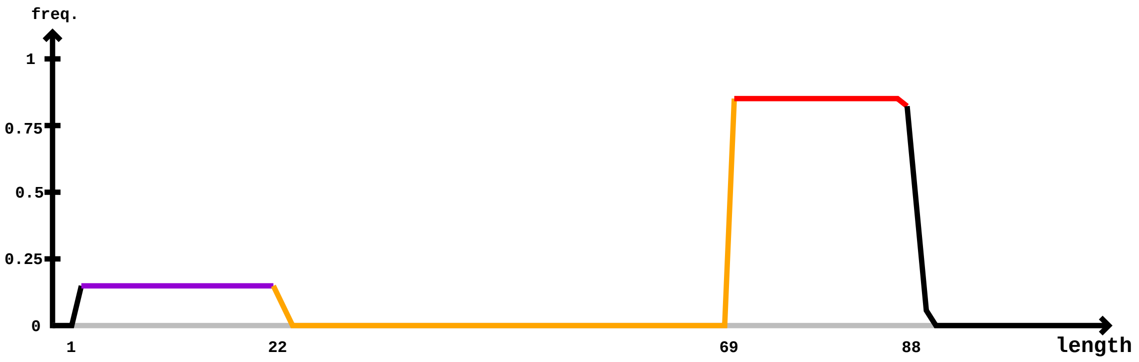

Star **Mature**

```
5'- auagucaaggaugacuugccuaguuuucauccauguucaaugcuucuuagccuuggcguggguucuuauggcagucaccuuggcuagccuggguggcucuugcugcuc   -3'   obs
    auagucaaggaugacuugccuaguuuucauccauguucaaugcuucuuagccuuggcguggguucuuauggcagucaccuuggcuagccuggguggcucuugcugcuc         exp
    .((((((((.(((((.(((.........((((((((.(((.(((....))).)))))))))))......)))))))))))))))))....(((..((....))..)))   reads   mm   sample
    .uagCcaaggaugacuugccua......................................................................................   16      1    NN8
    ....................................................................ggcagucUccuuggcuagc.....................   1       1    NN8

    .uagCcaaggaugacuugccua......................................................................................   5       1    FF1
    ....................................................................ggcagucUccuuggcuag......................   3       1    FF1
    ....................................................................ggcagucaccuuggcuag......................   1       0    FF1
    ....................................................................ggcagucUccuuggcuagc.....................   63      1    FF1
    ....................................................................ggcagucaccuuggcuagc.....................   17      0    FF1
    ....................................................................ggcagucGccuuggcuagc.....................   13      1    FF1
    ....................................................................ggcagucUccuuggcuagcc....................   13      1    FF1
    ....................................................................ggcagucGccuuggcuagcc....................   1       1    FF1
    ....................................................................ggcagucaccuuggcuagccu...................   1       0    FF1
    ....................................................................ggcagucUccuuggcuagccu...................   7       1    FF1
```
