# Supplementary material for: Identification of Novel miRNAs and miRNA Expression Profiling in Wheat Hybrid Necrosis
Source: PLoS One. 2015 Feb 23;10(2):e0117507. doi: 10.1371/journal.pone.0117507 (PMC4338152; doi:10.1371/journal.pone.0117507)
Supplement: S2 Fig — Red colored letter: mature miRNA sequence; yellow colored letter: loop sequence; blue colored letter: miRNA* sequence. (ZIP) [file pone.0117507.s002.zip › Figures s1/contig2110861_13447.pdf]

Provisional ID	:	contig2110861_13447
Score total	:	0
Score for star read(s)	:	-1.3
Score for read counts	:	0
Score for mfe	:	0.3
Score for randfold	:	1.6
Score for cons. seed	:	-0.6
Total read count	:	18
Mature read count	:	18
Loop read count	:	0
Star read count	:	0

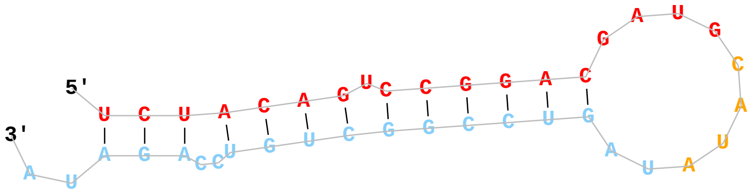

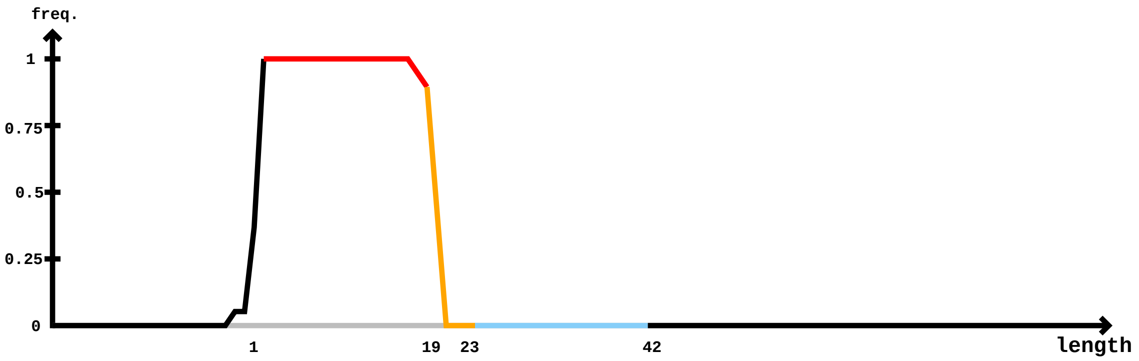

Mature **Star**

```
5'- gagucuuguaucuugacacuucuacaguccggacgaugcauauaguccggcuguccagauacccccuuauccaagacucccucaaauaccaugaguugaugcauuauu   -3'   exp
    ((((((((............(((((((.((((((..........))))))))))..)))............)))))))).((((.......))))............   reads   mm   sample
    ...................uucuacaguccggacgau.......................................................................   1       0    NN8
    ...................uucuacaguccggacgauC......................................................................   5       1    NN8
    ....................ucuacaguccggacgauC......................................................................   11      1    NN8

    .................aGuucuacaguccggacga........................................................................   1       1    FF1
    ....................ucuacaguccggacgauC......................................................................   1       1    FF1
```
