# Supplementary material for: Identification of Novel miRNAs and miRNA Expression Profiling in Wheat Hybrid Necrosis
Source: PLoS One. 2015 Feb 23;10(2):e0117507. doi: 10.1371/journal.pone.0117507 (PMC4338152; doi:10.1371/journal.pone.0117507)
Supplement: S2 Fig — Red colored letter: mature miRNA sequence; yellow colored letter: loop sequence; blue colored letter: miRNA* sequence. (ZIP) [file pone.0117507.s002.zip › Figures s1/contig2137453_13522.pdf]

Provisional ID	:	contig2137453_13522
Score total	:	3884.7
Score for star read(s)	:	3.9
Score for read counts	:	3873.8
Score for mfe	:	2.5
Score for randfold	:	1.6
Score for cons. seed	:	3
Total read count	:	7610
Mature read count	:	4810
Loop read count	:	0
Star read count	:	2800

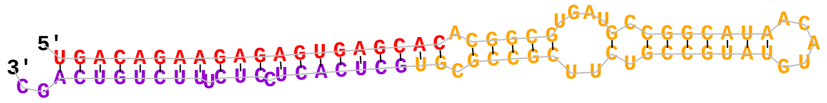

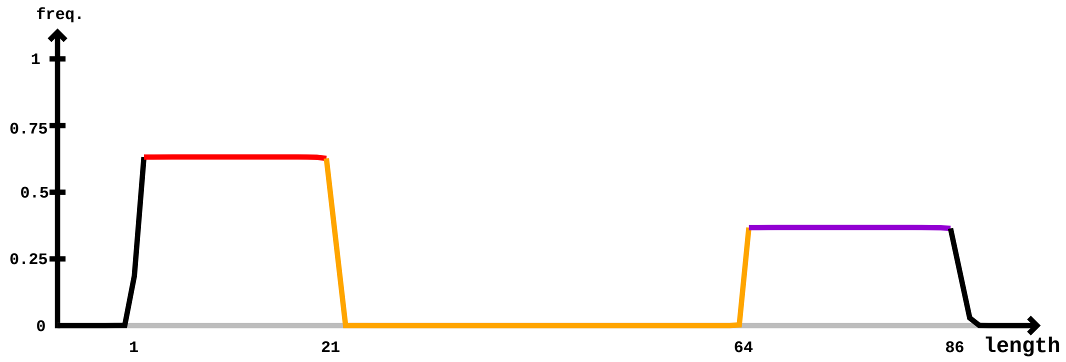

Mature **Star**

```
5'- gagagauugacagaagagagugagcacacggcgugaugccggcauaacauguaugccgucuucgccgcgugcucacuccucuuucugucagccucuuucu  -3'    obs
    gagagauugacagaagagagugagcacacggcgugaugccggcauaacauguaugccgucuucgccgcgugcucacuccucuuucugucagccucuuucu          exp
    (((((..((((((((((((((((((((.(((((....(.(((((((.....))))))).)..))))).))))))))).))).))))))))..)))))...    reads    mm    sample
    ......uugacagaagagagugaUcac.........................................................................    1        1     NN8
    ......uugacagaagagagugagcac.........................................................................    12       0     NN8
    ......uugacagaagagagugagcacU........................................................................    1        1     NN8
    .......ugacagaagagagugagc...........................................................................    1        0     NN8
    .......ugacagaagagagugagca..........................................................................    2        0     NN8
    .......ugacagaagagagugUgcac.........................................................................    1        1     NN8
    .......ugacagaagagagugagcac.........................................................................    135      0     NN8
    .......Agacagaagagagugagcac.........................................................................    1        1     NN8
    .......ugacagaagagGgugagcac.........................................................................    1        1     NN8
    .......ugacagaagagagugagcaU.........................................................................    1        1     NN8
    .......ugacagaagagagugagcacU........................................................................    11       1     NN8
    .........acagaagagagugagcac.........................................................................    1        0     NN8
    ......................................................................gcucacuccucuuucuguc...........    1        0     NN8
    ......................................................................gcucacuccucuuucuguca..........    3        0     NN8
    ......................................................................gcucacuccucuuucugucag.........    7        0     NN8
    ......................................................................gcucacuccucuuuGugucag.........    1        1     NN8
    ......................................................................gcCcacuccucuuucugucagc........    1        1     NN8
    ......................................................................gcucacuccuGuuucugucagc........    1        1     NN8
    ......................................................................gcucaUuccucuuucugucagc........    2        1     NN8
    ......................................................................gcucacuccucuCucugucagc........    3        1     NN8
    ......................................................................gcucacuccucuuucuguAagc........    1        1     NN8
    ......................................................................gcucacuccuAuuucugucagc........    2        1     NN8
    ......................................................................gcucacuccuUuuucugucagc........    1        1     NN8
    ......................................................................gcuGacuccucuuucugucagc........    1        1     NN8
    ......................................................................gcucUcuccucuuucugucagc........    2        1     NN8
    ......................................................................gcucacuccucuuucugucagG........    1        1     NN8
    ......................................................................gcucCcuccucuuucugucagc........    1        1     NN8
    ......................................................................gAucacuccucuuucugucagc........    1        1     NN8
    ......................................................................gcucacGccucuuucugucagc........    1        1     NN8
    ......................................................................gcucacuccucuuucuguUagc........    2        1     NN8
    ......................................................................gcucacuccucuuucAgucagc........    1        1     NN8
    ......................................................................gcucacuccucuuuAugucagc........    1        1     NN8
    ......................................................................gcucacuccucuuGcugucagc........    2        1     NN8
```

Mature **Star**

gagagauugacagaagagagugagcacacggcgugaugccggcauaaacauguaugccgucuucgccgcgugcucacuccucuuucugucagccucuuucu			
....gcucacuccucuuucuguGagc........	1	1	NN8
....gcucacuccucuAucugucagc........	1	1	NN8
....gcucacuccucuuucugucagc........	837	0	NN8
....gcucacuccucuuucugAcagcc.......	2	1	NN8
....gcucacuccucuuucuguAagcc.......	2	1	NN8
....gcucacuccucuuucugucUgcc.......	1	1	NN8
....gcucacuccucuuuGugucagcc.......	1	1	NN8
....gcucacuccucuuucugGcagcc.......	2	1	NN8
....gAucacuccucuuucugucagcc.......	1	1	NN8
....gcucacuccucuuucGgucagcc.......	1	1	NN8
....Acucacuccucuuucugucagcc.......	1	1	NN8
....gcucacuccucuuucugucagGc.......	1	1	NN8
....Ucucacuccucuuucugucagcc.......	1	1	NN8
....gcucacuccucuuucuguGagcc.......	2	1	NN8
....gcucacuccucuuucugucagUc.......	1	1	NN8
....gcucacuccucuuucugucagcU.......	2	1	NN8
....gcucacuccucuuucugucagcA.......	3	1	NN8
....gcucacuccuGuuucugucagcc.......	1	1	NN8
....gcucGcuccucuuucugucagcc.......	3	1	NN8
....gcucacuccuAuuucugucagcc.......	1	1	NN8
....gcucacuccucuuucugucagcc.......	589	0	NN8
....gcucacuccucGuucugucagcc.......	1	1	NN8
....gcucacuccucuuucugCcagcc.......	1	1	NN8
....gcucacuccGcuuucugucagcc.......	1	1	NN8
....gcucacuccucuuucAgucagcc.......	1	1	NN8
....gcucacucUucuuucugucagcc.......	1	1	NN8
....gcucacuccucuuucuguUagcc.......	1	1	NN8
....gGucacuccucuuucugucagccu......	1	1	NN8
....gcucacuccucuuuUugucagccu......	1	1	NN8
....gcucaGuccucuuucugucagccu......	1	1	NN8
....gcucacuccucuuucugucagccu......	89	0	NN8
....gcucacuccucuuucugucagccC......	1	1	NN8
....gcucacuccucuuucAgucagccu......	1	1	NN8
....gcucacuccucuuucugucagccA......	8	1	NN8
....gcucacuccucuuucugucagccuc.....	2	0	NN8
....cacuccucuuucugucagc........	1	0	NN8
....gauugacagaagagagugagcac..	2	0	FF1
.....auugacagaagagagugagcac..	1	0	FF1
......uugacagaagagagugag...	2	0	FF1
......uugacagaagagagugagc..	2	0	FF1
......uugacagaagagGgugagca...	1	1	FF1
......uugacagaagagagugagca...	5	0	FF1
......uugGcagaagagagugagcac..	1	1	FF1
......uugacaAaagagagugagcac..	2	1	FF1
......uuAacagaagagagugagcac..	1	1	FF1
......uugacagaagagagugagcaU..	3	1	FF1
......uugacagaagagagugagGac..	1	1	FF1
......uugacagaagagaguUagcac..	1	1	FF1
......uugacagaagagagugagAac..	2	1	FF1
......uugacagaagagaguAagcac..	3	1	FF1
......uugacagaagagagCgagcac..	3	1	FF1
......uugacagaagagagugagcac..	1307	0	FF1
......uugacUgaagagagugagcac..	1	1	FF1
......uugaGagaagagagugagcac..	1	1	FF1
......uugacagaagagagugagcaA..	1	1	FF1
......Augacagaagagagugagcac..	2	1	FF1
......uugaUagaagagagugagcac..	3	1	FF1
......uugacagaagagGgugagcac..	2	1	FF1
......uugacagaagagagAgagcac..	1	1	FF1
......uugacagaagagagugGgcac..	1	1	FF1
......uugacagaCgagagugagcac..	1	1	FF1
......uugacagaagagagugaUcac..	1	1	FF1
......uugCcagaagagagugagcac..	1	1	FF1
......uugacagaagagUgugagcac..	4	1	FF1
......uugacagaagagagugaAcac..	4	1	FF1
......uugacagaagagagugaCcac..	2	1	FF1
......uugacagaagagagGgagcac..	12	1	FF1
......uugacagaagagCgugagcac..	3	1	FF1
......uuCacagaagagagugagcac..	1	1	FF1

Mature **Star**

gagagauugacagaagagagugagcacacggcgugaugccggcauaaacauguaugccgucuucgccgcgugcucacuccucuuucugucagccucuuucu			
......uugacagaagagagugagcacU...	20	1	FF1
......uugacagaagagagugagcaca...	3	0	FF1
.......ugacagaagagagugagc..	3	0	FF1
.......ugacagaagagaCugagca...	1	1	FF1
.......Ggacagaagagagugagca...	1	1	FF1
.......ugacagaagagagugagca...	22	0	FF1
.......ugCcagaagagagugagca...	1	1	FF1
.......ugGcagaagagagugagcac..	2	1	FF1
.......ugacagaagagagugagcCc..	2	1	FF1
.......ugacagaagagagugaAcac..	1	1	FF1
.......uAacagaagagagugagcac..	1	1	FF1
.......ugacagaGgagagugagcac..	1	1	FF1
.......ugacagaagaAagugagcac..	1	1	FF1
.......ugaGagaagagagugagcac..	1	1	FF1
.......ugacagaagagagugaCcac..	1	1	FF1
.......Agacagaagagagugagcac..	1	1	FF1
.......ugacagaagagGgugagcac..	2	1	FF1
.......ugacagaagagagGgagcac..	8	1	FF1
.......ugacagaagagagugCgcac..	2	1	FF1
.......ugacagaagagagugGgcac..	11	1	FF1
.......ugacagaagagagugagcGc..	1	1	FF1
.......ugacagaagagaCugagcac..	2	1	FF1
.......ugacagaagagagugagcac..	2949	0	FF1
.......ugacagCagagagugagcac..	1	1	FF1
.......ugacagaagagaAugagcac..	3	1	FF1
.......ugacagaagagagugagAac..	5	1	FF1
.......ugacagaaCagagugagcac..	1	1	FF1
.......ugacagaagagCgugagcac..	1	1	FF1
.......ugacagaaAagagugagcac..	3	1	FF1
.......ugacagaagagagugaUcac..	1	1	FF1
.......ugacagGagagagugagcac..	2	1	FF1
.......ugacagaagagagAgagcac..	2	1	FF1
.......ugacagaagagagugagcaA..	2	1	FF1
.......ugacagaagagagugagGac..	6	1	FF1
.......ugacagaagagaUugagcac..	7	1	FF1
.......ugacagaagagagugagcaU..	11	1	FF1
.......Ggacagaagagagugagcac..	3	1	FF1
.......uUacagaagagagugagcac..	2	1	FF1
.......ugaAagaagagagugagcac..	4	1	FF1
.......ugaUagaagagagugagcac..	1	1	FF1
.......ugacagaagagagugagcacU...	151	1	FF1
.......ugacagaagagagugagcaca...	21	0	FF1
.........acagaagagagugagcacacgU..	1	1	FF1
..........cagaagagagugagcaca...	1	0	FF1
....ugcucacuccucuuucuguca..........	1	0	FF1
....ugcucacuccucuuucugucag.........	5	0	FF1
....ugcucacuccucuuucugucagc........	12	0	FF1
....ugcucacuccucuuucuguAagc........	1	1	FF1
....gcucacuccucuuucugu............	1	0	FF1
....gcucacuccucuuucuguc...........	2	0	FF1
....gcucacuccucuuucugucag.........	6	0	FF1
....gcucacuccucuuucuguGagc........	1	1	FF1
....gcucacuccucAuucugucagc........	1	1	FF1
....gcucacuccucuuucuguUagc........	1	1	FF1
....gcucacuccucuuucugCcagc........	1	1	FF1
....gcucacuccucuAucugucagc........	1	1	FF1
....gcucacuccucuCucugucagc........	2	1	FF1
....gcucacuccucuuucGgucagc........	2	1	FF1
....gUucacuccucuuucugucagc........	1	1	FF1
....gcucGcuccucuuucugucagc........	1	1	FF1
....gGucacuccucuuucugucagc........	1	1	FF1
....gcucacuccucuGucugucagc........	1	1	FF1
....gcucacuccucuuucAgucagc........	1	1	FF1
....gcucacGccucuuucugucagc........	2	1	FF1
....gcucacuccucuuucuCucagc........	1	1	FF1
....gcucacucGucuuucugucagc........	1	1	FF1
....gcucacAccucuuucugucagc........	1	1	FF1
....gcucacuccucuuucCgucagc........	1	1	FF1
....gcucacuccucuuucugucagU........	1	1	FF1
....gcucacuccucuuucugucagc........	574	0	FF1

Mature Star

```
gagagauugacagaagagagugagcacacggcgugaugccggcauaacauguaugccgucuucgccgcgugcucacuccucuuucugucagccucuuucu
......................................................................gcucacuccucuuucugGcagc........   3     1     FF1
......................................................................gcucacuAcucuuucugucagc........   1     1     FF1
......................................................................gcucacucAucuuucugucagc........   2     1     FF1
......................................................................gcuAacuccucuuucugucagc........   1     1     FF1
......................................................................gcucacuccucGuucugucagc........   1     1     FF1
......................................................................gcucacuccucuuucAgucagcc.......   1     1     FF1
......................................................................gcucacucGucuuucugucagcc.......   1     1     FF1
......................................................................gcucacuccucuuucugGcagcc.......   1     1     FF1
......................................................................gcucUcuccucuuucugucagcc.......   1     1     FF1
......................................................................gcucacucAucuuucugucagcc.......   1     1     FF1
......................................................................gGucacuccucuuucugucagcc.......   1     1     FF1
......................................................................gcucacuccucuCucugucagcc.......   2     1     FF1
......................................................................gcucacuccucuuucugucagcc.......   441   0     FF1
......................................................................gcucacAccucuuucugucagcc.......   1     1     FF1
......................................................................gcuGacuccucuuucugucagcc.......   1     1     FF1
......................................................................gcucacuccucuuucugCcagcc.......   3     1     FF1
......................................................................gcucUcuccucuuucugucagccu......   1     1     FF1
......................................................................gcucacuccucuuucugucagccA......   1     1     FF1
......................................................................gcucacuccucuuucugucagccC......   5     1     FF1
......................................................................gcucacuccucGuucugucagccu......   1     1     FF1
......................................................................gcucacuccucuuucugucagccG......   1     1     FF1
......................................................................gcucacuccucuuucuCucagccu......   1     1     FF1
......................................................................gcucacuccucuuucugucagccu......   100   0     FF1
......................................................................gcucacuccucuuucugGcagccu......   1     1     FF1
......................................................................gcucacuccucuuucugucagccuc.....   1     0     FF1
........................................................................ucacuccucuuucugucagccu......   1     0     FF1
........................................................................uAacuccucuuucugucagccu......   1     1     FF1
```
