# Supplementary material for: Identification of Novel miRNAs and miRNA Expression Profiling in Wheat Hybrid Necrosis
Source: PLoS One. 2015 Feb 23;10(2):e0117507. doi: 10.1371/journal.pone.0117507 (PMC4338152; doi:10.1371/journal.pone.0117507)
Supplement: S2 Fig — Red colored letter: mature miRNA sequence; yellow colored letter: loop sequence; blue colored letter: miRNA* sequence. (ZIP) [file pone.0117507.s002.zip › Figures s1/contig219800_4299.pdf]

Provisional ID	:	contig219800_4299
Score total	:	925.1
Score for star read(s)	:	3.9
Score for read counts	:	917.8
Score for mfe	:	2.4
Score for randfold	:	1.6
Score for cons. seed	:	-0.6
Total read count	:	1812
Mature read count	:	1509
Loop read count	:	0
Star read count	:	303

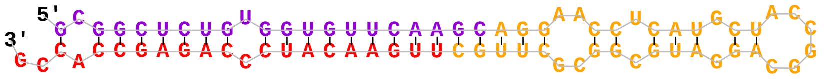

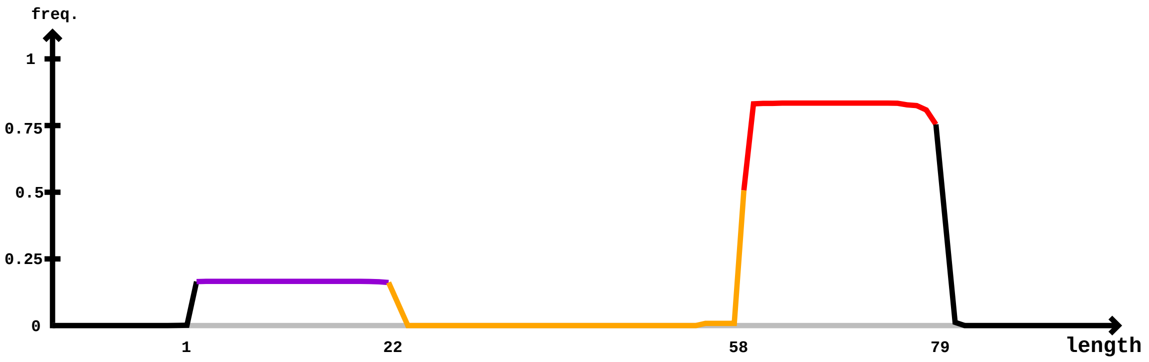

Star **Mature**

```
5'- gucggggagcccggcggcucugugguguucaagcaggaaccucaugcuaccggcaggaugcggcgcuugcuugaacaucccagagccaccggcgugccaaaauucacgc  -3'  obs
    gucggggagcccggcggcucugugguguucaagcaggaaccucaugcuaccggcaggaugcggcgcuugcuugaacaucccagagccaccggcgugccaaaauucacgc        exp
    .....((.((((((.(((((((.((((((((((((((..((.(((.((......)).))).))..))))))))))))))).))))))).)))).)).))...........  reads  mm  sample
    ......................................................................uugaacaucccagagccac....................  1      0   NN8
    ......................................................................uugaacaucccUgagccacc...................  1      1   NN8
    ......................................................................uugaacaucccagagccacc...................  21     0   NN8
    ......................................................................uugaacaucccaUagccaccg..................  1      1   NN8
    ......................................................................uugaacaucccGgagccaccg..................  1      1   NN8
    ......................................................................Gugaacaucccagagccaccg..................  2      1   NN8
    ......................................................................uugaacaucccagagccaccg..................  115    0   NN8
    ......................................................................uugaacaucAcagagccaccg..................  1      1   NN8
    .......................................................................ugaacaucccagagccac....................  2      0   NN8
    .......................................................................ugaacaucccagagccacc...................  4      0   NN8
    .......................................................................ugaacaucccagagccaccg..................  3      0   NN8
    .......................................................................ugaacaucccagagccaccgg.................  13     0   NN8
    .......................................................................ugaacaucccaCagccaccgg.................  1      1   NN8
    .......................................................................ugaacaucccagagccaccgU.................  1      1   NN8
    .......................................................................ugaacaucccaAagccaccggc................  1      1   NN8
    ........................................................................gaacaucccagagccaccggc................  1      0   NN8
    ..........................................................................acaucccagagccaccgg.................  1      0   NN8

    ...........cggcggcucugugguguucaa.............................................................................  1      0   FF1
    ............ggcggcucugugguguucaag............................................................................  1      0   FF1
    .............gcggcucugugguguuca..............................................................................  1      0   FF1
    .............gcggcucugugguguucaa.............................................................................  1      0   FF1
    .............gcggcucugugguguucaag............................................................................  4      0   FF1
    .............gcggcucuguggCguucaagc...........................................................................  2      1   FF1
    .............gAggcucugugguguucaagc...........................................................................  1      1   FF1
    .............gcggcucuguAguguucaagc...........................................................................  1      1   FF1
    .............gcggcucugugguguucaagc...........................................................................  275    0   FF1
    .............gcggcucuguggAguucaagc...........................................................................  4      1   FF1
    .............gUggcucugugguguucaagc...........................................................................  1      1   FF1
    .............gcggcucuguggugugcaagc...........................................................................  2      1   FF1
    .............gcggcucugGgguguucaagc...........................................................................  1      1   FF1
    .............gcggcucuguggGguucaagc...........................................................................  3      1   FF1
    .............gcggcucugugguggucaagc...........................................................................  2      1   FF1
```

```
             Star                                                Mature
gucggggagcccggcggcucugugguguucaagcaggaaccucaugcuaccggcaggaugcggcgcuugcuugaacaucccagagccaccggcgugccaaaauucacgc
.............gcggcucugugguguucaagcU..........................................................................  1    1  FF1
..............cggcucugugguguucaagc...........................................................................  2    0  FF1
..................................................................uugcuugaacaucccagagc.......................  1    0  FF1
..................................................................uugcuugaacaucccagagcc......................  11   0  FF1
..................................................................uugcuugaacaucccagagccU.....................  3    1  FF1
......................................................................uugaacaucccagagccU.....................  2    1  FF1
......................................................................uugaacaucccagagccac....................  10   0  FF1
......................................................................uugaacaucccagagccaU....................  1    1  FF1
......................................................................uugaacaGcccagagccacc...................  1    1  FF1
......................................................................uugaacaucccagagccacc...................  39   0  FF1
......................................................................uugaacaucccaAagccacc...................  1    1  FF1
......................................................................uugaacaucccagagccaccC..................  4    1  FF1
......................................................................uugaacaucccaCagccaccg..................  2    1  FF1
......................................................................uugaacauGccagagccaccg..................  2    1  FF1
......................................................................uuUaacaucccagagccaccg..................  1    1  FF1
......................................................................uugUacaucccagagccaccg..................  1    1  FF1
......................................................................uugaacaucccagagUcaccg..................  1    1  FF1
......................................................................Gugaacaucccagagccaccg..................  1    1  FF1
......................................................................uugaacaucccagagccaccU..................  15   1  FF1
......................................................................uugaacaucccagagGcaccg..................  2    1  FF1
......................................................................uGgaacaucccagagccaccg..................  1    1  FF1
......................................................................Augaacaucccagagccaccg..................  1    1  FF1
......................................................................uugaacauAccagagccaccg..................  1    1  FF1
......................................................................uugaacaucccagagcAaccg..................  1    1  FF1
......................................................................uugaacaucccagagccaccg..................  675  0  FF1
......................................................................uugaacauUccagagccaccg..................  1    1  FF1
......................................................................uugaacaucccGgagccaccg..................  1    1  FF1
......................................................................uugaacaucccagagccaccgU.................  4    1  FF1
......................................................................uugaacaucccagagccaccgC.................  1    1  FF1
.......................................................................ugaacaucccagagccac....................  13   0  FF1
.......................................................................Ggaacaucccagagccac....................  1    1  FF1
.......................................................................ugaacaucccagagccaU....................  2    1  FF1
.......................................................................ugaacaucccagagccacc...................  28   0  FF1
.......................................................................ugaacaucccagagccaUc...................  2    1  FF1
.......................................................................Ggaacaucccagagccacc...................  1    1  FF1
.......................................................................ugaacaucccagagccacU...................  1    1  FF1
.......................................................................ugaacaucccagagccaccg..................  15   0  FF1
.......................................................................ugaacaucccagagccaccU..................  5    1  FF1
.......................................................................ugaacaucccagUgccaccgg.................  1    1  FF1
.......................................................................uCaacaucccagagccaccgg.................  1    1  FF1
.......................................................................ugaacaucccagagcGaccgg.................  1    1  FF1
.......................................................................ugaacaucccagGgccaccgg.................  2    1  FF1
.......................................................................ugaacaucccagagccCccgg.................  1    1  FF1
.......................................................................ugaacaucccagagccacAgg.................  1    1  FF1
.......................................................................Ggaacaucccagagccaccgg.................  2    1  FF1
.......................................................................ugaacaucccagagccaccgA.................  2    1  FF1
.......................................................................ugaacaucccUgagccaccgg.................  2    1  FF1
.......................................................................ugaGcaucccagagccaccgg.................  1    1  FF1
.......................................................................ugaacaucccaUagccaccgg.................  1    1  FF1
.......................................................................ugaacaucccagagccaAcgg.................  1    1  FF1
.......................................................................ugaacaucccagCgccaccgg.................  1    1  FF1
.......................................................................ugaacaucccagagccaccgU.................  5    1  FF1
.......................................................................ugaacauGccagagccaccgg.................  1    1  FF1
.......................................................................ugaacaucccagagccaccgg.................  456  0  FF1
.......................................................................Cgaacaucccagagccaccgg.................  2    1  FF1
.......................................................................ugaacaucccagagccaccggc................  1    0  FF1
.......................................................................ugaacaucccagagccaccggA................  12   1  FF1
.......................................................................ugaacaucccagagccaccgUc................  1    1  FF1
.......................................................................ugaacaucccagagccaccggU................  4    1  FF1
........................................................................gaacaucccagagccaccggc................  2    0  FF1
..........................................................................acaucccagagccaccgg.................  1    0  FF1
```
