# Supplementary material for: Identification of Novel miRNAs and miRNA Expression Profiling in Wheat Hybrid Necrosis
Source: PLoS One. 2015 Feb 23;10(2):e0117507. doi: 10.1371/journal.pone.0117507 (PMC4338152; doi:10.1371/journal.pone.0117507)
Supplement: S2 Fig — Red colored letter: mature miRNA sequence; yellow colored letter: loop sequence; blue colored letter: miRNA* sequence. (ZIP) [file pone.0117507.s002.zip › Figures s1/contig2297694_13883.pdf]

Provisional ID	:	contig2297694_13883
Score total	:	2.3
Score for star read(s)	:	-1.3
Score for read counts	:	0
Score for mfe	:	2.6
Score for randfold	:	1.6
Score for cons. seed	:	-0.6
Total read count	:	342
Mature read count	:	333
Loop read count	:	0
Star read count	:	9

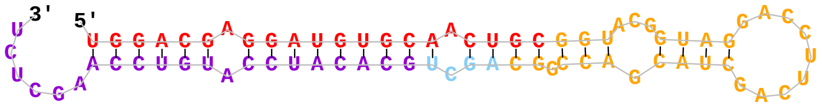

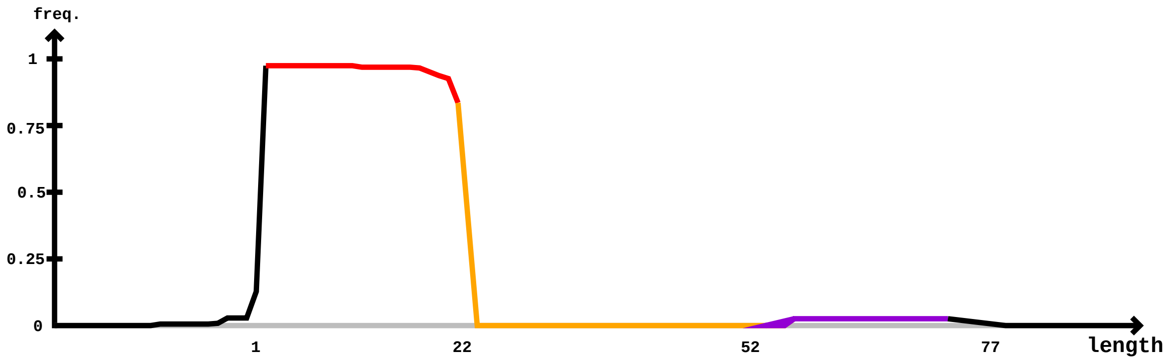

Mature **Star**

5'-	guacgauccuagcuagagcuuggacgaggaugugcaacugcgguacgguaggaccuucagcuacgaccggcagcugcacauccauguccaagcucuagcuacgaccggcag	-3'	obs		
	guacgauccuagcuagagcuuggacgaggaugugcaacugcgguacgguaggaccuucagcuacgaccggcagcugcacauccauguccaagcucuagcuacgaccggcag		exp		
	((.((.((.(((((((((((((((((.(((((((((.(((((((...((((.........)))).))).)))).))))))))).))))))))))))))))).)).))))..		reads	mm	sample
	....................uggacgaggaugugcaacugc..		4	0	NN8
	....gcacauccaugCccaagcucu...............		5	1	NN8
	.........uagcuagagcuuggacgagga...		2	0	FF1
	...............gagcuuggacgaggaugugca...		1	0	FF1
	................agcuuggacgaggaugugcaa..		4	0	FF1
	................agcuuggacgaggaugugcaac...		1	0	FF1
	................agcuuggacgaggaugugcaacu..		2	0	FF1
	...................uuggacgaggaugugcaa..		1	0	FF1
	...................uuggacgaggaugugcaac...		1	0	FF1
	...................uuggacgaggaugugcaacu..		2	0	FF1
	...................uuggacgaAgaugugcaacug...		1	1	FF1
	...................uuggacgaggaugugcaacug...		28	0	FF1
	...................uuggacgaggaugugcaacugA..		1	1	FF1
	...................uuggacgaggaugugcaacugc..		1	0	FF1
	....................uggacgaggaugugcaac...		3	0	FF1
	....................uggacgaggaugugcaacug...		3	0	FF1
	....................uggacgaggaugGgcaacugc..		1	1	FF1
	....................uggacgaggaugCgcaacugc..		1	1	FF1
	....................uggacgaggaugugcaCcugc..		2	1	FF1
	....................uggacgaggaugugcaacugc..		281	0	FF1
	....................uggacgaggaugugcaaUugc..		1	1	FF1
	....................Gggacgaggaugugcaacugc..		1	1	FF1
	....................uggacgGggaugugcaacugc..		1	1	FF1
	....gcacauccaugCccaagcucu...............		4	1	FF1
