# Supplementary material for: Identification of Novel miRNAs and miRNA Expression Profiling in Wheat Hybrid Necrosis
Source: PLoS One. 2015 Feb 23;10(2):e0117507. doi: 10.1371/journal.pone.0117507 (PMC4338152; doi:10.1371/journal.pone.0117507)
Supplement: S2 Fig — Red colored letter: mature miRNA sequence; yellow colored letter: loop sequence; blue colored letter: miRNA* sequence. (ZIP) [file pone.0117507.s002.zip › Figures s1/contig24155_977.pdf]

Provisional ID	:	contig24155_977
Score total	:	0.4
Score for star read(s)	:	-1.3
Score for read counts	:	0
Score for mfe	:	0.9
Score for randfold	:	-2.2
Score for cons. seed	:	3
Total read count	:	138
Mature read count	:	138
Loop read count	:	0
Star read count	:	0

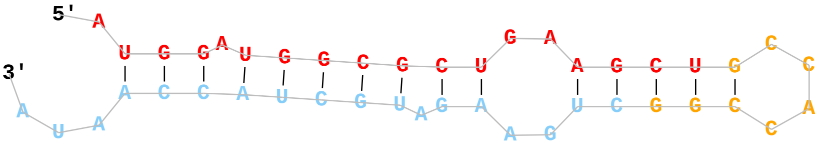

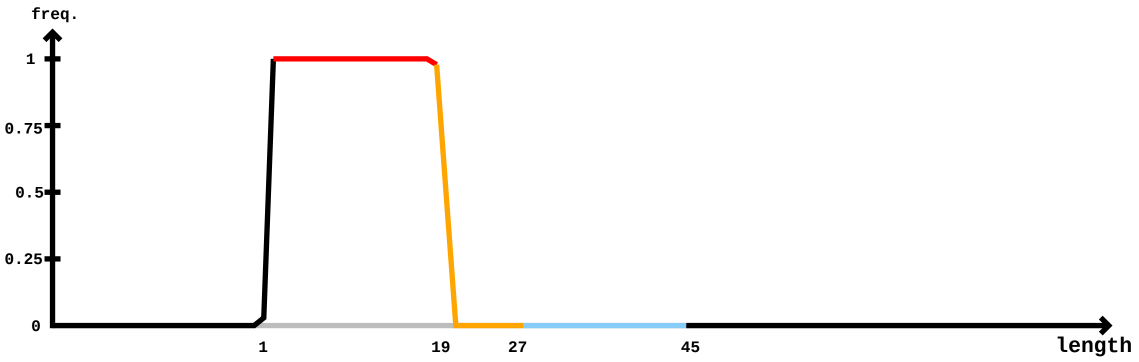

Mature Star

```
5'- ucuacugacguuguugcacugauggauggcgcugaagcugccaccggcugaagaugcuaccaauaagucugucgggccaucgagagacaucacacaacacaaucacau   -3'   exp
    .....(((...(((((......(((.(((((((..(((((....)))))..)).))))))))....((((.((((....)))).))))......)))))...)))...   reads   mm   sample
    ....................gauggauggcgcugaagc......................................................................   3       0    NN8
    ....................gauggauggcgcugaagcG.....................................................................   1       1    NN8
    .....................auggauggcgcugaagcG.....................................................................   119     1    NN8

    .....................auggauggcgcugaagcG.....................................................................   15      1    FF1
```
