# Supplementary material for: Identification of Novel miRNAs and miRNA Expression Profiling in Wheat Hybrid Necrosis
Source: PLoS One. 2015 Feb 23;10(2):e0117507. doi: 10.1371/journal.pone.0117507 (PMC4338152; doi:10.1371/journal.pone.0117507)
Supplement: S2 Fig — Red colored letter: mature miRNA sequence; yellow colored letter: loop sequence; blue colored letter: miRNA* sequence. (ZIP) [file pone.0117507.s002.zip › Figures s1/contig244456_4590.pdf]

Provisional ID	:	contig244456_4590
Score total	:	1.5
Score for star read(s)	:	-1.3
Score for read counts	:	0
Score for mfe	:	1.8
Score for randfold	:	1.6
Score for cons. seed	:	-0.6
Total read count	:	12
Mature read count	:	12
Loop read count	:	0
Star read count	:	0

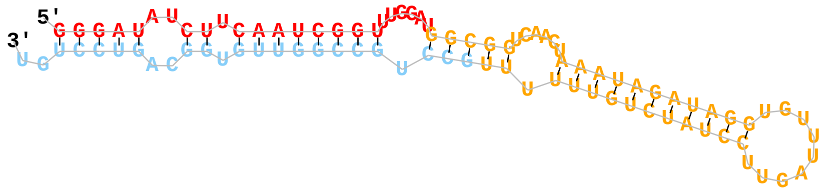

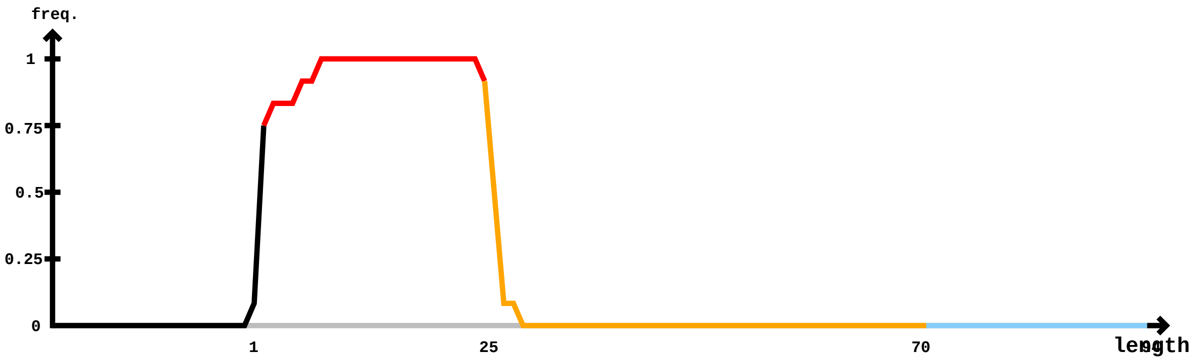

Mature Star

```
5'- ccguugagauacgguagcacgggauaucuucaaucgguuuggauggcggucaacuaaauagauagguguuuaguuccuaucuguuuuuugccugccgguuguggcaguccuguu  -3'   exp
    ((((......))))....(((((((..((.((((((((......(((((......(((((((((((.........))))))))))).))))).)))))))).))..))))))).   reads   mm   sample
    ....................ggAauaucuucaaucgguuuggau......................................................................   1       1    NN8
    ..........................uAuucaaucgguuuggau......................................................................   1       1    NN8

    ...................cgggauaucuucaaCcgguuugga.......................................................................   1       1    FF1
    ....................gggauauAuucaaucgguuuggau......................................................................   1       1    FF1
    ....................gggauaucuucaaCcgguuuggau......................................................................   2       1    FF1
    ....................gggauaucuucaaucgguuuggau......................................................................   4       0    FF1
    .....................ggauaucuucaaucgguuuggau......................................................................   1       0    FF1
    ........................uaucuucaaCcgguuuggauggc...................................................................   1       1    FF1
```
