# Supplementary material for: Identification of Novel miRNAs and miRNA Expression Profiling in Wheat Hybrid Necrosis
Source: PLoS One. 2015 Feb 23;10(2):e0117507. doi: 10.1371/journal.pone.0117507 (PMC4338152; doi:10.1371/journal.pone.0117507)
Supplement: S2 Fig — Red colored letter: mature miRNA sequence; yellow colored letter: loop sequence; blue colored letter: miRNA* sequence. (ZIP) [file pone.0117507.s002.zip › Figures s1/contig2460659_14297.pdf]

Provisional ID : contig2460659_14297
Score total : 2.7
Score for star read(s) : -1.3
Score for read counts : 0
Score for mfe : 3
Score for randfold : 1.6
Score for cons. seed : -0.6
Total read count : 116
Mature read count : 116
Loop read count : 0
Star read count : 0

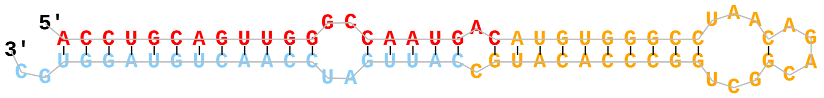


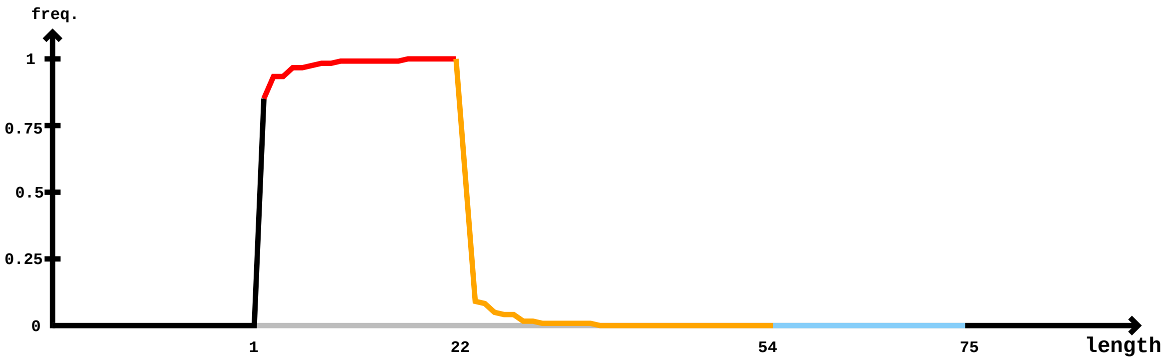


Mature Star

| 5'- aacuucagugccguaacuguaccugcaguugggccaaugacaugugggccuaacagacggcuggcccacaugccauugauccaacuguaggugcaguuacggcacccaagc -3' | exp | | |
|---|---|---|---|
| ..(((..(((((((((((((((((((((((((..(((((.((((((((((...(....)...)))))))))).)))))..)))))))))))))))))))))))))..))). | reads | mm | sample |
| ....................accGgcaguugggccaaugac...................................................................... | 1 | 1 | NN8 |
| ....................Gccugcaguugggccaaugac...................................................................... | 1 | 1 | NN8 |
| ....................accugcaguugggccaaugac...................................................................... | 52 | 0 | NN8 |
| ....................accugcagGugggccaaugac...................................................................... | 1 | 1 | NN8 |
| ....................accugcaguugggccaaugacaug................................................................... | 1 | 0 | NN8 |
| .....................ccugcaguugggccaaugac...................................................................... | 1 | 0 | NN8 |
| .....................ccugcaguugggccaaugaca..................................................................... | 1 | 0 | NN8 |
| .......................ugcaguugggccaaugacaugugg................................................................ | 2 | 0 | NN8 |
| ...................................aaugacaugugggccuaaca........................................................ | 1 | 0 | NN8 |
| | | | |
| ....................accugcaguugggccaaGgac...................................................................... | 1 | 1 | FF1 |
| ....................accugcaguugggccaaugac...................................................................... | 41 | 0 | FF1 |
| ....................accugcaguugggccaaugaca..................................................................... | 3 | 0 | FF1 |
| ....................accugcaguugggccaaugacau.................................................................... | 1 | 0 | FF1 |
| ....................accugcaguugggccaaugGcaug................................................................... | 1 | 1 | FF1 |
| .....................ccugcaguugggccaaugaca..................................................................... | 8 | 0 | FF1 |
| .......................ugcaguugggccaaugGcaug................................................................... | 2 | 1 | FF1 |
| .........................caguugggccaaugacaugu.................................................................. | 1 | 0 | FF1 |
| ..........................aguugggccaaugacaugugg................................................................ | 1 | 0 | FF1 |
| ............................uugggccaaugacaugugggc.............................................................. | 1 | 0 | FF1 |
