# Supplementary material for: Identification of Novel miRNAs and miRNA Expression Profiling in Wheat Hybrid Necrosis
Source: PLoS One. 2015 Feb 23;10(2):e0117507. doi: 10.1371/journal.pone.0117507 (PMC4338152; doi:10.1371/journal.pone.0117507)
Supplement: S2 Fig — Red colored letter: mature miRNA sequence; yellow colored letter: loop sequence; blue colored letter: miRNA* sequence. (ZIP) [file pone.0117507.s002.zip › Figures s1/contig2515702_14400.pdf]

Provisional ID	:	contig2515702_14400
Score total	:	0.4
Score for star read(s)	:	-1.3
Score for read counts	:	0
Score for mfe	:	0.9
Score for randfold	:	-2.2
Score for cons. seed	:	3
Total read count	:	150
Mature read count	:	149
Loop read count	:	0
Star read count	:	1

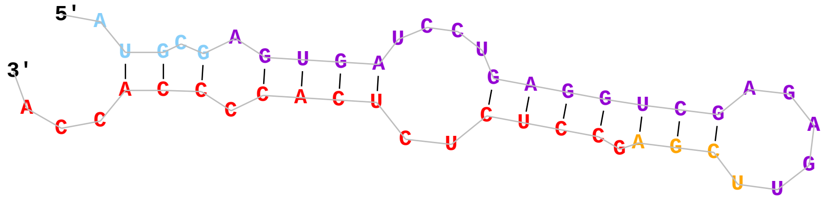

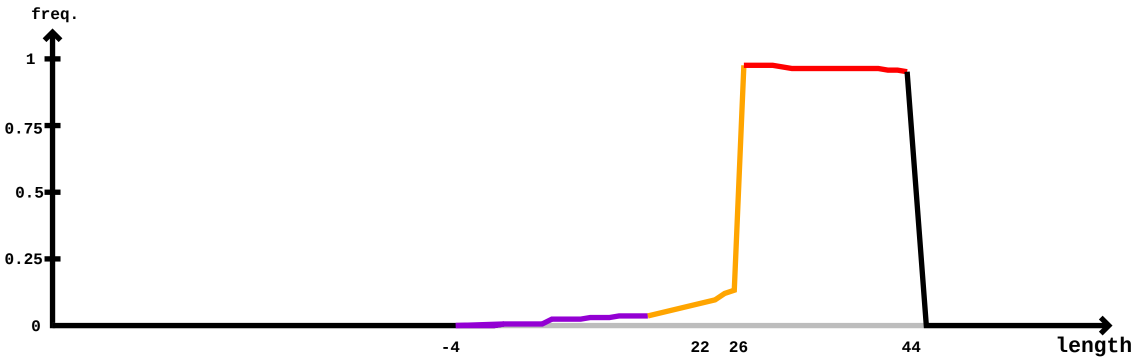


```
                                                 Star                 Mature
5'- gugguggcgcaguuggcuagcgcguaggucucauaacugaaugcgagugauccugaggucgagaguucgagccucucucaccccaccauuuauaagaccuuguaguga   -3'   obs
    gugguggcgcaguuggcuagcgcguaggucucauaacugaaugcgagugauccugaggucgagaguucgagccucucucaccccaccauuuauaagaccuuguaguga         exp
    (((.((((.......)))).)))..((((((.((((.((..((.(.((((....(((((((......))).))))..)))).))).)).)))).))))))........    reads      mm       sample
    ..................................................uccCgaggucgagaguucg.......................................    1          1        NN8
    ..................................................uccugaggGcgagaguucg.......................................    1          1        NN8
    .........................................................gucgagaguucgagAcuc.................................    1          1        NN8
    ................................................................guucgUgccucucucaccccacca....................    2          1        NN8
    .................................................................uucgUgccucucucaccccacca....................    2          1        NN8
    ..................................................................ucgUgccucucucaccccacc.....................    1          1        NN8
    ...................................................................cgUgccucucucaccccacca....................    2          1        NN8
    ....................................................................gUgccucucucaccccacca....................    1          1        NN8
    ....................................................................gagccucucucaccccacca....................    2          0        NN8
    .....................................................................agccucucucaccccacca....................    2          0        NN8
    .....................................................................Ugccucucucaccccacca....................    2          1        NN8
    ......................................................................gcAucucucaccccacca....................    2          1        NN8
    ......................................................................gccucucucaccccacca....................    90         0        NN8
    ......................................................................gccucucucaccUcacca....................    1          1        NN8

    .............................................agugauccugaggucAagagu..........................................    1          1        FF1
    ..................................................uAcugaggucgagaguucg.......................................    1          1        FF1
    ......................................................gaggucgagaguucgGgccu..................................    1          1        FF1
    .................................................................uucgUgccucucucaccccacca....................    1          1        FF1
    ...................................................................cgUgccucucucacccca.......................    1          1        FF1
    ...................................................................cgGgccucucucaccccacca....................    1          1        FF1
    ...................................................................cgUgccucucucaccccacca....................    1          1        FF1
    ....................................................................gagccucucucaccccacca....................    1          0        FF1
    .....................................................................Cgccucucucaccccacca....................    1          1        FF1
    ......................................................................gccucucucaccccacca....................    47         0        FF1
```
