# Supplementary material for: Identification of Novel miRNAs and miRNA Expression Profiling in Wheat Hybrid Necrosis
Source: PLoS One. 2015 Feb 23;10(2):e0117507. doi: 10.1371/journal.pone.0117507 (PMC4338152; doi:10.1371/journal.pone.0117507)
Supplement: S2 Fig — Red colored letter: mature miRNA sequence; yellow colored letter: loop sequence; blue colored letter: miRNA* sequence. (ZIP) [file pone.0117507.s002.zip › Figures s1/contig253044_4695.pdf]

Provisional ID	:	contig253044_4695
Score total	:	66.6
Score for star read(s)	:	3.9
Score for read counts	:	59.3
Score for mfe	:	2.4
Score for randfold	:	1.6
Score for cons. seed	:	-0.6
Total read count	:	128
Mature read count	:	115
Loop read count	:	0
Star read count	:	13

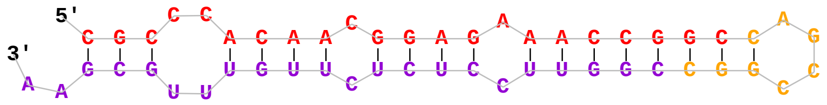

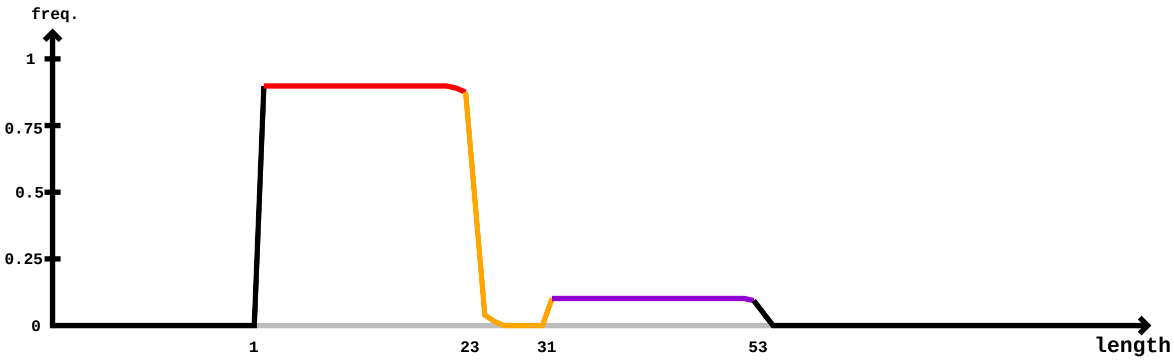

Mature Star

```
5'- gcgccaguccaucuuucucgcgcccacaacggagaaaccggccagccggccgguuccucucuuguuugcgaagagaaagacggauggcuuggcuggguuucaccgguacaag   -3'   obs
    gcgccaguccaucuuucucgcgcccacaacggagaaaccggccagccggccgguuccucucuuguuugcgaagagaaagacggauggcuuggcuggguuucaccgguacaag         exp
    ..((((.(((.((((((((.(((..((((.((((.((((((((....)))))))).)))).))))..)))..)))))))).)))))))...(((((......))))).....   reads   mm   sample
    ....................cgcccacaacggagaaaccggc......................................................................   12      0    NN8
    ....................cgcccacaacggagaaaccggcca....................................................................   1       0    NN8
    ....................cgcccacaacggagaaaccggccag...................................................................   2       0    NN8
    ..................................................cgguuccucucuuguuugcga.........................................   1       0    NN8
    ..................................................cgguuccucucuuguuugcgaa........................................   1       0    NN8
    ..................................................cgguuccucucuuguuugcgaU........................................   3       1    NN8

    ....................cgcccacaacggagaaaccg........................................................................   1       0    FF1
    ....................cgcccacaacggagaaaccgg.......................................................................   2       0    FF1
    ....................cgcccacaacggagaaaccggc......................................................................   91      0    FF1
    ....................Ggcccacaacggagaaaccggc......................................................................   1       1    FF1
    ....................cgcccacaacggagaaaAcggc......................................................................   1       1    FF1
    ....................cgcccacaacggagaaaccggcc.....................................................................   2       0    FF1
    ....................cgcccacaacggagaaaccggcca....................................................................   2       0    FF1
    ..................................................cgguuccucucuuguuugcgaU........................................   4       1    FF1
    ..................................................cgguuccucucCuguuugcgaa........................................   1       1    FF1
    ..................................................cgguuccucucuuguuugcgaa........................................   1       0    FF1
    ..................................................cgguuccucucuuguuugcgaC........................................   1       1    FF1
    ..................................................cgguuccucucuuguuugcgaag.......................................   1       0    FF1
```
