# Supplementary material for: Identification of Novel miRNAs and miRNA Expression Profiling in Wheat Hybrid Necrosis
Source: PLoS One. 2015 Feb 23;10(2):e0117507. doi: 10.1371/journal.pone.0117507 (PMC4338152; doi:10.1371/journal.pone.0117507)
Supplement: S2 Fig — Red colored letter: mature miRNA sequence; yellow colored letter: loop sequence; blue colored letter: miRNA* sequence. (ZIP) [file pone.0117507.s002.zip › Figures s1/contig2537991_14443.pdf]

Provisional ID	:	contig2537991_14443
Score total	:	175.5
Score for star read(s)	:	3.9
Score for read counts	:	164.8
Score for mfe	:	2.2
Score for randfold	:	1.6
Score for cons. seed	:	3
Total read count	:	335
Mature read count	:	223
Loop read count	:	0
Star read count	:	112

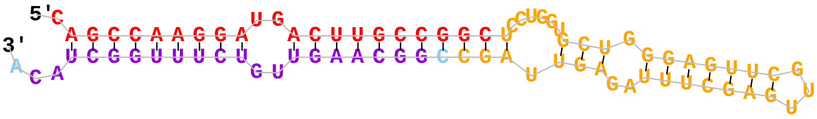

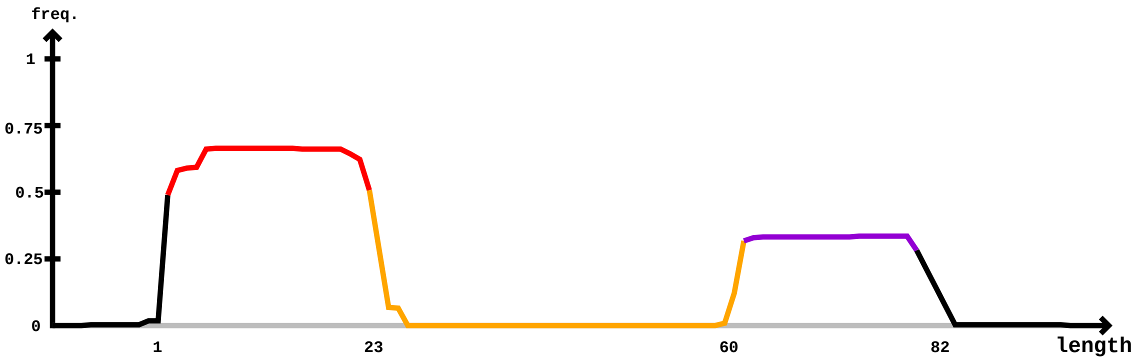

Mature **Star**

```
5'- gaacgggaugcagccaaggaugacuugccggcuccuggugcugggaguucguugagcuuuagaguuagccggcaaguugucuuuggcuacacuuaguucucuucuucu   -3'   obs
    gaacgggaugcagccaaggaugacuugccggcuccuggugcugggaguucguugagcuuuagaguuagccggcaaguugucuuuggcuacacuuaguucucuucuucu         exp
    ((((((.((.(((((((((..(((((((((((......(((.(((((((...)))))))..))).)))))))))))..))))))))).))))).)))).........   reads   mm   sample
    ..........Uagccaaggaugacuugccg..............................................................................   4       1    NN8
    ...........agccaaggaugacuugccggc............................................................................   6       0    NN8
    ............gccaaggaugacuugccgg.............................................................................   1       0    NN8
    ............gccaaggaugacuugccggc............................................................................   2       0    NN8
    ..............caaggaugacuugccggcucc.........................................................................   11      0    NN8
    ...............aaggaugacuugccggcu...........................................................................   1       0    NN8
    ....................................................................ccggcaaguugucuuuggcuac..................   1       0    NN8
    .....................................................................cggcaaguugucuuuAgcuac..................   1       1    NN8
    .....................................................................cggcaagCugucuuuggcuac..................   2       1    NN8
    .....................................................................cggcaaguugucuuuggcuac..................   10      0    NN8
    .....................................................................cggcaaguugucuuuggcuaca.................   2       0    NN8
    .....................................................................cggcaaguugucuuuggcuacU.................   1       1    NN8
    ......................................................................ggcaaguugucuuuggcu....................   8       0    NN8
    ......................................................................ggcaaguugucuuuggcua...................   3       0    NN8
    ......................................................................ggcaaguugucuuuggcuac..................   5       0    NN8
    ......................................................................ggcaaguugucuuuggcuaca.................   3       0    NN8
    ......................................................................ggcaagGugucuuuggcuaca.................   1       1    NN8
    .......................................................................gcaaguugucuuuggcuac..................   1       0    NN8

    ..acgggaugcagccaaggaugaU....................................................................................   1       1    FF1
    ........ugcagccaaggaugacuugcc...............................................................................   4       0    FF1
    ........ugcagAcaaggaugacuugcc...............................................................................   1       1    FF1
    ..........cagccaaggaugacuugcc...............................................................................   1       0    FF1
    ..........Uagccaaggaugacuugccg..............................................................................   2       1    FF1
    ..........cagccaaggaugacuugccU..............................................................................   1       1    FF1
    ..........cagccaaggaugacuugccgg.............................................................................   33      0    FF1
    ..........Uagccaaggaugacuugccgg.............................................................................   2       1    FF1
    ..........cagccaaggaugacuugccUg.............................................................................   1       1    FF1
    ..........cagGcaaggaugacuugccggc............................................................................   1       1    FF1
    ..........cagccaaggaugacuAgccggc............................................................................   1       1    FF1
    ..........cagccaaggaugacuugAcggc............................................................................   1       1    FF1
    ..........cagccaaggaugacuugccggc............................................................................   108     0    FF1
    ..........cagccaaggaugacuugccggU............................................................................   1       1    FF1
```

Mature   Star

```
gaacgggaugcagccaaggaugacuugccggcuccuggugcugggaguucguugagcuuuagaguuagccggcaaguugucuuuggcuacacuuaguucucuucuucu
..........caAccaaggaugacuugccggc............................................................................   1    1    FF1
..........cagccaaggaugUcuugccggc............................................................................   1    1    FF1
..........cagccaaggaugacuuCccggcu...........................................................................   1    1    FF1
...........agccaaggaugacuugccgg.............................................................................   1    0    FF1
...........agccaaggaugacuugcAgg.............................................................................   1    1    FF1
...........agccaaggaugacuugccggc............................................................................   17   0    FF1
...........agccaaggaugaAuugccggc............................................................................   1    1    FF1
...........Ggccaaggaugacuugccggcu...........................................................................   1    1    FF1
...........agccaaggaugacuugccggcu...........................................................................   4    0    FF1
.............ccaaggaugacuugccggc............................................................................   1    0    FF1
..............caaggaugacuugccggcuc..........................................................................   1    0    FF1
..............caaggaugacuugccggcucc.........................................................................   11   0    FF1
....................................................................ccggcaaguugucuuuggcuac..................   2    0    FF1
.....................................................................cggcaaguugucuuuggcu....................   4    0    FF1
.....................................................................cggcaaguugucuuuggcuac..................   16   0    FF1
.....................................................................cggcaaguugucuuuggcuacU.................   1    1    FF1
.....................................................................cggcaaguugucuuuggcuaca.................   1    0    FF1
......................................................................ggcaagGugucuuuggcu....................   1    1    FF1
......................................................................ggcaaguugucuuuggcu....................   5    0    FF1
......................................................................ggcaaguugucuuuggcua...................   7    0    FF1
......................................................................ggcaaguugucuuuggcuaU..................   1    1    FF1
......................................................................ggcaaguugucuuuggcuac..................   25   0    FF1
......................................................................ggcaaguugucuuuggcuaca.................   6    0    FF1
......................................................................ggcaaguugucuuuggcGaca.................   1    1    FF1
.......................................................................gcaaguugucuuuggcuac..................   1    0    FF1
.......................................................................gcaaguugucuuuggcuacU.................   1    1    FF1
.......................................................................gcaaguugucuuuggcuaca.................   1    0    FF1
........................................................................caaguugucuuuggcuac..................   1    0    FF1
..................................................................................uuggcuacacuuaguucucuuc....   1    0    FF1
```
