# Supplementary material for: Identification of Novel miRNAs and miRNA Expression Profiling in Wheat Hybrid Necrosis
Source: PLoS One. 2015 Feb 23;10(2):e0117507. doi: 10.1371/journal.pone.0117507 (PMC4338152; doi:10.1371/journal.pone.0117507)
Supplement: S2 Fig — Red colored letter: mature miRNA sequence; yellow colored letter: loop sequence; blue colored letter: miRNA* sequence. (ZIP) [file pone.0117507.s002.zip › Figures s1/contig288356_5066.pdf]

Provisional ID	:	contig288356_5066
Score total	:	684.6
Score for star read(s)	:	3.9
Score for read counts	:	674.1
Score for mfe	:	1.9
Score for randfold	:	1.6
Score for cons. seed	:	3
Total read count	:	1334
Mature read count	:	1332
Loop read count	:	0
Star read count	:	2

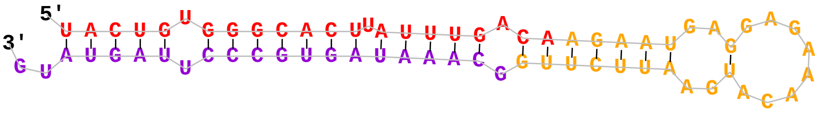

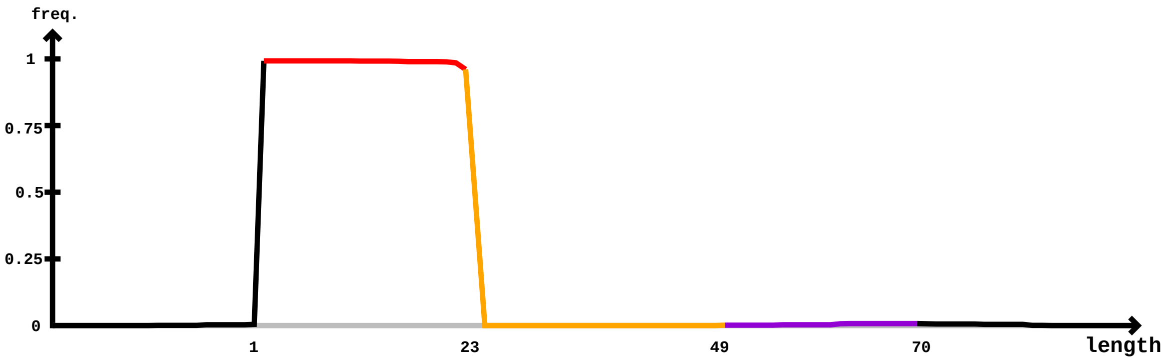

Mature **Star**

```
5'- ggcuauuggugagauuuccauacugugggcacuuauuugacaagaaugaggagaaacaugaauucuuggcaaauagugcccuuaguaugaaauucugaucaauaucuuugu  -3'   obs
    ggcuauuggugagauuuccauacugugggcacuuauuugacaagaaugaggagaaacaugaauucuuggcaaauagugcccuuaguaugaaauucugaucaauaucuuugu         exp
    ((.(((((((.(((.((.(((((((.(((((((.(((((.(((((((..(........)..))))))).)))))))))))).))))))).)).))).))))))).))....   reads   mm   sample
    ....................uacugugggcacuuauuugac......................................................................   1       0    NN8
    ....................uacAgugggcacuuauuugaca.....................................................................   1       1    NN8
    ....................uacugugggcacuuauuugaca.....................................................................   18      0    NN8

    .........ugagauuuccauacugugggc.................................................................................   1       0    FF1
    ..............uuuccauacugugggcacuu.............................................................................   1       0    FF1
    ..............Guuccauacugugggcacuua............................................................................   1       1    FF1
    ..............uuuccauacugugggcacuua............................................................................   1       0    FF1
    ...................Cuacugugggcacuuauuugaca.....................................................................   2       1    FF1
    ....................uacugugggcacuuauuuC........................................................................   1       1    FF1
    ....................Cacugugggcacuuauuuga.......................................................................   1       1    FF1
    ....................uacugugggcacuuauuuga.......................................................................   4       0    FF1
    ....................uacugugggcacuuauuugac......................................................................   32      0    FF1
    ....................uacugugggcacuuCuuugaca.....................................................................   1       1    FF1
    ....................uacugugggcaGuuauuugaca.....................................................................   1       1    FF1
    ....................Gacugugggcacuuauuugaca.....................................................................   4       1    FF1
    ....................uacGgugggcacuuauuugaca.....................................................................   2       1    FF1
    ....................Aacugugggcacuuauuugaca.....................................................................   2       1    FF1
    ....................uacugugggcacuuUuuugaca.....................................................................   1       1    FF1
    ....................uGcugugggcacuuauuugaca.....................................................................   3       1    FF1
    ....................uCcugugggcacuuauuugaca.....................................................................   1       1    FF1
    ....................uacugugggcacuuauuugaca.....................................................................   1236    0    FF1
    ....................uacCgugggcacuuauuugaca.....................................................................   1       1    FF1
    ....................uacugugggcacGuauuugaca.....................................................................   1       1    FF1
    ....................uaGugugggcacuuauuugaca.....................................................................   2       1    FF1
    ....................uacugugggcacuGauuugaca.....................................................................   1       1    FF1
    ....................uacugGgggcacuuauuugaca.....................................................................   2       1    FF1
    ....................uacugugggcacuuauuuAaca.....................................................................   1       1    FF1
    ....................uaAugugggcacuuauuugaca.....................................................................   1       1    FF1
    ....................uacugugggcaAuuauuugaca.....................................................................   2       1    FF1
    ....................uacuguggAcacuuauuugaca.....................................................................   1       1    FF1
    ....................uacugugggcacuuaGuugaca.....................................................................   1       1    FF1
    ....................uacugugggcacCuauuugaca.....................................................................   2       1    FF1
```

Mature **Star**

```
ggcuauuggugagauuuccauacugugggcacuuauuugacaagaaugaggagaaacaugaauucuuggcaaauagugcccuuaguaugaaauucugaucaauaucuuugu
....................uacugugggAacuuauuugaca.....................................................................    1    1    FF1
....................uacugugggcacuuauuugGca.....................................................................    1    1    FF1
....................uacugugggcacuuauAugaca.....................................................................    1    1    FF1
....................uacugugggcacuuaAuugaca.....................................................................    2    1    FF1
....................uacugugggcacuuauuugacaU....................................................................    1    1    FF1
....................................................................gcaaauagugcccuuaguaug......................    2    0    FF1
..........................................................................agugcccuuaguaugaaauuc................    2    0    FF1
................................................................................cuuaguaugaaauucugauc...........    5    0    FF1
.................................................................................uuaguaugaaauucugauAaa.........    1    1    FF1
```
