# Supplementary material for: Identification of Novel miRNAs and miRNA Expression Profiling in Wheat Hybrid Necrosis
Source: PLoS One. 2015 Feb 23;10(2):e0117507. doi: 10.1371/journal.pone.0117507 (PMC4338152; doi:10.1371/journal.pone.0117507)
Supplement: S2 Fig — Red colored letter: mature miRNA sequence; yellow colored letter: loop sequence; blue colored letter: miRNA* sequence. (ZIP) [file pone.0117507.s002.zip › Figures s1/contig2985567_15315.pdf]

Provisional ID	:	contig2985567_15315
Score total	:	2.2
Score for star read(s)	:	-1.3
Score for read counts	:	0
Score for mfe	:	2.5
Score for randfold	:	1.6
Score for cons. seed	:	-0.6
Total read count	:	13
Mature read count	:	13
Loop read count	:	0
Star read count	:	0

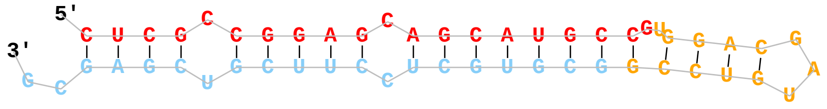

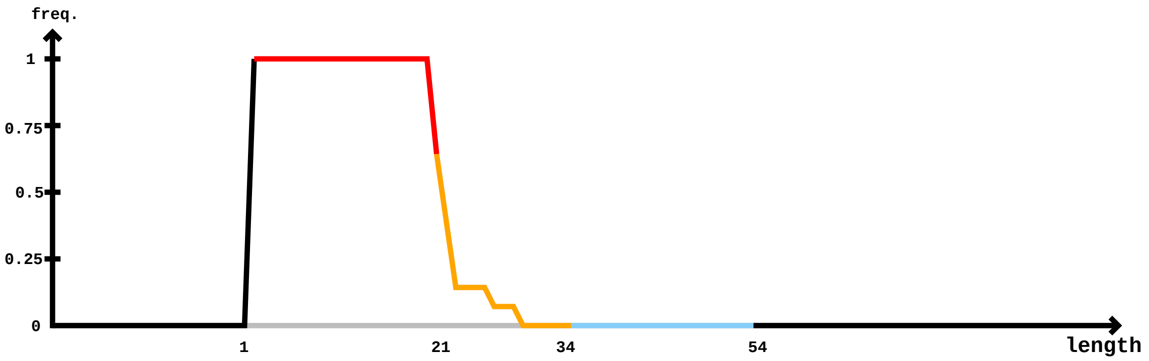

Mature **Star**

```
5'- uggcggcgcgccggcugcgcucgccggagcagcaugccguggacgauguccggcgugcuccuucgucgagcgugguugcuggcguccgugcacauggcgcaucaaggaa   -3'   exp
    ..((((.((((((((.(((((((.(((((.((((((((..((((...)))))))))))).))))).)))))))....))))))))))))((.....))..........   reads   mm   sample
    ...................cucgccggagcagcaugccA......................................................................   2       1    NN8

    ...................cucgccggagcagcaugcc.......................................................................   5       0    FF1
    ...................cucgccggagcagcaugccA......................................................................   5       1    FF1
    ...................cucgccggagcagcaugccAuggac.................................................................   1       1    FF1
    ...................cucgccggagcagcaugccguggacgaC..............................................................   1       1    FF1
```
