# Supplementary material for: Identification of Novel miRNAs and miRNA Expression Profiling in Wheat Hybrid Necrosis
Source: PLoS One. 2015 Feb 23;10(2):e0117507. doi: 10.1371/journal.pone.0117507 (PMC4338152; doi:10.1371/journal.pone.0117507)
Supplement: S2 Fig — Red colored letter: mature miRNA sequence; yellow colored letter: loop sequence; blue colored letter: miRNA* sequence. (ZIP) [file pone.0117507.s002.zip › Figures s1/contig3076104_15496.pdf]

Provisional ID	:	contig3076104_15496
Score total	:	0.6
Score for star read(s)	:	-1.3
Score for read counts	:	0
Score for mfe	:	0.9
Score for randfold	:	1.6
Score for cons. seed	:	-0.6
Total read count	:	14
Mature read count	:	14
Loop read count	:	0
Star read count	:	0

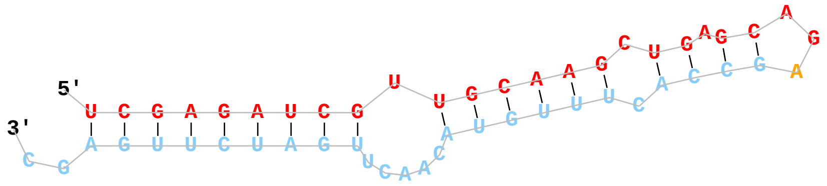

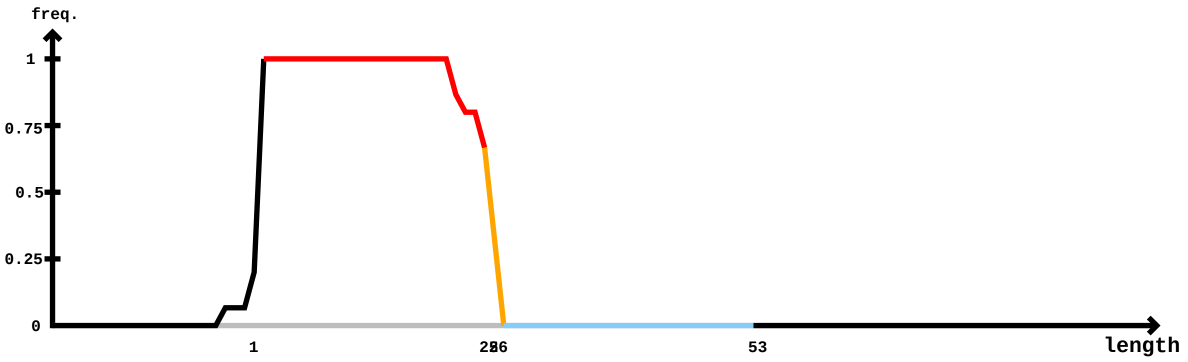

Mature **Star**

```
5'- uuauugagcucaaaauuagcucgagaucguugcaagcugagcagagccacuuuguacaacuugaucuugagcuucgcucaauuuaugcucgcuuaaauuuuaaaugaguugag   -3'    exp
    ..(((((((........((((((((((((.((((((.((.((...)))).))))))).....))))))))))))..)))))))....(((((.(((.....))).)))))....    reads      mm         sample
    ................uagcucgagaucguugcaagcuga.........................................................................    1          0          NN8
    ....................ucgagaucguugcaagcugagcaU.....................................................................    6          1          NN8

    ...................cucgagaucguugcaagcugagca......................................................................    2          0          FF1
    ....................ucgagaucguugcaagcuga.........................................................................    1          0          FF1
    ....................ucgagaucguuAcaagcugag........................................................................    1          1          FF1
    ....................ucgagaucguugcaagcugagcaU.....................................................................    4          1          FF1
```
