# Supplementary material for: Identification of Novel miRNAs and miRNA Expression Profiling in Wheat Hybrid Necrosis
Source: PLoS One. 2015 Feb 23;10(2):e0117507. doi: 10.1371/journal.pone.0117507 (PMC4338152; doi:10.1371/journal.pone.0117507)
Supplement: S2 Fig — Red colored letter: mature miRNA sequence; yellow colored letter: loop sequence; blue colored letter: miRNA* sequence. (ZIP) [file pone.0117507.s002.zip › Figures s1/contig31094_1188.pdf]

Provisional ID	: contig31094_1188
Score total	: 61.6
Score for star read(s)	: 3.9
Score for read counts	: 62.8
Score for mfe	: -2.2
Score for randfold	: -2.2
Score for cons. seed	: -0.6
Total read count	: 135
Mature read count	: 123
Loop read count	: 3
Star read count	: 9

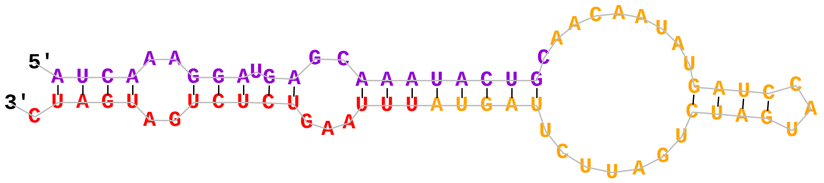

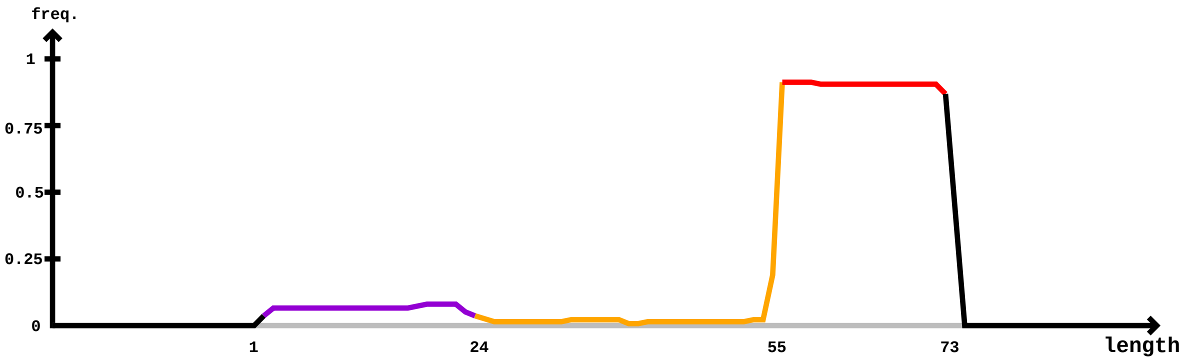

Star Mature

5'- acugucgccauggcgcauguaucaaaggaugagcaaauacugcaacaauaugauccaugaucugauucuuaguauuuaagucucugaugaucucucugccaaugcgaguuuuc -3' obs

acugucgccauggcgcauguaucaaaggaugagcaaauacugcaacaauaugauccaugaucugauucuuaguauuuaagucucugaugaucucucugccaaugcgaguuuuc exp

....((((..(((((.....((((..(((.((..((((((((.........((((...)))).......))))))))...)))))..)))).....)))))..))))......	reads	mm	sample
....................aucaaaggaugagAaaauacu..	1	1	NN8
....................................auacugUaacaauaugauccau...	1	1	NN8
.....................................uacugUaacaauaugauccau...	1	1	NN8
....ucugauucuuaguauuua...................................	1	0	NN8
....guauuGaagucucugaugauc.....................	1	1	NN8
....auuuaGgucucugaugau......................	2	1	NN8
....auuuaagucucAgaugau......................	1	1	NN8
....auuuaagucucugGugauc.....................	1	1	NN8
....auuuaagucucGgaugauc.....................	2	1	NN8
....auuuaagucucuAaugauc.....................	2	1	NN8
....auuuGagucucugaugauc.....................	1	1	NN8
....auuAaagucucugaugauc.....................	1	1	NN8
....auuuaagucucugaGgauc.....................	1	1	NN8
....auuuaagucAcugaugauc.....................	1	1	NN8
....auuuUagucucugaugauc.....................	1	1	NN8
....auuuaagucucugaCgauc.....................	1	1	NN8
....auuuaGgucucugaugauc.....................	1	1	NN8
....auCuaagucucugaugauc.....................	1	1	NN8
....auuuaaguAucugaugauc.....................	1	1	NN8
....auuuCagucucugaugauc.....................	1	1	NN8
....auGuaagucucugaugauc.....................	2	1	NN8
....uuuaagucucugaugauA.....................	83	1	NN8
....................aucaaaggaugagcaaauacu..	1	0	FF1
....................aucaaaggaugagcaaauacugc..	3	0	FF1
.....................ucaaaggaugagcaaauacu..	2	0	FF1
.....................ucaaaggaugagcaaauacug...	2	0	FF1
....auccaugaucugauucuuagu..	1	0	FF1
....auuGaagucucugaugau......................	1	1	FF1
....auuuaagucucAgaugau......................	1	1	FF1
....auuuaagucucGgaugauc.....................	1	1	FF1
....auuuaagucucuUaugauc.....................	1	1	FF1
....uuuaagucucugaugauA.....................	16	1	FF1

Star Mature

acugucgccauggcgcauguaucaaaggaugagcaaauacugcaacaauaugauccaugaucugauucuuaguauuuaagucucugaugaucucucugccaaugcgaguuuuc
