# Supplementary material for: Identification of Novel miRNAs and miRNA Expression Profiling in Wheat Hybrid Necrosis
Source: PLoS One. 2015 Feb 23;10(2):e0117507. doi: 10.1371/journal.pone.0117507 (PMC4338152; doi:10.1371/journal.pone.0117507)
Supplement: S2 Fig — Red colored letter: mature miRNA sequence; yellow colored letter: loop sequence; blue colored letter: miRNA* sequence. (ZIP) [file pone.0117507.s002.zip › Figures s1/contig316572_5328.pdf]

Provisional ID	:	contig316572_5328
Score total	:	9278.9
Score for star read(s)	:	3.9
Score for read counts	:	9267.7
Score for mfe	:	2.7
Score for randfold	:	1.6
Score for cons. seed	:	3
Total read count	:	18190
Mature read count	:	15464
Loop read count	:	0
Star read count	:	2726

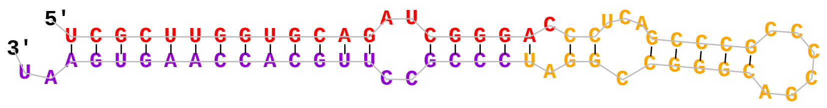

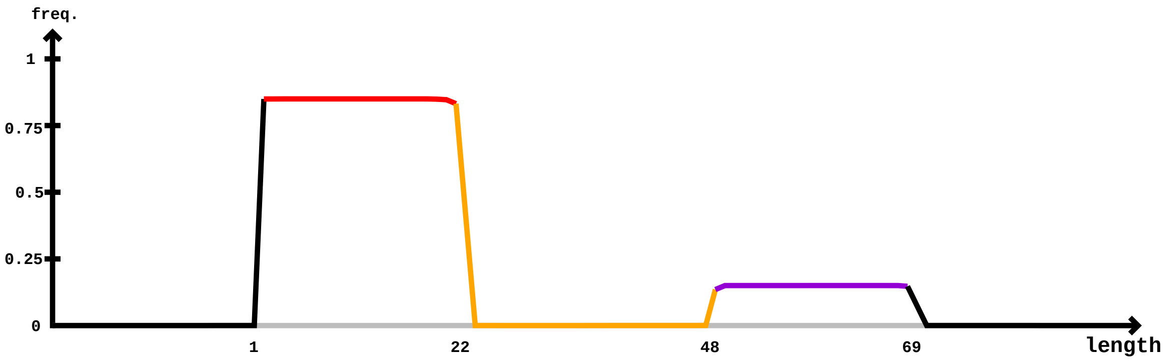

Mature **Star**

```
5'- ccgucgccgccgccucgggcucgcuuggugcagaucgggacccucagcccgccccgacgggccggaucccgccuugcaccaagugaaucggagccggcgcagcgaccacgc   -3'   obs
    ccgucgccgccgccucgggcucgcuuggugcagaucgggacccucagcccgccccgacgggccggaucccgccuugcaccaagugaaucggagccggcgcagcgaccacgc         exp
    ..((((((((((.(((.(..((((((((((((..(((((.((...(((((......))))).)).)))))..))))))))))))..).))).))))))..)))))).....   reads   mm   sample
    ....................ucgcuuggugcagaucgg.........................................................................   2       0    NN8
    ....................ucgcuuggugcagaucggg........................................................................   6       0    NN8
    ....................ucgcuuggugcagaucggga.......................................................................   80      0    NN8
    ....................Ccgcuuggugcagaucggga.......................................................................   1       1    NN8
    ....................ucgcuuggGgcagaucggga.......................................................................   1       1    NN8
    ....................ucgcGuggugcagaucggga.......................................................................   1       1    NN8
    ....................ucgcuuggugcagauUgggac......................................................................   2       1    NN8
    ....................ucgcuCggugcagaucgggac......................................................................   1       1    NN8
    ....................ucAcuuggugcagaucgggac......................................................................   2       1    NN8
    ....................ucgcuuggugcagaCcgggac......................................................................   2       1    NN8
    ....................Gcgcuuggugcagaucgggac......................................................................   2       1    NN8
    ....................ucgcuuggugcGgaucgggac......................................................................   2       1    NN8
    ....................uGgcuuggugcagaucgggac......................................................................   4       1    NN8
    ....................ucgcuuggugcagaucgUgac......................................................................   1       1    NN8
    ....................Acgcuuggugcagaucgggac......................................................................   3       1    NN8
    ....................ucgcuuggugcagaucggUac......................................................................   1       1    NN8
    ....................ucgcuuggugcagaucgggaA......................................................................   1       1    NN8
    ....................uAgcuuggugcagaucgggac......................................................................   1       1    NN8
    ....................ucgcuuggugcagauGgggac......................................................................   1       1    NN8
    ....................ucgcuuggugcagaGcgggac......................................................................   1       1    NN8
    ....................ucgcuuggugcagaucgAgac......................................................................   1       1    NN8
    ....................ucgcuuggugcUgaucgggac......................................................................   2       1    NN8
    ....................ucgcuuggugcagCucgggac......................................................................   1       1    NN8
    ....................ucgcuuggAgcagaucgggac......................................................................   3       1    NN8
    ....................ucgcuuggugcagaucgggac......................................................................   1931    0    NN8
    ....................ucgcuuAgugcagaucgggac......................................................................   2       1    NN8
    ....................ucgcuuggugcaUaucgggac......................................................................   2       1    NN8
    ....................ucgcuugguUcagaucgggac......................................................................   1       1    NN8
    ....................ucgcuuggugcagaucAggac......................................................................   3       1    NN8
    ....................ucgcuuggugcCgaucgggac......................................................................   2       1    NN8
    ....................ucgcuuggugGagaucgggac......................................................................   2       1    NN8
    ....................ucgAuuggugcagaucgggac......................................................................   1       1    NN8
    ....................ucgcuuggugUagaucgggac......................................................................   1       1    NN8
```

Mature   Star

```
ccgucgccgccgccucgggcucgcuuggugcagaucgggacccucagcccgccccgacgggccggaucccgccuugcaccaagugaaucggagccggcgcagcgaccacgc
....................ucgcuuggGgcagaucgggac......................................................................   5     1   NN8
....................ucgcuuggugcaAaucgggac......................................................................   2     1   NN8
....................ucgcuuggugcagaucgggaU......................................................................   20    1   NN8
....................ucgcCuggugcagaucgggac......................................................................   1     1   NN8
....................ucgcuuggugcagauAgggac......................................................................   2     1   NN8
....................ucCcuuggugcagaucgggac......................................................................   1     1   NN8
....................ucgcuuggugcagaucggAac......................................................................   1     1   NN8
....................uUgcuuggugcagaucgggac......................................................................   1     1   NN8
....................ucgcuGggugcagaucgggac......................................................................   1     1   NN8
....................ucgcuuUgugcagaucgggac......................................................................   1     1   NN8
....................ucgcuuggugAagaucgggac......................................................................   4     1   NN8
....................ucgcuuggugcagGucgggac......................................................................   1     1   NN8
....................ucgcGuggugcagaucgggac......................................................................   1     1   NN8
....................ucgcuuggugcagaucgggacc.....................................................................   33    0   NN8
....................ucgcuuggugcagaucgggacU.....................................................................   16    1   NN8
....................ucgcuuggugcagaucgggacA.....................................................................   1     1   NN8
....................ucgcuuggugcagaucgggacccuc..................................................................   1     0   NN8
.....................cgcuuggugcagaucgggac......................................................................   2     0   NN8
......................gcuuggugcagaucgggac......................................................................   1     0   NN8
........................................................acgggccggaucccgccuugcaccaagugaau.......................   1     0   NN8
..............................................................cggaucccgccuugcaccaagugaU........................   1     1   NN8
..................................................................Ccccgccuugcaccaagugaau.......................   1     1   NN8
...................................................................cccgccuugcaccaagugaa........................   9     0   NN8
...................................................................cccgccuugcaccaGgugaau.......................   1     1   NN8
...................................................................cccgccuugcaccaagugaGu.......................   1     1   NN8
...................................................................cccgccuugcaccUagugaau.......................   1     1   NN8
...................................................................cccgccuugcaccaagugaaA.......................   3     1   NN8
...................................................................cccgccuugcaccaagugaau.......................   342   0   NN8
...................................................................cccgcAuugcaccaagugaau.......................   1     1   NN8
...................................................................cccgccuugcaccaagugaCu.......................   1     1   NN8
...................................................................cccgccuugcaccaaAugaau.......................   2     1   NN8
...................................................................cccgcGuugcaccaagugaau.......................   1     1   NN8
...................................................................cccgccuugcaccaagugaauU......................   1     1   NN8
....................................................................ccgccuugcaccaagugaa........................   5     0   NN8
....................................................................ccgccuAgcaccaagugaau.......................   1     1   NN8
....................................................................Acgccuugcaccaagugaau.......................   2     1   NN8
....................................................................ccgccuugcaccaagugaau.......................   108   0   NN8
....................................................................ccgcGuugcaccaagugaau.......................   2     1   NN8
....................................................................ccgccuugcaccaagugaUu.......................   2     1   NN8
....................................................................ccgccuugcaccaagugaauU......................   1     1   NN8

...............cgggcucgcuuggugcagauc...........................................................................   1     0   FF1
....................ucgcuuggAgcagaucgg.........................................................................   1     1   FF1
....................ucgcuuggugcagaucgU.........................................................................   1     1   FF1
....................ucgcuuggugcagaucgg.........................................................................   11    0   FF1
....................ucgcuuggugcaUaucggg........................................................................   1     1   FF1
....................ucgcuuggugcaAaucggg........................................................................   1     1   FF1
....................ucgcuuggugcagaucggg........................................................................   30    0   FF1
....................ucgcuuggugcagaucgggG.......................................................................   1     1   FF1
....................ucAcuuggugcagaucggga.......................................................................   1     1   FF1
....................Gcgcuuggugcagaucggga.......................................................................   3     1   FF1
....................ucgcuuggugcagaCcggga.......................................................................   1     1   FF1
....................ucgcuuggugcagaucggga.......................................................................   181   0   FF1
....................ucgcuuggugcagaucgggU.......................................................................   1     1   FF1
....................uAgcuuggugcagaucggga.......................................................................   1     1   FF1
....................ucgcuuggugcagaucCggac......................................................................   4     1   FF1
....................uGgcuuggugcagaucgggac......................................................................   21    1   FF1
....................Acgcuuggugcagaucgggac......................................................................   19    1   FF1
....................ucgcuuggugcagauUgggac......................................................................   11    1   FF1
....................ucgcuuggugcagCucgggac......................................................................   1     1   FF1
....................ucgcuugguAcagaucgggac......................................................................   2     1   FF1
....................ucgcuuUgugcagaucgggac......................................................................   4     1   FF1
....................ucgUuuggugcagaucgggac......................................................................   2     1   FF1
....................ucgcuuggugcagaucggAac......................................................................   6     1   FF1
....................ucgcuuggugcagaucgggCc......................................................................   1     1   FF1
....................ucgcuuggAgcagaucgggac......................................................................   20    1   FF1
....................ucgcuuggugcagauAgggac......................................................................   18    1   FF1
....................uAgcuuggugcagaucgggac......................................................................   8     1   FF1
....................ucgcuuggugcagaucgAgac......................................................................   9     1   FF1
....................ucgcuuggugcagGucgggac......................................................................   4     1   FF1
```

Mature **Star**

ccgucgccgccgccucgggcucgcuuggugcagaucgggacccucagcccgccccgacgggccggaucccgccuugcaccaagugaaucggagccggcgcagcgaccacgc			
....................ucgcuuggugGagaucgggac..	7	1	FF1
....................ucgcuuggugcaUaucgggac..	11	1	FF1
....................ucgcuuggugcagaucUggac..	5	1	FF1
....................ucgcCuggugcagaucgggac..	1	1	FF1
....................ucgcuuggugcagaCcgggac..	3	1	FF1
....................ucgAuuggugcagaucgggac..	5	1	FF1
....................ucgcuuggGgcagaucgggac..	47	1	FF1
....................ucgcuuggugcagaucgggaU..	161	1	FF1
....................ucgcuugCugcagaucgggac..	1	1	FF1
....................ucgcuuggugcaAaucgggac..	9	1	FF1
....................ucgcuuggugcagaucgggaA..	3	1	FF1
....................ucgcuuggugcagaucgCgac..	9	1	FF1
....................ucUcuuggugcagaucgggac..	2	1	FF1
....................ucgcuuggugcagaucggUac..	4	1	FF1
....................ucgcuuggugcaCaucgggac..	26	1	FF1
....................ucgcuuggugcagaucgUgac..	4	1	FF1
....................ucgcuugguCcagaucgggac..	2	1	FF1
....................ucgcuuggugcagaGcgggac..	1	1	FF1
....................Ccgcuuggugcagaucgggac..	8	1	FF1
....................ucgcuuggugcagaucgggac..	12265	0	FF1
....................ucAcuuggugcagaucgggac..	12	1	FF1
....................ucgcuuggugUagaucgggac..	3	1	FF1
....................ucgcuuggugcagaucggCac..	3	1	FF1
....................ucgcuuggugcagUucgggac..	2	1	FF1
....................ucgcAuggugcagaucgggac..	3	1	FF1
....................ucgcuuAgugcagaucgggac..	4	1	FF1
....................ucCcuuggugcagaucgggac..	6	1	FF1
....................Gcgcuuggugcagaucgggac..	42	1	FF1
....................ucgGuuggugcagaucgggac..	11	1	FF1
....................ucgcuugAugcagaucgggac..	7	1	FF1
....................ucgcuuggCgcagaucgggac..	14	1	FF1
....................ucgcuAggugcagaucgggac..	2	1	FF1
....................ucgcuuggugcagaucgggGc..	7	1	FF1
....................uUgcuuggugcagaucgggac..	6	1	FF1
....................ucgcuuCgugcagaucgggac..	4	1	FF1
....................ucgcuuggugAagaucgggac..	8	1	FF1
....................ucgcuuggugcGgaucgggac..	11	1	FF1
....................ucgcuuggugcagauGgggac..	28	1	FF1
....................ucgcuuggugcagaucgggacA...	9	1	FF1
....................ucgcuuggugcagaucgggacc...	74	0	FF1
....................ucgcuuggugcagauGgggacc...	1	1	FF1
....................uUgcuuggugcagaucgggacc...	1	1	FF1
....................ucgcuuggCgcagaucgggacc...	1	1	FF1
....................ucgcuuggugcaAaucgggacc...	1	1	FF1
....................Acgcuuggugcagaucgggacc...	2	1	FF1
....................ucgcuuggugcagaucgggacU...	97	1	FF1
....................ucgcuuggugcagaucgggaccU..	1	1	FF1
....................ucgcuuggugcagaucgggaccc..	1	0	FF1
......................gcuuggugcagaucgggac..	2	0	FF1
.......................cuuggugcagaucgggacccu...	1	0	FF1
....cgacgggccggaucccgccuugcaccaagGga.........................	1	1	FF1
....acgggccggaucccgccuugcaccaagug..........................	1	0	FF1
....ucccgccuugcaccaagugaa........................	1	0	FF1
....Ccccgccuugcaccaagugaau.......................	1	1	FF1
....ucccgccuugcaccaagugaau.......................	1	0	FF1
....cccgccuugcaccaagugaU........................	1	1	FF1
....cccgccuugcaccaagugaa........................	32	0	FF1
....Gccgccuugcaccaagugaau.......................	4	1	FF1
....cccgccuugcacUaagugaau.......................	4	1	FF1
....cccgccuugcaccaaAugaau.......................	3	1	FF1
....ccUgccuugcaccaagugaau.......................	1	1	FF1
....cAcgccuugcaccaagugaau.......................	4	1	FF1
....cccgccuugcaccaagugaGu.......................	1	1	FF1
....cccgccuugcaccaagGgaau.......................	5	1	FF1
....cccgccuugcaccaaguCaau.......................	1	1	FF1
....cccgccuugcacGaagugaau.......................	4	1	FF1
....cccgccuugcaccaagugaau.......................	1973	0	FF1
....cccgccuugcaccaagugUau.......................	2	1	FF1
....cccgccuugcaccaagugaaA.......................	1	1	FF1
....cccgccuugcaccaagAgaau.......................	2	1	FF1

```
                    Mature                                         Star
ccgucgccgccgccucgggcucgcuuggugcagaucgggacccucagcccgccccgacgggccggaucccgccuugcaccaagugaaucggagccggcgcagcgaccacgc
...................................................................cccgccuugcaccaagCgaau.......................   5     1    FF1
...................................................................cUcgccuugcaccaagugaau.......................   2     1    FF1
...................................................................cccUccuugcaccaagugaau.......................   1     1    FF1
...................................................................ccGgccuugcaccaagugaau.......................   2     1    FF1
...................................................................cccgccuugcaccaaguAaau.......................   3     1    FF1
...................................................................cccgccCugcaccaagugaau.......................   1     1    FF1
...................................................................Accgccuugcaccaagugaau.......................   4     1    FF1
...................................................................cccCccuugcaccaagugaau.......................   1     1    FF1
...................................................................cccgccuugcaccaaguUaau.......................   2     1    FF1
...................................................................cGcgccuugcaccaagugaau.......................   4     1    FF1
...................................................................cccgccuugcGccaagugaau.......................   1     1    FF1
...................................................................cccgccuugcacAaagugaau.......................   5     1    FF1
...................................................................Uccgccuugcaccaagugaau.......................   4     1    FF1
...................................................................cccgccuugcaccaagugaauU......................   11    1    FF1
...................................................................cccgccuugcaccaagugaauA......................   2     1    FF1
....................................................................ccgccuugcaccaagugaa........................   2     0    FF1
....................................................................ccgccuugcaccaagugaau.......................   142   0    FF1
....................................................................ccgccuugcaGcaagugaau.......................   1     1    FF1
....................................................................cGgccuugcaccaagugaau.......................   1     1    FF1
....................................................................Ucgccuugcaccaagugaau.......................   1     1    FF1
....................................................................cUgccuugcaccaagugaau.......................   2     1    FF1
....................................................................ccgccuugcaccaaguAaau.......................   1     1    FF1
....................................................................ccgccuugcaccaagugaauA......................   1     1    FF1
........................................................................cuugcaccaagugaaucggag..................   1     0    FF1
.............................................................................accaagugaaucggagccggcgca..........   1     0    FF1
```
