# Supplementary material for: Identification of Novel miRNAs and miRNA Expression Profiling in Wheat Hybrid Necrosis
Source: PLoS One. 2015 Feb 23;10(2):e0117507. doi: 10.1371/journal.pone.0117507 (PMC4338152; doi:10.1371/journal.pone.0117507)
Supplement: S2 Fig — Red colored letter: mature miRNA sequence; yellow colored letter: loop sequence; blue colored letter: miRNA* sequence. (ZIP) [file pone.0117507.s002.zip › Figures s1/contig3205398_15694.pdf]

Provisional ID	:	contig3205398_15694
Score total	:	1.9
Score for star read(s)	:	-1.3
Score for read counts	:	0
Score for mfe	:	2.2
Score for randfold	:	1.6
Score for cons. seed	:	-0.6
Total read count	:	12
Mature read count	:	12
Loop read count	:	0
Star read count	:	0

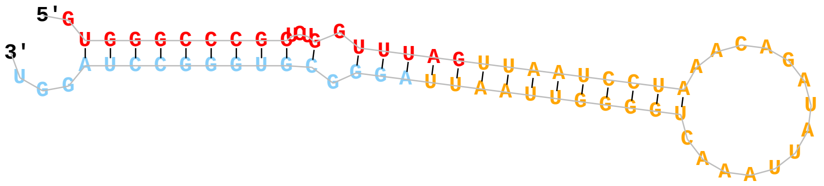

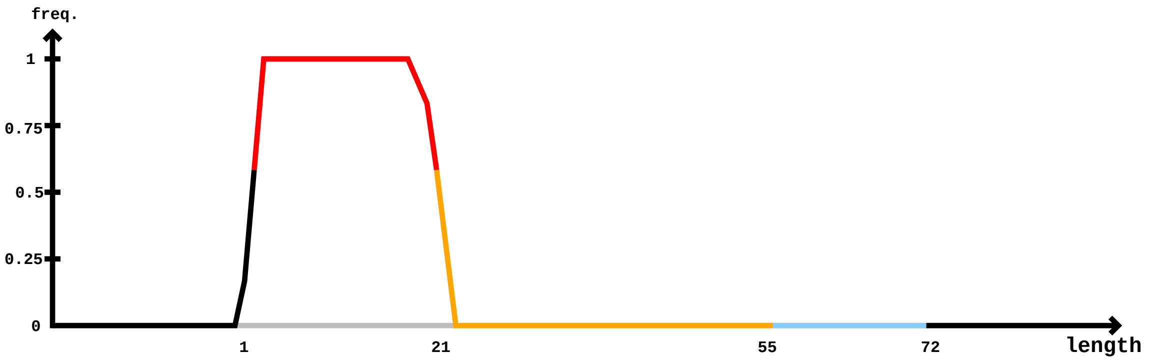

Mature **Star**

```
5'- gcuuggucaaaacugacgcgugggcccgcucugguuuaguuaauccuaaacagauauuaaacugggguuaauuagggcgugggccuaggugucagugacacggugcggu   -3'    exp
    (((((((...((((((((.(((((((((...(.((((((((((((((.............)))))))))))))).))))))))).))))))))))).))).))...        reads        mm           sample
    ..................cAugggcccgcucugguuua.......................................................................        1            1            NN8
    ....................ugggcccgcucugguuCa.......................................................................        2            1            NN8
    ....................ugggcccgcucugguuuag......................................................................        2            0            NN8
    ....................ugggcccgcucugguuuagA.....................................................................        1            1            NN8

    ..................cAugggcccgcucugguu.........................................................................        1            1            FF1
    ...................Augggcccgcucugguuu........................................................................        1            1            FF1
    ...................Augggcccgcucugguuuag......................................................................        4            1            FF1
```
