# Supplementary material for: Identification of Novel miRNAs and miRNA Expression Profiling in Wheat Hybrid Necrosis
Source: PLoS One. 2015 Feb 23;10(2):e0117507. doi: 10.1371/journal.pone.0117507 (PMC4338152; doi:10.1371/journal.pone.0117507)
Supplement: S2 Fig — Red colored letter: mature miRNA sequence; yellow colored letter: loop sequence; blue colored letter: miRNA* sequence. (ZIP) [file pone.0117507.s002.zip › Figures s1/contig346286_5611.pdf]

Provisional ID	:	contig346286_5611
Score total	:	2
Score for star read(s)	:	-1.3
Score for read counts	:	0
Score for mfe	:	2.3
Score for randfold	:	1.6
Score for cons. seed	:	-0.6
Total read count	:	228
Mature read count	:	228
Loop read count	:	0
Star read count	:	0

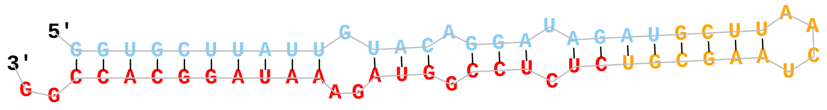

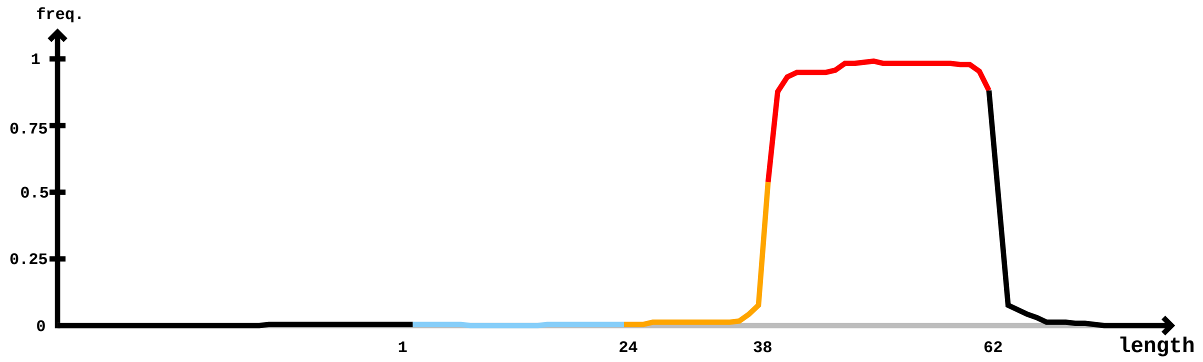

Star Mature

```
5'- ugcuuagagaauaaaccaggcuuuucuuaagcaccggugcuuauuguacaggauagaugcuuaacuaagcgucucuccgguagaaauaggcaccggugcuucagaaaaaaaccg   -3'    exp
    ..................((.((((((.((((((((((((((((.(((.(((.(((((((((....))))))))).))).)))..)))))))))))))))).))))))...)).   reads   mm   sample
    ............................................................uuaacuaagcgucucuccgguaga..............................   2       0    NN8
    ......................................................................gucucuccgguagaaauaggcacc....................   3       0    NN8
    ........................................................................cucuccUguagaaauaggcaccg...................   3       1    NN8
    ........................................................................cucuccUguagaaauaggcaccgg..................   40      1    NN8
    .........................................................................ucuccUguagaaauaggcaccg...................   1       1    NN8
    .........................................................................ucuccUguagaaauaggcaccgg..................   4       1    NN8
    .........................................................................ucuccUguagaaauaggcaccggu.................   42      1    NN8
    ..........................................................................cuccUguagaaauaggcaccggu.................   2       1    NN8
    ..........................................................................cuccUguagaaauaggcaccggug................   2       1    NN8
    ...........................................................................uccUguagaaauaggcaccggugc...............   2       1    NN8
    ...........................................................................uccCguagaaauaggcaccggugc...............   1       1    NN8

    ....................cuuuucuuaagcaccggugcu.........................................................................   1       0    FF1
    .................................................aggauagaugcuuaacuaagcguc.........................................   1       0    FF1
    .....................................................................cgucucuccgguagaaauaggca......................   1       0    FF1
    ......................................................................gucucuccgguagaaauaggcacc....................   2       0    FF1
    ......................................................................gucucuccCguagaaauaggcacc....................   1       1    FF1
    .......................................................................ucucuccUguagaaauaggcaccg...................   7       1    FF1
    .......................................................................ucucuccUguagaaauaggcaccgg..................   1       1    FF1
    ........................................................................cucuccUguagaaauaggcaccg...................   4       1    FF1
    ........................................................................cucuccUguagaaauaggcaccgg..................   62      1    FF1
    .........................................................................ucuccUguagaaauaggcaccg...................   1       1    FF1
    .........................................................................ucuccUguagaaauaggcaccgg..................   2       1    FF1
    .........................................................................ucuccUguagaaauaggcaccggu.................   31      1    FF1
    ..........................................................................cuccUguagaaauaggcaccg...................   1       1    FF1
    ..........................................................................cuccUguagaaauaggcaccgg..................   5       1    FF1
    ..........................................................................cuccUguagaaauaggcaccggu.................   1       1    FF1
    ..........................................................................cuccUguagaaauaggcaccggug................   2       1    FF1
    ...........................................................................uccCguagaaauaggcaccggugc...............   1       1    FF1
    ...............................................................................guagaaauaggcaccggugcu..............   2       0    FF1
    ................................................................................uagaaauaggcaUcggugcu..............   1       1    FF1
    ................................................................................uagaaauaggcaUcggugcuu.............   4       1    FF1
    ................................................................................uagaaauaggcaccggugcuucag..........   1       0    FF1
    ..................................................................................gaaauaggcaccggugcuucUgaa........   1       1    FF1
```

```
                                            Star                                          Mature
ugcuuagagaauaaaccaggcuuuucuuaagcaccggugcuuauuguacaggauagaugcuuaacuaagcgucucuccgguagaaauaggcaccggugcuucagaaaaaaaccg
...................................................................................aaauaggcaccgguAcuucagaaa.......    1    1    FF1
```
