# Supplementary material for: Identification of Novel miRNAs and miRNA Expression Profiling in Wheat Hybrid Necrosis
Source: PLoS One. 2015 Feb 23;10(2):e0117507. doi: 10.1371/journal.pone.0117507 (PMC4338152; doi:10.1371/journal.pone.0117507)
Supplement: S2 Fig — Red colored letter: mature miRNA sequence; yellow colored letter: loop sequence; blue colored letter: miRNA* sequence. (ZIP) [file pone.0117507.s002.zip › Figures s1/contig346461_5614.pdf]

Provisional ID	:	contig346461_5614
Score total	:	4.3
Score for star read(s)	:	-1.3
Score for read counts	:	-0.8
Score for mfe	:	1.9
Score for randfold	:	1.6
Score for cons. seed	:	3
Total read count	:	10
Mature read count	:	10
Loop read count	:	0
Star read count	:	0

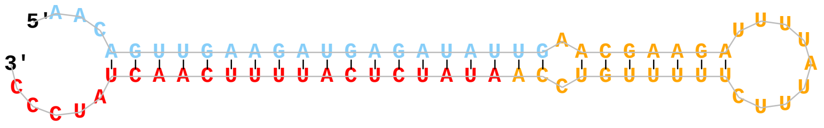

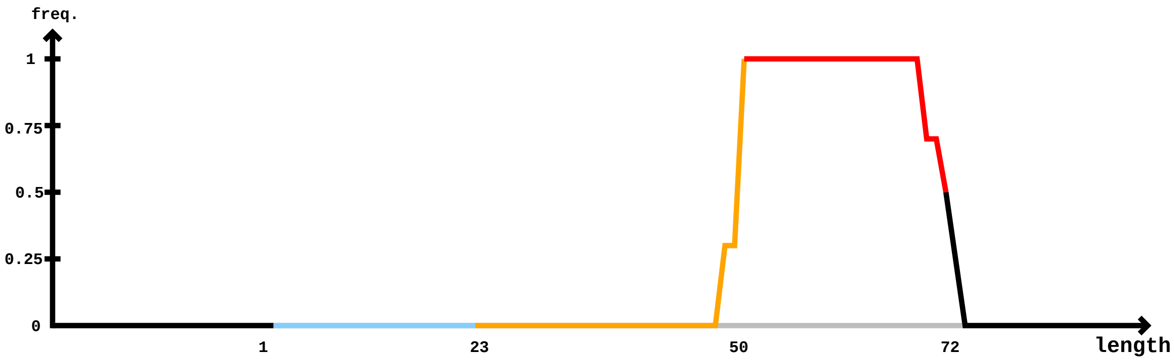

Star Mature

```
5'- ggaugaaaccaaaucuucaggaacaguugaagaugagauauugaacgaagauuuuauuucuuuuuguccaauaucucauuuucaacuaucccugaugauacccacaacaucu   -3'   exp
    (((((.......(((.(((((...(((((((((((((((((((.(((((((.........))))))).)))))))))))))))))))...))))).)))........)))))     reads   mm   sample
    ....................................................................caauaucucauuuucaacuau.......................     2       0    NN8
    ....................................................................caauaucAcauuuucaacuau.......................     1       1    NN8

    ......................................................................auaucucauuuucaacuaucc.....................     1       0    FF1
    ......................................................................aGaucucauuuucaacuaucc.....................     1       1    FF1
    ......................................................................auaucucauuuucaacuauccc....................     5       0    FF1
```
