# Supplementary material for: Identification of Novel miRNAs and miRNA Expression Profiling in Wheat Hybrid Necrosis
Source: PLoS One. 2015 Feb 23;10(2):e0117507. doi: 10.1371/journal.pone.0117507 (PMC4338152; doi:10.1371/journal.pone.0117507)
Supplement: S2 Fig — Red colored letter: mature miRNA sequence; yellow colored letter: loop sequence; blue colored letter: miRNA* sequence. (ZIP) [file pone.0117507.s002.zip › Figures s1/contig3589520_16257.pdf]

Provisional ID	:	contig3589520_16257
Score total	:	2.5
Score for star read(s)	:	-1.3
Score for read counts	:	0
Score for mfe	:	2.8
Score for randfold	:	1.6
Score for cons. seed	:	-0.6
Total read count	:	51
Mature read count	:	51
Loop read count	:	0
Star read count	:	0

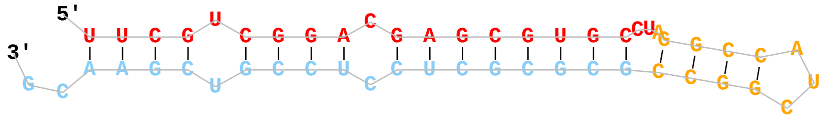

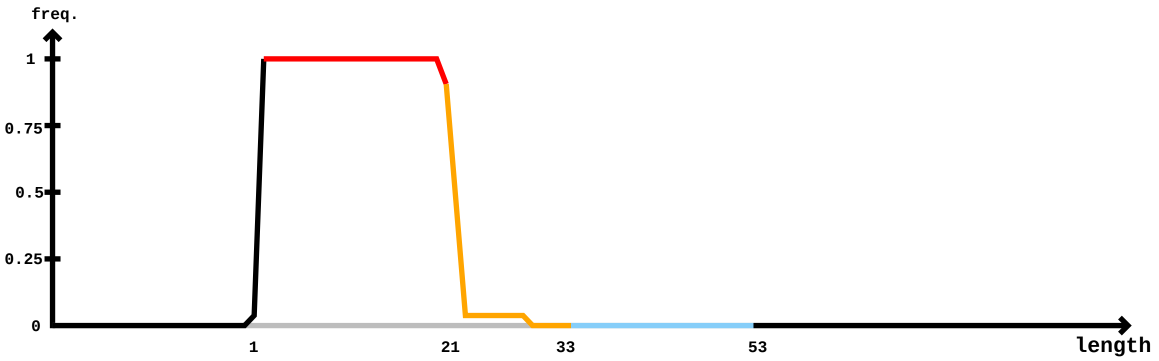

Mature Star

```
5'- cauggcggcaugguggcccguucgucggacgagcgugccuaggccaucggccgcgcgcuccuccgucgaacggcgccugccgugucgcgcgcaugcaguuaggcggcggg   -3'    exp
    ....((((((((((((((((((((.((((.((((((((...((((...)))))))))))).)))).))))))).))).)))))))))).(((.(((......))))))..    reads     mm      sample
    ...................guucgucggacgagcgugccu......................................................................    1         0       NN8

    ...................guucgucggacgagcgugcc.......................................................................    1         0       FF1
    ....................uucgucggacgagcgugcc.......................................................................    4         0       FF1
    ....................uucgucggaAgagcgugccu......................................................................    1         1       FF1
    ....................uucgucggacgagcgugccu......................................................................    42        0       FF1
    ....................uucgucggacgagcgugccA......................................................................    1         1       FF1
    ....................uucgucggacgagcgugccua.....................................................................    1         0       FF1
    ....................uucgucggacgagcgugccuaggccauc..............................................................    2         0       FF1
```
