# Supplementary material for: Identification of Novel miRNAs and miRNA Expression Profiling in Wheat Hybrid Necrosis
Source: PLoS One. 2015 Feb 23;10(2):e0117507. doi: 10.1371/journal.pone.0117507 (PMC4338152; doi:10.1371/journal.pone.0117507)
Supplement: S2 Fig — Red colored letter: mature miRNA sequence; yellow colored letter: loop sequence; blue colored letter: miRNA* sequence. (ZIP) [file pone.0117507.s002.zip › Figures s1/contig360034_5734.pdf]

| | | |
|---|---|---|
| Provisional ID | : | contig360034_5734 |
| Score total | : | 4.2 |
| Score for star read(s) | : | -1.3 |
| Score for read counts | : | 0 |
| Score for mfe | : | 0.9 |
| Score for randfold | : | 1.6 |
| Score for cons. seed | : | 3 |
| Total read count | : | 42 |
| Mature read count | : | 42 |
| Loop read count | : | 0 |
| Star read count | : | 0 |

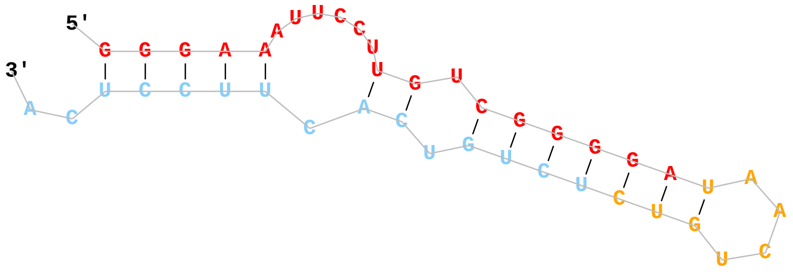


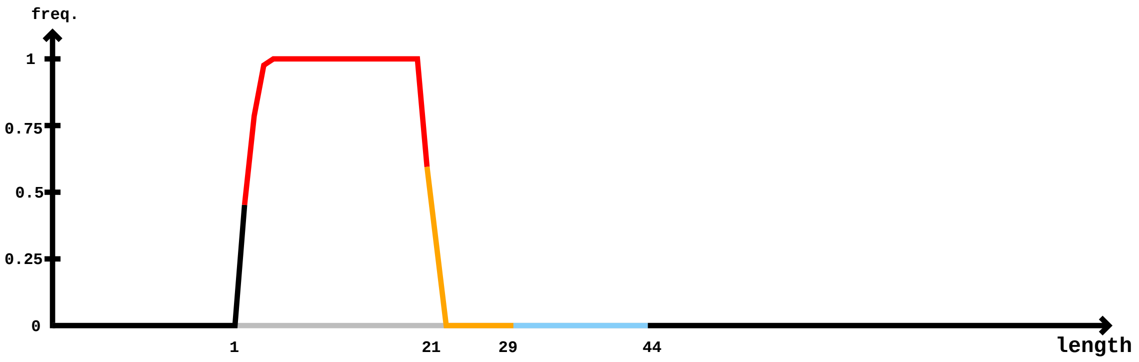


Mature Star

```
5'- uacauaauccauucuugggggaaauuccuugucggggauaacugucucugucacuuccucaucaggggguugugugcauucugcaauaaacucugccaacacccugcu   -3'   exp
    ................((((((......((.(((((((....))))))).)).))))))..(((((((((((.(((.....)))........)))))).)))))..   reads   mm   sample
    .................gCgaaauuccuugucgggg.........................................................................   5       1    NN8
    .................gCgaaauuccuugucgggga........................................................................   10      1    NN8
    .................gggaaauuccuugucgggUa........................................................................   1       1    NN8
    ..................Cgaaauuccuugucgggg.........................................................................   6       1    NN8
    ..................Cgaaauuccuugucgggga........................................................................   3       1    NN8
    ..................ggaaauuccuugucgggUa........................................................................   1       1    NN8
    ...................gaaauuccuugucgggCa........................................................................   3       1    NN8
    ...................gaaauuccuugucgggga........................................................................   1       0    NN8
    ...................gaaauuccuugucgggAa........................................................................   1       1    NN8
    ...................gaaauuccuugucggggaA.......................................................................   3       1    NN8
    ....................aaauuccuugucggggaA.......................................................................   1       1    NN8

    .................gCgaaauuccuugucgggg.........................................................................   3       1    FF1
    ..................ggaaauuccuugucgggU.........................................................................   1       1    FF1
    ..................Cgaaauuccuugucgggg.........................................................................   2       1    FF1
    ..................ggaaauuccuugucgggUa........................................................................   1       1    FF1
```
