# Supplementary material for: Identification of Novel miRNAs and miRNA Expression Profiling in Wheat Hybrid Necrosis
Source: PLoS One. 2015 Feb 23;10(2):e0117507. doi: 10.1371/journal.pone.0117507 (PMC4338152; doi:10.1371/journal.pone.0117507)
Supplement: S2 Fig — Red colored letter: mature miRNA sequence; yellow colored letter: loop sequence; blue colored letter: miRNA* sequence. (ZIP) [file pone.0117507.s002.zip › Figures s1/contig370756_5807.pdf]

Provisional ID	:	contig370756_5807
Score total	:	5.3
Score for star read(s)	:	-1.3
Score for read counts	:	0
Score for mfe	:	2
Score for randfold	:	1.6
Score for cons. seed	:	3
Total read count	:	19
Mature read count	:	19
Loop read count	:	0
Star read count	:	0

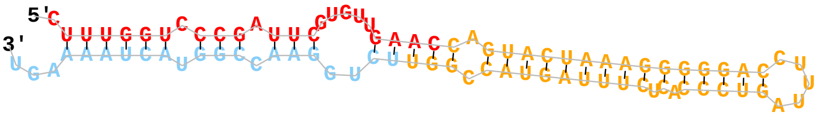

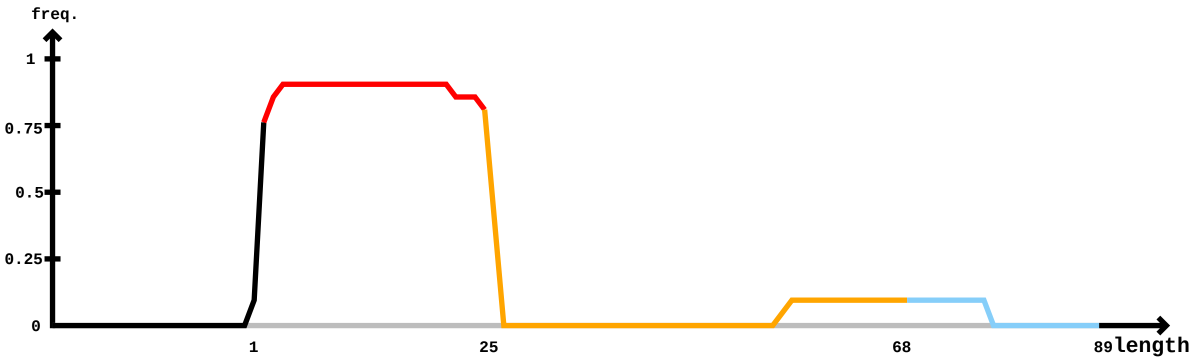

Mature Star

```
5'- ccucgugccacguggugggccuuuggucccgauucguguugaaccaguacuaaagggggaccuuuagucccacucuuuaguaccgguucuggaaccgguacuaaaagucguuuu   -3'    exp
    ((.((.....)).))..(((.((((((.(((.(((.....(((((.(((((((((((((((.....))))).).))))))))).)))))..))).))).)))))).))).....    reads    mm    sample
    ....................cuuggucccgauucguguugaac.......................................................................    4        0     NN8
    ....................cuuggucccgGuucguguugaac.......................................................................    6        1     NN8
    .........................................................................cuuuaguCccgguucuggaacc...................    1        1     NN8

    ...................ccuuggucccgauucguguu...........................................................................    1        0     FF1
    ...................ccuuggucccgauucguguugaa........................................................................    1        0     FF1
    ....................cuuggucccgGuucguguugaac.......................................................................    4        1     FF1
    .....................uuggucccgGuucguguugaac.......................................................................    1        1     FF1
    .....................uuggucccgauucguguugaacU......................................................................    1        1     FF1
    ......................uuAgucccgauucguguugaac......................................................................    1        1     FF1
    ..........................................................................uuuaguaccgguucuggaacc...................    1        0     FF1
```
