# Supplementary material for: Identification of Novel miRNAs and miRNA Expression Profiling in Wheat Hybrid Necrosis
Source: PLoS One. 2015 Feb 23;10(2):e0117507. doi: 10.1371/journal.pone.0117507 (PMC4338152; doi:10.1371/journal.pone.0117507)
Supplement: S2 Fig — Red colored letter: mature miRNA sequence; yellow colored letter: loop sequence; blue colored letter: miRNA* sequence. (ZIP) [file pone.0117507.s002.zip › Figures s1/contig370756_5808.pdf]

Provisional ID	:	contig370756_5808
Score total	:	5.6
Score for star read(s)	:	-1.3
Score for read counts	:	0
Score for mfe	:	2.3
Score for randfold	:	1.6
Score for cons. seed	:	3
Total read count	:	22
Mature read count	:	19
Loop read count	:	3
Star read count	:	0

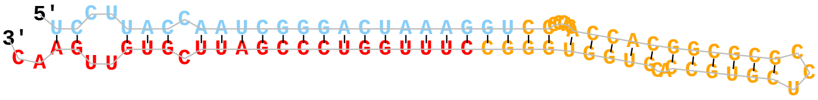

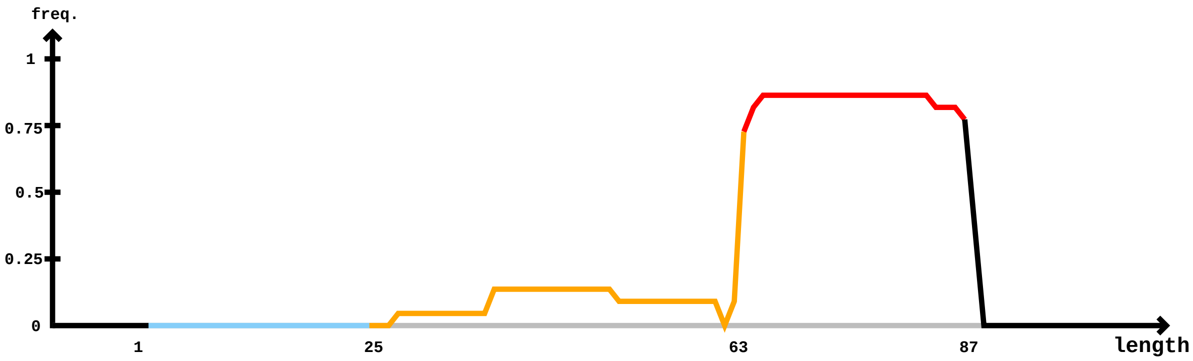

Star Mature

```
5'- aaaaaaaauccuuaccaaucgggacuaaagguccccc agaccacggcgcgccucgugccacguggugggccuuuggucccgauucguguugaaccaguacuaaagggggaccuu   -3'    exp
    ........((..(((.(((((((((((((((((((.....((((((((((...))))))..)))))))))))))))))))))))).)))..))..........((((....))))   reads     mm      sample
    ......................................................................cuuuggucccgauucguguugaac....................   4         0       NN8
    ......................................................................cuuuggucccgGuucguguugaac....................   6         1       NN8

    ..................................cccagaccacggcgcgccucgug.........................................................   1         0       FF1
    ............................................ggcgcgccucgugccacguggugg..............................................   2         0       FF1
    .....................................................................ccuuuggucccgauucguguu........................   1         0       FF1
    .....................................................................ccuuuggucccgauucguguugaa.....................   1         0       FF1
    ......................................................................cuuuggucccgGuucguguugaac....................   4         1       FF1
    .......................................................................uuuggucccgGuucguguugaac....................   1         1       FF1
    .......................................................................uuuggucccgauucguguugaacU...................   1         1       FF1
    ........................................................................uuAgucccgauucguguugaac....................   1         1       FF1
```
