# Supplementary material for: Identification of Novel miRNAs and miRNA Expression Profiling in Wheat Hybrid Necrosis
Source: PLoS One. 2015 Feb 23;10(2):e0117507. doi: 10.1371/journal.pone.0117507 (PMC4338152; doi:10.1371/journal.pone.0117507)
Supplement: S2 Fig — Red colored letter: mature miRNA sequence; yellow colored letter: loop sequence; blue colored letter: miRNA* sequence. (ZIP) [file pone.0117507.s002.zip › Figures s1/contig370930_5810.pdf]

Provisional ID	:	contig370930_5810
Score total	:	1
Score for star read(s)	:	-1.3
Score for read counts	:	0
Score for mfe	:	-2.2
Score for randfold	:	1.6
Score for cons. seed	:	3
Total read count	:	16
Mature read count	:	16
Loop read count	:	0
Star read count	:	0

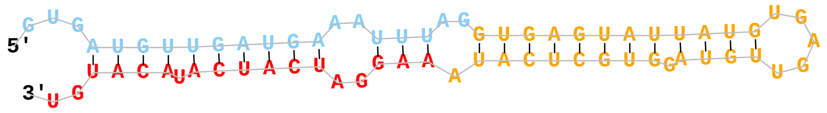

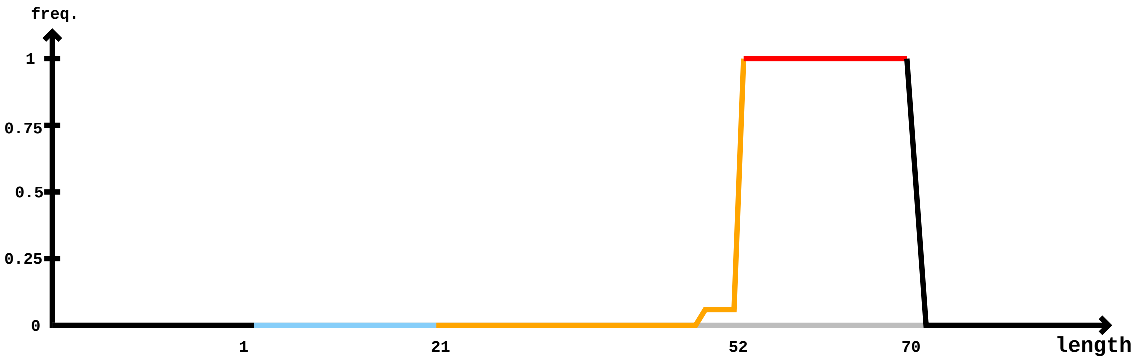

Star Mature

```
5'- cuguaaauauggacaaagagugauguugaugaaauuuaggugaguauuaugugaguuguaggugcucauaaaggaucaucauacauguguucuuagagacaagaacca   -3'    exp
    .(((...((.(((((....(((.(((.(((((..(((..((((((((((((.....)))).)))))))))).)))..)))))))))).))))).))...)))....... reads    mm     sample
    ..................................................................cauaaaggaucaucauacaugu.................... 1        0      NN8
    ......................................................................aaggaucaucauacaugu.................... 13       0      NN8

    ......................................................................aaggaucaucauacaugu.................... 3        0      FF1
```
