# Supplementary material for: Identification of Novel miRNAs and miRNA Expression Profiling in Wheat Hybrid Necrosis
Source: PLoS One. 2015 Feb 23;10(2):e0117507. doi: 10.1371/journal.pone.0117507 (PMC4338152; doi:10.1371/journal.pone.0117507)
Supplement: S2 Fig — Red colored letter: mature miRNA sequence; yellow colored letter: loop sequence; blue colored letter: miRNA* sequence. (ZIP) [file pone.0117507.s002.zip › Figures s1/contig379598_5894.pdf]

| | | |
|---|---|---|
| Provisional ID | : | contig379598_5894 |
| Score total | : | 24.8 |
| Score for star read(s) | : | 3.9 |
| Score for read counts | : | 17.5 |
| Score for mfe | : | 2.4 |
| Score for randfold | : | 1.6 |
| Score for cons. seed | : | -0.6 |
| Total read count | : | 46 |
| Mature read count | : | 33 |
| Loop read count | : | 0 |
| Star read count | : | 13 |

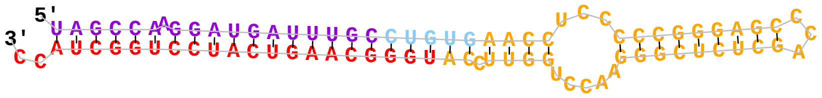


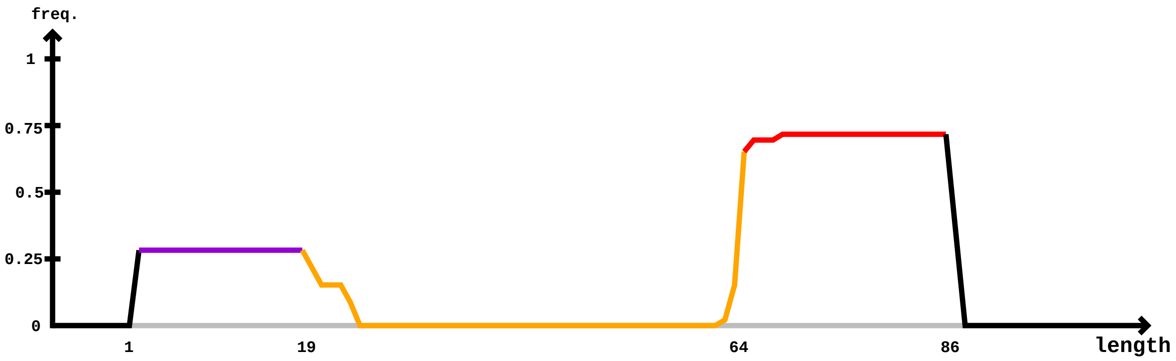


**Star** **Mature**

```
5'- ucuucgauagccaaggaugauuugccugugaaccucccccgggagcccagcucucgggaaccugguuccaugggcaagucauccuggcuacccaaguaccucuuaccauacc  -3'   obs
    ucuucgauagccaaggaugauuugccugugaaccucccccgggagcccagcucucgggaaccugguuccaugggcaagucauccuggcuacccaaguaccucuuaccauacc        exp
    .(((.(.((((((.((((((((((((((((((((...(((((((((...))))))))).....)))).)))))))))))))))))))))).).)))................   reads    mm    sample
    .......uagccaaggaugauuugccugu...................................................................................   1        0     NN8
    ..........................................................................caagucauccuggcuacc....................   1        0     NN8

    .......uagccaaggaugauuugU.......................................................................................   1        1     FF1
    .......uagccaaggaugauuugc.......................................................................................   5        0     FF1
    .......uagccaaggaugauuugccugu...................................................................................   2        0     FF1
    .......uagccaaggaugauuugccugGg.................................................................................   1        1     FF1
    .......uagccaaggaugauuugccugug.................................................................................   2        0     FF1
    .......uagccaGggaugauuugccugug.................................................................................   1        1     FF1
    ....................................................................caugggcaagucaCccuggcuacc....................   1        1     FF1
    .....................................................................augggcaagucaCccuggcuacc....................   5        1     FF1
    .....................................................................augggcaagucauccuggcuacc....................   1        0     FF1
    ......................................................................ugggcaagGcauccuggcuacc....................   1        1     FF1
    ......................................................................ugggcaagucaCccuggcuacc....................   6        1     FF1
    ......................................................................ugggcaagucauccuggcuacc....................   16       0     FF1
    .......................................................................gggcaagucauccuggcuacc....................   1        0     FF1
    .......................................................................gggcaagucaCccuggcuacc....................   1        1     FF1
```
