# Supplementary material for: Identification of Novel miRNAs and miRNA Expression Profiling in Wheat Hybrid Necrosis
Source: PLoS One. 2015 Feb 23;10(2):e0117507. doi: 10.1371/journal.pone.0117507 (PMC4338152; doi:10.1371/journal.pone.0117507)
Supplement: S2 Fig — Red colored letter: mature miRNA sequence; yellow colored letter: loop sequence; blue colored letter: miRNA* sequence. (ZIP) [file pone.0117507.s002.zip › Figures s1/contig3827810_16742.pdf]

Provisional ID : contig3827810_16742
Score total : 3.8
Score for star read(s) : -1.3
Score for read counts : -1.8
Score for mfe : 2.4
Score for randfold : 1.6
Score for cons. seed : 3
Total read count : 8
Mature read count : 8
Loop read count : 0
Star read count : 0

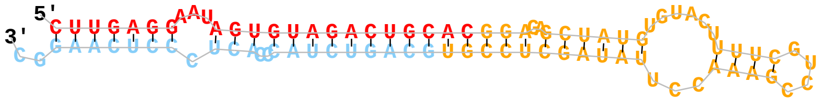

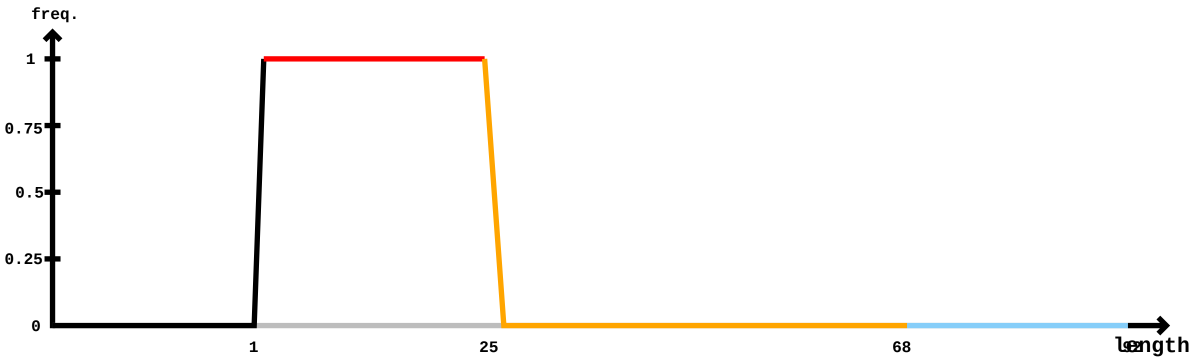


```
                     Mature                                                       Star
5'- cacguugcuuaucuucaggccuugaggaauaguguagacugcacggagagcuauguguacuuuucguccgaaaccuuauagcuccgugcagucuacccacucccucaaggccug   -3'    exp
    ...............((((((((((((...(((((((((((((((((..((((((......((((....))))...))))))))))))))))))))..))).))))))))))))    reads      mm         sample
    ....................cuCgaggaauaguguagacugcac......................................................................    1          1          NN8
    ....................cuugaCgaauaguguagacugcac......................................................................    1          1          NN8
    ....................cuugaggaauaguguagacugcac......................................................................    5          0          NN8

    ....................cuugaggaauaguguagacugcac......................................................................    1          0          FF1
```
