# Supplementary material for: Identification of Novel miRNAs and miRNA Expression Profiling in Wheat Hybrid Necrosis
Source: PLoS One. 2015 Feb 23;10(2):e0117507. doi: 10.1371/journal.pone.0117507 (PMC4338152; doi:10.1371/journal.pone.0117507)
Supplement: S2 Fig — Red colored letter: mature miRNA sequence; yellow colored letter: loop sequence; blue colored letter: miRNA* sequence. (ZIP) [file pone.0117507.s002.zip › Figures s1/contig38447_1368.pdf]

Provisional ID	:	contig38447_1368
Score total	:	9279
Score for star read(s)	:	3.9
Score for read counts	:	9267.7
Score for mfe	:	2.7
Score for randfold	:	1.6
Score for cons. seed	:	3
Total read count	:	18190
Mature read count	:	15464
Loop read count	:	0
Star read count	:	2726

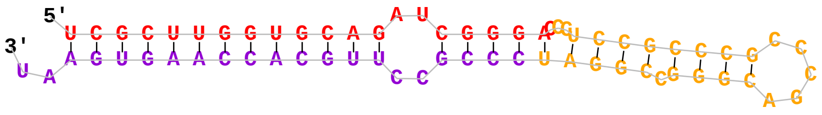

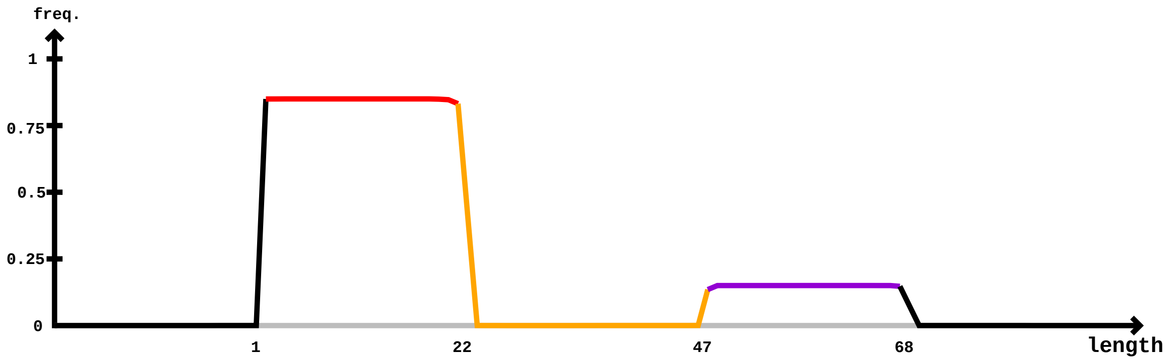

Mature Star

```
5'- ccgucgccgccgcucgggcucgcuuggugcagaucgggacccuccgcccgcccgacgggccggaucccgccuugcaccaagugaaucggagccggcgcagcgaccccgcc  -3'   obs
    ccgucgccgccgcucgggcucgcuuggugcagaucgggacccuccgcccgcccgacgggccggaucccgccuugcaccaagugaaucggagccggcgcagcgaccccgcc         exp
    ..(((((((((.(((.(..(((((((((((((..(((((...((((((((.....)))).)))))))))..)))))))))))))..).))).)))))..)))))......   reads   mm   sample
    ...................ucgcuuggugcagaucgg.........................................................................   2       0    NN8
    ...................ucgcuuggugcagaucggg........................................................................   6       0    NN8
    ...................ucgcuuggugcagaucggga.......................................................................   80      0    NN8
    ...................Ccgcuuggugcagaucggga.......................................................................   1       1    NN8
    ...................ucgcuuggGgcagaucggga.......................................................................   1       1    NN8
    ...................ucgcGuggugcagaucggga.......................................................................   1       1    NN8
    ...................ucgcuuggugcagauUgggac......................................................................   2       1    NN8
    ...................ucgcuCggugcagaucgggac......................................................................   1       1    NN8
    ...................ucAcuuggugcagaucgggac......................................................................   2       1    NN8
    ...................ucgcuuggugcagaCcgggac......................................................................   2       1    NN8
    ...................Gcgcuuggugcagaucgggac......................................................................   2       1    NN8
    ...................ucgcuuggugcGgaucgggac......................................................................   2       1    NN8
    ...................uGgcuuggugcagaucgggac......................................................................   4       1    NN8
    ...................ucgcuuggugcagaucgUgac......................................................................   1       1    NN8
    ...................Acgcuuggugcagaucgggac......................................................................   3       1    NN8
    ...................ucgcuuggugcagaucggUac......................................................................   1       1    NN8
    ...................ucgcuuggugcagaucgggaA......................................................................   1       1    NN8
    ...................uAgcuuggugcagaucgggac......................................................................   1       1    NN8
    ...................ucgcuuggugcagauGgggac......................................................................   1       1    NN8
    ...................ucgcuuggugcagaGcgggac......................................................................   1       1    NN8
    ...................ucgcuuggugcagaucgAgac......................................................................   1       1    NN8
    ...................ucgcuuggugcUgaucgggac......................................................................   2       1    NN8
    ...................ucgcuuggugcagCucgggac......................................................................   1       1    NN8
    ...................ucgcuuggAgcagaucgggac......................................................................   3       1    NN8
    ...................ucgcuuggugcagaucgggac......................................................................   1931    0    NN8
    ...................ucgcuuAgugcagaucgggac......................................................................   2       1    NN8
    ...................ucgcuuggugcaUaucgggac......................................................................   2       1    NN8
    ...................ucgcuugguUcagaucgggac......................................................................   1       1    NN8
    ...................ucgcuuggugcagaucAggac......................................................................   3       1    NN8
    ...................ucgcuuggugcCgaucgggac......................................................................   2       1    NN8
    ...................ucgcuuggugGagaucgggac......................................................................   2       1    NN8
    ...................ucgAuuggugcagaucgggac......................................................................   1       1    NN8
    ...................ucgcuuggugUagaucgggac......................................................................   1       1    NN8
```

Mature Star

```
ccgucgccgccgccucgggcucgcuuggugcagaucgggacccuccgcccgcccgacgggccggaucccgccuugcaccaagugaaucggagccggcgcagcgaccccgcc
....................ucgcuuggGgcagaucgggac......................................................................  5    1  NN8
....................ucgcuuggugcaAaucgggac......................................................................  2    1  NN8
....................ucgcuuggugcagaucgggaU......................................................................  20   1  NN8
....................ucgcCuggugcagaucgggac......................................................................  1    1  NN8
....................ucgcuuggugcagauAgggac......................................................................  2    1  NN8
....................ucCcuuggugcagaucgggac......................................................................  1    1  NN8
....................ucgcuuggugcagaucggAac......................................................................  1    1  NN8
....................uUgcuuggugcagaucgggac......................................................................  1    1  NN8
....................ucgcuGggugcagaucgggac......................................................................  1    1  NN8
....................ucgcuuUgugcagaucgggac......................................................................  1    1  NN8
....................ucgcuuggugAagaucgggac......................................................................  4    1  NN8
....................ucgcuuggugcagGucgggac......................................................................  1    1  NN8
....................ucgcGuggugcagaucgggac......................................................................  1    1  NN8
....................ucgcuuggugcagaucgggacc.....................................................................  33   0  NN8
....................ucgcuuggugcagaucgggacU.....................................................................  16   1  NN8
....................ucgcuuggugcagaucgggacA.....................................................................  1    1  NN8
....................ucgcuuggugcagaucgggacccuc..................................................................  1    0  NN8
.....................cgcuuggugcagaucgggac......................................................................  2    0  NN8
......................gcuuggugcagaucgggac......................................................................  1    0  NN8
.......................................................acgggccggaucccgccuugcaccaagugaau........................  1    0  NN8
.............................................................cggaucccgccuugcaccaagugaU.........................  1    1  NN8
.................................................................Ccccgccuugcaccaagugaau........................  1    1  NN8
..................................................................cccgccuugcaccaagugaa.........................  9    0  NN8
..................................................................cccgccuugcaccaGgugaau........................  1    1  NN8
..................................................................cccgccuugcaccaagugaGu........................  1    1  NN8
..................................................................cccgccuugcaccUagugaau........................  1    1  NN8
..................................................................cccgccuugcaccaagugaaA........................  3    1  NN8
..................................................................cccgccuugcaccaagugaau........................  342  0  NN8
..................................................................cccgcAuugcaccaagugaau........................  1    1  NN8
..................................................................cccgccuugcaccaagugaCu........................  1    1  NN8
..................................................................cccgccuugcaccaaAugaau........................  2    1  NN8
..................................................................cccgcGuugcaccaagugaau........................  1    1  NN8
..................................................................cccgccuugcaccaagugaauU.......................  1    1  NN8
...................................................................ccgccuugcaccaagugaa.........................  5    0  NN8
...................................................................ccgccuAgcaccaagugaau........................  1    1  NN8
...................................................................Acgccuugcaccaagugaau........................  2    1  NN8
...................................................................ccgccuugcaccaagugaau........................  108  0  NN8
...................................................................ccgcGuugcaccaagugaau........................  2    1  NN8
...................................................................ccgccuugcaccaagugaUu........................  2    1  NN8
...................................................................ccgccuugcaccaagugaauU.......................  1    1  NN8

...............cgggcucgcuuggugcagauc...........................................................................  1    0  FF1
....................ucgcuuggAgcagaucgg.........................................................................  1    1  FF1
....................ucgcuuggugcagaucgU.........................................................................  1    1  FF1
....................ucgcuuggugcagaucgg.........................................................................  11   0  FF1
....................ucgcuuggugcaUaucggg........................................................................  1    1  FF1
....................ucgcuuggugcaAaucggg........................................................................  1    1  FF1
....................ucgcuuggugcagaucggg........................................................................  30   0  FF1
....................ucgcuuggugcagaucgggG.......................................................................  1    1  FF1
....................ucAcuuggugcagaucggga.......................................................................  1    1  FF1
....................Gcgcuuggugcagaucggga.......................................................................  3    1  FF1
....................ucgcuuggugcagaCcggga.......................................................................  1    1  FF1
....................ucgcuuggugcagaucggga.......................................................................  181  0  FF1
....................ucgcuuggugcagaucgggU.......................................................................  1    1  FF1
....................uAgcuuggugcagaucggga.......................................................................  1    1  FF1
....................ucgcuuggugcagaucCggac......................................................................  4    1  FF1
....................uGgcuuggugcagaucgggac......................................................................  21   1  FF1
....................Acgcuuggugcagaucgggac......................................................................  19   1  FF1
....................ucgcuuggugcagauUgggac......................................................................  11   1  FF1
....................ucgcuuggugcagCucgggac......................................................................  1    1  FF1
....................ucgcuugguAcagaucgggac......................................................................  2    1  FF1
....................ucgcuuUgugcagaucgggac......................................................................  4    1  FF1
....................ucgUuuggugcagaucgggac......................................................................  2    1  FF1
....................ucgcuuggugcagaucggAac......................................................................  6    1  FF1
....................ucgcuuggugcagaucgggCc......................................................................  1    1  FF1
....................ucgcuuggAgcagaucgggac......................................................................  20   1  FF1
....................ucgcuuggugcagauAgggac......................................................................  18   1  FF1
....................uAgcuuggugcagaucgggac......................................................................  8    1  FF1
....................ucgcuuggugcagaucgAgac......................................................................  9    1  FF1
....................ucgcuuggugcagGucgggac......................................................................  4    1  FF1
```

```
                    Mature                                      Star
ccgucgccgccgccucgggcucgcuuggugcagaucgggacccuccgcccgcccgacgggccggaucccgccuugcaccaagugaaucggagccggcgcagcgaccccgcc
....................ucgcuuggugGagaucgggac......................................................................   7       1   FF1
....................ucgcuuggugcaUaucgggac......................................................................   11      1   FF1
....................ucgcuuggugcagaucUggac......................................................................   5       1   FF1
....................ucgcCuggugcagaucgggac......................................................................   1       1   FF1
....................ucgcuuggugcagaCcgggac......................................................................   3       1   FF1
....................ucgAuuggugcagaucgggac......................................................................   5       1   FF1
....................ucgcuuggGgcagaucgggac......................................................................   47      1   FF1
....................ucgcuuggugcagaucgggaU......................................................................   161     1   FF1
....................ucgcuugCugcagaucgggac......................................................................   1       1   FF1
....................ucgcuuggugcaAaucgggac......................................................................   9       1   FF1
....................ucgcuuggugcagaucgggaA......................................................................   3       1   FF1
....................ucgcuuggugcagaucgCgac......................................................................   9       1   FF1
....................ucUcuuggugcagaucgggac......................................................................   2       1   FF1
....................ucgcuuggugcagaucggUac......................................................................   4       1   FF1
....................ucgcuuggugcaCaucgggac......................................................................   26      1   FF1
....................ucgcuuggugcagaucgUgac......................................................................   4       1   FF1
....................ucgcuugguCcagaucgggac......................................................................   2       1   FF1
....................ucgcuuggugcagaGcgggac......................................................................   1       1   FF1
....................Ccgcuuggugcagaucgggac......................................................................   8       1   FF1
....................ucgcuuggugcagaucgggac......................................................................   12265   0   FF1
....................ucAcuuggugcagaucgggac......................................................................   12      1   FF1
....................ucgcuuggugUagaucgggac......................................................................   3       1   FF1
....................ucgcuuggugcagaucggCac......................................................................   3       1   FF1
....................ucgcuuggugcagUucgggac......................................................................   2       1   FF1
....................ucgcAuggugcagaucgggac......................................................................   3       1   FF1
....................ucgcuuAgugcagaucgggac......................................................................   4       1   FF1
....................ucCcuuggugcagaucgggac......................................................................   6       1   FF1
....................Gcgcuuggugcagaucgggac......................................................................   42      1   FF1
....................ucgGuuggugcagaucgggac......................................................................   11      1   FF1
....................ucgcuugAugcagaucgggac......................................................................   7       1   FF1
....................ucgcuuggCgcagaucgggac......................................................................   14      1   FF1
....................ucgcuAggugcagaucgggac......................................................................   2       1   FF1
....................ucgcuuggugcagaucgggGc......................................................................   7       1   FF1
....................uUgcuuggugcagaucgggac......................................................................   6       1   FF1
....................ucgcuuCgugcagaucgggac......................................................................   4       1   FF1
....................ucgcuuggugAagaucgggac......................................................................   8       1   FF1
....................ucgcuuggugcGgaucgggac......................................................................   11      1   FF1
....................ucgcuuggugcagauGgggac......................................................................   28      1   FF1
....................ucgcuuggugcagaucgggacA.....................................................................   9       1   FF1
....................ucgcuuggugcagaucgggacc.....................................................................   74      0   FF1
....................ucgcuuggugcagauGgggacc.....................................................................   1       1   FF1
....................uUgcuuggugcagaucgggacc.....................................................................   1       1   FF1
....................ucgcuuggCgcagaucgggacc.....................................................................   1       1   FF1
....................ucgcuuggugcaAaucgggacc.....................................................................   1       1   FF1
....................Acgcuuggugcagaucgggacc.....................................................................   2       1   FF1
....................ucgcuuggugcagaucgggacU.....................................................................   97      1   FF1
....................ucgcuuggugcagaucgggaccU....................................................................   1       1   FF1
....................ucgcuuggugcagaucgggaccc....................................................................   1       0   FF1
......................gcuuggugcagaucgggac......................................................................   2       0   FF1
.......................cuuggugcagaucgggacccu...................................................................   1       0   FF1
.....................................................cgacgggccggaucccgccuugcaccaagGga..........................   1       1   FF1
.......................................................acgggccggaucccgccuugcaccaagug...........................   1       0   FF1
.................................................................ucccgccuugcaccaagugaa.........................   1       0   FF1
.................................................................Ccccgccuugcaccaagugaau........................   1       1   FF1
.................................................................ucccgccuugcaccaagugaau........................   1       0   FF1
..................................................................cccgccuugcaccaagugaU.........................   1       1   FF1
..................................................................cccgccuugcaccaagugaa.........................   32      0   FF1
..................................................................Gccgccuugcaccaagugaau........................   4       1   FF1
..................................................................cccgccuugcacUaagugaau........................   4       1   FF1
..................................................................cccgccuugcaccaaAugaau........................   3       1   FF1
..................................................................ccUgccuugcaccaagugaau........................   1       1   FF1
..................................................................cAcgccuugcaccaagugaau........................   4       1   FF1
..................................................................cccgccuugcaccaagugaGu........................   1       1   FF1
..................................................................cccgccuugcaccaagGgaau........................   5       1   FF1
..................................................................cccgccuugcaccaaguCaau........................   1       1   FF1
..................................................................cccgccuugcacGaagugaau........................   4       1   FF1
..................................................................cccgccuugcaccaagugaau........................   1973    0   FF1
..................................................................cccgccuugcaccaagugUau........................   2       1   FF1
..................................................................cccgccuugcaccaagugaaA.......................   1       1   FF1
..................................................................cccgccuugcaccaagAgaau........................   2       1   FF1
```

```
                    Mature                                       Star
ccgucgccgccgccucgggcucgcuuggugcagaucgggacccuccgcccgcccgacgggccggaucccgccuugcaccaagugaaucggagccggcgcagcgaccccgcc
..................................................................cccgccuugcaccaagCgaau........................   5     1    FF1
..................................................................cUcgccuugcaccaagugaau........................   2     1    FF1
..................................................................cccUccuugcaccaagugaau........................   1     1    FF1
..................................................................ccGgccuugcaccaagugaau........................   2     1    FF1
..................................................................cccgccuugcaccaaguAaau........................   3     1    FF1
..................................................................cccgccCugcaccaagugaau........................   1     1    FF1
..................................................................Accgccuugcaccaagugaau........................   4     1    FF1
..................................................................cccCccuugcaccaagugaau........................   1     1    FF1
..................................................................cccgccuugcaccaaguUaau........................   2     1    FF1
..................................................................cGcgccuugcaccaagugaau........................   4     1    FF1
..................................................................cccgccuugcGccaagugaau........................   1     1    FF1
..................................................................cccgccuugcacAaagugaau........................   5     1    FF1
..................................................................Uccgccuugcaccaagugaau........................   4     1    FF1
..................................................................cccgccuugcaccaagugaauU.......................   11    1    FF1
..................................................................cccgccuugcaccaagugaauA.......................   2     1    FF1
...................................................................ccgccuugcaccaagugaa.........................   2     0    FF1
...................................................................ccgccuugcaccaagugaau........................   142   0    FF1
...................................................................ccgccuugcaGcaagugaau........................   1     1    FF1
...................................................................cGgccuugcaccaagugaau........................   1     1    FF1
...................................................................Ucgccuugcaccaagugaau........................   1     1    FF1
...................................................................cUgccuugcaccaagugaau........................   2     1    FF1
...................................................................ccgccuugcaccaaguAaau........................   1     1    FF1
...................................................................ccgccuugcaccaagugaauA.......................   1     1    FF1
.......................................................................cuugcaccaagugaaucggag...................   1     0    FF1
............................................................................accaagugaaucggagccggcgca...........   1     0    FF1
```
