# Supplementary material for: Identification of Novel miRNAs and miRNA Expression Profiling in Wheat Hybrid Necrosis
Source: PLoS One. 2015 Feb 23;10(2):e0117507. doi: 10.1371/journal.pone.0117507 (PMC4338152; doi:10.1371/journal.pone.0117507)
Supplement: S2 Fig — Red colored letter: mature miRNA sequence; yellow colored letter: loop sequence; blue colored letter: miRNA* sequence. (ZIP) [file pone.0117507.s002.zip › Figures s1/contig399813_6090.pdf]

Provisional ID	:	contig399813_6090
Score total	:	80.9
Score for star read(s)	:	3.9
Score for read counts	:	70
Score for mfe	:	2.4
Score for randfold	:	1.6
Score for cons. seed	:	3
Total read count	:	149
Mature read count	:	146
Loop read count	:	0
Star read count	:	3

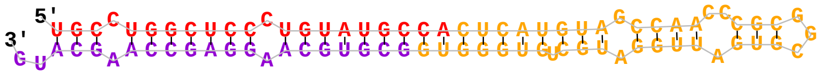

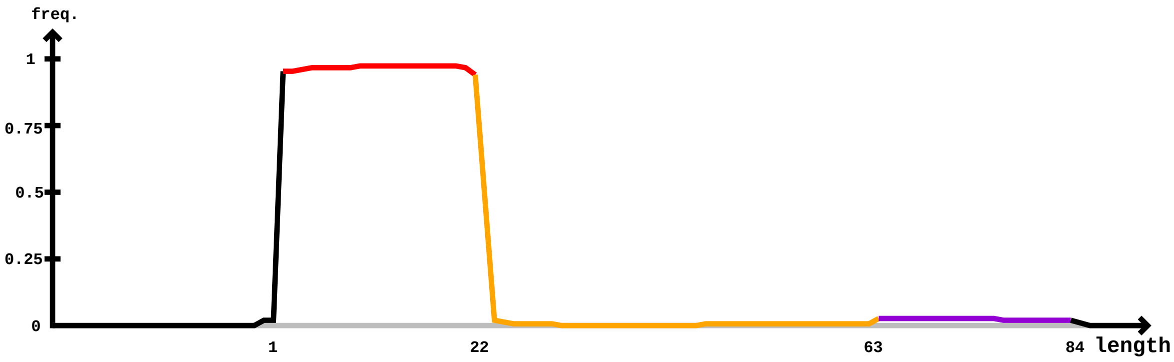

Mature Star

```
5'- ucgccgugcgugcgugcuugugugccuggcucccuguaugccacucauguagccaacccgcggcgugauuggaugcugugggugGcgugcaaggagccaagcaugcguacau  -3'  obs
    ucgccgugcgugcgugcuugugugccuggcucccuguaugccacucauguagccaacccgcggcgugauuggaugcuguggguggcgugcaaggagccaagcaugcguacau        exp
    ..((((....))).))..((((((.(((((((.((((((((((((((((.((((..(((...))).)))).))).))))))))))))))).))))))).))))))......   reads   mm   sample
    ......................ugccuggcucccuguaugcca.....................................................................   10      0    NN8
    ..............................ucccuguaugccacucaugua.............................................................   1       0    NN8
    ..................................................................gauuggaugcuguggguggcgugcaaggagU...............   1       1    NN8

    ....................ugugccuggcucccuguaugcc......................................................................   3       0    FF1
    ......................ugccuggcucccuguauUc.......................................................................   1       1    FF1
    ......................ugccuggcucccuguaugcc......................................................................   1       0    FF1
    ......................uAccuggcucccuguaugcca.....................................................................   1       1    FF1
    ......................ugccuggcucccuguGugcca.....................................................................   1       1    FF1
    ......................ugccuggcucccuguaugcca.....................................................................   124     0    FF1
    ......................ugccuggcucccuguCugcca.....................................................................   1       1    FF1
    ......................ugccuggAucccuguaugcca.....................................................................   1       1    FF1
    ......................ugccuggcucccuguaugccac....................................................................   1       0    FF1
    ........................ccuggcucccuguaugccacu...................................................................   1       0    FF1
    .........................cuggcucccuguaugccacuc..................................................................   1       0    FF1
    ....................................................................................gcgugcaaggagccaagcaug.......   3       0    FF1
```
