# Supplementary material for: Identification of Novel miRNAs and miRNA Expression Profiling in Wheat Hybrid Necrosis
Source: PLoS One. 2015 Feb 23;10(2):e0117507. doi: 10.1371/journal.pone.0117507 (PMC4338152; doi:10.1371/journal.pone.0117507)
Supplement: S2 Fig — Red colored letter: mature miRNA sequence; yellow colored letter: loop sequence; blue colored letter: miRNA* sequence. (ZIP) [file pone.0117507.s002.zip › Figures s1/contig4115927_17337.pdf]

Provisional ID	:	contig4115927_17337
Score total	:	0.1
Score for star read(s)	:	-1.3
Score for read counts	:	-4.4
Score for mfe	:	1.3
Score for randfold	:	1.6
Score for cons. seed	:	3
Total read count	:	3
Mature read count	:	3
Loop read count	:	0
Star read count	:	0

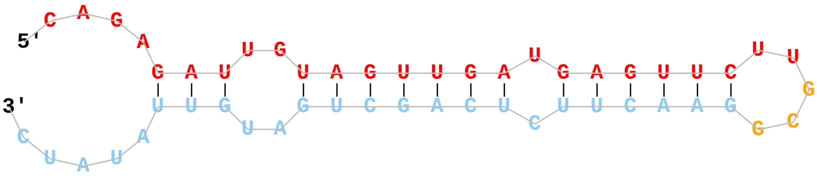

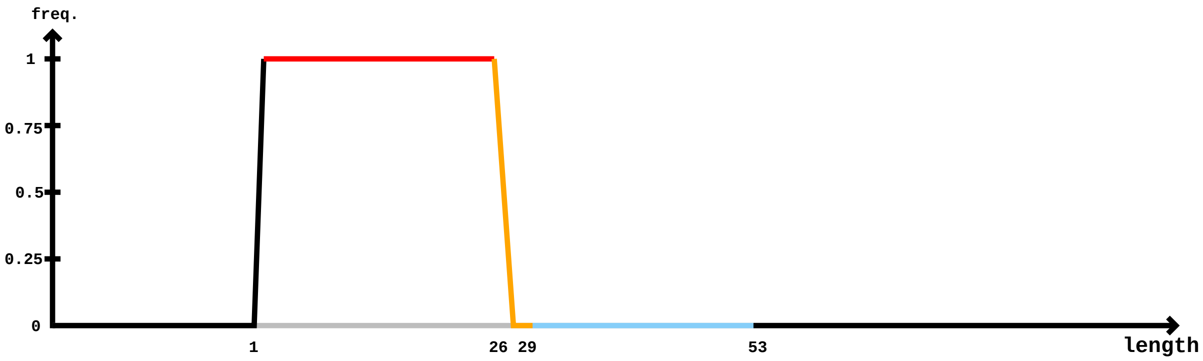

Mature Star

```
5'- ugagucugaaggaggcagggcagagauuguaguugaugaguucuugcggaacuucucagcugauguuauaucaagagcuucuuuuggaagcaguuuugucgaaggcaaagagaua   -3'    exp
    ((..((..((((((((..(..(..(((..(((((((.((((((.....)))))).)))))))..))).)..)....))))))))..))..)).((((((.....)))))).....      reads       mm          sample
    ....................cagagauuguaguugaugaguucuu......................................................................      3           0           NN8
```
