# Supplementary material for: Identification of Novel miRNAs and miRNA Expression Profiling in Wheat Hybrid Necrosis
Source: PLoS One. 2015 Feb 23;10(2):e0117507. doi: 10.1371/journal.pone.0117507 (PMC4338152; doi:10.1371/journal.pone.0117507)
Supplement: S2 Fig — Red colored letter: mature miRNA sequence; yellow colored letter: loop sequence; blue colored letter: miRNA* sequence. (ZIP) [file pone.0117507.s002.zip › Figures s1/contig4199570_17569.pdf]

Provisional ID	:	contig4199570_17569
Score total	:	0.1
Score for star read(s)	:	-1.3
Score for read counts	:	0
Score for mfe	:	0.6
Score for randfold	:	-2.2
Score for cons. seed	:	3
Total read count	:	30
Mature read count	:	30
Loop read count	:	0
Star read count	:	0

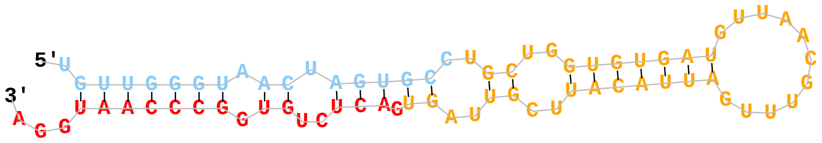

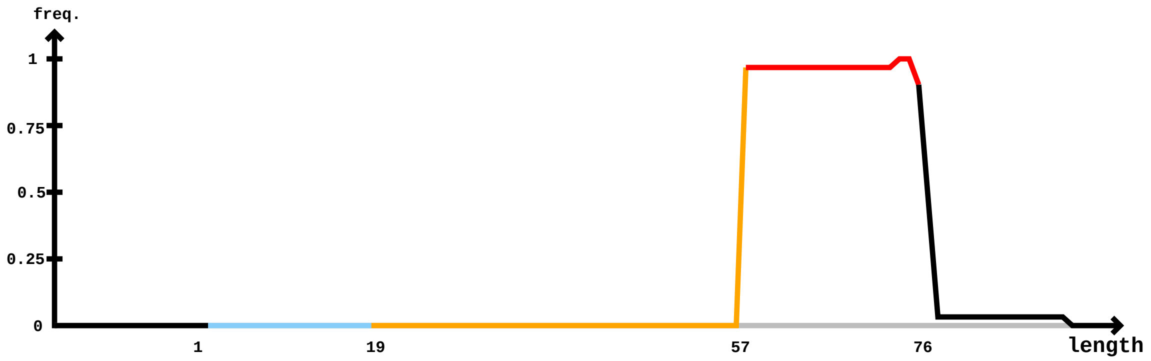


```
                 Star                                        Mature
5'- ugugaugucaggccuguuggguaacuagugccugcuggugugauguuaacguuugauuacauucguuagugacucuguggcccaauggauaaggcgcuggucuacgaaa   -3'    exp
    .(((...(((((((((((((((.((.(((((..((..(((((((...........)))))))..))..)).)))..)).))))))).....)))).)))).)))....    reads      mm         sample
    ......................................................................gacucuguCgcccaaugg.....................    1          1          NN8
    ......................................................................................ggauaaggcgcuggucuG.....    1          1          NN8

    ......................................................................gacucuguUgcccaaugg.....................    1          1          FF1
    ......................................................................gacucuguggcccaaugg.....................    1          0          FF1
    ......................................................................gacAcuguggcccaaugga....................    1          1          FF1
    ......................................................................gacucuguUgcccaaugga....................    7          1          FF1
    ......................................................................gacucuguAgcccaaugga....................    2          1          FF1
    ......................................................................gacucuguCgcccaaugga....................    4          1          FF1
    ......................................................................gacucuguggcccaaugga....................    13         0          FF1
```
