# Supplementary material for: Identification of Novel miRNAs and miRNA Expression Profiling in Wheat Hybrid Necrosis
Source: PLoS One. 2015 Feb 23;10(2):e0117507. doi: 10.1371/journal.pone.0117507 (PMC4338152; doi:10.1371/journal.pone.0117507)
Supplement: S2 Fig — Red colored letter: mature miRNA sequence; yellow colored letter: loop sequence; blue colored letter: miRNA* sequence. (ZIP) [file pone.0117507.s002.zip › Figures s1/contig4238746_17655.pdf]

Provisional ID	:	contig4238746_17655
Score total	:	15
Score for star read(s)	:	3.9
Score for read counts	:	7.8
Score for mfe	:	2.3
Score for randfold	:	1.6
Score for cons. seed	:	-0.6
Total read count	:	27
Mature read count	:	22
Loop read count	:	0
Star read count	:	5

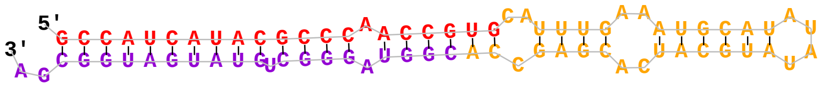

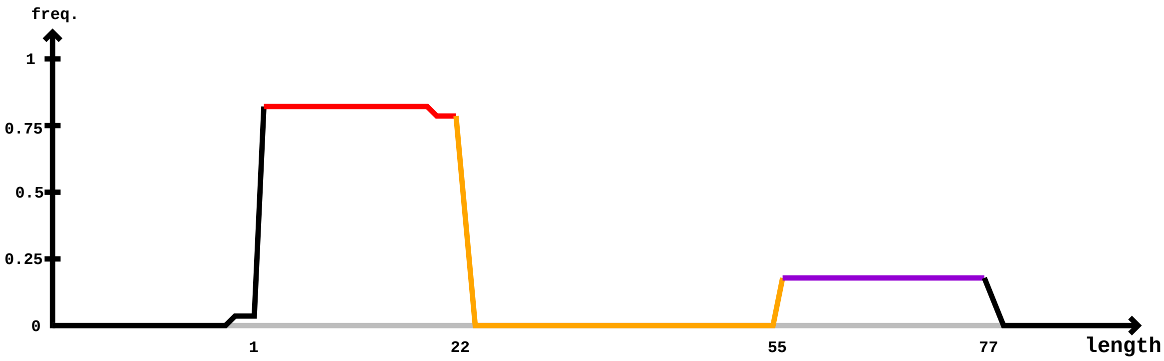


```
                         Mature                                                Star
5'- aauauaaaucauucagauucgccaucauacgcccaaccgugcauuugaaaugcauauauaugcaucacgagccacgguagggcuguaugauggcgauaccgauugguuugu   -3'    obs
    aauauaaaucauucagauucgccaucauacgcccaaccgugcauuugaaaugcauauauaugcaucacgagccacgguagggcuguaugauggcgauaccgauugguuugu          exp
    ...((((((((.((....((((((((((((((((.((((((..((((..((((((....))))))..)))).)))))).)))).))))))))))))....)).))))))))    reads      mm        sample
    ....................gccaucauacgcccaaccgug......................................................................    14         0         NN8

    .................uucgccaucauacgcccaacc.........................................................................    1          0         FF1
    ....................gccaucauacgcccaaccgug......................................................................    5          0         FF1
    ....................gccaucauacgcccaaccguU......................................................................    1          1         FF1
    ....................gccaucauacgcccaaccgugU.....................................................................    2          1         FF1
    ..........................................................................cgguagggcuguaugauggcga...............    4          0         FF1
    ..........................................................................cgguagggcuguaugauggUga...............    1          1         FF1
```
