# Supplementary material for: Identification of Novel miRNAs and miRNA Expression Profiling in Wheat Hybrid Necrosis
Source: PLoS One. 2015 Feb 23;10(2):e0117507. doi: 10.1371/journal.pone.0117507 (PMC4338152; doi:10.1371/journal.pone.0117507)
Supplement: S2 Fig — Red colored letter: mature miRNA sequence; yellow colored letter: loop sequence; blue colored letter: miRNA* sequence. (ZIP) [file pone.0117507.s002.zip › Figures s1/contig443571_6462.pdf]

Provisional ID	:	contig443571_6462
Score total	:	66.8
Score for star read(s)	:	3.9
Score for read counts	:	62.8
Score for mfe	:	-0.8
Score for randfold	:	1.6
Score for cons. seed	:	-0.6
Total read count	:	135
Mature read count	:	123
Loop read count	:	3
Star read count	:	9

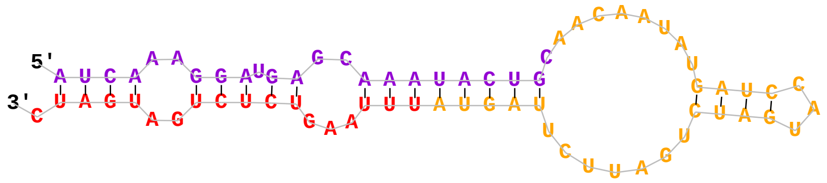

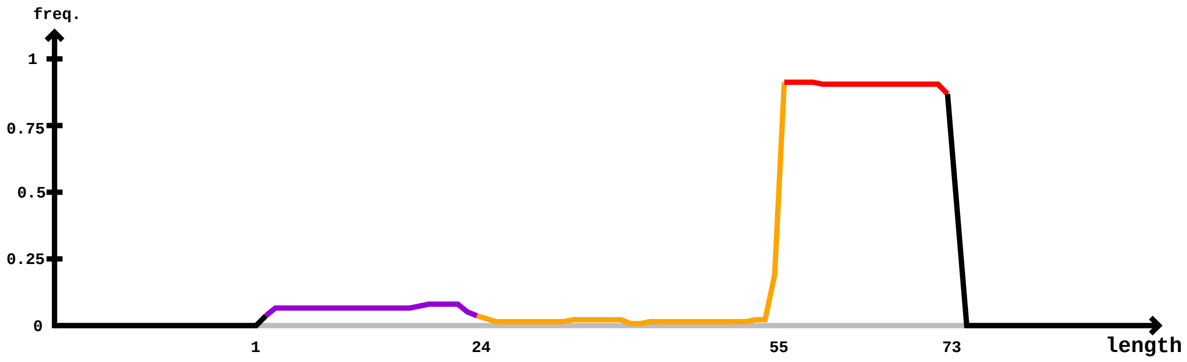

Star **Mature**

```
5'- acugucgccauggccuauguaucaaaggaugagcaaauacugcaacaauaugauccaugaucugauucuuaguauuuaagucucugaugaucucucugccauugcgauuucuc   -3'   obs
    acugucgccauggccuauguaucaaaggaugagcaaauacugcaacaauaugauccaugaucugauucuuaguauuuaagucucugaugaucucucugccauugcgauuucuc         exp
    ...(((((.(((((......((((..(((.((..((((((((.........((((...)))).......))))))))...)))))..))))......))))).)))))..... reads  mm  sample
    ....................aucaaaggaugagAaaauacu........................................................................ 1      1   NN8
    ....................................auacugUaacaauaugauccau....................................................... 1      1   NN8
    .....................................uacugUaacaauaugauccau....................................................... 1      1   NN8
    ............................................................ucugauucuuaguauuua................................... 1      0   NN8
    .......................................................................guauuGaagucucugaugauc..................... 1      1   NN8
    .........................................................................auuuaGgucucugaugau...................... 2      1   NN8
    .........................................................................auuuaagucucAgaugau...................... 1      1   NN8
    .........................................................................auuuaagucucugGugauc..................... 1      1   NN8
    .........................................................................auuuaagucucGgaugauc..................... 2      1   NN8
    .........................................................................auuuaagucucuAaugauc..................... 2      1   NN8
    .........................................................................auuuGagucucugaugauc..................... 1      1   NN8
    .........................................................................auuAaagucucugaugauc..................... 1      1   NN8
    .........................................................................auuuaagucucugaGgauc..................... 1      1   NN8
    .........................................................................auuuaagucAcugaugauc..................... 1      1   NN8
    .........................................................................auuuUagucucugaugauc..................... 1      1   NN8
    .........................................................................auuuaagucucugaCgauc..................... 1      1   NN8
    .........................................................................auuuaGgucucugaugauc..................... 1      1   NN8
    .........................................................................auCuaagucucugaugauc..................... 1      1   NN8
    .........................................................................auuuaaguAucugaugauc..................... 1      1   NN8
    .........................................................................auuuCagucucugaugauc..................... 1      1   NN8
    .........................................................................auGuaagucucugaugauc..................... 2      1   NN8
    ..........................................................................uuuaagucucugaugauA..................... 83     1   NN8

    ....................aucaaaggaugagcaaauacu........................................................................ 1      0   FF1
    ....................aucaaaggaugagcaaauacugc...................................................................... 3      0   FF1
    .....................ucaaaggaugagcaaauacu........................................................................ 2      0   FF1
    .....................ucaaaggaugagcaaauacug....................................................................... 2      0   FF1
    ....................................................auccaugaucugauucuuagu........................................ 1      0   FF1
    .........................................................................auuGaagucucugaugau...................... 1      1   FF1
    .........................................................................auuuaagucucAgaugau...................... 1      1   FF1
    .........................................................................auuuaagucucGgaugauc..................... 1      1   FF1
    .........................................................................auuuaagucucuUaugauc..................... 1      1   FF1
    ..........................................................................uuuaagucucugaugauA..................... 16     1   FF1
```

Star Mature

acugucgccauggccuauguaucaaaggaugagcaaauacugcaacaauaugauccaugaucugauucuuaguauuuaagucucugaugaucucucugccauugcgauuucuc
