# Supplementary material for: Identification of Novel miRNAs and miRNA Expression Profiling in Wheat Hybrid Necrosis
Source: PLoS One. 2015 Feb 23;10(2):e0117507. doi: 10.1371/journal.pone.0117507 (PMC4338152; doi:10.1371/journal.pone.0117507)
Supplement: S2 Fig — Red colored letter: mature miRNA sequence; yellow colored letter: loop sequence; blue colored letter: miRNA* sequence. (ZIP) [file pone.0117507.s002.zip › Figures s1/contig444816_6492.pdf]

Provisional ID	:	contig444816_6492
Score total	:	2.5
Score for star read(s)	:	-1.3
Score for read counts	:	0
Score for mfe	:	2.8
Score for randfold	:	1.6
Score for cons. seed	:	-0.6
Total read count	:	29
Mature read count	:	26
Loop read count	:	0
Star read count	:	3

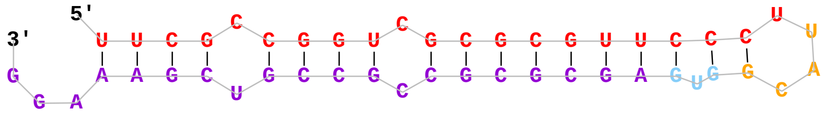

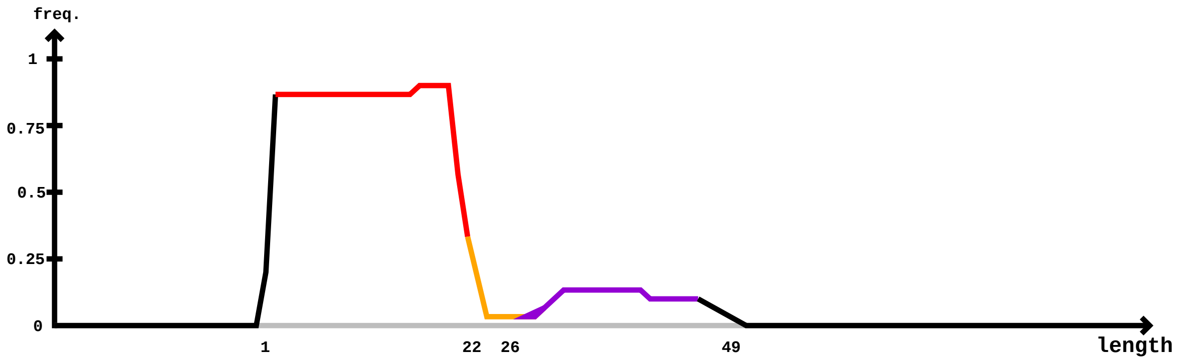

Mature Star

```
5'- ggugggcgccguugccaucccuuucgccggucgcgcguucccuuacggugagcgcgccgccgucgaaagguuuggccgcggugcccuuugccccucucggggugguucuucgu  -3'  obs
    ggugggcgccguugccaucccuuucgccggucgcgcguucccuuacggugagcgcgccgccgucgaaagguuuggccgcggugcccuuugccccucucggggugguucuucgu       exp
    ((.((((((((.((((..(((((((.((((.((((((((((....)).)))))))).)))).)))))))..)))).))))))))))(..((((....))))..)........  reads  mm  sample
    .....................uucgccggucgcgcguucU........................................................................  1      1   NN8
    .................................................agcgcgccgccgAcgaaagg...........................................  1      1   NN8
    ..................................................gcgcgccgccgucgaaagg...........................................  1      0   NN8
    ...................................................cgcgccgccgucgaaagg...........................................  1      0   NN8

    ....................uuucgccggucgcgcguucU........................................................................  1      1   FF1
    ....................uuucgccggucgcgcguuccc.......................................................................  1      0   FF1
    ....................Cuucgccggucgcgcguuccc.......................................................................  1      1   FF1
    ....................uuucgccggucgcgcguucccu......................................................................  3      0   FF1
    .....................uucgcUggucgcgcguucc........................................................................  1      1   FF1
    .....................uucgccggucgcgcguucU........................................................................  4      1   FF1
    .....................uucgccggucgcgcguucc........................................................................  3      0   FF1
    .....................uucgccggucgcgcguuccc.......................................................................  2      0   FF1
    .....................uucgccggucgcgcguuccU.......................................................................  3      1   FF1
    .....................uucgccggucgcgcguucccu......................................................................  5      0   FF1
    .....................uucgccggucgcgcguucccuu.....................................................................  1      0   FF1
    ....................................uucccuuacggugagcgcgccgcc....................................................  1      0   FF1
```
