# Supplementary material for: Identification of Novel miRNAs and miRNA Expression Profiling in Wheat Hybrid Necrosis
Source: PLoS One. 2015 Feb 23;10(2):e0117507. doi: 10.1371/journal.pone.0117507 (PMC4338152; doi:10.1371/journal.pone.0117507)
Supplement: S2 Fig — Red colored letter: mature miRNA sequence; yellow colored letter: loop sequence; blue colored letter: miRNA* sequence. (ZIP) [file pone.0117507.s002.zip › Figures s1/contig4476999_18095.pdf]

| | | |
|---|---|---|
| Provisional ID | : | contig4476999_18095 |
| Score total | : | 2 |
| Score for star read(s) | : | -1.3 |
| Score for read counts | : | 0 |
| Score for mfe | : | 2.3 |
| Score for randfold | : | 1.6 |
| Score for cons. seed | : | -0.6 |
| Total read count | : | 19 |
| Mature read count | : | 19 |
| Loop read count | : | 0 |
| Star read count | : | 0 |

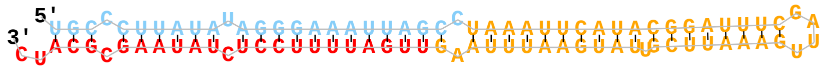

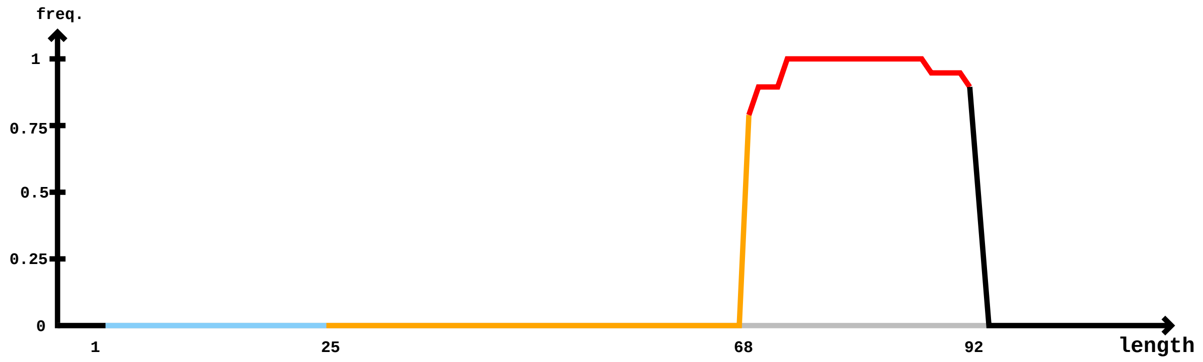


```
       Star                                                                 Mature
5'- agaugcccuuauauagggaaauuagccuaaauucauacggauuucgauugaaauucguuaugaauuuaaguugauuuuccucuauaagcgcaucuaugcucauuucugaguagu   -3'    exp
    ((((((.((((((.((((((((((((.((((((((((((((((((....)))))))).)))))))))).))))))))))))).)))))).)))))).((((((....))))))..     reads   mm    sample
    ......................................................................uugaAuuuccucuauaagc.........................     1       1     FF1
    ......................................................................uugaAuuuccucuauaagcgcau.....................     1       1     FF1
    ......................................................................uuAauuuuccucuauaagcgcauc....................     1       1     FF1
    ......................................................................Augauuuuccucuauaagcgcauc....................     1       1     FF1
    ......................................................................uugaAuuuccucuauaagcgcauc....................     6       1     FF1
    ......................................................................uugauuuuccucuauaagcgcauc....................     4       0     FF1
    ......................................................................uugauuuuccucuauaagcgcauUu...................     1       1     FF1
    .......................................................................ugaAuuuccucuauaagcgcaucu...................     2       1     FF1
    ..........................................................................uuuuccucuauaagcgcaucu...................     2       0     FF1
```
