# Supplementary material for: Identification of Novel miRNAs and miRNA Expression Profiling in Wheat Hybrid Necrosis
Source: PLoS One. 2015 Feb 23;10(2):e0117507. doi: 10.1371/journal.pone.0117507 (PMC4338152; doi:10.1371/journal.pone.0117507)
Supplement: S2 Fig — Red colored letter: mature miRNA sequence; yellow colored letter: loop sequence; blue colored letter: miRNA* sequence. (ZIP) [file pone.0117507.s002.zip › Figures s1/contig474865_6755.pdf]

Provisional ID	:	contig474865_6755
Score total	:	5.7
Score for star read(s)	:	-1.3
Score for read counts	:	0
Score for mfe	:	2.4
Score for randfold	:	1.6
Score for cons. seed	:	3
Total read count	:	1324
Mature read count	:	1323
Loop read count	:	1
Star read count	:	0

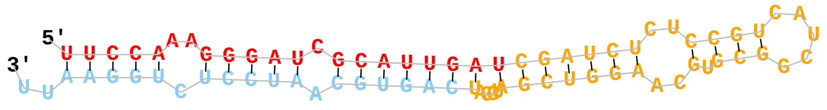

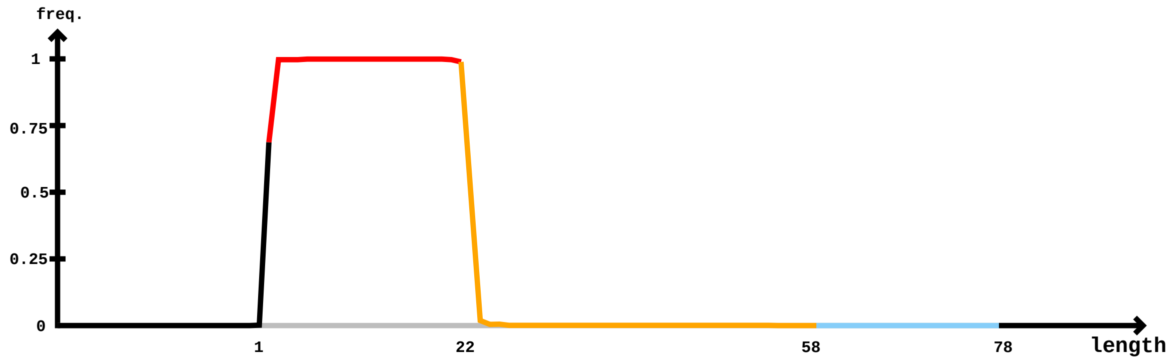

Mature Star

```
5'- uucaggaagcuaguggaggauuccaaagggaucgcauugaucgaucucuccgucaucggcgugcaaggucgauggaucagugcaauccucuggaauucuccgcucgccucc -3'   exp
    ....(((.((.((((((((((((((..(((((.(((((((((((((..((((.....))).)..)))))))....))))))).))))).))))))))))))))).)).)))   reads   mm   sample
    ....................uuccaaagggaucgcauugau......................................................................   7       0    NN8
    ....................uuccaaagggaucgcauugauU.....................................................................   2       1    NN8
    .....................uccaaagggaucgcauugau......................................................................   1       0    NN8
    .....................uccaaagggaucgcauugauc.....................................................................   28      0    NN8
    .....................uGcaaagggaucgcauugauc.....................................................................   1       1    NN8
    .....................uccaaagggaucgcauugaucU....................................................................   2       1    NN8
    .....................uccCaagggaucgcauugaucgau..................................................................   1       1    NN8
    .....................uccaaagggaucgcauugaucgau..................................................................   1       0    NN8
    ........................aaagggaucgcauugauU.....................................................................   1       1    NN8
    ........................aaagggaucgcauugauc.....................................................................   1       0    NN8

    ...................auuccaaagggaucgcauugU.......................................................................   2       1    FF1
    ....................uuccaaagggaucgcauuU........................................................................   1       1    FF1
    ....................uuccaaagggaucgcauuC........................................................................   2       1    FF1
    ....................uuccaaagggaucgcauuga.......................................................................   9       0    FF1
    ....................uuGcaaagggaucgcauugau......................................................................   1       1    FF1
    ....................uuccaaagggaucgGauugau......................................................................   1       1    FF1
    ....................uuccaaagggaucgcGuugau......................................................................   1       1    FF1
    ....................uuccaaagggaucgcauugUu......................................................................   2       1    FF1
    ....................Guccaaagggaucgcauugau......................................................................   2       1    FF1
    ....................uuccaaagggaAcgcauugau......................................................................   2       1    FF1
    ....................uuccaaaggAaucgcauugau......................................................................   1       1    FF1
    ....................uuccaaagggaucgcauGgau......................................................................   1       1    FF1
    ....................uuccaaagggaucgcauugau......................................................................   690     0    FF1
    ....................uuccaUagggaucgcauugau......................................................................   1       1    FF1
    ....................uuccaaagggaucgcauAgau......................................................................   1       1    FF1
    ....................uuccaaagggauAgcauugau......................................................................   1       1    FF1
    ....................uuccaaagggauGgcauugau......................................................................   1       1    FF1
    ....................uuccaGagggaucgcauugau......................................................................   1       1    FF1
    ....................uuccaaagggauUgcauugau......................................................................   1       1    FF1
    ....................uGccaaagggaucgcauugau......................................................................   4       1    FF1
    ....................uuccaaagggaGcgcauugau......................................................................   1       1    FF1
    ....................uuccGaagggaucgcauugau......................................................................   2       1    FF1
    ....................uuccaaagggaucgcaAugau......................................................................   1       1    FF1
```

```
                     Mature                                                  Star
uucaggaagcuaguggaggauuccaaagggaucgcauugaucgaucucuccgucaucggcgugcaaggucgauggaucagugcaauccucuggaauucuccgcucgccucc
....................uuccaaagggaucgcauugauG.....................................................................    1     1     FF1
....................uuccaaagggaucgcauugauA.....................................................................    3     1     FF1
....................uuccaaagggaucgcauugauU.....................................................................    102   1     FF1
....................uAccaaagggaucgcauugauc.....................................................................    1     1     FF1
....................Guccaaagggaucgcauugauc.....................................................................    1     1     FF1
....................uuccaaagggaucgcauugauc.....................................................................    55    0     FF1
....................uuccaaagggaucgcauugaucU....................................................................    8     1     FF1
....................uuccaaagggaucgcauugaucg....................................................................    1     0     FF1
.....................uccaaagggaucgcauugau......................................................................    2     0     FF1
.....................uccaaagggaucCcauugau......................................................................    1     1     FF1
.....................uccaaagggaucgcauugauA.....................................................................    2     1     FF1
.....................Gccaaagggaucgcauugauc.....................................................................    2     1     FF1
.....................Cccaaagggaucgcauugauc.....................................................................    1     1     FF1
.....................uccaaagggaucgcauAgauc.....................................................................    1     1     FF1
.....................uccaaagggaucgcaCugauc.....................................................................    1     1     FF1
.....................uccaaagggaucCcauugauc.....................................................................    2     1     FF1
.....................uccaaagggaucgcauCgauc.....................................................................    1     1     FF1
.....................uccaaagggaucgcauugauU.....................................................................    4     1     FF1
.....................uccaaagggaucgcauugauc.....................................................................    344   0     FF1
.....................ucAaaagggaucgcauugauc.....................................................................    2     1     FF1
.....................uAcaaagggaucgcauugauc.....................................................................    1     1     FF1
.....................uccaaagggaucgcauugaucU....................................................................    7     1     FF1
.....................uccaaagggaucgcauugaucA....................................................................    1     1     FF1
.....................uccaaagggaucgcauugaucgau..................................................................    4     0     FF1
........................aaagggaucgcauugauU.....................................................................    1     1     FF1
............................................ucucuccgucaucggcgugcaaggucgau......................................    1     0     FF1
```
