# Supplementary material for: Identification of Novel miRNAs and miRNA Expression Profiling in Wheat Hybrid Necrosis
Source: PLoS One. 2015 Feb 23;10(2):e0117507. doi: 10.1371/journal.pone.0117507 (PMC4338152; doi:10.1371/journal.pone.0117507)
Supplement: S2 Fig — Red colored letter: mature miRNA sequence; yellow colored letter: loop sequence; blue colored letter: miRNA* sequence. (ZIP) [file pone.0117507.s002.zip › Figures s1/contig476910_6775.pdf]

Provisional ID	:	contig476910_6775
Score total	:	0.2
Score for star read(s)	:	-1.3
Score for read counts	:	-1.3
Score for mfe	:	1.9
Score for randfold	:	1.6
Score for cons. seed	:	-0.6
Total read count	:	9
Mature read count	:	8
Loop read count	:	1
Star read count	:	0

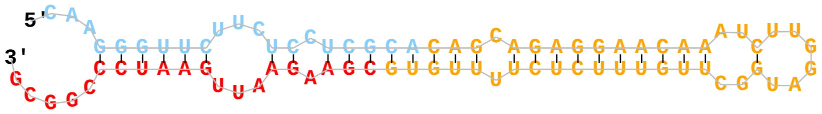

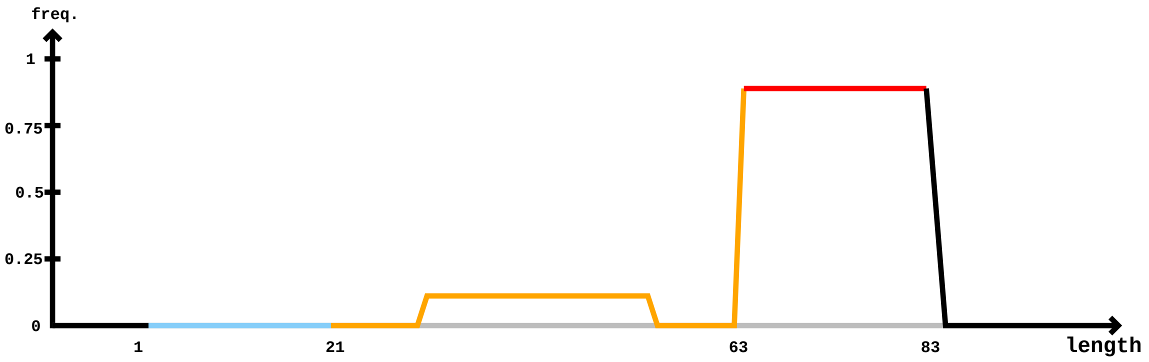

Star Mature

```
5'- ccgcgcgacaaggguucuucuccucgcacagcagaggaacaaaucuuggauggcuuguuucucuuuugugcgaagaauugaaucccggcgaaacgugcuuaguauauuu   -3'    exp
    ..(((((.(..((((((...((.((((((((.((((((((((..(......)..)))))))))).)))))))).))...))))))..).....)))))...........   reads     mm      sample
    ......................................aacaaaucuuggauggcuuguuuc...............................................   1         0       FF1
    ......................................................................cgaagaauugaaucccggcgU...................   1         1       FF1
    ......................................................................cgaagaauugaaucccggcA...................   6         1       FF1
    ......................................................................cgaagaauugaaucccggcg...................   1         0       FF1
```
