# Supplementary material for: Identification of Novel miRNAs and miRNA Expression Profiling in Wheat Hybrid Necrosis
Source: PLoS One. 2015 Feb 23;10(2):e0117507. doi: 10.1371/journal.pone.0117507 (PMC4338152; doi:10.1371/journal.pone.0117507)
Supplement: S2 Fig — Red colored letter: mature miRNA sequence; yellow colored letter: loop sequence; blue colored letter: miRNA* sequence. (ZIP) [file pone.0117507.s002.zip › Figures s1/contig4778939_18647.pdf]

| | | |
|---|---|---|
| Provisional ID | : | contig4778939_18647 |
| Score total | : | 145.2 |
| Score for star read(s) | : | 3.9 |
| Score for read counts | : | 139.8 |
| Score for mfe | : | 0.5 |
| Score for randfold | : | 1.6 |
| Score for cons. seed | : | -0.6 |
| Total read count | : | 286 |
| Mature read count | : | 238 |
| Loop read count | : | 1 |
| Star read count | : | 47 |

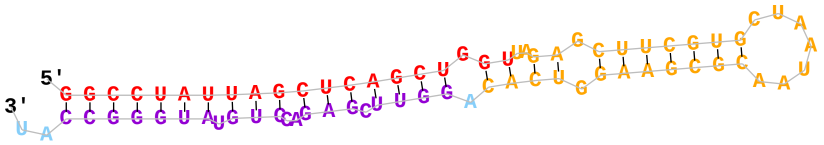


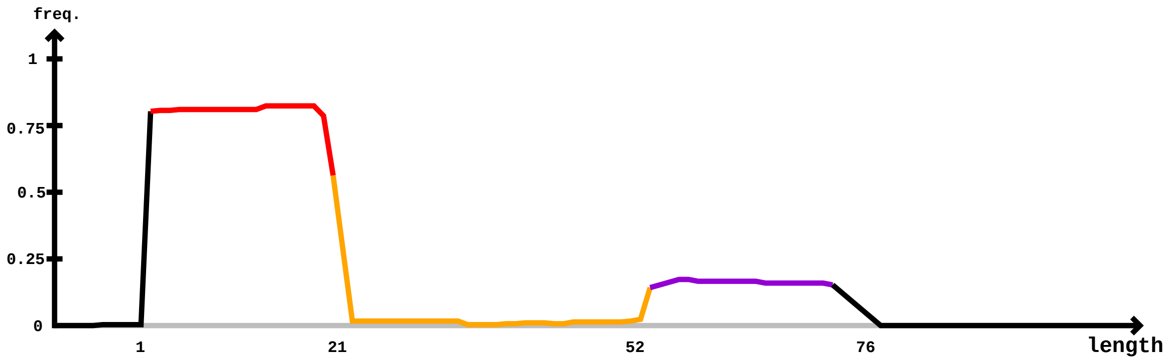


**Mature** **Star**

```
5'- acgcaaaaggccuauuagcucagcugguuagagcuucgugcuaauaaacgcgaaggucacagguucgagaccuguaugggccauuaacauuuuuuuuuacuuuuuuuuuacu  -3'  obs
    acgcaaaaggccuauuagcucagcugguuagagcuucgugcuaauaaacgcgaaggucacagguucgagaccuguaugggccauuaacauuuuuuuuuacuuuuuuuuuacu        exp
    ........(((((((((((((((((.((..((.(((((((........))))))).)))).)))).)))..))).)))))))..............................   reads   mm   sample
    ........ggccuauuaUcucagcug......................................................................................   1       1    NN8
    ........ggccuauuagcucagcug......................................................................................   1       0    NN8
    .........gccuauuagcucagcugg.....................................................................................   1       0    NN8
    ...........cuauuagcucagcugguu...................................................................................   1       0    NN8
    ....................cagcugguuagagcuucgugc.......................................................................   3       0    NN8
    ..............................gagcuucgugcuGauaacgc..............................................................   1       1    NN8
    .....................................................aggucacagguucgGaccu........................................   2       1    NN8
    ............................................................agguucgGgaccuguaugggc...............................   1       1    NN8
    .............................................................gguucgUgaccuguaugggc...............................   5       1    NN8
    .............................................................gguucgGgaccuguaugggc...............................   5       1    NN8
    .............................................................gguucgUgaccuguaugggcc..............................   8       1    NN8
    .............................................................gguAcgagaccuguaugggcc..............................   1       1    NN8
    .............................................................gguucgGgaccuguaugggcc..............................   8       1    NN8
    ..............................................................guucgGgaccuguauggg................................   1       1    NN8
    ..............................................................guucgGgaccuguaugggcc..............................   1       1    NN8
    ...............................................................uucgGgaccuguaugggc...............................   2       1    NN8
    ...............................................................uucgUgaccuguaugggc...............................   1       1    NN8
    ................................................................ucgUgaccuguaugggcc..............................   1       1    NN8
    ................................................................ucgGgaccuguaugggcc..............................   1       1    NN8

    ...Aaaaaggccuauuagcucagcuggu....................................................................................   1       1    FF1
    ........ggccuauuagcucagcAg......................................................................................   1       1    FF1
    ........ggccuauuagcucagcug......................................................................................   8       0    FF1
    ........ggccuauuagcucagcugg.....................................................................................   61      0    FF1
    ........ggccuauuaAcucagcugg.....................................................................................   2       1    FF1
    ........gUccuauuagcucagcugg.....................................................................................   2       1    FF1
    ........ggccuauuagcucagcuggG....................................................................................   1       1    FF1
    ........ggccuauuaAcucagcuggu....................................................................................   1       1    FF1
    ........ggccuauuagcucagcuggu....................................................................................   144     0    FF1
    ........ggccuauuagcucagcugguu...................................................................................   12      0    FF1
    ........ggccuauuagcucagcGgguu...................................................................................   1       1    FF1
    ........ggccuauuagcucagcugguuU..................................................................................   1       1    FF1
    ....................cagcugguuagagcuucgugc.......................................................................   1       0    FF1
```

```
    Mature                                               Star
acgcaaaaggccuauuagcucagcugguuagagcuucgugcuaauaaacgcgaaggucacaggguucgagaccuguaugggccauuaacauuuuuuuuuacuuuuuuuuuacu
..............................................aacgcgaagAucacagguuc..............................................    1    1    FF1
................................................cgcgaagAucacagguuc..............................................    1    1    FF1
...........................................................cagguucgGgaccuguauggg................................    1    1    FF1
............................................................agguucgUgaccuguaugggc...............................    1    1    FF1
.............................................................gguucgGgaccuguaugggc...............................    2    1    FF1
.............................................................gguucgUgaccuguaugggc...............................    2    1    FF1
.............................................................gguucgagaccuguaAgggcc..............................    1    1    FF1
.............................................................gguucgUgaccuguaugggcc..............................    2    1    FF1
.............................................................gguucgGgaccuguaugggcc..............................    1    1    FF1
..............................................................guucgUgaccuguaugggcc..............................    1    1    FF1
................................................................ucgGgaccuguaugggcc..............................    1    1    FF1
```
