# Supplementary material for: Identification of Novel miRNAs and miRNA Expression Profiling in Wheat Hybrid Necrosis
Source: PLoS One. 2015 Feb 23;10(2):e0117507. doi: 10.1371/journal.pone.0117507 (PMC4338152; doi:10.1371/journal.pone.0117507)
Supplement: S2 Fig — Red colored letter: mature miRNA sequence; yellow colored letter: loop sequence; blue colored letter: miRNA* sequence. (ZIP) [file pone.0117507.s002.zip › Figures s1/contig496321_6931.pdf]

Provisional ID : contig496321_6931
Score total : 2.1
Score for star read(s) : -1.3
Score for read counts : 0
Score for mfe : 2.4
Score for randfold : 1.6
Score for cons. seed : -0.6
Total read count : 237
Mature read count : 224
Loop read count : 0
Star read count : 13

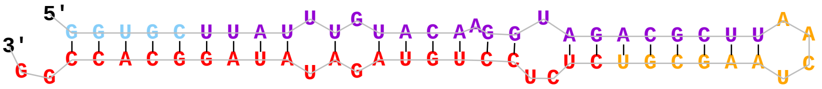

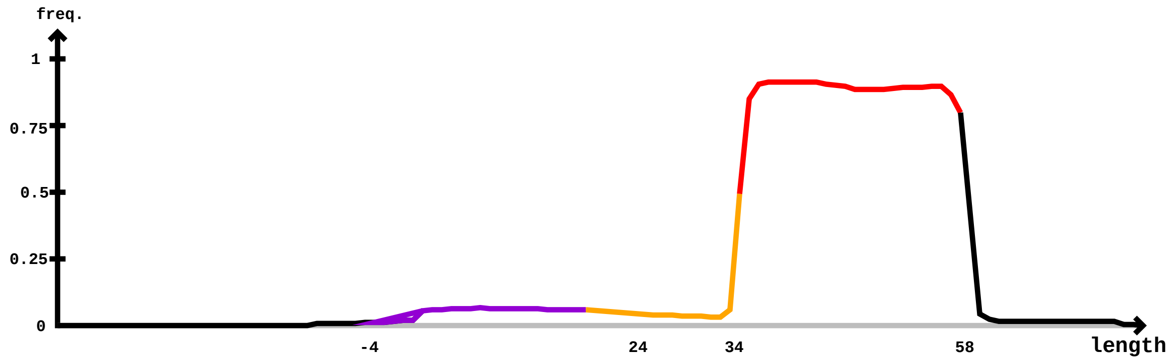

Star Mature

```
5'- cuuagagaauaaccagacuuucuuaagcaccggugcuuauuuguacaagguagacgcuuaacuaagcgucucuccuguagauauaggcaccggugcuucagaaaaucucgg -3' obs
    cuuagagaauaaccagacuuucuuaagcaccggugcuuauuuguacaagguagacgcuuaacuaagcgucucuccuguagauauaggcaccggugcuucagaaaaucucgg     exp
    ............(((((.(((((.((((((((((((((((.(.((((.((.((((((((....))))))))..)))))).).))))))))))))))))).))))).))).))  reads  mm  sample
    ..............................cCgugcuuauuuguacaag..............................................................  1  1  NN8
    ..................................gcuuauuuguacaagguagacgcu.....................................................  1  0  NN8
    ....................................uuauuuguacaGgguagacgcuu....................................................  3  1  NN8
    ....................................uuauuuguacaUgguagacgcuu....................................................  3  1  NN8
    ..........................................guacaGgguagacgcuuaacuaag.............................................  1  1  NN8
    .......................................................gcuuaacuaagUgucucuccugu.................................  2  1  NN8
    .......................................................gcuuaacuaagcgucuUuccugua................................  1  1  NN8
    .........................................................uuCacuaagcgucucuccuguag...............................  1  1  NN8
    .........................................................uuaacuaagcgucuUuccuguaga..............................  1  1  NN8
    .....................................................................cucuccuguagaAauaggcaccg...................  3  1  NN8
    .....................................................................cucuccuguagaAauaggcaccgg..................  40  1  NN8
    ......................................................................ucuccuguagaAauaggcaccg...................  1  1  NN8
    ......................................................................ucuccuguagaAauaggcaccgg..................  4  1  NN8
    ......................................................................ucuccuguagaAauaggcaccggu.................  42  1  NN8
    .......................................................................cuccuguagaAauaggcaccggu.................  2  1  NN8
    .......................................................................cuccuguagaAauaggcaccggug................  2  1  NN8
    ........................................................................uccuguagaAauaggcaccggugc...............  2  1  NN8
    .........................................................................................ccggugcuucagaaaaAcuc..  1  1  NN8
    .............................................................................................ugcuucagaaaaucucgg  1  0  NN8

    .........................agcaccggugcuuauuug....................................................................  1  0  FF1
    .........................agcaccggugcuuauuugua..................................................................  1  0  FF1
    .................................ugcuuauuuguacaGgguagacgcu.....................................................  1  1  FF1
    ....................................uuauuuguacaUgguagacgcuu....................................................  2  1  FF1
    ....................................uuauuuguacaGgguagacgcuu....................................................  1  1  FF1
    .....................................uauuuguacaagAuagacgcuuaacu................................................  1  1  FF1
    .......................................uuuguacaagguagacgcuu....................................................  1  0  FF1
    .............................................caGgguagacgcuuaacuaagcg...........................................  1  1  FF1
    .........................................................uuaacuaagcgucucucUuguaga..............................  1  1  FF1
    .........................................................uuaacuaagcAucucuccuguaga..............................  1  1  FF1
    ....................................................................ucucuccuguagaAauaggcaccg...................  7  1  FF1
    ....................................................................ucucuccuguagaAauaggcaccgg..................  1  1  FF1
    .....................................................................cucuccuguagaAauaggcaccg...................  4  1  FF1
```

Star **Mature**

```
cuuagagaauaaccagacuuucuuaagcaccggugcuuauuuguacaagguagacgcuuaacuaagcgucucuccuguagauauaggcaccggugcuucagaaaaucucgg
.....................................................................cucuccuguagaAauaggcaccgg..................   62   1   FF1
.....................................................................cucuccuguagauauaggcaccgg..................   1    0   FF1
......................................................................Gcuccuguagauauaggcacc....................   1    1   FF1
......................................................................ucuccuguagauauaggcacc....................   7    0   FF1
......................................................................ucuccuguagaAauaggcaccg...................   1    1   FF1
......................................................................uGuccuguagauauaggcaccgg..................   1    1   FF1
......................................................................ucuccuguagaAauaggcaccgg..................   2    1   FF1
......................................................................ucuccuguagaAauaggcaccggu.................   31   1   FF1
.......................................................................cuccuguagaAauaggcaccg...................   1    1   FF1
.......................................................................cuccuguagaAauaggcaccgg..................   5    1   FF1
.......................................................................cuccuguagaAauaggcaccggu.................   1    1   FF1
.......................................................................cuccuguagaGauaggcaccggug................   1    1   FF1
.......................................................................cuccuguagaAauaggcaccggug................   2    1   FF1
.....................................................................................ggcaccggugcuucagaaaaucuc..   1    0   FF1
......................................................................................Ccaccggugcuucagaaaaucuc..   1    1   FF1
```
