# Supplementary material for: Identification of Novel miRNAs and miRNA Expression Profiling in Wheat Hybrid Necrosis
Source: PLoS One. 2015 Feb 23;10(2):e0117507. doi: 10.1371/journal.pone.0117507 (PMC4338152; doi:10.1371/journal.pone.0117507)
Supplement: S2 Fig — Red colored letter: mature miRNA sequence; yellow colored letter: loop sequence; blue colored letter: miRNA* sequence. (ZIP) [file pone.0117507.s002.zip › Figures s1/contig5043762_19076.pdf]

Provisional ID	:	contig5043762_19076
Score total	:	289.3
Score for star read(s)	:	3.9
Score for read counts	:	282.1
Score for mfe	:	2.3
Score for randfold	:	1.6
Score for cons. seed	:	-0.6
Total read count	:	565
Mature read count	:	564
Loop read count	:	0
Star read count	:	1

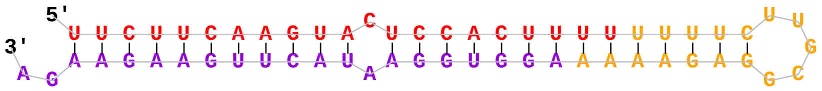

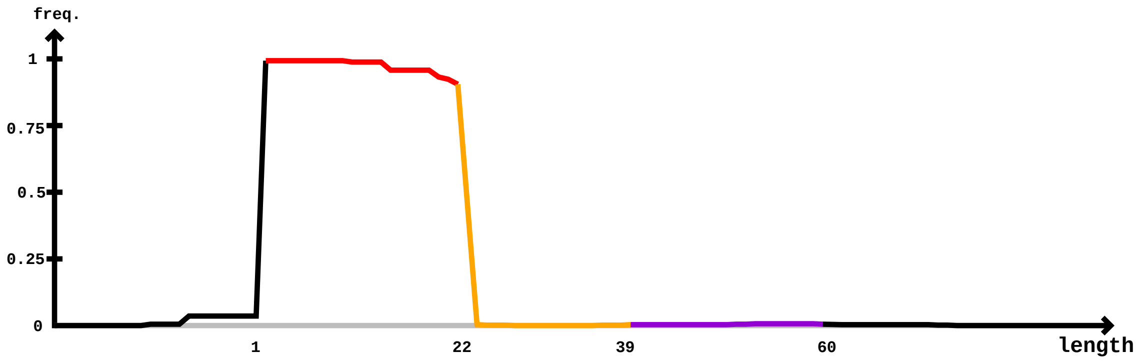

Mature Star

```
5'- agcuggaguaccugaacgucuucuucaaguacuccacuuuuuuuucuugcggagaaaaagguggaauacuugaagaagauuuucaggucguccauuagaaaucucacc  -3'   obs
    agcuggaguaccugaacgucuucuucaaguacuccacuuuuuuuucuugcggagaaaaagguggaauacuugaagaagauuuucaggucguccauuagaaaucucacc         exp
    ...((((..(((((((.(((((((((((((.(((((((((((((.....))))))))))))).))))))))))))).))))))).))))..................   reads   mm   sample
    ....................uucuucaaguacuccacu......................................................................   1       0    NN8
    ....................uucuucaaguacuccacuuuu...................................................................   7       0    NN8

    ........uaccugaacgucuucuucaag...............................................................................   3       0    FF1
    ............ugaacgucuucuCcaaguacu...........................................................................   1       1    FF1
    ............ugaacgucuucuucaaguacu...........................................................................   17      0    FF1
    ....................uucuucaaguacuccacA......................................................................   1       1    FF1
    ....................uucuucaaguacuccacu......................................................................   13      0    FF1
    ....................uucuucaaguacuccacuu.....................................................................   5       0    FF1
    ....................uucuucaaguacuccacAuu....................................................................   1       1    FF1
    ....................uucuucaaguacuccacuuu....................................................................   8       0    FF1
    ....................uucuucaaguacuccacuCu....................................................................   1       1    FF1
    ....................uucuucaaguacuccacCuu....................................................................   1       1    FF1
    ....................uucuucaaguacuccGcuuuu...................................................................   1       1    FF1
    ....................uucuucaaguacuccacAuuu...................................................................   1       1    FF1
    ....................Cucuucaaguacuccacuuuu...................................................................   1       1    FF1
    ....................uucuCcaaguacuccacuuuu...................................................................   1       1    FF1
    ....................uucuucCaguacuccacuuuu...................................................................   1       1    FF1
    ....................uucuucaaguacCccacuuuu...................................................................   3       1    FF1
    ....................uucuucaaguacuccacuuuu...................................................................   505     0    FF1
    ....................uucuucaaguacAccacuuuu...................................................................   1       1    FF1
    ....................uucuucaaguacuccaGuuuu...................................................................   1       1    FF1
    ....................uAcuucaaguacuccacuuuu...................................................................   1       1    FF1
    ....................uucuucaaguacuccacCuuu...................................................................   4       1    FF1
    ....................uucuucGaguacuccacuuuu...................................................................   1       1    FF1
    ....................uucuucaaguacuccacuuuuu..................................................................   2       0    FF1
    ....................uucuucaaguacuccacuuuuA..................................................................   1       1    FF1
    ....................uucuucaaguacuccacuuuCuu.................................................................   1       1    FF1
    ....................uucuucaaguacuccacuuuCuuuuc..............................................................   1       1    FF1
    ......................................................aaaagguggaauacCugaagaag...............................   1       1    FF1
    ..........................................................agguggaauaUuugaagaaga.............................   1       1    FF1
    .....................................................................uugaagaagauuuucaggucg..................   1       0    FF1
    .......................................................................gaagaagauuuucaggucguc................   1       0    FF1
```

Mature Star

agcuggaguaccugaacgucuucuucaaguacuccacuuuuuuuucuugcggagaaaaagguggaauacuugaagaagauuuucaggucguccauuagaaaucucacc
