# Supplementary material for: Identification of Novel miRNAs and miRNA Expression Profiling in Wheat Hybrid Necrosis
Source: PLoS One. 2015 Feb 23;10(2):e0117507. doi: 10.1371/journal.pone.0117507 (PMC4338152; doi:10.1371/journal.pone.0117507)
Supplement: S2 Fig — Red colored letter: mature miRNA sequence; yellow colored letter: loop sequence; blue colored letter: miRNA* sequence. (ZIP) [file pone.0117507.s002.zip › Figures s1/contig5111788_19161.pdf]

Provisional ID	:	contig5111788_19161
Score total	:	30
Score for star read(s)	:	3.9
Score for read counts	:	22.6
Score for mfe	:	2.6
Score for randfold	:	1.6
Score for cons. seed	:	-0.6
Total read count	:	56
Mature read count	:	45
Loop read count	:	0
Star read count	:	11

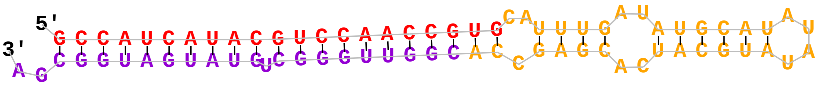

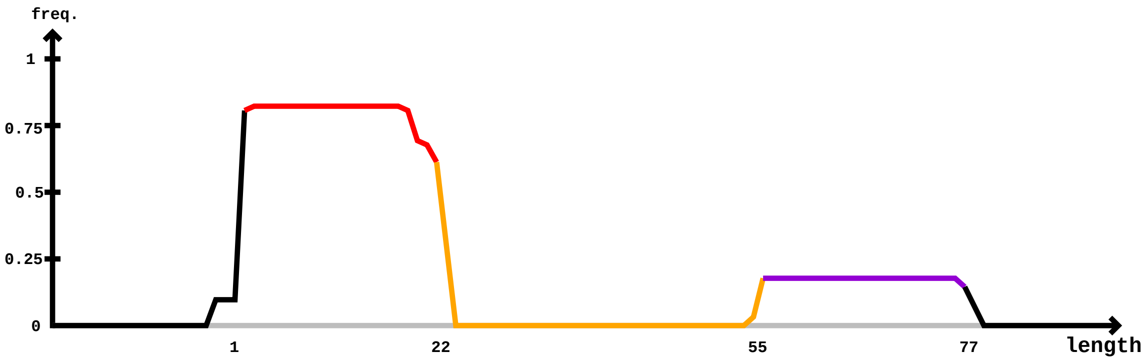

Mature **Star**

```
5'- uacaaaucauucggauucgccaucauacguccaaccgugcauuugauaugcauauauaugcaucacgagccacgguugggcuguaugauggcgauaccgauugguuugu  -3'   obs
    uacaaaucauucggauucgccaucauacguccaaccgugcauuugauaugcauauauaugcaucacgagccacgguugggcuguaugauggcgauaccgauugguuugu        exp
    .((((((((.((((..(((((((((((((((((((((((..((((..((((((....))))))..)))).))))))))))).))))))))))))..)))).))))))))  reads   mm   sample
    ..................gccaucauacguccaaccAu.......................................................................  3       1    NN8
    ..................gccaucauacguccaaccgug......................................................................  10      0    NN8
    ..................gAcaucauacguccaaccgug......................................................................  1       1    NN8
    ..................gccaucauacguccaacUgug......................................................................  1       1    NN8
    ..................gccaucauacguccaaccAug......................................................................  2       1    NN8

    ...............uucgccaucauacguccaac..........................................................................  1       0    FF1
    ...............uucgccaucauUcguccaacc.........................................................................  1       1    FF1
    ...............uucgccaucauacguccaacc.........................................................................  4       0    FF1
    ..................gccaucauacguccaacc.........................................................................  2       0    FF1
    ..................gccaucauacguccaaccg........................................................................  1       0    FF1
    ..................gccaucauacguccaaccAu.......................................................................  1       1    FF1
    ..................gccaucauacguccaaccgug......................................................................  19      0    FF1
    ..................gccaucauacguAcaaccgug......................................................................  1       1    FF1
    ..................gccaucauacguccaacUgug......................................................................  1       1    FF1
    ..................gccaucauacguccaaccAug......................................................................  1       1    FF1
    ..................gccaucauacguccaaccgugU.....................................................................  1       1    FF1
    ...................ccaucauacguccaaccgug......................................................................  1       0    FF1
    .......................................................................acgguugggcuguaugauggcg................  2       0    FF1
    ........................................................................cgguugggcuguaugauggcga...............  8       0    FF1
    ........................................................................cgguugggcuguaugauggcgaC..............  1       1    FF1
```
