# Supplementary material for: Identification of Novel miRNAs and miRNA Expression Profiling in Wheat Hybrid Necrosis
Source: PLoS One. 2015 Feb 23;10(2):e0117507. doi: 10.1371/journal.pone.0117507 (PMC4338152; doi:10.1371/journal.pone.0117507)
Supplement: S2 Fig — Red colored letter: mature miRNA sequence; yellow colored letter: loop sequence; blue colored letter: miRNA* sequence. (ZIP) [file pone.0117507.s002.zip › Figures s1/contig533924_7175.pdf]

Provisional ID	:	contig533924_7175
Score total	:	5.6
Score for star read(s)	:	-1.3
Score for read counts	:	0
Score for mfe	:	2.3
Score for randfold	:	1.6
Score for cons. seed	:	3
Total read count	:	21
Mature read count	:	21
Loop read count	:	0
Star read count	:	0

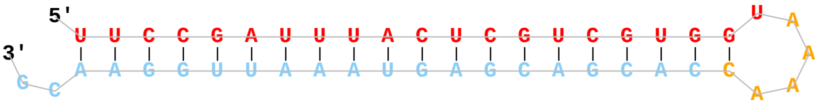

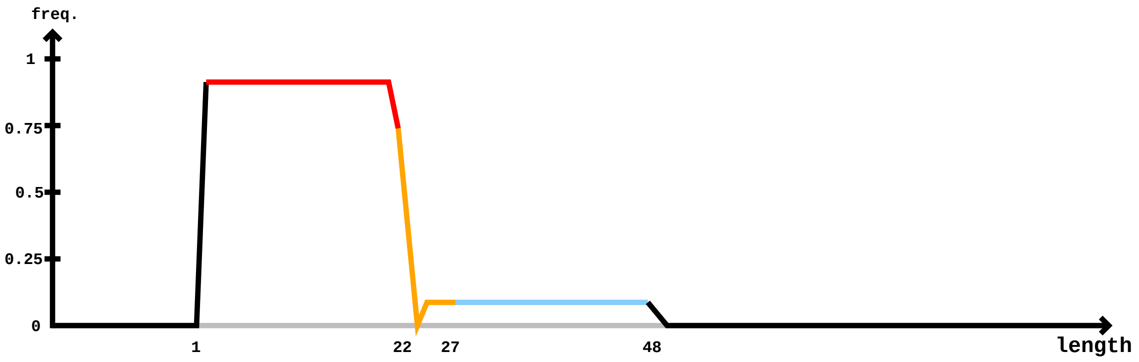

Mature **Star**

```
5'- agguacucccacaguuccgauuuacucgucgugguaaaaccacgacgaguaaauuggaacgaagggaguagcucuguaugaaacuguuaguagguugcugguucuaua   -3'    exp
    ((.(((((((...(((((((((((((((((((((.....)))))))))))))))))))))...))))))).))................(((((........))))).    reads      mm        sample
    ..............uuccAauuuacucgucguggu.........................................................................    1          1         NN8

    ..............uuccAauuuacucgucgugg..........................................................................    4          1         FF1
    ..............uuccAauuuacucgucguggu.........................................................................    14         1         FF1
    ..............uucUgauuuacucgucguggu.........................................................................    2          1         FF1
    .....................................aaccacgacgaguaaUuuggaacg...............................................    2          1         FF1
```
