# Supplementary material for: Identification of Novel miRNAs and miRNA Expression Profiling in Wheat Hybrid Necrosis
Source: PLoS One. 2015 Feb 23;10(2):e0117507. doi: 10.1371/journal.pone.0117507 (PMC4338152; doi:10.1371/journal.pone.0117507)
Supplement: S2 Fig — Red colored letter: mature miRNA sequence; yellow colored letter: loop sequence; blue colored letter: miRNA* sequence. (ZIP) [file pone.0117507.s002.zip › Figures s1/contig580941_7454.pdf]

Provisional ID	:	contig580941_7454
Score total	:	3.2
Score for star read(s)	:	-1.3
Score for read counts	:	-1.3
Score for mfe	:	1.3
Score for randfold	:	1.6
Score for cons. seed	:	3
Total read count	:	9
Mature read count	:	9
Loop read count	:	0
Star read count	:	0

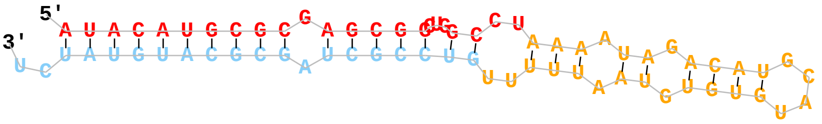

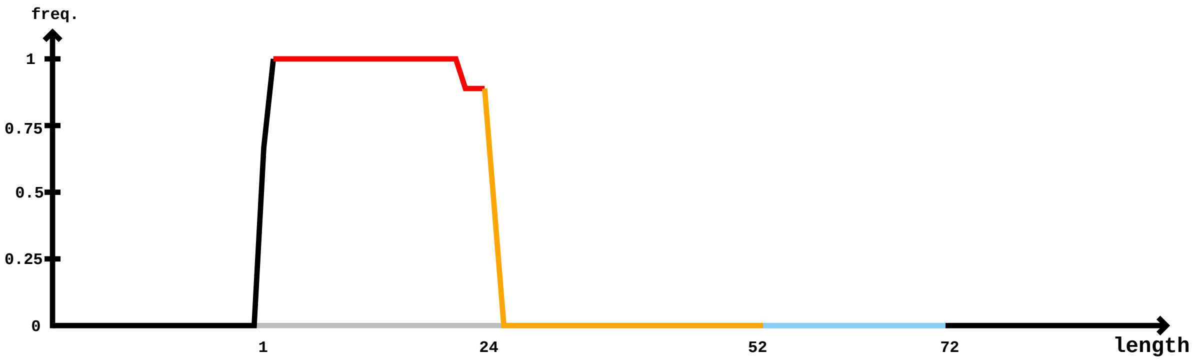

Mature Star

```
5'- gcccacuagcgcgauggggccauacaugcgcgagcggcuggccuaaaauagacaugcauguguguaauuuuuguccgcuagcgcauguaucuuuuagcauuuaauacauucaca   -3'    exp
    ((((.(.........))))).(((((((((.(((((...((..(((.((.((((....)))).)).)))..))))))).)))))))))..........................   reads      mm       sample
    ....................cauacaugcgcgagcggcugg.........................................................................   1          0        NN8
    ....................cauacaugcgcgagcggcuggccu......................................................................   1          0        NN8
    ....................cauacaugcgcgagcggcuggcUu......................................................................   2          1        NN8

    ....................cauacaugcgcgagcggcuggccu......................................................................   2          0        FF1
    .....................auacaugcgcgagcggcuggccu......................................................................   3          0        FF1
```
