# Supplementary material for: Identification of Novel miRNAs and miRNA Expression Profiling in Wheat Hybrid Necrosis
Source: PLoS One. 2015 Feb 23;10(2):e0117507. doi: 10.1371/journal.pone.0117507 (PMC4338152; doi:10.1371/journal.pone.0117507)
Supplement: S2 Fig — Red colored letter: mature miRNA sequence; yellow colored letter: loop sequence; blue colored letter: miRNA* sequence. (ZIP) [file pone.0117507.s002.zip › Figures s1/contig60031_1846.pdf]

Provisional ID	:	contig60031_1846
Score total	:	0.3
Score for star read(s)	:	-1.3
Score for read counts	:	0
Score for mfe	:	0.6
Score for randfold	:	1.6
Score for cons. seed	:	-0.6
Total read count	:	30
Mature read count	:	30
Loop read count	:	0
Star read count	:	0

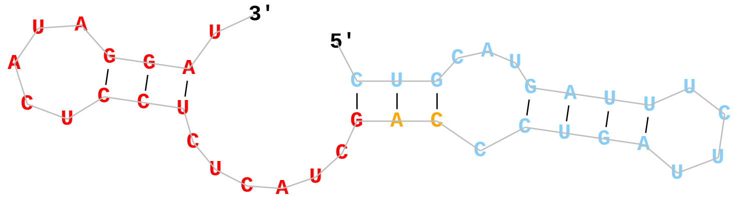

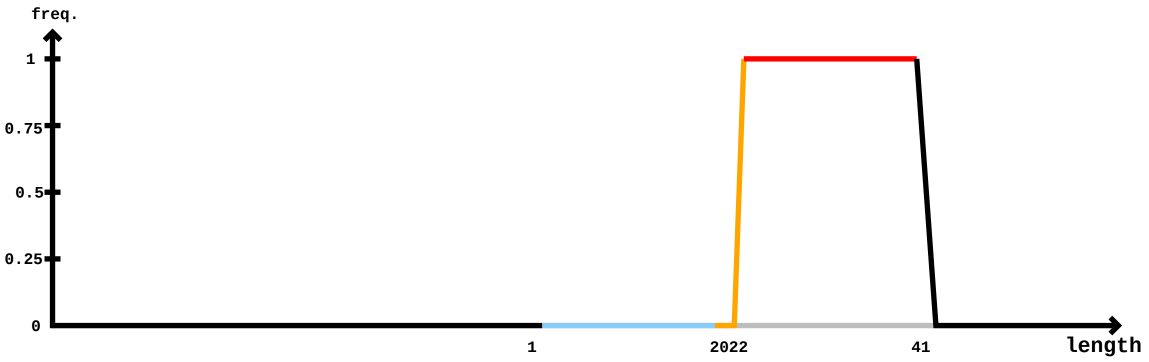

Star Mature

```
5'- cgcuuugguugcugcagagucuagcaacuugcuggacaguucauacggucugcaugauuucuuaguccca gcuacucuccucauaggauccuaaugaaaagcacaaucu   -3'    exp
    ......(((((.(((...(((((((.....)))))))..(((((..(((((..((((.....((((....))))......)))).)))))...)))))..)))))))).   reads      mm          sample
    ......................................................................gcuacucuccucauaggau....................   18         0           NN8
    ......................................................................gcuacucuccucauaAgau....................   1          1           NN8

    ......................................................................gcuacucuccucauaggau....................   10         0           FF1
    ......................................................................gcuacucuccucauaggauc...................   1          0           FF1
```
