# Supplementary material for: Identification of Novel miRNAs and miRNA Expression Profiling in Wheat Hybrid Necrosis
Source: PLoS One. 2015 Feb 23;10(2):e0117507. doi: 10.1371/journal.pone.0117507 (PMC4338152; doi:10.1371/journal.pone.0117507)
Supplement: S2 Fig — Red colored letter: mature miRNA sequence; yellow colored letter: loop sequence; blue colored letter: miRNA* sequence. (ZIP) [file pone.0117507.s002.zip › Figures s1/contig605321_7636.pdf]

Provisional ID	:	contig605321_7636
Score total	:	0.7
Score for star read(s)	:	-1.3
Score for read counts	:	0
Score for mfe	:	1.2
Score for randfold	:	-2.2
Score for cons. seed	:	3
Total read count	:	28
Mature read count	:	28
Loop read count	:	0
Star read count	:	0

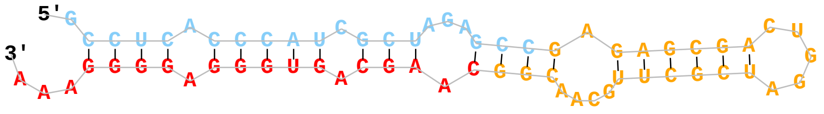

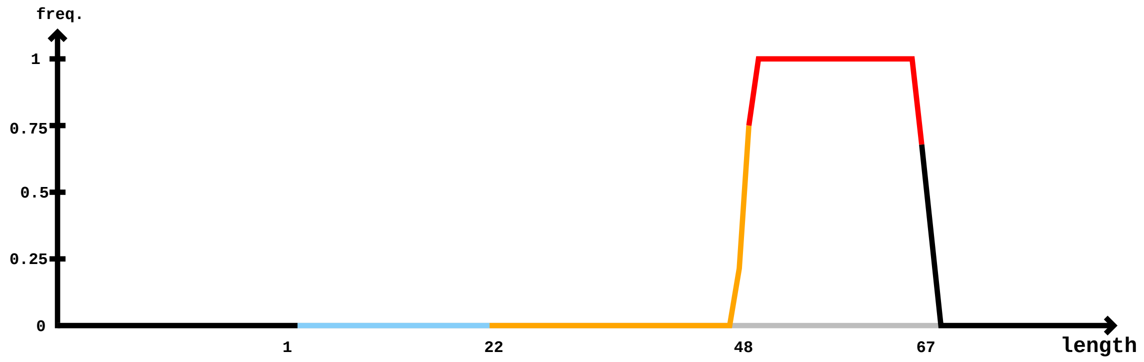

Star Mature

```
5'- ggauaacuaaugagccacagcaugccucacccaucgcuagagccgagagcgacuggaucgcuugcaacggcaagcagugggaggggaaacacugccagagccgacuaa   -3'    exp
    ..........((.((....(((((((((.(((((.(((...((((.((((((.....))))))....)))).))).))))).))))...)).)))....)))).....    reads    mm    sample
    .....................................................................Ccaagcagugggaggggaa....................    2        1     NN8
    .....................................................................Ccaagcagugggaggggaaa...................    3        1     NN8
    ......................................................................caagcagugggaggggaa....................    5        0     NN8
    ......................................................................caagcagugggaggggaaa...................    6        0     NN8
    ......................................................................caagcagugggaggggaaaG..................    1        1     NN8
    .......................................................................aagcagugggaggggaaa...................    5        0     NN8
    .......................................................................aagcagugggaggggaaaG..................    2        1     NN8

    .....................................................................Ccaagcagugggaggggaaa...................    1        1     FF1
    ......................................................................caagcagugggaggggaa....................    2        0     FF1
    ......................................................................caagcagugggaggggaaaG..................    1        1     FF1
```
