# Supplementary material for: Identification of Novel miRNAs and miRNA Expression Profiling in Wheat Hybrid Necrosis
Source: PLoS One. 2015 Feb 23;10(2):e0117507. doi: 10.1371/journal.pone.0117507 (PMC4338152; doi:10.1371/journal.pone.0117507)
Supplement: S2 Fig — Red colored letter: mature miRNA sequence; yellow colored letter: loop sequence; blue colored letter: miRNA* sequence. (ZIP) [file pone.0117507.s002.zip › Figures s1/contig61492_1872.pdf]

Provisional ID	:	contig61492_1872
Score total	:	0
Score for star read(s)	:	-1.3
Score for read counts	:	-0.8
Score for mfe	:	1.1
Score for randfold	:	1.6
Score for cons. seed	:	-0.6
Total read count	:	10
Mature read count	:	5
Loop read count	:	5
Star read count	:	0

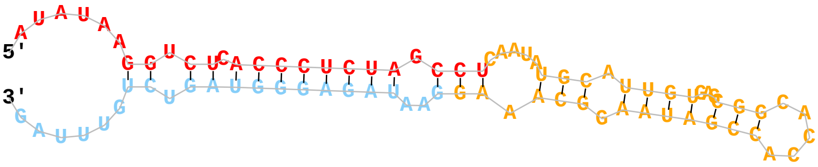

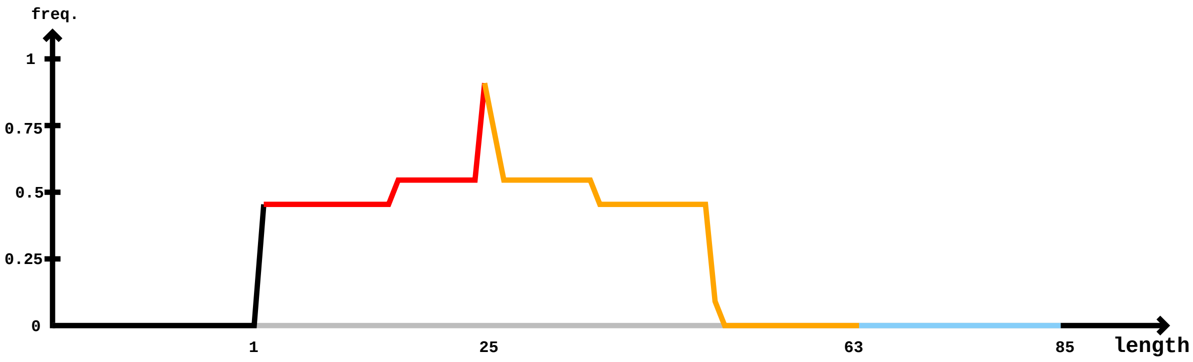

Mature Star

```
5'- auaucuggaacuuggagucuauauaaggucucacccucuagccucaauaugcauugugagcggcaccaccgauaaggcaaaggaauagaggguagucuguuuagcuccaaaaac   -3'    exp
    ..........((((((.(((....(((.((.((((((((.(((.....(((.((((...(((.....))))))).))).)))..)))))))))))))...)))))))))....   reads    mm    sample
    ..................................ccucuagccucaauaugcauu...........................................................   1        0     NN8
    ...........................................ucaauaugcauCgugagcggcacc...............................................   1        1     NN8

    ....................auauaaggucucacccucuagccu......................................................................   5        0     FF1
    ...........................................ucaauGugcauugugagcggcacc...............................................   1        1     FF1
    ...........................................uGaauaugcauugugagcggcacc...............................................   1        1     FF1
    ...........................................ucaauaugcauugugagcggcacc...............................................   1        0     FF1
    ............................................caauaugcauugugagcggcacca..............................................   1        0     FF1
```
