# Supplementary material for: Identification of Novel miRNAs and miRNA Expression Profiling in Wheat Hybrid Necrosis
Source: PLoS One. 2015 Feb 23;10(2):e0117507. doi: 10.1371/journal.pone.0117507 (PMC4338152; doi:10.1371/journal.pone.0117507)
Supplement: S2 Fig — Red colored letter: mature miRNA sequence; yellow colored letter: loop sequence; blue colored letter: miRNA* sequence. (ZIP) [file pone.0117507.s002.zip › Figures s1/contig701555_8210.pdf]

Provisional ID	:	contig701555_8210
Score total	:	1
Score for star read(s)	:	-1.3
Score for read counts	:	0
Score for mfe	:	-2.2
Score for randfold	:	1.6
Score for cons. seed	:	3
Total read count	:	13
Mature read count	:	13
Loop read count	:	0
Star read count	:	0

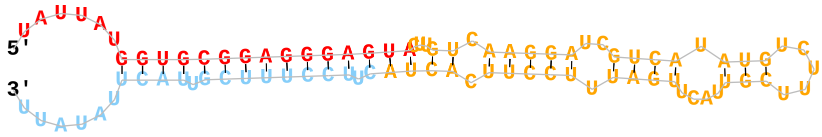

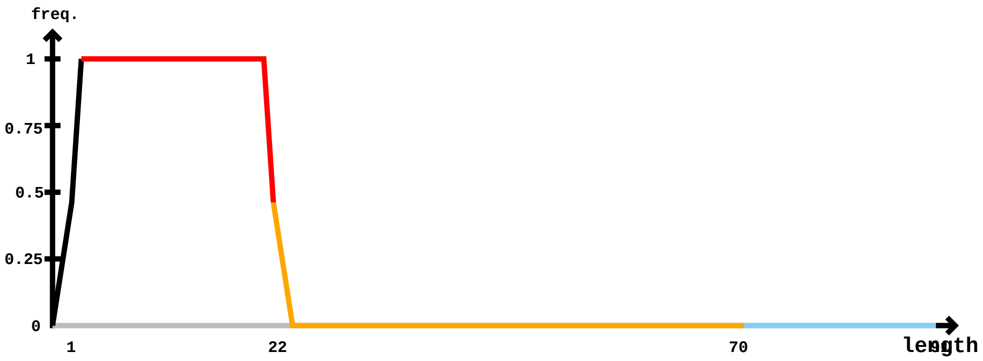

Mature **Star**

```
5'- auauuauggugcggagggaguacuugucaaggaucgucauaugucuuucguuacuugauuuccuucacuacuuccuuucguuacuuauauua  -3'   exp
    ((((...(((((((((((((((...((.(((((..((((.(((.....)))....)))).))))).))))).)))))))))).)))).))))..   reads   mm   sample
    auauuauggGgcggagggagu.......................................................................   1       1    NN8
    auauuauggugcggagggagu.......................................................................   4       0    NN8
    .uauuauggugcggagggagua......................................................................   1       0    NN8

    auauuauggGgcggagggagu.......................................................................   1       1    FF1
    .uauuauggugcggagggagu.......................................................................   1       0    FF1
    .uauuauggugcggagggagua......................................................................   5       0    FF1
```
