# Supplementary material for: Identification of Novel miRNAs and miRNA Expression Profiling in Wheat Hybrid Necrosis
Source: PLoS One. 2015 Feb 23;10(2):e0117507. doi: 10.1371/journal.pone.0117507 (PMC4338152; doi:10.1371/journal.pone.0117507)
Supplement: S2 Fig — Red colored letter: mature miRNA sequence; yellow colored letter: loop sequence; blue colored letter: miRNA* sequence. (ZIP) [file pone.0117507.s002.zip › Figures s1/contig748329_8480.pdf]

Provisional ID	:	contig748329_8480
Score total	:	1.7
Score for star read(s)	:	-1.3
Score for read counts	:	0
Score for mfe	:	2
Score for randfold	:	1.6
Score for cons. seed	:	-0.6
Total read count	:	69
Mature read count	:	69
Loop read count	:	0
Star read count	:	0

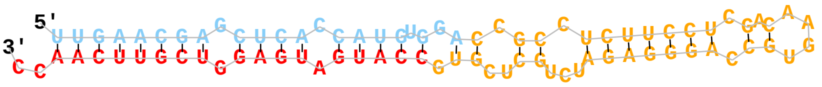

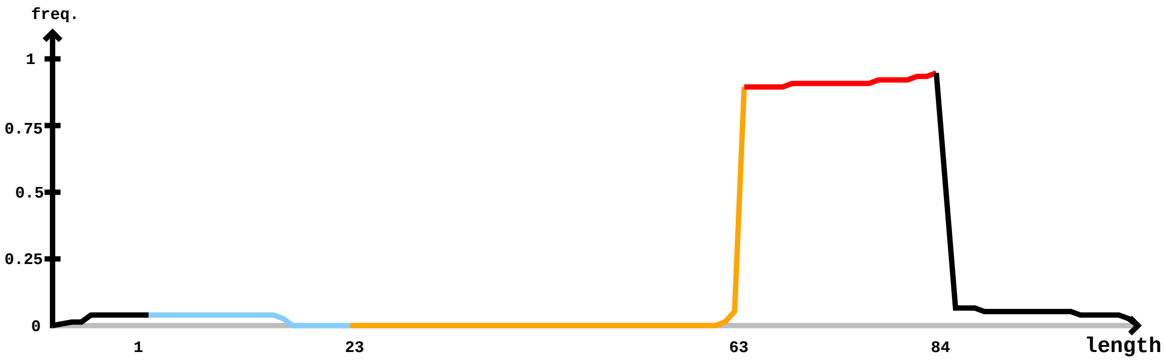

Star   Mature

```
5'- uuugccaguugaacgagcucaccauguggaccgccucuuccucgacaagugccagggagaucugcucgugccaugaugaggucguucaaccagcaaaacggcggcggucgu   -3'    exp
    .((((..(((((((((.((((.((((.(.((.((.(((((((.(.(....)).)))))))...))..)).))))).)))).)))))))))..)))).(((((....)))))    reads   mm   sample
    ....................................................................ugccaCgaugaggucguucaac.....................    1       1    NN8
    .....................................................................gccaCgaugaggucguucaacc....................    3       1    NN8
    ......................................................................ccaCgaugaggucguucaacc....................    1       1    NN8
    ......................................................................ccaugaugaggucguucaacc....................    8       0    NN8
    ........................................................................................accagcaaaacggcggcggucU.    1       1    NN8
    ..........................................................................................cagcaaaacggcggcggucgu    2       0    NN8

    uuugccaguugaacgagcucac.........................................................................................    1       0    FF1
    ..ugccaguugaacgagcucacc........................................................................................    2       0    FF1
    ......................................................................ccaugaGgaggucguucaacc....................    1       1    FF1
    ......................................................................ccaugaugaggucguucaacc....................    50      0    FF1
    ......................................................................ccaCgaugaggucguucaacc....................    3       1    FF1
    ......................................................................ccUugaugaggucguucaacc....................    1       1    FF1
    ...........................................................................augaggucguucaaccagca................    1       0    FF1
    ....................................................................................uucaaccagcaaaacggcggc......    1       0    FF1
```
