# Supplementary material for: Identification of Novel miRNAs and miRNA Expression Profiling in Wheat Hybrid Necrosis
Source: PLoS One. 2015 Feb 23;10(2):e0117507. doi: 10.1371/journal.pone.0117507 (PMC4338152; doi:10.1371/journal.pone.0117507)
Supplement: S2 Fig — Red colored letter: mature miRNA sequence; yellow colored letter: loop sequence; blue colored letter: miRNA* sequence. (ZIP) [file pone.0117507.s002.zip › Figures s1/contig754184_8517.pdf]

Provisional ID	:	contig754184_8517
Score total	:	5.5
Score for star read(s)	:	-1.3
Score for read counts	:	0
Score for mfe	:	2.2
Score for randfold	:	1.6
Score for cons. seed	:	3
Total read count	:	2265
Mature read count	:	2265
Loop read count	:	0
Star read count	:	0

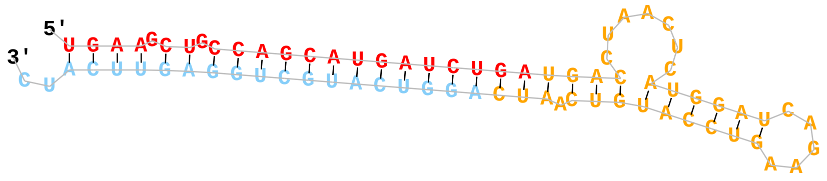

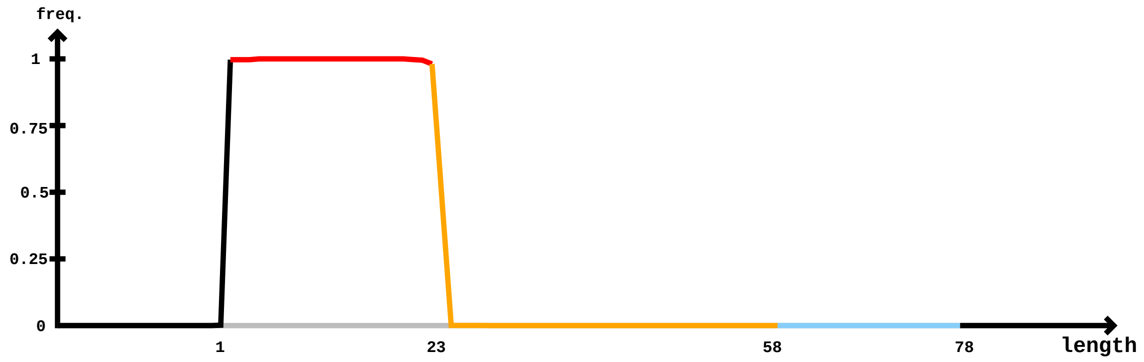

Mature Star

```
5'- ugcaccacgaagcuggugaagcugccagcaugaucugaugaccuaacucauggaucagaaguccaugucaaucaggucaugcuggaguucaucugcuggucggagcac   -3'    exp
    (((.((((..(((.((((((.((.((((((((((((((((((.......((((((.....))))))))).))))))))))))))))))))))).))).)).)).))).    reads    mm    sample
    ................ugaagcugccagcaugaucuga......................................................................    15       0     NN8
    ................ugaagcugccagcaugaucugC......................................................................    20       1     NN8
    ...................agcugccagcaugaucuga......................................................................    1        0     NN8
    ...................agcugccagcaugaucugC......................................................................    6        1     NN8

    ...............Cugaagcugccagcaugauc.........................................................................    2        1     FF1
    ...............Cugaagcugccagcaugaucug.......................................................................    1        1     FF1
    ................ugaagcugccagcaugauc.........................................................................    4        0     FF1
    ................ugaagcugccagcaugaucu........................................................................    4        0     FF1
    ................ugaagcugccagcaugaucA........................................................................    1        1     FF1
    ................ugaagcugccagcaugaucuU.......................................................................    1        1     FF1
    ................ugaagcugccagcaugaucuC.......................................................................    1        1     FF1
    ................ugaagcugccagcaugaucug.......................................................................    28       0     FF1
    ................ugaagcugccagcauAaucug.......................................................................    1        1     FF1
    ................ugaagcugccagcaugaucugU......................................................................    10       1     FF1
    ................ugaagcugccagAaugaucuga......................................................................    3        1     FF1
    ................ugaagcugccagcaugaucugC......................................................................    949      1     FF1
    ................ugaagcugccagcaugaGcuga......................................................................    1        1     FF1
    ................uUaagcugccagcaugaucuga......................................................................    1        1     FF1
    ................ugaagcugccagcGugaucuga......................................................................    1        1     FF1
    ................ugaagcugccagcaugaAcuga......................................................................    1        1     FF1
    ................ugaagcugccagcaugaucuga......................................................................    1179     0     FF1
    ................ugaagcugccagcauUaucuga......................................................................    3        1     FF1
    ................ugaagcGgccagcaugaucuga......................................................................    2        1     FF1
    ................uAaagcugccagcaugaucuga......................................................................    1        1     FF1
    ................Cgaagcugccagcaugaucuga......................................................................    1        1     FF1
    ................ugaagcugAcagcaugaucuga......................................................................    1        1     FF1
    ................Ggaagcugccagcaugaucuga......................................................................    4        1     FF1
    ................ugaagcugccaAcaugaucuga......................................................................    1        1     FF1
    ................ugaagcugccagUaugaucuga......................................................................    1        1     FF1
    ................ugaagcugccagcaugUucuga......................................................................    1        1     FF1
    ................ugaagcugccagGaugaucuga......................................................................    3        1     FF1
    ................ugaagcugccaUcaugaucuga......................................................................    2        1     FF1
    ................ugaagcugccagcaugaucugG......................................................................    5        1     FF1
```

```
                Mature                                                    Star
ugcaccacgaagcuggugaagcugccagcaugaucugaugaccuaacucauggaucagaaguccaugucaaucaggucaugcuggaguucaucugcuggucggagcac
................ugaagcugccagcauAaucuga......................................................................    1    1    FF1
................ugaUgcugccagcaugaucuga......................................................................    1    1    FF1
................ugaagcugccagcaCgaucuga......................................................................    1    1    FF1
................ugaagcugccGgcaugaucuga......................................................................    1    1    FF1
................ugaagcugccagcaugaucugaA.....................................................................    5    1    FF1
................ugaagcugccagcaugaucugaugacc.................................................................    1    0    FF1
```
