# Supplementary material for: Identification of Novel miRNAs and miRNA Expression Profiling in Wheat Hybrid Necrosis
Source: PLoS One. 2015 Feb 23;10(2):e0117507. doi: 10.1371/journal.pone.0117507 (PMC4338152; doi:10.1371/journal.pone.0117507)
Supplement: S2 Fig — Red colored letter: mature miRNA sequence; yellow colored letter: loop sequence; blue colored letter: miRNA* sequence. (ZIP) [file pone.0117507.s002.zip › Figures s1/contig839524_8997.pdf]

Provisional ID	:	contig839524_8997
Score total	:	217.6
Score for star read(s)	:	3.9
Score for read counts	:	207.1
Score for mfe	:	2
Score for randfold	:	1.6
Score for cons. seed	:	3
Total read count	:	418
Mature read count	:	416
Loop read count	:	0
Star read count	:	2

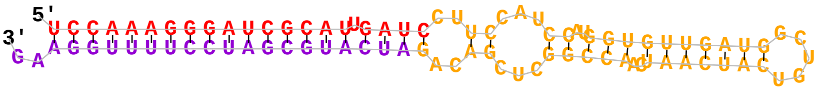

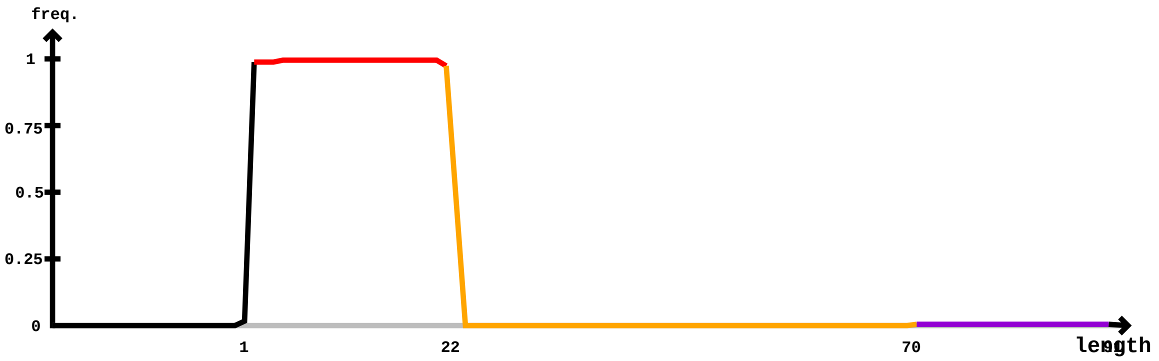

Mature **Star**

```
5'- acaaugccuuggggaagcauccaaagggaucgcauugauccuuccauccaugguguugauggcugucaucaaucaaccggcucgacagaucaugcgauccuuuuggaagc  -3'   obs
    acaaugccuuggggaagcauccaaagggaucgcauugauccuuccauccaugguguugauggcugucaucaaucaaccggcucgacagaucaugcgauccuuuuggaagc        exp
    ......((....))..((.((((((((((((((((.((((..((...((..((((((((((.....)))))))..)))))...))..)))))))))))))))))))).))   reads      mm        sample
    ...................uccaaagggaucgcauugau.......................................................................   1          0         NN8
    ...................uGcaaagggaucgcauugauc......................................................................   1          1         NN8
    ...................uccaaagggaucgcauugauc......................................................................   28         0         NN8
    ...................uccaaagggaucgcauugaucU.....................................................................   2          1         NN8
    ......................aaagggaucgcauugauc......................................................................   1          0         NN8
    ......................aaagggaucgcauugauU......................................................................   1          1         NN8

    ..................auccaaagggaucgcauugau.......................................................................   3          0         FF1
    ..................Guccaaagggaucgcauugau.......................................................................   2          1         FF1
    ..................auccaaagggaucgcauugauc......................................................................   1          0         FF1
    ..................Guccaaagggaucgcauugauc......................................................................   1          1         FF1
    ...................uccaaagggaucCcauugau.......................................................................   1          1         FF1
    ...................uccaaagggaucgcauugau.......................................................................   2          0         FF1
    ...................ucAaaagggaucgcauugauc......................................................................   2          1         FF1
    ...................Gccaaagggaucgcauugauc......................................................................   2          1         FF1
    ...................uAcaaagggaucgcauugauc......................................................................   1          1         FF1
    ...................Cccaaagggaucgcauugauc......................................................................   1          1         FF1
    ...................uccaaagggaucCcauugauc......................................................................   2          1         FF1
    ...................uccaaagggaucgcauAgauc......................................................................   1          1         FF1
    ...................uccaaagggaucgcauugauA......................................................................   2          1         FF1
    ...................uccaaagggaucgcaCugauc......................................................................   1          1         FF1
    ...................uccaaagggaucgcauCgauc......................................................................   1          1         FF1
    ...................uccaaagggaucgcauugauc......................................................................   344        0         FF1
    ...................uccaaagggaucgcauugauU......................................................................   4          1         FF1
    ...................uccaaagggaucgcauugaucU.....................................................................   7          1         FF1
    ...................uccaaagggaucgcauugaucc.....................................................................   2          0         FF1
    ...................uccaaagggaucgcauugaucA.....................................................................   1          1         FF1
    ......................aaagggaucgcauugauU......................................................................   1          1         FF1
    ........................................................................................aucaugcgauccuuuuggaag.   2          0         FF1
```
