# Supplementary material for: Identification of Novel miRNAs and miRNA Expression Profiling in Wheat Hybrid Necrosis
Source: PLoS One. 2015 Feb 23;10(2):e0117507. doi: 10.1371/journal.pone.0117507 (PMC4338152; doi:10.1371/journal.pone.0117507)
Supplement: S2 Fig — Red colored letter: mature miRNA sequence; yellow colored letter: loop sequence; blue colored letter: miRNA* sequence. (ZIP) [file pone.0117507.s002.zip › Figures s1/contig853436_9080.pdf]

Provisional ID	:	contig853436_9080
Score total	:	9279
Score for star read(s)	:	3.9
Score for read counts	:	9267.7
Score for mfe	:	2.7
Score for randfold	:	1.6
Score for cons. seed	:	3
Total read count	:	18190
Mature read count	:	15464
Loop read count	:	0
Star read count	:	2726

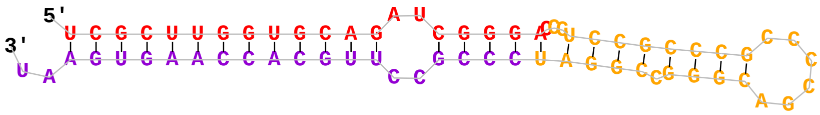

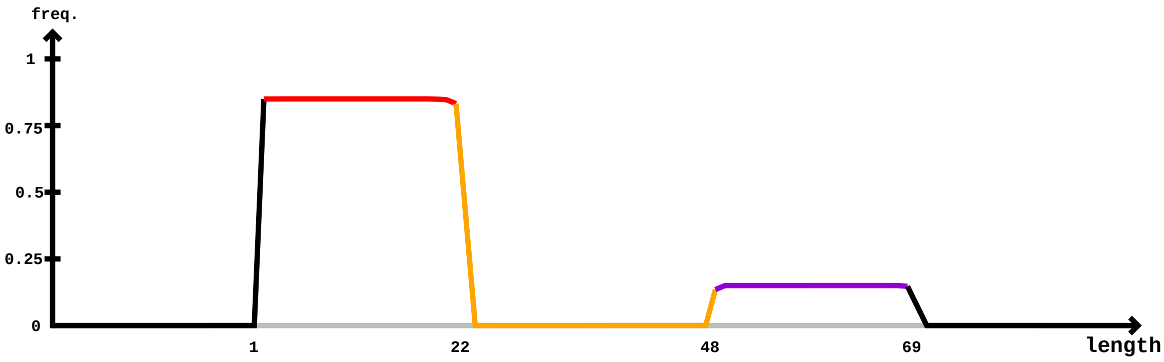

Mature Star

```
5'- ccgucgccgccgccucgggcucgcuuggugcagaucgggacccuccgcccgccccgacgggccggaucccgccuugcaccaagugaaucggagccggcgcagcgaccucgc   -3'   obs
    ccgucgccgccgccucgggcucgcuuggugcagaucgggacccuccgcccgccccgacgggccggaucccgccuugcaccaagugaaucggagccggcgcagcgaccucgc         exp
    ..((((((((((.(((.(..((((((((((((((..(((((...((((((((......)))).)))))))))..)))))))))))))..).))).)))))..)))))..... reads   mm   sample
    ....................ucgcuuggugcagaucgg......................................................................... 2      0    NN8
    ....................ucgcuuggugcagaucggg........................................................................ 6      0    NN8
    ....................ucgcuuggugcagaucggga....................................................................... 80     0    NN8
    ....................Ccgcuuggugcagaucggga....................................................................... 1      1    NN8
    ....................ucgcuuggGgcagaucggga....................................................................... 1      1    NN8
    ....................ucgcGuggugcagaucggga....................................................................... 1      1    NN8
    ....................ucgcuuggugcagauUgggac...................................................................... 2      1    NN8
    ....................ucgcuCggugcagaucgggac...................................................................... 1      1    NN8
    ....................ucAcuuggugcagaucgggac...................................................................... 2      1    NN8
    ....................ucgcuuggugcagaCcgggac...................................................................... 2      1    NN8
    ....................Gcgcuuggugcagaucgggac...................................................................... 2      1    NN8
    ....................ucgcuuggugcGgaucgggac...................................................................... 2      1    NN8
    ....................uGgcuuggugcagaucgggac...................................................................... 4      1    NN8
    ....................ucgcuuggugcagaucgUgac...................................................................... 1      1    NN8
    ....................Acgcuuggugcagaucgggac...................................................................... 3      1    NN8
    ....................ucgcuuggugcagaucggUac...................................................................... 1      1    NN8
    ....................ucgcuuggugcagaucgggaA...................................................................... 1      1    NN8
    ....................uAgcuuggugcagaucgggac...................................................................... 1      1    NN8
    ....................ucgcuuggugcagauGgggac...................................................................... 1      1    NN8
    ....................ucgcuuggugcagaGcgggac...................................................................... 1      1    NN8
    ....................ucgcuuggugcagaucgAgac...................................................................... 1      1    NN8
    ....................ucgcuuggugcUgaucgggac...................................................................... 2      1    NN8
    ....................ucgcuuggugcagCucgggac...................................................................... 1      1    NN8
    ....................ucgcuuggAgcagaucgggac...................................................................... 3      1    NN8
    ....................ucgcuuggugcagaucgggac...................................................................... 1931   0    NN8
    ....................ucgcuuAgugcagaucgggac...................................................................... 2      1    NN8
    ....................ucgcuuggugcaUaucgggac...................................................................... 2      1    NN8
    ....................ucgcuugguUcagaucgggac...................................................................... 1      1    NN8
    ....................ucgcuuggugcagaucAggac...................................................................... 3      1    NN8
    ....................ucgcuuggugcCgaucgggac...................................................................... 2      1    NN8
    ....................ucgcuuggugGagaucgggac...................................................................... 2      1    NN8
    ....................ucgAuuggugcagaucgggac...................................................................... 1      1    NN8
    ....................ucgcuuggugUagaucgggac...................................................................... 1      1    NN8
```

Mature / Star

```
ccgucgccgccgccucgggcucgcuuggugcagaucgggacccuccgcccgccccgacgggccggaucccgccuugcaccaagugaaucggagccggcgcagcgaccucgc
....................ucgcuuggGgcagaucgggac......................................................................  5    1  NN8
....................ucgcuuggugcaAaucgggac......................................................................  2    1  NN8
....................ucgcuuggugcagaucgggaU......................................................................  20   1  NN8
....................ucgcCuggugcagaucgggac......................................................................  1    1  NN8
....................ucgcuuggugcagauAgggac......................................................................  2    1  NN8
....................ucCcuuggugcagaucgggac......................................................................  1    1  NN8
....................ucgcuuggugcagaucggAac......................................................................  1    1  NN8
....................uUgcuuggugcagaucgggac......................................................................  1    1  NN8
....................ucgcuGggugcagaucgggac......................................................................  1    1  NN8
....................ucgcuuUgugcagaucgggac......................................................................  1    1  NN8
....................ucgcuuggugAagaucgggac......................................................................  4    1  NN8
....................ucgcuuggugcagGucgggac......................................................................  1    1  NN8
....................ucgcGuggugcagaucgggac......................................................................  1    1  NN8
....................ucgcuuggugcagaucgggacc.....................................................................  33   0  NN8
....................ucgcuuggugcagaucgggacU.....................................................................  16   1  NN8
....................ucgcuuggugcagaucgggacA.....................................................................  1    1  NN8
....................ucgcuuggugcagaucgggacccuc..................................................................  1    0  NN8
.....................cgcuuggugcagaucgggac......................................................................  2    0  NN8
......................gcuuggugcagaucgggac......................................................................  1    0  NN8
........................................................acgggccggaucccgccuugcaccaagugaau.......................  1    0  NN8
..............................................................cggaucccgccuugcaccaagugaU........................  1    1  NN8
..................................................................Ccccgccuugcaccaagugaau.......................  1    1  NN8
...................................................................cccgccuugcaccaagugaa........................  9    0  NN8
...................................................................cccgccuugcaccaGgugaau.......................  1    1  NN8
...................................................................cccgccuugcaccaagugaGu.......................  1    1  NN8
...................................................................cccgccuugcaccaagugaaA.......................  3    1  NN8
...................................................................cccgccuugcaccaagugaau.......................  342  0  NN8
...................................................................cccgcAuugcaccaagugaau.......................  1    1  NN8
...................................................................cccgccuugcaccUagugaau.......................  1    1  NN8
...................................................................cccgccuugcaccaagugaCu.......................  1    1  NN8
...................................................................cccgccuugcaccaaAugaau.......................  2    1  NN8
...................................................................cccgcGuugcaccaagugaau.......................  1    1  NN8
...................................................................cccgccuugcaccaagugaauU......................  1    1  NN8
....................................................................ccgccuugcaccaagugaa........................  5    0  NN8
....................................................................ccgccuAgcaccaagugaau.......................  1    1  NN8
....................................................................Acgccuugcaccaagugaau.......................  2    1  NN8
....................................................................ccgccuugcaccaagugaau.......................  108  0  NN8
....................................................................ccgcGuugcaccaagugaau.......................  2    1  NN8
....................................................................ccgccuugcaccaagugaUu.......................  2    1  NN8
....................................................................ccgccuugcaccaagugaauU......................  1    1  NN8

...............cgggcucgcuuggugcagauc...........................................................................  1    0  FF1
....................ucgcuuggAgcagaucgg.........................................................................  1    1  FF1
....................ucgcuuggugcagaucgU.........................................................................  1    1  FF1
....................ucgcuuggugcagaucgg.........................................................................  11   0  FF1
....................ucgcuuggugcaUaucggg........................................................................  1    1  FF1
....................ucgcuuggugcaAaucggg........................................................................  1    1  FF1
....................ucgcuuggugcagaucggg........................................................................  30   0  FF1
....................ucgcuuggugcagaucgggG.......................................................................  1    1  FF1
....................ucAcuuggugcagaucggga.......................................................................  1    1  FF1
....................Gcgcuuggugcagaucggga.......................................................................  3    1  FF1
....................ucgcuuggugcagaCcggga.......................................................................  1    1  FF1
....................ucgcuuggugcagaucggga.......................................................................  181  0  FF1
....................ucgcuuggugcagaucgggU.......................................................................  1    1  FF1
....................uAgcuuggugcagaucggga.......................................................................  1    1  FF1
....................ucgcuuggugcagaucCggac......................................................................  4    1  FF1
....................uGgcuuggugcagaucgggac......................................................................  21   1  FF1
....................Acgcuuggugcagaucgggac......................................................................  19   1  FF1
....................ucgcuuggugcagauUgggac......................................................................  11   1  FF1
....................ucgcuuggugcagCucgggac......................................................................  1    1  FF1
....................ucgcuugguAcagaucgggac......................................................................  2    1  FF1
....................ucgcuuggugAagaucgggac......................................................................  8    1  FF1
....................ucgUuuggugcagaucgggac......................................................................  2    1  FF1
....................ucgcuuggugcagaucggAac......................................................................  6    1  FF1
....................ucgcuuggugcagaucgggCc......................................................................  1    1  FF1
....................ucgcuuggAgcagaucgggac......................................................................  20   1  FF1
....................ucgcuuggugcagauAgggac......................................................................  18   1  FF1
....................uAgcuuggugcagaucgggac......................................................................  8    1  FF1
....................ucgcuuggugcagaucgAgac......................................................................  9    1  FF1
....................ucgcuuggugcagGucgggac......................................................................  4    1  FF1
```

```
                    Mature                                              Star
ccgucgccgccgccucgggcucgcuuggugcagaucgggacccuccgcccgccccgacgggccggaucccgccuugcaccaagugaaucggagccggcgcagcgaccucgc
....................ucgcuuggugGagaucgggac......................................................................   7      1   FF1
....................ucgcuuggugcaUaucgggac......................................................................   11     1   FF1
....................ucgcuuggugcagaucUggac......................................................................   5      1   FF1
....................ucgcCuggugcagaucgggac......................................................................   1      1   FF1
....................ucgcuuggugcagaCcgggac......................................................................   3      1   FF1
....................ucgAuuggugcagaucgggac......................................................................   5      1   FF1
....................ucgcuuggGgcagaucgggac......................................................................   47     1   FF1
....................ucgcuuggugcagaucgggaU......................................................................   161    1   FF1
....................ucgcuugCugcagaucgggac......................................................................   1      1   FF1
....................ucgcuuggugcaAaucgggac......................................................................   9      1   FF1
....................ucgcuuggugcagaucgggaA......................................................................   3      1   FF1
....................ucgcuuggugcagaucgCgac......................................................................   9      1   FF1
....................ucUcuuggugcagaucgggac......................................................................   2      1   FF1
....................ucgcuuggugcagaucggUac......................................................................   4      1   FF1
....................ucgcuuggugcaCaucgggac......................................................................   26     1   FF1
....................ucgcuuggugcagaucgUgac......................................................................   4      1   FF1
....................ucgcuugguCcagaucgggac......................................................................   2      1   FF1
....................ucgcuuggugcagaGcgggac......................................................................   1      1   FF1
....................Ccgcuuggugcagaucgggac......................................................................   8      1   FF1
....................ucgcuuggugcagaucgggac......................................................................   12265  0   FF1
....................ucAcuuggugcagaucgggac......................................................................   12     1   FF1
....................ucgcuuggugUagaucgggac......................................................................   3      1   FF1
....................ucgcuuggugcagaucggCac......................................................................   3      1   FF1
....................ucgcuuggugcagUucgggac......................................................................   2      1   FF1
....................ucgcAuggugcagaucgggac......................................................................   3      1   FF1
....................ucCcuuggugcagaucgggac......................................................................   6      1   FF1
....................ucgcuuAgugcagaucgggac......................................................................   4      1   FF1
....................Gcgcuuggugcagaucgggac......................................................................   42     1   FF1
....................ucgGuuggugcagaucgggac......................................................................   11     1   FF1
....................ucgcuugAugcagaucgggac......................................................................   7      1   FF1
....................ucgcuuggCgcagaucgggac......................................................................   14     1   FF1
....................ucgcuAggugcagaucgggac......................................................................   2      1   FF1
....................ucgcuuggugcagaucgggGc......................................................................   7      1   FF1
....................ucgcuuCgugcagaucgggac......................................................................   4      1   FF1
....................uUgcuuggugcagaucgggac......................................................................   6      1   FF1
....................ucgcuuUgugcagaucgggac......................................................................   4      1   FF1
....................ucgcuuggugcGgaucgggac......................................................................   11     1   FF1
....................ucgcuuggugcagauGgggac......................................................................   28     1   FF1
....................ucgcuuggugcagaucgggacA.....................................................................   9      1   FF1
....................ucgcuuggugcagaucgggacc.....................................................................   74     0   FF1
....................ucgcuuggugcagauGgggacc.....................................................................   1      1   FF1
....................Acgcuuggugcagaucgggacc.....................................................................   2      1   FF1
....................ucgcuuggCgcagaucgggacc.....................................................................   1      1   FF1
....................ucgcuuggugcaAaucgggacc.....................................................................   1      1   FF1
....................uUgcuuggugcagaucgggacc.....................................................................   1      1   FF1
....................ucgcuuggugcagaucgggacU.....................................................................   97     1   FF1
....................ucgcuuggugcagaucgggaccc....................................................................   1      0   FF1
....................ucgcuuggugcagaucgggaccU....................................................................   1      1   FF1
......................gcuuggugcagaucgggac......................................................................   2      0   FF1
.......................cuuggugcagaucgggacccu...................................................................   1      0   FF1
......................................................cgacgggccggaucccgccuugcaccaagGga.........................   1      1   FF1
........................................................acgggccggaucccgccuugcaccaagug..........................   1      0   FF1
..................................................................ucccgccuugcaccaagugaa........................   1      0   FF1
..................................................................Ccccgccuugcaccaagugaau.......................   1      1   FF1
..................................................................ucccgccuugcaccaagugaau.......................   1      0   FF1
...................................................................cccgccuugcaccaagugaU........................   1      1   FF1
...................................................................cccgccuugcaccaagugaa........................   32     0   FF1
...................................................................cccgccuugcacAaagugaau.......................   5      1   FF1
...................................................................cccgccuugcacUaagugaau.......................   4      1   FF1
...................................................................cccgccuugcaccaaAugaau.......................   3      1   FF1
...................................................................ccUgccuugcaccaagugaau.......................   1      1   FF1
...................................................................cAcgccuugcaccaagugaau.......................   4      1   FF1
...................................................................cccgccuugcaccaagugaGu.......................   1      1   FF1
...................................................................cccgccuugcaccaagGgaau.......................   5      1   FF1
...................................................................cUcgccuugcaccaagugaau.......................   2      1   FF1
...................................................................cccgccuugcaccaaguCaau.......................   1      1   FF1
...................................................................cccgccuugcacGaagugaau.......................   4      1   FF1
...................................................................cccgccuugcaccaagugaau.......................   1973   0   FF1
...................................................................cccgccuugcaccaagugUau.......................   2      1   FF1
...................................................................cccgccuugcaccaagugaaA......................   1      1   FF1
```

```
                    Mature                                        Star
ccgucgccgccgccucgggcucgcuuggugcagaucgggacccuccgcccgccccgacgggccggaucccgccuugcaccaagugaaucggagccggcgcagcgaccucgc
...................................................................cccgccuugcaccaagAgaau.......................    2     1     FF1
...................................................................cccgccuugcaccaagCgaau.......................    5     1     FF1
...................................................................cccUccuugcaccaagugaau.......................    1     1     FF1
...................................................................cccgccuugcaccaaguAaau.......................    3     1     FF1
...................................................................ccGgccuugcaccaagugaau.......................    2     1     FF1
...................................................................cccgccCugcaccaagugaau.......................    1     1     FF1
...................................................................Accgccuugcaccaagugaau.......................    4     1     FF1
...................................................................cccCccuugcaccaagugaau.......................    1     1     FF1
...................................................................cccgccuugcaccaaguUaau.......................    2     1     FF1
...................................................................cGcgccuugcaccaagugaau.......................    4     1     FF1
...................................................................cccgccuugcGccaagugaau.......................    1     1     FF1
...................................................................Gccgccuugcaccaagugaau.......................    4     1     FF1
...................................................................Uccgccuugcaccaagugaau.......................    4     1     FF1
...................................................................cccgccuugcaccaagugaauU......................    11    1     FF1
...................................................................cccgccuugcaccaagugaauA......................    2     1     FF1
....................................................................ccgccuugcaccaagugaa........................    2     0     FF1
....................................................................ccgccuugcaccaagugaau.......................    142   0     FF1
....................................................................ccgccuugcaGcaagugaau.......................    1     1     FF1
....................................................................cGgccuugcaccaagugaau.......................    1     1     FF1
....................................................................Ucgccuugcaccaagugaau.......................    1     1     FF1
....................................................................cUgccuugcaccaagugaau.......................    2     1     FF1
....................................................................ccgccuugcaccaaguAaau.......................    1     1     FF1
....................................................................ccgccuugcaccaagugaauA......................    1     1     FF1
........................................................................cuugcaccaagugaaucggag..................    1     0     FF1
.............................................................................accaagugaaucggagccggcgca..........    1     0     FF1
```
