# Supplementary material for: Identification of Novel miRNAs and miRNA Expression Profiling in Wheat Hybrid Necrosis
Source: PLoS One. 2015 Feb 23;10(2):e0117507. doi: 10.1371/journal.pone.0117507 (PMC4338152; doi:10.1371/journal.pone.0117507)
Supplement: S2 Fig — Red colored letter: mature miRNA sequence; yellow colored letter: loop sequence; blue colored letter: miRNA* sequence. (ZIP) [file pone.0117507.s002.zip › Figures s1/contig862671_9103.pdf]

Provisional ID	:	contig862671_9103
Score total	:	925.2
Score for star read(s)	:	3.9
Score for read counts	:	917.8
Score for mfe	:	2.5
Score for randfold	:	1.6
Score for cons. seed	:	-0.6
Total read count	:	1812
Mature read count	:	1509
Loop read count	:	0
Star read count	:	303

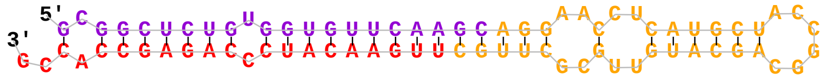

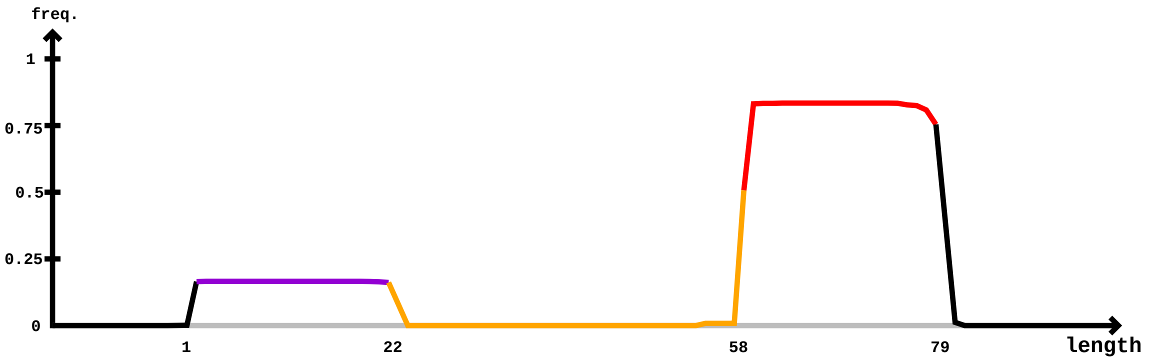


```
                      Star                                               Mature
5'- guuggggagcccggcggcucuggugguguucaagcaggaaccucaugcuaccggcagcauguugcgcuugcuugaacaucccagagccaccgggcgugccaaaauucacgc  -3'  obs
    guuggggagcccggcggcucuggugguguucaagcaggaaccucaugcuaccggcagcauguugcgcuugcuugaacaucccagagccaccgggcgugccaaaauucacgc       exp
    .((((...((((((.(((((((.((((((((((((((..(..((((((......))))))..)..)))))))))))))).))))))).)))).)).))))..........   reads   mm   sample
    .......................................................................uugaacaucccagagccac....................   1       0    NN8
    .......................................................................uugaacaucccUgagccacc...................   1       1    NN8
    .......................................................................uugaacaucccagagccacc...................   21      0    NN8
    .......................................................................uugaacaucccaUagccaccg..................   1       1    NN8
    .......................................................................uugaacaucccGgagccaccg..................   1       1    NN8
    .......................................................................Gugaacaucccagagccaccg..................   2       1    NN8
    .......................................................................uugaacaucccagagccaccg..................   115     0    NN8
    .......................................................................uugaacaucAcagagccaccg..................   1       1    NN8
    ........................................................................ugaacaucccagagccac....................   2       0    NN8
    ........................................................................ugaacaucccagagccacc...................   4       0    NN8
    ........................................................................ugaacaucccagagccaccg..................   3       0    NN8
    ........................................................................ugaacaucccagagccaccgg.................   13      0    NN8
    ........................................................................ugaacaucccaCagccaccgg.................   1       1    NN8
    ........................................................................ugaacaucccagagccaccgU.................   1       1    NN8
    ........................................................................ugaacaucccaAagccaccggc................   1       1    NN8
    .........................................................................gaacaucccagagccaccggc................   1       0    NN8
    ...........................................................................acaucccagagccaccgg.................   1       0    NN8

    ...........cggcggcucugugguguucaa..............................................................................   1       0    FF1
    ............ggcggcucugugguguucaag.............................................................................   1       0    FF1
    .............gcggcucugugguguuca...............................................................................   1       0    FF1
    .............gcggcucugugguguucaa..............................................................................   1       0    FF1
    .............gcggcucugugguguucaag.............................................................................   4       0    FF1
    .............gcggcucuguggCguucaagc............................................................................   2       1    FF1
    .............gAggcucugugguguucaagc............................................................................   1       1    FF1
    .............gcggcucuguAguguucaagc............................................................................   1       1    FF1
    .............gcggcucugugguguucaagc............................................................................   275     0    FF1
    .............gcggcucuguggAguucaagc............................................................................   4       1    FF1
    .............gUggcucugugguguucaagc............................................................................   1       1    FF1
    .............gcggcucuggugguGcaagc.............................................................................   2       1    FF1
    .............gcggcucugGgguguucaagc............................................................................   1       1    FF1
    .............gcggcucuguggGguucaagc............................................................................   3       1    FF1
    .............gcggcucuguggugGucaagc............................................................................   2       1    FF1
```

Star **Mature**

guuggggagcccggcggcucugugguguucaagcaggaaccucaugcuaccggcagcauguugcgcuugcuugaacaucccagagccaccggcgugccaaaauucacgc			
.............gcggcucugugguguucaagcU..	1	1	FF1
..............cggcucugugguguucaagc...	2	0	FF1
....uugcuugaacaucccagagc.......................	1	0	FF1
....uugcuugaacaucccagagcc......................	11	0	FF1
....uugcuugaacaucccagagccU.....................	3	1	FF1
....uugaacaucccagagccU.....................	2	1	FF1
....uugaacaucccagagccac....................	10	0	FF1
....uugaacaucccagagccaU....................	1	1	FF1
....uugaacaGcccagagccacc...................	1	1	FF1
....uugaacaucccagagccacc...................	39	0	FF1
....uugaacaucccaAagccacc...................	1	1	FF1
....uugaacaucccagagccaccC..................	4	1	FF1
....uugaacaucccaCagccaccg..................	2	1	FF1
....uugaacauGccagagccaccg..................	2	1	FF1
....uuUaacaucccagagccaccg..................	1	1	FF1
....uugUacaucccagagccaccg..................	1	1	FF1
....uugaacaucccagagUcaccg..................	1	1	FF1
....Gugaacaucccagagccaccg..................	1	1	FF1
....uugaacaucccagagccaccU..................	15	1	FF1
....uugaacaucccagagGcaccg..................	2	1	FF1
....uGgaacaucccagagccaccg..................	1	1	FF1
....Augaacaucccagagccaccg..................	1	1	FF1
....uugaacauAccagagccaccg..................	1	1	FF1
....uugaacaucccagagcAaccg..................	1	1	FF1
....uugaacaucccagagccaccg..................	675	0	FF1
....uugaacauUccagagccaccg..................	1	1	FF1
....uugaacaucccGgagccaccg..................	1	1	FF1
....uugaacaucccagagccaccgU.................	4	1	FF1
....uugaacaucccagagccaccgC.................	1	1	FF1
....ugaacaucccagagccac....................	13	0	FF1
....Ggaacaucccagagccac....................	1	1	FF1
....ugaacaucccagagccaU....................	2	1	FF1
....ugaacaucccagagccacc...................	28	0	FF1
....ugaacaucccagagccaUc...................	2	1	FF1
....Ggaacaucccagagccacc...................	1	1	FF1
....ugaacaucccagagccacU...................	1	1	FF1
....ugaacaucccagagccaccg..................	15	0	FF1
....ugaacaucccagagccaccU..................	5	1	FF1
....ugaacaucccagUgccaccgg.................	1	1	FF1
....uCaacaucccagagccaccgg.................	1	1	FF1
....ugaacaucccagagcGaccgg.................	1	1	FF1
....ugaacaucccagGgccaccgg.................	2	1	FF1
....ugaacaucccagagccCccgg.................	1	1	FF1
....ugaacaucccagagccacAgg.................	1	1	FF1
....Ggaacaucccagagccaccgg.................	2	1	FF1
....ugaacaucccagagccaccgA.................	2	1	FF1
....ugaacaucccUgagccaccgg.................	2	1	FF1
....ugaGcaucccagagccaccgg.................	1	1	FF1
....ugaacaucccaUagccaccgg.................	1	1	FF1
....ugaacaucccagagccaAcgg.................	1	1	FF1
....ugaacaucccagCgccaccgg.................	1	1	FF1
....ugaacaucccagagccaccgU.................	5	1	FF1
....ugaacauGccagagccaccgg.................	1	1	FF1
....ugaacaucccagagccaccgg.................	456	0	FF1
....Cgaacaucccagagccaccgg.................	2	1	FF1
....ugaacaucccagagccaccggc................	1	0	FF1
....ugaacaucccagagccaccggA................	12	1	FF1
....ugaacaucccagagccaccgUc................	1	1	FF1
....ugaacaucccagagccaccggU................	4	1	FF1
....gaacaucccagagccaccggc................	2	0	FF1
....acaucccagagccaccgg.................	1	0	FF1
