# Supplementary material for: Identification of Novel miRNAs and miRNA Expression Profiling in Wheat Hybrid Necrosis
Source: PLoS One. 2015 Feb 23;10(2):e0117507. doi: 10.1371/journal.pone.0117507 (PMC4338152; doi:10.1371/journal.pone.0117507)
Supplement: S2 Fig — Red colored letter: mature miRNA sequence; yellow colored letter: loop sequence; blue colored letter: miRNA* sequence. (ZIP) [file pone.0117507.s002.zip › Figures s1/contig863626_9114.pdf]

Provisional ID	:	contig863626_9114
Score total	:	782.4
Score for star read(s)	:	3.9
Score for read counts	:	775.1
Score for mfe	:	2.4
Score for randfold	:	1.6
Score for cons. seed	:	-0.6
Total read count	:	1532
Mature read count	:	1509
Loop read count	:	0
Star read count	:	23

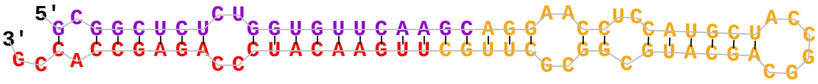

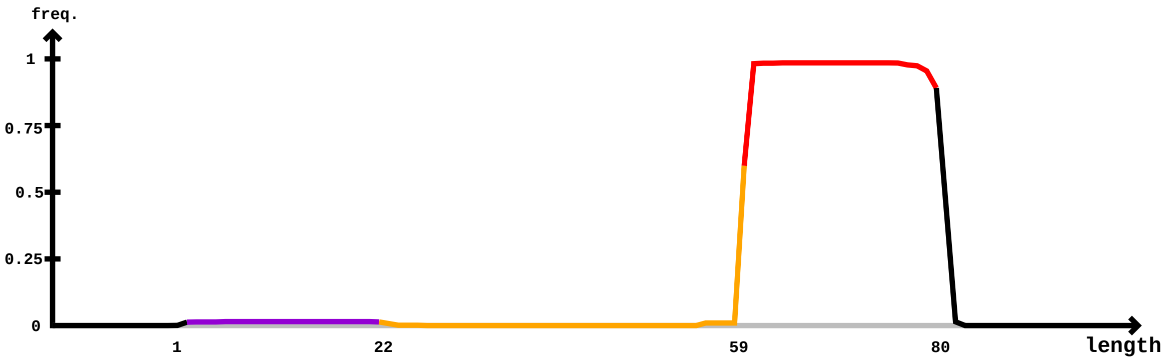

Star **Mature**

```
5'- ucggggagcccggcggcucucugguguucaagcaggaaccuccaugcuaccggcagcaugcggcgcuugcuugaacaucccagagccaccggcgugccaaauucacgcagg  -3'  obs
    ucggggagcccggcggcucucugguguucaagcaggaaccuccaugcuaccggcagcaugcggcgcuugcuugaacaucccagagccaccggcgugccaaauucacgcagg       exp
    ....((.((((((.((((((..((((((((((((((..((..((((((......)))))).))..))))))))))))))..)))))).)))).)).))............  reads  mm  sample
    ......................................................................uugaacaucccagagccac......................  1      0   NN8
    ......................................................................uugaacaucccagagccacc.....................  21     0   NN8
    ......................................................................uugaacaucccUgagccacc.....................  1      1   NN8
    ......................................................................uugaacaucAcagagccaccg....................  1      1   NN8
    ......................................................................uugaacaucccGgagccaccg....................  1      1   NN8
    ......................................................................uugaacaucccaUagccaccg....................  1      1   NN8
    ......................................................................uugaacaucccagagccaccg....................  115    0   NN8
    ......................................................................Gugaacaucccagagccaccg....................  2      1   NN8
    .......................................................................ugaacaucccagagccac......................  2      0   NN8
    .......................................................................ugaacaucccagagccacc.....................  4      0   NN8
    .......................................................................ugaacaucccagagccaccg....................  3      0   NN8
    .......................................................................ugaacaucccagagccaccgg...................  13     0   NN8
    .......................................................................ugaacaucccaCagccaccgg...................  1      1   NN8
    .......................................................................ugaacaucccagagccaccgU...................  1      1   NN8
    .......................................................................ugaacaucccaAagccaccggc..................  1      1   NN8
    ........................................................................gaacaucccagagccaccggc..................  1      0   NN8
    ..........................................................................acaucccagagccaccgg...................  1      0   NN8

    ...........ggcggcucucugguguucaag...............................................................................  1      0   FF1
    ............gcggcucucugguguucaag...............................................................................  1      0   FF1
    ............Acggcucucugguguucaagc..............................................................................  1      1   FF1
    ............gcggcucucugguguucaagc..............................................................................  17     0   FF1
    .............cggcucucugguguucaagc..............................................................................  1      0   FF1
    ................cucucugguguucaagcagga..........................................................................  2      0   FF1
    ..................................................................uugcuugaacaucccagagc.........................  1      0   FF1
    ..................................................................uugcuugaacaucccagagcc........................  11     0   FF1
    ..................................................................uugcuugaacaucccagagccU.......................  3      1   FF1
    ......................................................................uugaacaucccagagccU.......................  2      1   FF1
    ......................................................................uugaacaucccagagccac......................  10     0   FF1
    ......................................................................uugaacaucccagagccaU......................  1      1   FF1
    ......................................................................uugaacaucccagagccacc.....................  39     0   FF1
    ......................................................................uugaacaGcccagagccacc.....................  1      1   FF1
    ......................................................................uugaacaucccaAagccacc.....................  1      1   FF1
```

```
            Star                                                         Mature
ucggggagcccggcggcucucugguguucaagcaggaaccuccaugcuaccggcagcaugcggcgcuugcuugaacaucccagagccaccggcgugccaaauucacgcagg
......................................................................Augaacaucccagagccaccg....................    1     1     FF1
......................................................................uugUacaucccagagccaccg....................    1     1     FF1
......................................................................uugaacaucccagagGcaccg....................    2     1     FF1
......................................................................uugaacaucccGgagccaccg....................    1     1     FF1
......................................................................uugaacaucccagagccaccC....................    4     1     FF1
......................................................................uugaacaucccagagccaccU....................    15    1     FF1
......................................................................Gugaacaucccagagccaccg....................    1     1     FF1
......................................................................uugaacauUccagagccaccg....................    1     1     FF1
......................................................................uGgaacaucccagagccaccg....................    1     1     FF1
......................................................................uugaacauGccagagccaccg....................    2     1     FF1
......................................................................uuUaacaucccagagccaccg....................    1     1     FF1
......................................................................uugaacauAccagagccaccg....................    1     1     FF1
......................................................................uugaacaucccagagUcaccg....................    1     1     FF1
......................................................................uugaacaucccaCagccaccg....................    2     1     FF1
......................................................................uugaacaucccagagcAaccg....................    1     1     FF1
......................................................................uugaacaucccagagccaccg....................    675   0     FF1
......................................................................uugaacaucccagagccaccgC...................    1     1     FF1
......................................................................uugaacaucccagagccaccgU...................    4     1     FF1
.......................................................................ugaacaucccagagccac......................    13    0     FF1
.......................................................................ugaacaucccagagccaU......................    2     1     FF1
.......................................................................Ggaacaucccagagccac......................    1     1     FF1
.......................................................................ugaacaucccagagccacU.....................    1     1     FF1
.......................................................................ugaacaucccagagccacc.....................    28    0     FF1
.......................................................................ugaacaucccagagccaUc.....................    2     1     FF1
.......................................................................Ggaacaucccagagccacc.....................    1     1     FF1
.......................................................................ugaacaucccagagccaccg....................    15    0     FF1
.......................................................................ugaacaucccagagccaccU....................    5     1     FF1
.......................................................................ugaacaucccagagccaccgg...................    456   0     FF1
.......................................................................ugaacaucccagGgccaccgg...................    2     1     FF1
.......................................................................ugaacaucccUgagccaccgg...................    2     1     FF1
.......................................................................Ggaacaucccagagccaccgg...................    2     1     FF1
.......................................................................ugaacaucccagagccCccgg...................    1     1     FF1
.......................................................................ugaacaucccaUagccaccgg...................    1     1     FF1
.......................................................................ugaacaucccagagccaccgA...................    2     1     FF1
.......................................................................ugaacaucccagCgccaccgg...................    1     1     FF1
.......................................................................ugaacaucccagagccaccgU...................    5     1     FF1
.......................................................................ugaacaucccagagccaAcgg...................    1     1     FF1
.......................................................................ugaacaucccagagcGaccgg...................    1     1     FF1
.......................................................................Cgaacaucccagagccaccgg...................    2     1     FF1
.......................................................................ugaacauGccagagccaccgg...................    1     1     FF1
.......................................................................ugaacaucccagagccacAgg...................    1     1     FF1
.......................................................................uCaacaucccagagccaccgg...................    1     1     FF1
.......................................................................ugaGcaucccagagccaccgg...................    1     1     FF1
.......................................................................ugaacaucccagUgccaccgg...................    1     1     FF1
.......................................................................ugaacaucccagagccaccggc..................    1     0     FF1
.......................................................................ugaacaucccagagccaccggU..................    4     1     FF1
.......................................................................ugaacaucccagagccaccggA..................    12    1     FF1
.......................................................................ugaacaucccagagccaccgUc..................    1     1     FF1
........................................................................gaacaucccagagccaccggc..................    2     0     FF1
..........................................................................acaucccagagccaccgg...................    1     0     FF1
```
