# Supplementary material for: Identification of Novel miRNAs and miRNA Expression Profiling in Wheat Hybrid Necrosis
Source: PLoS One. 2015 Feb 23;10(2):e0117507. doi: 10.1371/journal.pone.0117507 (PMC4338152; doi:10.1371/journal.pone.0117507)
Supplement: S2 Fig — Red colored letter: mature miRNA sequence; yellow colored letter: loop sequence; blue colored letter: miRNA* sequence. (ZIP) [file pone.0117507.s002.zip › Figures s1/contig881186_9199.pdf]

Provisional ID	:	contig881186_9199
Score total	:	1.1
Score for star read(s)	:	-1.3
Score for read counts	:	0
Score for mfe	:	1.6
Score for randfold	:	-2.2
Score for cons. seed	:	3
Total read count	:	28
Mature read count	:	28
Loop read count	:	0
Star read count	:	0

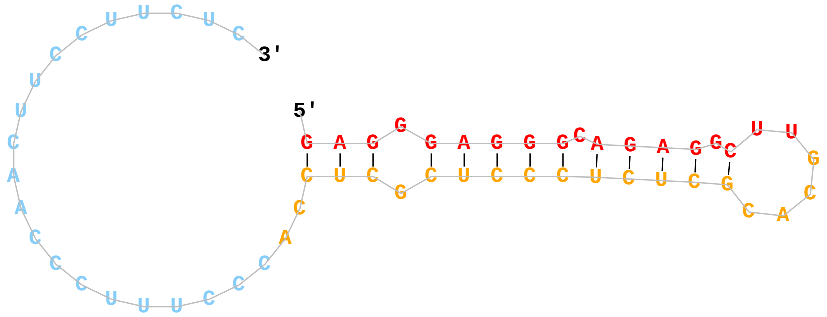

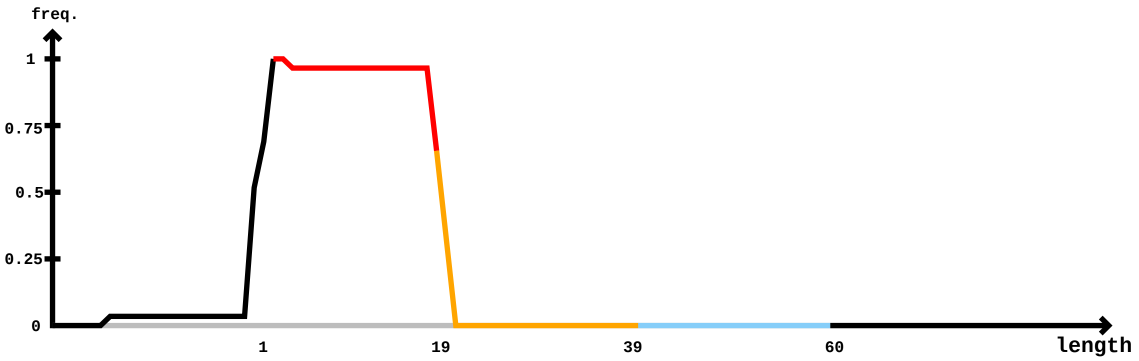

Mature Star

```
5'- ggccuauaaauagggggaggagagggagggcagaggcuugcacgcucucccucgcuccacccuuucccaacuuccuucuccuuccuuccgccgccgcagcgccccauu -3'    exp
    (((.........((((((((((((((((((.(((((..((...((........))..)).))))))))....)))))))))))))))..((....))...))).....      reads    mm    sample
    ....uauaaauaAggggaggaga.....................................................................................      1        1     NN8
    ...................gagagggagggcagaggcu......................................................................      1        0     NN8
    ...................gagagggagggcagaggcC......................................................................      6        1     NN8
    ...................gagagggagggcagaggcCu.....................................................................      7        1     NN8
    ....................agagggagggcagaggcC......................................................................      2        1     NN8
    ....................agagggagggcagaggcCu.....................................................................      3        1     NN8
    .....................gagggagggcagaggcCu.....................................................................      8        1     NN8
    .....................gagggagggcagaggcuu.....................................................................      1        0     NN8
```
