# Supplementary material for: Identification of Novel miRNAs and miRNA Expression Profiling in Wheat Hybrid Necrosis
Source: PLoS One. 2015 Feb 23;10(2):e0117507. doi: 10.1371/journal.pone.0117507 (PMC4338152; doi:10.1371/journal.pone.0117507)
Supplement: S2 Fig — Red colored letter: mature miRNA sequence; yellow colored letter: loop sequence; blue colored letter: miRNA* sequence. (ZIP) [file pone.0117507.s002.zip › Figures s1/contig923344_9386.pdf]

Provisional ID : contig923344_9386
Score total : 125.1
Score for star read(s) : 3.9
Score for read counts : 117.9
Score for mfe : 2.3
Score for randfold : 1.6
Score for cons. seed : -0.6
Total read count : 243
Mature read count : 242
Loop read count : 0
Star read count : 1

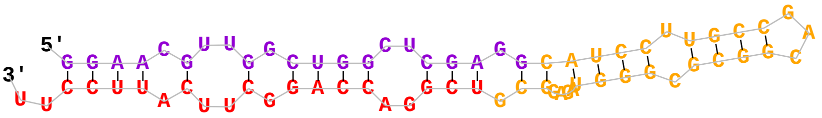

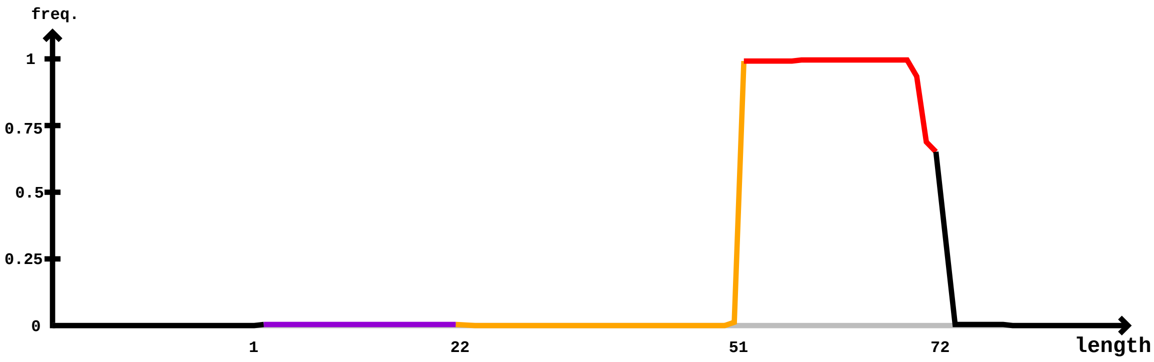

Star Mature

```
5'- guggccauggcgguuuguggggaacguuggcuggcucgaggcauccuugccgacggcgcggguagaggcgucggaccaggcuucauuccuuucaaaccgccgcguccauc  -3'  obs
    guggccauggcgguuuguggggaacguuggcuggcucgaggcauccuugccgacggcgcggguagaggcgucggaccaggcuucauuccuuucaaaccgccgcguccauc       exp
    ((((.(.((((((((((..(((((.(..(.((((..(((.((((((.((((...)))).))))....)).)))..)))).)..).))))).)))))))))))).).)))).        reads   mm   sample
    ......................................................................ucggaccaggcuucauuc......................        2       0    NN8
    ......................................................................ucggaccaggcuucauuccuu...................        2       0    NN8

    ....................ggaacguuggcuggcucgagg.....................................................................        1       0    FF1
    .....................................................................Uucggaccaggcuucauuccu....................        1       1    FF1
    .....................................................................gucggaccaggcuucauuccG....................        2       1    FF1
    ......................................................................ucggaccaggcuucauuU......................        1       1    FF1
    ......................................................................ucggaccaggcuucauuc......................        12      0    FF1
    ......................................................................ucggaccaggcuucauucc.....................        54      0    FF1
    ......................................................................ucggaccaggcuucaGucc.....................        1       1    FF1
    ......................................................................ucggaccaggcuucauCcc.....................        1       1    FF1
    ......................................................................ucggaccaggcuucauucU.....................        3       1    FF1
    ......................................................................ucggGccaggcuucauucc.....................        1       1    FF1
    ......................................................................ucggaccaggcuucauuccu....................        6       0    FF1
    ......................................................................ucggaccaggcuucauuUcuu...................        1       1    FF1
    ......................................................................ucggaccaggcuucauuccuu...................        145     0    FF1
    ......................................................................ucggaccaggcuAcauuccuu...................        1       1    FF1
    ......................................................................ucggaccaggcuucauuccuC...................        7       1    FF1
    ......................................................................ucggaccaggcuucauuccuuu..................        2       0    FF1
    ............................................................................caggcuucauuccuuucaaacc............        1       0    FF1
```
