# Supplementary material for: Identification of Novel miRNAs and miRNA Expression Profiling in Wheat Hybrid Necrosis
Source: PLoS One. 2015 Feb 23;10(2):e0117507. doi: 10.1371/journal.pone.0117507 (PMC4338152; doi:10.1371/journal.pone.0117507)
Supplement: S2 Fig — Red colored letter: mature miRNA sequence; yellow colored letter: loop sequence; blue colored letter: miRNA* sequence. (ZIP) [file pone.0117507.s002.zip › Figures s1/contig936343_9448.pdf]

Provisional ID	:	contig936343_9448
Score total	:	2476.3
Score for star read(s)	:	3.9
Score for read counts	:	2465.6
Score for mfe	:	2.2
Score for randfold	:	1.6
Score for cons. seed	:	3
Total read count	:	4848
Mature read count	:	4807
Loop read count	:	0
Star read count	:	41

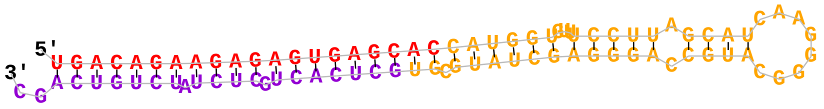

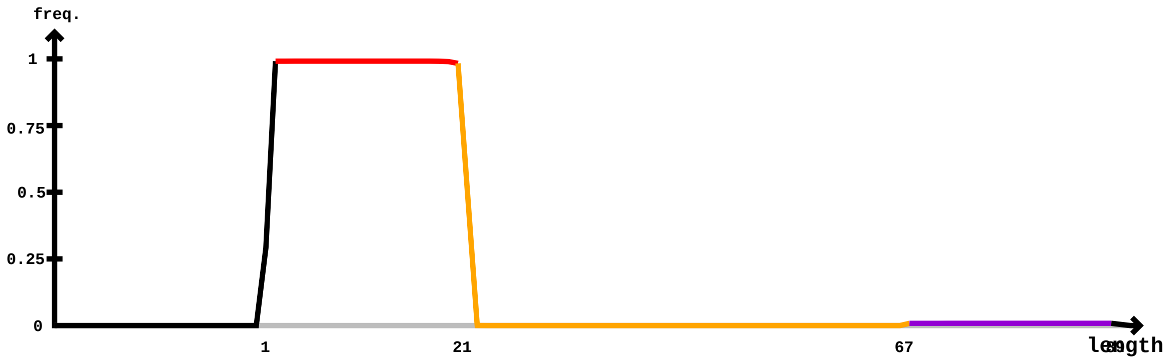

Mature **Star**

```
5'- gugggugcuguguguggaaguugacagaagagagugagcaccaugguguuuccuuagcaucaaggggcaugccagggagcuaugcgugcucacugcucuaucugucagcca   -3'   obs
    gugggugcuguguguggaaguugacagaagagagugagcaccaugguguuuccuuagcaucaaggggcaugccagggagcuaugcgugcucacugcucuaucugucagcca         exp
    ...................((((((((((((((((((((((((((((...(((((.((((........)))).))))))))))).))))))))).)))).)))))))))..   reads   mm   sample
    ....................uugacagaagagagugaUcac......................................................................   1       1    NN8
    ....................uugacagaagagagugagcac......................................................................   12      0    NN8
    ....................uugacagaagagagugagcacU.....................................................................   1       1    NN8
    .....................ugacagaagagagugagc........................................................................   1       0    NN8
    .....................ugacagaagagagugagca.......................................................................   2       0    NN8
    .....................ugacagaagagGgugagcac......................................................................   1       1    NN8
    .....................ugacagaagagagugUgcac......................................................................   1       1    NN8
    .....................ugacagaagagagugagcaU......................................................................   1       1    NN8
    .....................Agacagaagagagugagcac......................................................................   1       1    NN8
    .....................ugacagaagagagugagcac......................................................................   135     0    NN8
    .....................ugacagaagagagugagcacU.....................................................................   11      1    NN8
    .......................acagaagagagugagcac......................................................................   1       0    NN8
    .......................................................................................gcucacugcucuaucugucagc..   14      0    NN8
    .......................................................................................gcucacuCcucuaucugucagc..   1       1    NN8
    .......................................................................................gGucacugcucuaucugucagc..   1       1    NN8
    ...........................................................................................acugcucuaucugucagc..   1       0    NN8

    ....................uugacagaagagagugag.........................................................................   2       0    FF1
    ....................uugacagaagagagugagc........................................................................   2       0    FF1
    ....................uugacagaagagagugagca.......................................................................   5       0    FF1
    ....................uugacagaagagGgugagca.......................................................................   1       1    FF1
    ....................uugacagaagagagugagcaA......................................................................   1       1    FF1
    ....................uugacagaagagagugagAac......................................................................   2       1    FF1
    ....................Augacagaagagagugagcac......................................................................   2       1    FF1
    ....................uuAacagaagagagugagcac......................................................................   1       1    FF1
    ....................uugacagaagagagGgagcac......................................................................   12      1    FF1
    ....................uugacagaagagagugaUcac......................................................................   1       1    FF1
    ....................uugacagaagagaguAagcac......................................................................   3       1    FF1
    ....................uugacaAaagagagugagcac......................................................................   2       1    FF1
    ....................uugaGagaagagagugagcac......................................................................   1       1    FF1
    ....................uugacagaagagagugagcac......................................................................   1307    0    FF1
    ....................uugacagaagagCgugagcac......................................................................   3       1    FF1
    ....................uugacagaagagagugaAcac......................................................................   4       1    FF1
```

Mature **Star**

```
gugggugcuguguguggaaguugacagaagagagugagcaccauggguguuuccuuagcaucaaggggcaugccagggagcuaugcgugcucacugcucuaucugucagcca
....................uugacagaagagagugagcaU.......................................................................   3     1   FF1
....................uugCcagaagagagugagcac.......................................................................   1     1   FF1
....................uugacUgaagagagugagcac.......................................................................   1     1   FF1
....................uugacagaagagagAgagcac.......................................................................   1     1   FF1
....................uuCacagaagagagugagcac.......................................................................   1     1   FF1
....................uugacagaagagagugagGac.......................................................................   1     1   FF1
....................uugacagaagagGgugagcac.......................................................................   2     1   FF1
....................uugacagaCgagagugagcac.......................................................................   1     1   FF1
....................uugaUagaagagagugagcac.......................................................................   3     1   FF1
....................uugacagaagagaguUagcac.......................................................................   1     1   FF1
....................uugGcagaagagagugagcac.......................................................................   1     1   FF1
....................uugacagaagagagCgagcac.......................................................................   3     1   FF1
....................uugacagaagagagugGgcac.......................................................................   1     1   FF1
....................uugacagaagagagugaCcac.......................................................................   2     1   FF1
....................uugacagaagagUgugagcac.......................................................................   4     1   FF1
....................uugacagaagagagugagcacU......................................................................   20    1   FF1
....................uugacagaagagagugagcacc......................................................................   4     0   FF1
.....................ugacagaagagagugagc.........................................................................   3     0   FF1
.....................ugacagaagagaCugagca........................................................................   1     1   FF1
.....................ugCcagaagagagugagca........................................................................   1     1   FF1
.....................Ggacagaagagagugagca........................................................................   1     1   FF1
.....................ugacagaagagagugagca........................................................................   22    0   FF1
.....................Ggacagaagagagugagcac.......................................................................   3     1   FF1
.....................ugacagaagagagAgagcac.......................................................................   2     1   FF1
.....................ugacagaagagagugCgcac.......................................................................   2     1   FF1
.....................ugacagaagagGgugagcac.......................................................................   2     1   FF1
.....................ugacagaagagagugGgcac.......................................................................   11    1   FF1
.....................ugacagCagagagugagcac.......................................................................   1     1   FF1
.....................ugacagaagagagugaCcac.......................................................................   1     1   FF1
.....................ugacagaaCagagugagcac.......................................................................   1     1   FF1
.....................ugacagaagagagugagcac.......................................................................   2949  0   FF1
.....................ugacagaagagagugaUcac.......................................................................   1     1   FF1
.....................ugacagaagagaAugagcac.......................................................................   3     1   FF1
.....................ugacagaagagaUugagcac.......................................................................   7     1   FF1
.....................uAacagaagagagugagcac.......................................................................   1     1   FF1
.....................ugacagaagagagugaAcac.......................................................................   1     1   FF1
.....................ugacagaagagagugagcaA.......................................................................   2     1   FF1
.....................ugacagGagagagugagcac.......................................................................   2     1   FF1
.....................ugacagaagagagugagcCc.......................................................................   2     1   FF1
.....................ugGcagaagagagugagcac.......................................................................   2     1   FF1
.....................ugacagaaAagagugagcac.......................................................................   3     1   FF1
.....................ugaGagaagagagugagcac.......................................................................   1     1   FF1
.....................ugacagaagagCgugagcac.......................................................................   1     1   FF1
.....................uUacagaagagagugagcac.......................................................................   2     1   FF1
.....................Agacagaagagagugagcac.......................................................................   1     1   FF1
.....................ugacagaagaAagugagcac.......................................................................   1     1   FF1
.....................ugacagaagagagugagcaU.......................................................................   11    1   FF1
.....................ugaUagaagagagugagcac.......................................................................   1     1   FF1
.....................ugacagaagagagugagAac.......................................................................   5     1   FF1
.....................ugacagaagagagGgagcac.......................................................................   8     1   FF1
.....................ugacagaagagagugagcGc.......................................................................   1     1   FF1
.....................ugacagaagagagugagGac.......................................................................   6     1   FF1
.....................ugacagaGgagagugagcac.......................................................................   1     1   FF1
.....................ugacagaagagaCugagcac.......................................................................   2     1   FF1
.....................ugaAagaagagagugagcac.......................................................................   4     1   FF1
.....................ugacagaagagagugagcacc......................................................................   18    0   FF1
.....................ugacagaagagagugagcaAc......................................................................   1     1   FF1
.....................ugacagaagagagugagcacU......................................................................   151   1   FF1
.....................ugacagaagagagugagcaccU.....................................................................   1     1   FF1
........................................................................................gcucacugcucuaucugucagc..   22    0   FF1
........................................................................................gcucacuCcucuaucugucagc..   1     1   FF1
........................................................................................gcucacugcucuaucugGcagc..   1     1   FF1
```
