# Supplementary material for: Identification of Novel miRNAs and miRNA Expression Profiling in Wheat Hybrid Necrosis
Source: PLoS One. 2015 Feb 23;10(2):e0117507. doi: 10.1371/journal.pone.0117507 (PMC4338152; doi:10.1371/journal.pone.0117507)
Supplement: S2 Fig — Red colored letter: mature miRNA sequence; yellow colored letter: loop sequence; blue colored letter: miRNA* sequence. (ZIP) [file pone.0117507.s002.zip › Figures s1/contig936343_9449.pdf]

Provisional ID	:	contig936343_9449
Score total	:	2103
Score for star read(s)	:	3.9
Score for read counts	:	2091.9
Score for mfe	:	2.6
Score for randfold	:	1.6
Score for cons. seed	:	3
Total read count	:	4115
Mature read count	:	3427
Loop read count	:	0
Star read count	:	688

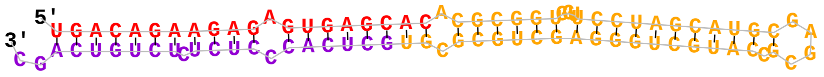

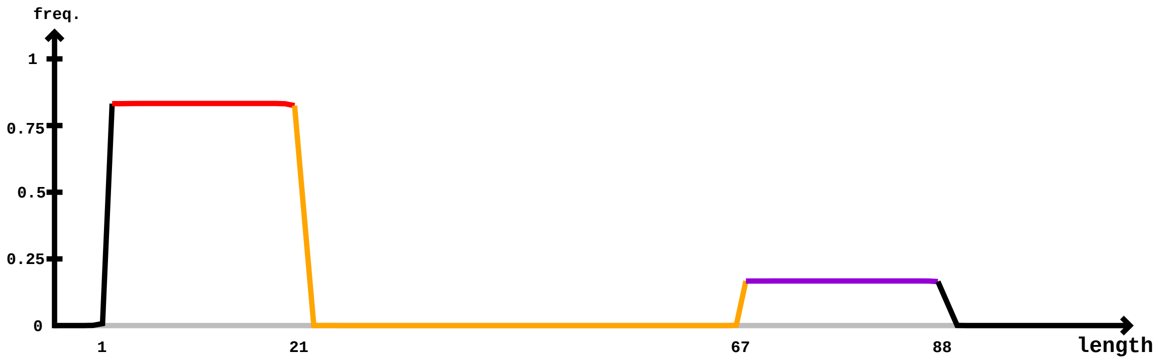

Mature Star

```
5'- ggucugacagaagagagugagcacacgcggugguuccuagcaugcgagcgccaugcugggagcugcgcgugcucacccucucucugucagccauucaccaugcccaucuc  -3'    obs
    ggucugacagaagagagugagcacacgcggugguuccuagcaugcgagcgccaugcugggagcugcgcgugcucacccucucucugucagccauucaccaugcccaucuc          exp
    ((.((((((((((((.((((((((.((((((...(((((((((((....).)))))))))))))))))).)))))))).)))).))))))))))..................   reads    mm     sample
    ....ugacagaagagagugagc........................................................................................   1        0      NN8
    ....ugacagaagagagugagca.......................................................................................   2        0      NN8
    ....Agacagaagagagugagcac......................................................................................   1        1      NN8
    ....ugacagaagagagugUgcac......................................................................................   1        1      NN8
    ....ugacagaagagagugagcaU......................................................................................   1        1      NN8
    ....ugacagaagagagugagcac......................................................................................   135      0      NN8
    ....ugacagaagagGgugagcac......................................................................................   1        1      NN8
    ....ugacagaagagagugagcacU.....................................................................................   11       1      NN8
    ......acagaagagagugagcac......................................................................................   1        0      NN8
    .....................................................................Cgcucacccucucucugucagcc..................   3        1      NN8
    ......................................................................gcucacccucucucuguca.....................   1        0      NN8
    ......................................................................gcucacccucucucugucag....................   5        0      NN8
    ......................................................................gcucGcccucucucugucagc...................   1        1      NN8
    ......................................................................gcucacccucucucugucagc...................   412      0      NN8
    ......................................................................gcucacccucucucuUucagc...................   1        1      NN8
    ......................................................................gcucacccucucucugucGgc...................   1        1      NN8
    ......................................................................gcucaUccucucucugucagc...................   1        1      NN8
    ......................................................................gcucacccucGcucugucagc...................   1        1      NN8
    ......................................................................gcucacccucucucugucagcG..................   1        1      NN8
    ......................................................................gcucacccucucucugucagcc..................   6        0      NN8
    .........................................................................cacccucucucugucagc...................   1        0      NN8

    ..ucugacagaagagagugagcac......................................................................................   3        0      FF1
    ...cugacagaagagagugagca.......................................................................................   2        0      FF1
    ...Augacagaagagagugagcac......................................................................................   2        1      FF1
    ...cugacagaagagagugagcac......................................................................................   21       0      FF1
    ...cGgacagaagagagugagcac......................................................................................   1        1      FF1
    ...cugacagaagagUgugagcac......................................................................................   1        1      FF1
    ....ugacagaagagagugagc........................................................................................   3        0      FF1
    ....ugacagaagagaCugagca.......................................................................................   1        1      FF1
    ....ugacagaagagagugagca.......................................................................................   22       0      FF1
    ....Ggacagaagagagugagca.......................................................................................   1        1      FF1
    ....ugCcagaagagagugagca.......................................................................................   1        1      FF1
```

Mature **Star**

```
ggucugacagaagagagugagcacacgcggugguuccuagcaugcgagcgccaugcugggagcugcgcgugcucacccucucucugucagccauucaccaugcccaucuc
....ugGcagaagagagugagcac......................................................................................   2     1   FF1
....ugaGagaagagagugagcac......................................................................................   1     1   FF1
....ugacagaagaAagugagcac......................................................................................   1     1   FF1
....ugacagaagagagGgagcac......................................................................................   8     1   FF1
....ugacagaagagagugagcGc......................................................................................   1     1   FF1
....ugacagaagagagAgagcac......................................................................................   2     1   FF1
....ugacagaGgagagugagcac......................................................................................   1     1   FF1
....ugacagaagagaAugagcac......................................................................................   3     1   FF1
....Agacagaagagagugagcac......................................................................................   1     1   FF1
....ugacagCagagagugagcac......................................................................................   1     1   FF1
....ugaUagaagagagugagcac......................................................................................   1     1   FF1
....ugacagaagagagugaAcac......................................................................................   1     1   FF1
....uUacagaagagagugagcac......................................................................................   2     1   FF1
....ugacagaagagagugagAac......................................................................................   5     1   FF1
....ugacagaagagagugagcac......................................................................................   2949  0   FF1
....ugacagGagagagugagcac......................................................................................   2     1   FF1
....ugacagaagagaUugagcac......................................................................................   7     1   FF1
....uAacagaagagagugagcac......................................................................................   1     1   FF1
....ugacagaagagGgugagcac......................................................................................   2     1   FF1
....Ggacagaagagagugagcac......................................................................................   3     1   FF1
....ugacagaagagagugagGac......................................................................................   6     1   FF1
....ugacagaagagagugCgcac......................................................................................   2     1   FF1
....ugacagaagagagugagcaA......................................................................................   2     1   FF1
....ugacagaagagagugagcaU......................................................................................   11    1   FF1
....ugaAagaagagagugagcac......................................................................................   4     1   FF1
....ugacagaagagagugaUcac......................................................................................   1     1   FF1
....ugacagaagagagugaCcac......................................................................................   1     1   FF1
....ugacagaagagagugGgcac......................................................................................   11    1   FF1
....ugacagaagagagugagcCc......................................................................................   2     1   FF1
....ugacagaagagCgugagcac......................................................................................   1     1   FF1
....ugacagaaCagagugagcac......................................................................................   1     1   FF1
....ugacagaagagaCugagcac......................................................................................   2     1   FF1
....ugacagaaAagagugagcac......................................................................................   3     1   FF1
....ugacagaagagagugagcaca.....................................................................................   21    0   FF1
....ugacagaagagagugagcacU.....................................................................................   151   1   FF1
......acagaagagagugagcacacgU..................................................................................   1     1   FF1
.......cagaagagagugagcaca.....................................................................................   1     0   FF1
.....................................................................ugcucacccucucucugucagc...................   1     0   FF1
......................................................................gcucacccucucucugucag....................   3     0   FF1
......................................................................gcucacccucucGcugucagc...................   3     1   FF1
......................................................................gcucacGcucucucugucagc...................   1     1   FF1
......................................................................Acucacccucucucugucagc...................   2     1   FF1
......................................................................gcucaccGucucucugucagc...................   1     1   FF1
......................................................................gcucacccucucucuAucagc...................   1     1   FF1
......................................................................gcucacccucucucugucagc...................   231   0   FF1
......................................................................gcucacccucucucugucagcc..................   9     0   FF1
......................................................................gcucacccucucucugucagccU.................   2     1   FF1
```
