# Supplementary material for: Identification of Novel miRNAs and miRNA Expression Profiling in Wheat Hybrid Necrosis
Source: PLoS One. 2015 Feb 23;10(2):e0117507. doi: 10.1371/journal.pone.0117507 (PMC4338152; doi:10.1371/journal.pone.0117507)
Supplement: S2 Fig — Red colored letter: mature miRNA sequence; yellow colored letter: loop sequence; blue colored letter: miRNA* sequence. (ZIP) [file pone.0117507.s002.zip › Figures s1/contig94531_2506.pdf]

Provisional ID	:	contig94531_2506
Score total	:	107
Score for star read(s)	:	3.9
Score for read counts	:	99.5
Score for mfe	:	2.5
Score for randfold	:	1.6
Score for cons. seed	:	-0.6
Total read count	:	207
Mature read count	:	205
Loop read count	:	0
Star read count	:	2

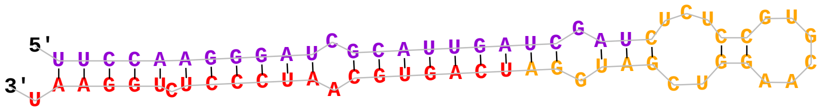

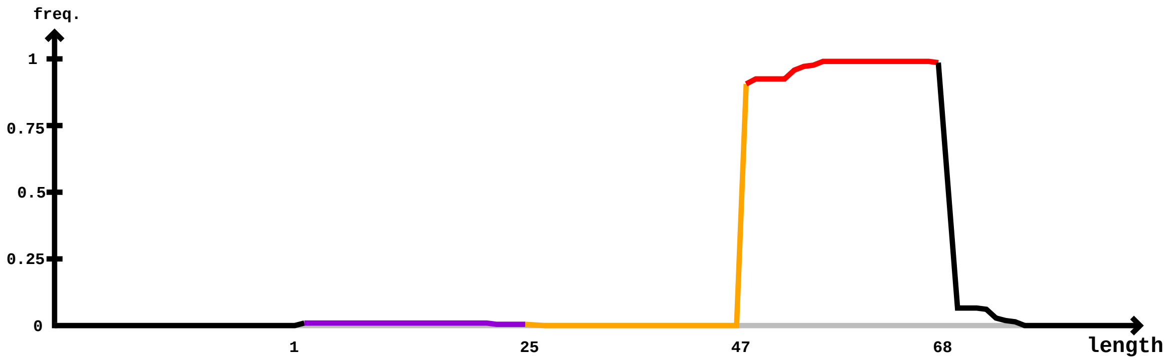

Star Mature

```
5'- agcuuucaggaagcuaguggaggauuccaagggaucgcauugaucgaucucuccgugcaaggucgauggaucagugcaaucccucuggaauucuccgcuugccuccgccuc   -3'    obs
    agcuuucaggaagcuaguggaggauuccaagggaucgcauugaucgaucucuccgugcaaggucgauggaucagugcaaucccucuggaauucuccgcuugccuccgccuc          exp
    ........(((.((.((((((((((((((((((((.(((((((((.(((...((......))..))).))))))))).)))))).)))))))))))))).)).))).....    reads      mm      sample
    ........................uCccaagggaucgcauugaucgau...............................................................    1          1       NN8
    ......................................................................ucagugcaaucccucuggaau....................    19         0       NN8
    ......................................................................ucagugcaaucccucuggaauu...................    1          0       NN8
    ...........................................................................gcaaucccucuggaauucucc...............    1          0       NN8

    ........................uuccaagggaucgcauugau...................................................................    1          0       FF1
    ......................................................................ucagugcaaucccucuggaa.....................    1          0       FF1
    ......................................................................ucGgugcaaucccucuggaau....................    1          1       FF1
    ......................................................................ucagugcaGucccucuggaau....................    1          1       FF1
    ......................................................................Gcagugcaaucccucuggaau....................    2          1       FF1
    ......................................................................ucUgugcaaucccucuggaau....................    1          1       FF1
    ......................................................................ucagugcaaucccCcuggaau....................    1          1       FF1
    ......................................................................ucagugcUaucccucuggaau....................    1          1       FF1
    ......................................................................ucagugcaaucccucuAgaau....................    1          1       FF1
    ......................................................................ucagugcaaucccucuggaau....................    159        0       FF1
    ......................................................................Acagugcaaucccucuggaau....................    1          1       FF1
    ......................................................................ucagugcaaucccucuggaauA...................    1          1       FF1
    ......................................................................ucagugcaaucccucuggaauu...................    3          0       FF1
    .......................................................................cagugcaaucccucuggaauu...................    3          0       FF1
    .......................................................................cagugcaaucccucuCgaauu...................    1          1       FF1
    ...........................................................................gcaaucccucuggaauucuc................    1          0       FF1
    ...........................................................................gcaaucccucuggaauucucc...............    5          0       FF1
    ............................................................................caaucccucuggaauucucc...............    1          0       FF1
    ............................................................................caaucccucuggaauucuccC..............    1          1       FF1
    ............................................................................caaucccucuCgaauucuccg..............    1          1       FF1
    .............................................................................aaucccucuggaauucuccgc.............    1          0       FF1
    ..............................................................................aucccucuggaauucuccgcu............    3          0       FF1
```
