# Supplementary material for: Identification of Novel miRNAs and miRNA Expression Profiling in Wheat Hybrid Necrosis
Source: PLoS One. 2015 Feb 23;10(2):e0117507. doi: 10.1371/journal.pone.0117507 (PMC4338152; doi:10.1371/journal.pone.0117507)
Supplement: S3 Fig — (DOCX) [file pone.0117507.s003.docx]

**
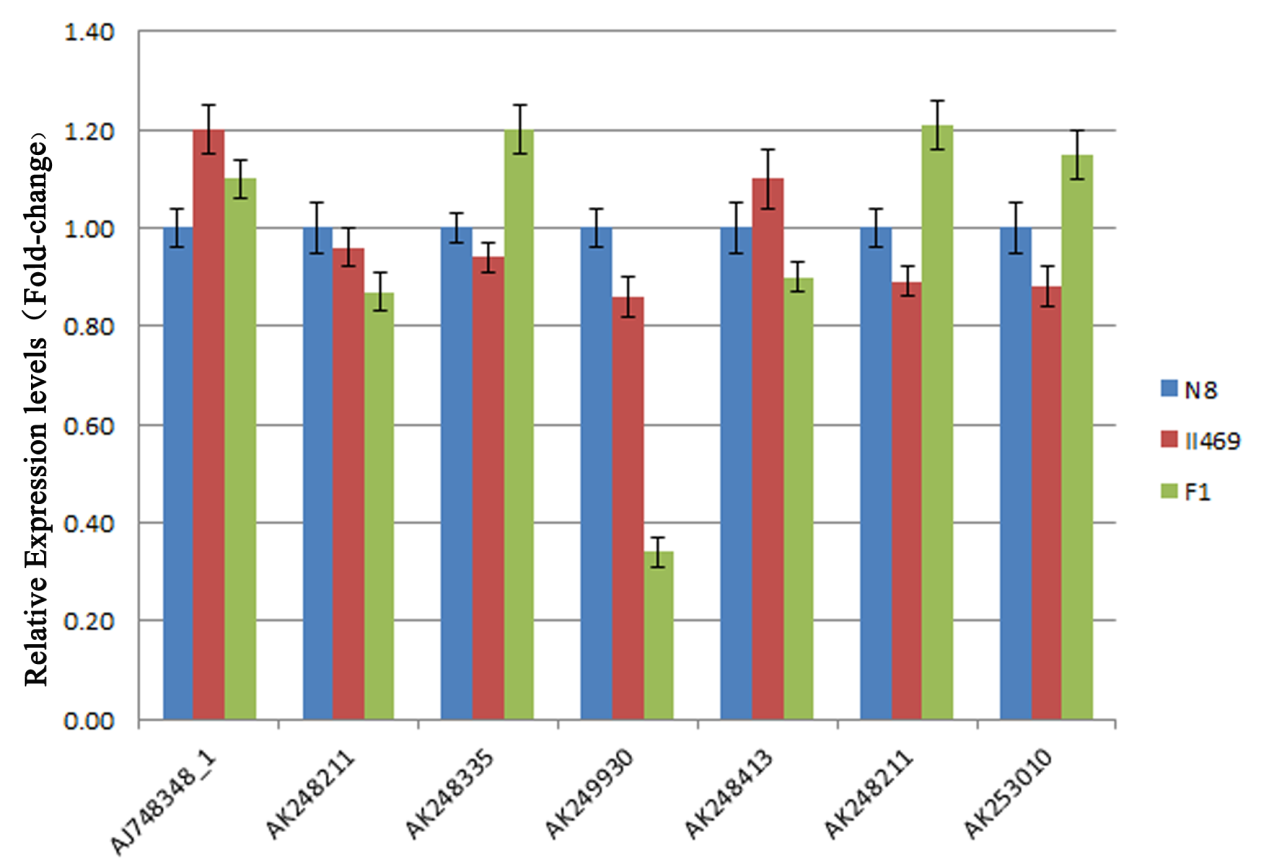
**

**Figure S3 qRT-PCR analysis of some predicated targets expression levels**

AJ748348_1 and AK248211 as predicated targets for miR159，AK248335 and AK249930 as predicated targets for miR165/166，AK248413 and RFL_Contig2908 as predicated targets for miR167, AK253010 as predicated targets for miR5072
