# Supplementary material for: Identification of Novel miRNAs and miRNA Expression Profiling in Wheat Hybrid Necrosis
Source: PLoS One. 2015 Feb 23;10(2):e0117507. doi: 10.1371/journal.pone.0117507 (PMC4338152; doi:10.1371/journal.pone.0117507)
Supplement: S2 Table — (DOCX) [file pone.0117507.s005.docx]

**Table S2 Known miRNAs identified from wheat and their expression patterns**

| GeneNames/Family | Total | F1 | P(N8+ II 469) | Signature(p-value < 0.001) | Species | No. of targets * | Targets prediction in TriFLDB: |
| --- | --- | --- | --- | --- | --- | --- | --- |
| miR166 | 98607 | 88822 | 9785 | yes | aly, aqc, ath, bdi, bna, cme, crt, csi, ctr, dpr, far, ghr, gma, hbr, hpa, hvu, mdm, mtr, nta, osa, pab, pde, ppt, pta, ptc, pvu, rco, sbi, sly, smo, ssl, tcc, vvi, zma | 91 | AK251614, AK248335, AK249930, AK250547, AK251305, AK251532, AK251614, AK252042, AK252491, AK252945, DQ333377_1, RFL_Contig2026, RFL_Contig23, RFL_Contig2575, RFL_Contig3121, RFL_Contig4276, RFL_Contig4908, RFL_Contig5320 |
| miR168 | 30573 | 15537 | 15036 | yes | aly, ath, bdi, bna, cca, ccl, cme, crt, gma, hvu, mdm, mes, mtr, nta, osa, ptc, rco, sbi, sof, ssp, tcc, vun, vvi, zma | 48 | AK249071, AK249441, AK249642, AK250165, AK250484, AK250693, DQ344624_1, RFL_Contig3744, RFL_Contig5816 |
| miR159 | 5712 | 5510 | 202 | yes | ahy, aly, aqc, ath, bdi, bna, bra, cme, csi, far, gma, hbr, htu, hvu, mdm, mes, mtr, nta, osa, pde, pta, ptc, pvu, rco, sbi, sly, smo, sof, ssp, tae, vvi, zma | 65 | AJ748348_1**(*CPI13*)** AK248211, AK248871, AK249781, AK249782, AK249920, AK250554, AK250584, AK250639, AK251585, AK251691, AK251726, RFL_Contig1268, RFL_Contig1341, RFL_Contig1720, RFL_Contig2060, RFL_Contig2416, RFL_Contig3991, RFL_Contig4798, RFL_Contig4966, RFL_Contig5146, RFL_Contig5245, RFL_Contig5245, RFL_Contig5421, RFL_Contig6122 |
| miR156 | 6658 | 5490 | 1168 | no | ahy, aly, aqc, ath, bcy, bdi, bgy, bna, cca, cme, csi, ctr, dpr, far, ghr, gma, han, har, hbr, hci, hpa, htu, hvu, mdm, mtr, nta, osa, ppt, ptc, rco, sbi, sly, smo, sof, ssl, ssp, tae, tcc, vun, vvi, zma | 43 | AK248333, AK248516, AK248564, AK248710, AK249692, AK249875, AK250175, AK250228, AK250327, AK251726, AK251899, AK252702, AY270159_1 ***(resistance protein T10 rga2-1A)***, RFL_Contig3928, RFL_Contig4144, RFL_Contig4702, RFL_Contig5059, RFL_Contig5715 |
| miR396 | 5895 | 4734 | 1161 | yes | aau, aly, amg, aqc, ath, bcy, bdi, bgy, bna, cca, ccl, cme, csi, dpr, far, ghr, gma, hbr, lji, mdm, mtr, nta, osa, pab, pde, pta, ptc, rco, sbi, smo, sof, ssl, ssp, tcc, vvi, zma | 29 | AF029895_1**(*acetyl-coenzyme A carboxylase*)**, AK248289, AK250253, AK250411, AK250518, AK250842, AK250947, AK252648, AK252975, AY987028_1**(*DRPL*)**, RFL_Contig1661, RFL_Contig2035, RFL_Contig2295, RFL_Contig4008, RFL_Contig4905, RFL_Contig5255, RFL_Contig5614, RFL_Contig5616, RFL_Contig5996, RFL_Contig6007 |
| miR167 | 2886 | 2587 | 299 | yes | ahy, aly, aqc, ath, bdi, bna, bra, cca, ccl, cme, csi, ctr, dpr, ghr, gma, gso, lja, mdm, mes, mtr, nta, osa, ppt, ptc, rco, sbi, sly, sof, ssp, tae, tcc, vvi, zma | 144 | AK248287, AK248413, AK248512, AK248901, AK249000, AK249072, AK249110, AK250049, AK250187, AK250755, AK251794, AK251910, AK252088, AK252446, AK252486, AK252565, RFL_Contig2908, RFL_Contig3600, RFL_Contig4572, RFL_Contig4774, RFL_Contig4952, RFL_Contig5729, RFL_Contig5743, RFL_Contig6101, RFL_Contig862 |
| miR398 | 30 | 2 | 28 | yes | ahy, aly, aqc, ath, bdi, bol, cca, cme, csi, gma, gra, mdm, mtr, nta, osa, ptc, rco, sbi, ssl, tae, tcc, vvi, zma | 18 | AK248890, AK249406, AK250170, AK250247, AK250513, AK251366, AK251625, AK252224, AK252290, AK252295, AK252349, DQ789026_1**(*J-domain protein,J3*)**, EF368365_1**(*WRKY46*)**, RFL_Contig2188, RFL_Contig3464, RFL_Contig4073, RFL_Contig412, RFL_Contig5189, RFL_Contig5389, RFL_Contig5482, RFL_Contig5825, RFL_Contig6081, RFL_Contig965, RFL_Contig983 |
| miR393 | 1882 | 1576 | 306 | no | aly, ath, bdi, bna, cca, cme, csi, ghr, gma, htu, mdm, mtr, osa, ptc, rco, sbi, tcc, vvi, zma | 52 | AK248583, AK249235, AK249581, AK249746, AK250083, AK250101, AK250797, AK252227, AK252377, AK252688, RFL_Contig1995, RFL_Contig5137, RFL_Contig6162 |
| miR169 | 2110 | 322 | 1788 | yes | aly, aqc, ath, bdi, bna, cca, cme, far, ghb, ghr, gma, hvu, mdm, mtr, nta, osa, pde, ptc, rco, sbi, sly, ssl, ssp, tcc, vun, vvi, zma | 72 | AB089270_1**(*fructan exohydrolase*)**, AF015523_1**(*LCT1*)**, AF315811_1**(*RNA-binding protein*)**, AJ400712_1**(*vp1A*)**, AK248190, AK248243, AK248286, AK248287, AK248433, AK248505, AK248636, AK248636, AK248887, AK248967, AK249062, AK249134, AK249190, AK249392, AK249550, AK249571, AK249596, AK249649, AK250201, AK250337, AK250590, AK250755, AK250842, AK251001, AK251182, AK251388, AK251637, AK251779, AK251936, AK252034, AK252289, AK252312, AK252375, AK252735, AK252890, AK252938, AY568306_1**(*WHAP12*)**, EF028777_1**(*CBFIVc-14.1*)**, RFL_Contig1803, RFL_Contig200, RFL_Contig2032, RFL_Contig2214, RFL_Contig2245, RFL_Contig2383, RFL_Contig2409, RFL_Contig2426, RFL_Contig245, RFL_Contig258, RFL_Contig3420, RFL_Contig3734, RFL_Contig3949, RFL_Contig4258, RFL_Contig4384, RFL_Contig5396, RFL_Contig5508, RFL_Contig5546, RFL_Contig5711, RFL_Contig5716, RFL_Contig69, RFL_Contig983, RFL_Contig998 |
| miR172 | 64 | 8 | 56 | yes | aau, aly, aqc, ata, ath, bdi, bna, bol, bra, cca, cme, csi, dpr, egu, gma, mdm, mes, mtr, nta, osa, ptc, rco, sbi, sly, ssl, tcc, vun, vvi, zma | 90 | AK248306, AK248773, AK249042, AK249973, AK250084, AK250491, AK250541, AK251982, AK253007, AK253046, AY069953_1**(*AP2L1*)**, AY714342_1**(*floral homeotic protein ,Q gene*)**, DQ195068_1**(*Dreb1*)**, RFL_Contig1024, RFL_Contig1956, RFL_Contig3759, RFL_Contig5446, RFL_Contig5974, RFL_Contig6029 |
| miR160 | 339 | 200 | 139 | yes | ahy,aly,aqc,ath,bdi,bna,bra,cca,cme,csi,dpr,far,gma,htu,mdm, mtr,nta,osa,pab,ppt,ptc,rco,sbi,sly,smo,tae,tcc,ttu,vun,vvi, zma | 39 | AB236423_1**(*beta-glucosidase*)**, AK248317, AK248370, AK248602, AK248710, AK250031, AK250101, AK250870, AK250908, AK250985, AK251606, AK251744, AK252100, EF368364_1**(*WRKY53-b*)**, RFL_Contig1422, RFL_Contig2804, RFL_Contig3147, RFL_Contig3275, RFL_Contig3995, RFL_Contig4332, RFL_Contig5271, RFL_Contig6061 |
| miR319 | 274 | 260 | 14 | yes | aau, aly, amg, aqc, ath, bdi, cca, cme, csi, ctr, gma, hbr, mdm, mtr, nta, osa, ppt, pta, ptc, pvu, rco, sbi, sly, tae, tcc, vun, vvi, zma | 38 | AK248195, AK248506, AK248669, AK248713, AK249722, AK249746, AK250381, AK250493, AK250785, AK250884, AK250899, AK251726, AK252107, AK252159, AK252174, AK252642, AK253009, RFL_Contig3398, RFL_Contig3703, RFL_Contig4814, RFL_Contig5247, RFL_Contig5687, RFL_Contig935, RFL_Contig989 |
| miR171 | 87 | 67 | 21 | no | aly, aqc, ath, bdi, bna, bol, bra, cca, ccl, cme, crt, csi, ctr, far, gma, hpa, htu, hvu, mdm, mtr, nta, osa, pde, ppt, ptc, rco, sbi, sly, smo, ssl, tae, tcc, vvi, zma | 6 | AB042240_15**(*chloroplast DNA*)**, AB159786_1**(*sucrose 1-fructosyltransferase*)**, AB334130_1**(*basic region/leucine zipper protein*)**, AJ784275_1**(*lrk1*)**, AK248167, AK248396, AK248512, AK248587, AK248687, AK248825, AK248994, AK249359, AK249544, AK250009, AK250428, AK250433, AK250475, AK250547, AK251250, AK251363, AK251457, AK251976, AK252267, AK252449, AK253119, EU327996_1**(*cereal cyst nematode resistance protein*)**, RFL_Contig1417, RFL_Contig1792,RFL_Contig2216,RFL_Contig2341,RFL_Contig2458,RFL_Contig2592,RFL_Contig3352,RFL_Contig3405,RFL_Contig3854,RFL_Contig4009,RFL_Contig441,RFL_Contig4645,RFL_Contig5299,RFL_Contig5564,RFL_Contig605,Y08625_1**(*subtilisin-chymotrypsin inhibitor 2*)** |
| miR394 | 60 | 60 | 0 | yes | ahy, aly, ath, bdi, bna, cca, cme, csi, ghr, gma, mdm, mes, nta, osa, ptc, sbi, ssl, tcc, vun, vvi, zma | 14 | AB042240_22**(*chloroplast DNA*)**, AK248356, AK249694, AK250473, AK252561, EF208805_1**(*folylpolyglutamate synthetase*)**, RFL_Contig149, RFL_Contig3134, RFL_Contig3835, RFL_Contig5693, RFL_Contig5797 |
| miR399 | 40 | 33 | 7 | no | aly, aqc, ath, bdi, bna, cca, cme, csi, ghr, gma, hvu, mdm, mes, mtr, osa, ptc, pvu, rco, sbi, sly, ssl, tae, tcc, vun, vvi, zma | 40 | AB302972_1**(*xylanase inhibitor*)***,* AK248279, AK248635, AK248860, AK249216, AK249253, AK249585, AK249882, AK250009, AK250106, AK250636, AK250858, AK250896, AK251479, AK251663, AK251703, AK251807, AK252613, AK252784, AK252925, DQ286568_1 *(LRR2)*, RFL_Contig1750, RFL_Contig2322, RFL_Contig2833, RFL_Contig4177, RFL_Contig4318, RFL_Contig4645, RFL_Contig4823, RFL_Contig5800, RFL_Contig5827, RFL_Contig6000, RFL_Contig6110, RFL_Contig674 |
| miR397 | 26 | 26 | 0 | yes | aly, ath, bdi, cme, csi, dpr, gma, hvu, mdm, osa, ptc, rco, sbi, sly, ssl, tcc, vvi | 94 | AK248279, AK248417, AK248714, AK249126, AK249775, AK250608, AK250633, AK250758, AK251676, AM075205_1, RFL_Contig1777, RFL_Contig181, RFL_Contig2008, RFL_Contig4611, RFL_Contig4961, U76384_1**(*o-methyltransferase*)**, X13158_1**(*chloroplast psaC*)** |
| miR164 | 43 | 29 | 14 | no | aly, ath, bdi, bna, bra, cca, cme, csi, ctr, far, ghr, gma, hci, mdm, mtr, nta, osa, ptc, rco, sbi, ssl, ssp, tae, tcc, vvi, zma | 523 | AK248229, AK248500, AK248692, AK249066, AK249325, AK250475, AK250533, AK250844, AK250961, AK251275, AK251877, AK252181, RFL_Contig1770, RFL_Contig2493, RFL_Contig2659, RFL_Contig4657, RFL_Contig5022, RFL_Contig5546 |
| miR2911 | 3808 | 1729 | 2079 | yes | han, nta, peu | 195 | Not found |
| miR827 | 105 | 105 | 0 | yes | aly, ath, bdi, csi, ghr, ssp, tcc, zma | 188 | AK250198, AK252441, AK252445, AK252534, RFL_Contig2299, RFL_Contig4151 |
| miR530 | 74 | 74 | 0 | yes | aqc, cme, csi, gma, htu, mtr, osa, ptc, tcc | 27 | AK248246, AK248356, AK249639, AK250177, AK250184, AK250762, AK250881, AK251649, AK252179, AM075205_1**(*6-FEH*)**, AY053452_1**(*NRT2.3*)**, DQ072270_1**(*ALMT1*)**, RFL_Contig1933, RFL_Contig2809, RFL_Contig2934, RFL_Contig3842, RFL_Contig3922, RFL_Contig5027 |
| miR170 | 71 | 71 | 0 | yes | aly, ath | 24 | RFL_Contig4645, RFL_Contig2216 |
| miR5072 | 1516 | 69 | 1447 | yes | osa | 45 | AK253010 |
| miR1432 | 89 | 68 | 21 | no | osa, sbi, ssp, zma | 27 | AK248973, RFL_Contig4384, RFL_Contig1213, AK251061 |
| miR5200 | 260 | 239 | 21 | yes | bdi | 8 | EF055987_1 **(*late flowering allele, FT*)** |
| miR4995 | 8487 | 218 | 8269 | yes | gma | 130 | AK251284 |
| miR5139 | 746 | 51 | 695 | yes | rgl | 12 | RFL_Contig6086 |
| miR1511 | 711 | 50 | 661 | yes | gma, mdm | 14 | AK251021, RFL_Contig5800, AK250661, AK252453 |
| miR5054 | 396 | 41 | 355 | yes | bdi | 17 | AK250941 |
| miR1120 | 252 | 29 | 223 | yes | hvu, tae | 100 | RFL_Contig1987, RFL_Contig6004 |
| miR6478 | 27 | 27 | 0 | yes | ptc | 31 | AK250912 |
| miR1125 | 82 | 26 | 56 | yes | tae | 80 | AK252099 |
| miR444 | 405 | 342 | 63 | no | bdi, hvu, osa, ssp, tae, zma | 189 | AK249841, AK250910, AM502900_1**(*WM30*)** |
| miR165 | 79571 | 70947 | 8624 | yes | aly, ath | 167 | RFL_Contig2575, AK250475, AK252949 |
| miR894 | 8265 | 823 | 7442 | yes | ppt | 20 | RFL_Contig4150 |
| miR5062 | 750 | 493 | 257 | yes | bdi | 44 | AK248833 |
| miR5048 | 9157 | 8545 | 612 | yes | hvu | 122 | AK248748 |
| miR1136 | 146 | 21 | 125 | yes | tae | 86 | AK251136 |
| miR818 | 200 | 19 | 181 | yes | osa | 131 | RFL_Contig1987, RFL_Contig1593 |
| miR3630 | 255 | 12 | 243 | yes | han | 72 | AK250333, RFL_Contig4688, RFL_Contig5540, RFL_Contig4019 |
| miR6300 | 25 | 11 | 14 | yes | gma | 3 | RFL_Contig1432 |
| miR1127 | 29 | 8 | 21 | yes | tae | 7 | RFL_Contig1104, RFL_Contig1391 |
| miR5077 | 2894 | 196 | 2698 | yes | osa | 0 | RFL_Contig3586 |
| miR5368 | 4779 | 140 | 4639 | yes | gma | 27 | AK253048 |
| miR1318 | 126 | 105 | 21 | no | osa | 11 | Not found |
| miR5083 | 314 | 8 | 306 | yes | osa | 158 | AK252914 |
| miR1128 | 35 | 7 | 28 | yes | ssp, tae | 48 | AK250735, RFL_Contig1104 |
| miR1436 | 28 | 7 | 21 | yes | hvu, osa | 55 | RFL_Contig6004 |
| miR5049 | 26 | 5 | 21 | yes | hvu | 24 | AK249841, AK251330, AK248405, AK250735, AK248614, AK248405, AK249841 |
| miR5073 | 26 | 5 | 21 | yes | osa | 67 | RFL_Contig2086 |
| miR5082 | 179 | 5 | 174 | yes | osa | 419 | RFL_Contig1892 |
| miR5071 | 328 | 300 | 28 | yes | osa | 55 | AK249338 |
| miR5064 | 324 | 289 | 35 | yes | bdi | 34 | RFL_Contig4707 |
| miR5203 | 29 | 1 | 28 | yes | bdi | 17 | RFL_Contig5201 |
| miR158 | 90 | 0 | 90 | yes | aly, ath | 4 | RFL_Contig4362, RFL_Contig4295 |
| miR5538 | 21 | 0 | 21 | yes | osa | 35 | AK252358 |
| miR6191 | 21 | 0 | 21 | yes | hvu | 20 | AK249934 |
| miR6203 | 21 | 0 | 21 | yes | hvu | 3 | DQ013357_1 **(*WAK2*)** |

Aau, Acacia auriculiformis;Ahy，Arachis hypogaea;aly, Arabidopsis lyrata;amg, Acacia mangium;aqc, Aquilegia caerulea;ata, Aegilops tauschii,ath, Arabidopsis thaliana;bcy, Bruguiera cylindrical;bdi, Brachypodium distachyon;bgy, Bruguiera gymnorhiza;bna, Brassica napus;bol, Brassica oleracea;bra, Brassica rapa;cca, Cynara cardunculus;ccl, Citrus clementine;cme, Cucumis melo;crt, Citrus reticulate;csi, Citrus sinensis;ctr, Citrus trifoliate;dpr, Digitalis purpurea;egu, Elaeis guineensis;far, Festuca arundinacea;ghb, Gossypium herbaceum;ghr, Gossypium hirsutum;gma, Glycine max;gra, Gossypium raimondii;gso, Glycine soja;han, Helianthus annuus;har, Helianthus argophyllus;hbr, Hevea brasiliensis;hci, Helianthus ciliaris;hpa, Helianthus paradoxus;htu, Helianthus tuberosus;hvu, Hordeum vulgare;lja, Lotus japonicas;mdm, Malus domestica;mes, Manihot esculenta;mtr, Medicago truncatula;nta, Nicotiana tabacum;osa, Oryza sativa;pab, Picea abies;pde, Pinus densata;ppt, Physcomitrella patens;pta, Pinus taeda;ptc, Populus trichocarpa;pvu, Phaseolus vulgaris;rco, Ricinus communis;rgl, Rehmannia glutinosa;sbi, Sorghum bicolor;sly, Solanum lycopersicum;smo, Selaginella moellendorffii;sof, Saccharum officinarum;ssl, Salvia sclarea;ssp, Saccharum ssp.;tae, Triticum aestivum;tcc, Theobroma cacao;vun, Vigna unguiculata;vvi, Vitis vinifera;zma, Zea mays.

The miRBase database and the EST sequence of wheat were used as reference. * Targets prediction in EST database and the detailed information is listed listed in Table S2.

The highlighted in red letter indicated that the consensus miRNAs have been reported in wheat. And the bold and italic indicated the annotation gene.
