# Supplementary material for: Identification of Novel miRNAs and miRNA Expression Profiling in Wheat Hybrid Necrosis
Source: PLoS One. 2015 Feb 23;10(2):e0117507. doi: 10.1371/journal.pone.0117507 (PMC4338152; doi:10.1371/journal.pone.0117507)
Supplement: S3 Table — (DOCX) [file pone.0117507.s006.docx]

**Table S3 Prediction of novel miRNAs and their precursors, expression and targets prediction**

| Genename | #provisional_id | Consensus mature sequence | Consensus star sequence | Consensus precursor sequence | All_samples(total[mature,loop,star]) | F1 total [mature,loop,star] | N8_II469 total [mature,loop,star] | Significant randfold p-value | No. of targets * | Targets prediction in TriFLDB |
| --- | --- | --- | --- | --- | --- | --- | --- | --- | --- | --- |
| Tae-2131a | contig38447_1368 | ucgcuuggugcagaucgggac | cccgccuugcaccaagugaau | ucgcuuggugcagaucgggacccuccgcccgcccgacgggccggaucccgccuugcaccaagugaau | 18190[15464,0,2726] | 15543[13302,0,2241] | 2647[2162,0,485] | yes | 89 | RFL_Contig5077, RFL_Contig6139, RFL_Contig3744 |
| Tae-2131b | contig853436_9080 | ucgcuuggugcagaucgggac | cccgccuugcaccaagugaau | ucgcuuggugcagaucgggacccuccgcccgccccgacgggccggaucccgccuugcaccaagugaau | 18190[15464,0,2726] | 15543[13302,0,2241] | 2647[2162,0,485] | yes | 89 | RFL_Contig5077, RFL_Contig6139, RFL_Contig3744 |
| Tae-2131c | contig316572_5328 | ucgcuuggugcagaucgggac | cccgccuugcaccaagugaau | ucgcuuggugcagaucgggacccucagcccgccccgacgggccggaucccgccuugcaccaagugaau | 18190[15464,0,2726] | 15543[13302,0,2241] | 2647[2162,0,485] | yes | 89 | RFL_Contig5077, RFL_Contig6139, RFL_Contig3744 |
| Tae-2132a | contig106724_2720 | ugacagaagagagugagcac | gcucacuccucuuucugucagc | ugacagaagagagugagcacauggcgugaugccggcauaacauguaugccgucuucgccgcgugcucacuccucuuucugucagc | 7610[4810,0,2800] | 5841[4642,0,1199] | 1769[168,0,1601] | yes | 23 | RFL_Contig3928,AK249633, RFL_Contig4589 |
| Tae-2132b | contig2137453_13522 | ugacagaagagagugagcac | gcucacuccucuuucugucagc | ugacagaagagagugagcacacggcgugaugccggcauaacauguaugccgucuucgccgcgugcucacuccucuuucugucagc | 7610[4810,0,2800] | 5841[4642,0,1199] | 1769[168,0,1601] | yes | 23 | RFL_Contig3928  AK249633  RFL_Contig4589 |
| Tae-2132c | contig936343_9448 | ugacagaagagagugagcac | gcucacugcucuaucugucagc | ugacagaagagagugagcaccaugguguuuccuuagcaucaaggggcaugccagggagcuaugcgugcucacugcucuaucugucagc | 4848[4807,0,41] | 4663[4639,0,24] | 185[168,0,17] | yes | 23 | RFL_Contig3928,AK249633, RFL_Contig4589 |
| Tae-2132d | contig936343_9449 | ugacagaagagagugagcac | gcucacccucucucugucagc | ugacagaagagagugagcacacgcggugguuccuagcaugcgagcgccaugcugggagcugcgcgugcucacccucucucugucagc | 4115[3427,0,688] | 3527[3273,0,254] | 588[154,0,434] | yes | 23 | RFL_Contig3928,AK249633, RFL_Contig4589 |
| Tae-2132e | contig1299632_10941 | ugacagaagagagugagcac | gcucacugcucuuccugucauc | ugacagaagagagugagcacacggccggucguuacggcaccgcccgggugugccgucgcggccgcgugcucacugcucuuccugucauc | 3972[3406,0,566] | 3503[3252,0,251] | 469[154,0,315] | yes | 23 | RFL_Contig3928,AK249633, RFL_Contig4589 |
| Tae-2132f | contig1109357_10188 | ugacagaagagagugagcac | gcucacugcucuuccugucauc | ugacagaagagagugagcacacggccggucguuacggcaccgcccgggugugccgucgcggccgcgugcucacugcucuuccugucauc | 3972[3406,0,566] | 3503[3252,0,251] | 469[154,0,315] | yes | 23 | RFL_Contig3928,AK249633, RFL_Contig4589 |
| Tae-2132g | contig1461569_11549 | ugacagaagagagugagcac | gcucacugcucuuccugucauc | ugacagaagagagugagcacacggccggucguuacggcaccgccgggugugccgucgcggccgcgugcucacugcucuuccugucauc | 3972[3406,0,566] | 3503[3252,0,251] | 469[154,0,315] | yes | 23 | RFL_Contig3928AK249633, RFL_Contig4589 |
| Tae-2133a | contig106305_2702 | ugaagcugccagcaugaucuga | aggucaugcuggaguuucauc | ugaagcugccagcaugaucugaugaccuaagucauggaucagaauccaugucaaucaggucaugcuggaguuucauc | 2338[2265,0,73] | 2295[2223,0,72] | 43[42,0,1] | yes | 305 | AK251007, AK248901 |
| Tae-2133b | contig754184_8517 | ugaagcugccagcaugaucuga | aggucaugcuggaguucauc | ugaagcugccagcaugaucugaugaccuaacucauggaucagaaguccaugucaaucaggucaugcuggaguucauc | 2265[2265,0,0] | 2223[2223,0,0] | 42[42,0,0] | yes | 216 | AK251007, AK248901 |
| Tae-2134a | contig862671_9103 | uugaacaucccagagccaccg | gcggcucugugguguucaagc | gcggcucugugguguucaagcaggaaccucaugcuaccggcagcauguugcgcuugcuugaacaucccagagccaccg | 1812[1509,0,303] | 1642[1339,0,303] | 170[170,0,0] | yes | 20 | RFL_Contig3813 |
| Tae-2134b | contig219800_4299 | uugaacaucccagagccaccg | gcggcucugugguguucaagc | gcggcucugugguguucaagcaggaaccucaugcuaccggcaggaugcggcgcuugcuugaacaucccagagccaccg | 1812[1509,0,303] | 1642[1339,0,303] | 170[170,0,0] | yes | 20 | RFL_Contig3813 |
| Tae-2134c | contig863626_9114 | uugaacaucccagagccaccg | gcggcucucugguguucaagc | gcggcucucugguguucaagcaggaaccuccaugcuaccggcagcaugcggcgcuugcuugaacaucccagagccaccg | 1532[1509,0,23] | 1362[1339,0,23] | 170[170,0,0] | yes | 20 | RFL_Contig3813 |
| Tae-2135 | contig288356_5066 | uacugugggcacuuauuugaca | gcaaauagugcccuuaguaug | uacugugggcacuuauuugacaagaaugaggagaaacaugaauucuuggcaaauagugcccuuaguaug | 1334[1332,0,2] | 1314[1312,0,2] | 20[20,0,0] | yes | 24 | AK248973 |
| Tae-2136a | contig862671_9106 | uucuucaaguacuccacuuuu | agguggaauacuugaagaaga | uucuucaaguacuccacuuuuuuuucucgcggagaaaaagguggaauacuugaagaaga | 565[564,0,1] | 557[556,0,1] | 8[8,0,0] | yes | 150 | AK250391 |
| Tae-2136b | contig5043762_19076 | uucuucaaguacuccacuuuu | agguggaauacuugaagaaga | uucuucaaguacuccacuuuuuuuucuugcggagaaaaagguggaauacuugaagaaga | 565[564,0,1] | 557[556,0,1] | 8[8,0,0] | yes | 150 | AK250391 |
| Tae-2137 | contig839524_8997 | uccaaagggaucgcauugauc | aucaugcgauccuuuuggaag | uccaaagggaucgcauugauccuuccauccaugguguugauggcugucaucaaucaaccggcucgacagaucaugcgauccuuuuggaag | 418[416,0,2] | 384[382,0,2] | 34[34,0,0] | yes | 35 | RFL_Contig4097 |
| Tae-2138 | contig1597271_12023 | uuccacagcuuucuugaacug | uucaauaaagcugugggaaa | uuccacagcuuucuugaacugcaucugcaauugguggaugcuagcuucuaccggcaagaucugcaguucaauaaagcugugggaaa | 362[361,0,1] | 314[313,0,1] | 48[48,0,0] | yes | 83 | RFL_Contig5614 |
| Tae-2139a | contig4164108_17456 | uggacgaggaugugcagcugc | agcugcacauccacuuccaag | uggacgaggaugugcagcugcgguaggacguucagcuacgaccggcagcugcacauccacuuccaag | 349[322,0,27] | 340[321,0,19] | 9[1,0,8] | yes | 354 | RFL_Contig5429 |
| Tae-2139b | contig254772_4721 | uggacgaggaugugcagcugc | agcugcacauccacguccaag | uggacgaggaugugcagcugcgguaggacucucagcuacgaccggcagcugcacauccacguccaag | 329[322,0,7] | 327[321,0,6] | 2[1,0,1] | yes | 354 | RFL_Contig5429 |
| Tae-2140 | contig2537991_14443 | cagccaaggaugacuugccggc | ggcaaguugucuuuggcuac | cagccaaggaugacuugccggcuccuggugcugggaguucguugagcuuuagaguuagccggcaaguugucuuuggcuac | 335[223,0,112] | 272[198,0,74] | 63[25,0,38] | yes | 81 | AK251359 |
| Tae-2141 | contig4778939_18647 | ggccuauuagcucagcuggu | gguucgagaccuguaugggcc | ggccuauuagcucagcugguuagagcuucgugcuaauaacgcgaaggucacagguucgagaccuguaugggcc | 288[238,3,47] | 248[234,2,12] | 40[4,1,35] | yes | 14 | AK249434 |
| Tae-2142a | contig923344_9386 | ucggaccaggcuucauuccuu | ggaacguuggcuggcucgagg | ggaacguuggcuggcucgaggcauccuugccgacggcgcggguagaggcgucggaccaggcuucauuccuu | 243[242,0,1] | 239[238,0,1] | 4[4,0,0] | yes | 81 | AK252042 |
| Tae-2142b | contig422991_6267 | ucggaccaggcuucauuccuu | ggaacguuggcuggcucgagg | ggaacguuggcuggcucgaggcauccuugccgacggcgcgggucgaggcgucggaccaggcuucauuccuu | 243[242,0,1] | 239[238,0,1] | 4[4,0,0] | yes | 81 | AK252042 |
| Tae-2142c | contig70790_2116 | ucggaccaggcuucauuccuu | ggaacguuggcuggcucgagg | ggaacguuggcuggcucgaggcauccuugccgacggcgcggguagaggcgucggaccaggcuucauuccuu | 243[242,0,1] | 239[238,0,1] | 4[4,0,0] | yes | 81 | AK252042 |
| Tae-2143 | contig94531_2506 | ucagugcaaucccucuggaau | uuccaagggaucgcauugaucgau | uuccaagggaucgcauugaucgaucucuccgugcaaggucgauggaucagugcaaucccucuggaau | 207[205,0,2] | 185[184,0,1] | 22[21,0,1] | yes | 16 | AY485643_5 |
| Tae-2144a | contig399813_6090 | ugccuggcucccuguaugcca | gcgugcaaggagccaagcaug | ugccuggcucccuguaugccacucauguagccaacccgcggcgugauuggaugcuguggguggcgugcaaggagccaagcaug | 149[146,0,3] | 139[136,0,3] | 10[10,0,0] | yes | 37 | RFL_Contig6089, RFL_Contig4228, AK251681 |
| Tae-2144b | contig515251_7046 | ugccuggcucccuguaugcca | gcgugcaaggggccaagcaug | ugccuggcucccuguaugccacacauguagcccaacccauggaugugguugccugcugguggcgugcaaggggccaagcaug | 145[144,0,1] | 135[134,0,1] | 10[10,0,0] | yes | 37 | RFL_Contig6089, RFL_Contig4228, AK251681 |
| Tae-2144c | contig1169132_10464 | ugccuggcucccuguaugcca | gcgugcaagggccaagcaug | ugccuggcucccuguaugccacacauguagcccaacccauggaugugguugccugcugugguggcgugcaagggccaagcaug | 144[144,0,0] | 134[134,0,0] | 10[10,0,0] | yes | 37 | RFL_Contig6089, RFL_Contig4228, AK251681 |
| Tae-2144d | contig767379_8575 | ugccuggcucccuguaugcca | gcgugcacggauccaagcaua | ugccuggcucccuguaugccacucauccagagcaacaccuuuugcaauaagguugccugcgauggauggcgugcacggauccaagcaua | 146[146,0,0] | 136[136,0,0] | 10[10,0,0] | yes | 37 | RFL_Contig6089, RFL_Contig4228, AK251681 |
| Tae-2145 | contig1984885_13090 | ggcagucaccuuggcuagc | uagucaaggaugacuugccua | uagucaaggaugacuugccuaguuuucauccauguucaaugcuucuuagccuuggcguggguucuuauggcagucaccuuggcuagc | 141[120,0,21] | 124[119,0,5] | 17[1,0,16] | yes | 10 | RFL_Contig5439 |
| Tae-2146 | contig1941326_12979 | guaggauggccggugcaaugg | auagcaucauccauccuacc | auagcaucauccauccuaccuucaauggcggcugcugcugcccacggugguaggauggccggugcaaugg | 135[99,0,36] | 129[98,0,31] | 6[1,0,5] | yes | 20 | AK249447 |
| Tae-2147a | contig443571_6462 | uuuaagucucugaugauc | aucaaaggaugagcaaauacugc | aucaaaggaugagcaaauacugcaacaauaugauccaugaucugauucuuaguauuuaagucucugaugauc | 134[123,2,9] | 29[20,1,8] | 105[103,1,1] | yes | 6 | AK251953 |
| Tae-2147b | contig31094_1188 | uuuaagucucugaugauc | aucaaaggaugagcaaauacugc | aucaaaggaugagcaaauacugcaacaauaugauccaugaucugauucuuaguauuuaagucucugaugauc | 134[123,2,9] | 29[20,1,8] | 105[103,1,1] | no | 6 | AK251953 |
| Tae-2148 | contig253044_4695 | cgcccacaacggagaaaccggc | cgguuccucucuuguuugcgaa | cgcccacaacggagaaaccggccagccggccgguuccucucuuguuugcgaa | 128[115,0,13] | 108[100,0,8] | 20[15,0,5] | yes | 3 | RFL_Contig1747 |
| Tae-2149a | contig157376_3524 | accugcaguugggccaaugac | cauugacccaacugcaggugc | accugcaguugggccaaugacaugugggccuaacagacggcuggcccacaugccauugacccaacugcaggugc | 119[118,0,1] | 60[60,0,0] | 59[58,0,1] | yes | 91 | AK248787 |
| Tae-2149b | contig700267_8204 | accugcaguugggccaaugac | cauugacccaacugcaggugc | accugcaguugggccaaugacaugugggccuaacagacggcuggcccacaugccauugacccaacugcaggugc | 119[118,0,1] | 60[60,0,0] | 59[58,0,1] | yes | 91 | AK248787 |
| Tae-2149c | contig2460659_14297 | accugcaguugggccaaugac | cauugauccaacuguaggugc | accugcaguugggccaaugacaugugggccuaacagacggcuggcccacaugccauugauccaacuguaggugc | 116[116,0,0] | 58[58,0,0] | 58[58,0,0] | yes | 91 | AK248787 |
| Tae-2150 | contig4244526_17663 | acggcauagaggcacugcaaa | ugcaguccucgaugucguag | acggcauagaggcacugcaaacauccgagaugagcaaacuaagcuuguccguaguuugcaguccucgaugucguag | 117[34,0,83] | 97[33,0,64] | 20[1,0,19] | yes | 11 | AK250554 |
| Tae-2151a | contig600599_7592 | aucaggagagaugacaccgac | ggugucaccucgccugaaca | aucaggagagaugacaccgacgccgaucggaugggucggcuuaaccaggccaugcauccgucuggccggaguuggugucaccucgccugaa | 98[97,0,1] | 94[93,0,1] | 4[4,0,0] | yes | 25 | AK252860 |
| Tae-2151b | contig17780_764 | aucaggagagaugacaccgac | ggugucaccucgccugaaca | aucaggagagaugacaccgacgccgaucagaugggucggcuuaaccaggccaugcauccgucuguucggaguuggugucaccucgccugaa | 98[97,0,1] | 94[93,0,1] | 4[4,0,0] | yes | 25 | AK252860 |
| Tae-2152 | contig1423883_11407 | uuugcaugaccgaggagccgc | agcugcucguucaugguuccc | agcugcucguucaugguucccacuauccuaucuccauagaaaacgaggagguaggccugugguuugcaugaccgaggagccgc | 78[59,2,17] | 74[58,2,14] | 4[1,0,3] | yes | 27 | AK249029 |
| Tae-2153 | contig07156_419 | gucucugccaauucuucgugu | gcgaaggauuugcagauacuc | gcgaaggauuugcagauacuccguaaaaucacuuuauggagucucugccaauucuucgugu | 78[52,0,26] | 69[51,0,18] | 9[1,0,8] | yes | 29 | AY846821_1 (***ribosomal protein L10A***) |
| Tae-2154a | contig4020558_17163 | ugauugagccgcgccaauauc | uauuggcucggcucacucagg | uauuggcucggcucacucaggucaucguguugcagaagcauaugcuucucgguauaaucugauugagccgcgccaauauc | 67[62,0,5] | 64[59,0,5] | 3[3,0,0] | yes | 8 | RFL_Contig4645 |
| Tae-2154b | contig4055796_17218 | ugauugagccgcgccaauauc | uauuggcucggcucacucagg | uauuggcucggcucacucaggucaucguguugcagaagcauaugcuucucgguauaaucugauugagccgcgccaauauc | 67[62,0,5] | 64[59,0,5] | 3[3,0,0] | yes | 8 | RFL_Contig4645 |
| Tae-2155a | contig1844859_12689 | ccaugaugaggucguucaacc | uugaacgaccucaccaugucga | uugaacgaccucaccaugucgaccgccucuuccucgacaagugccagggagaucugcucgugccaugaugaggucguucaacc | 71[69,0,2] | 58[56,0,2] | 13[13,0,0] | yes | 127 | AK250387 |
| Tae-2155b | contig748329_8480 | ccaugaugaggucguucaacc | uugaacgagcucaccaugugga | uugaacgagcucaccauguggaccgccucuuccucgacaagugccagggagaucugcucgugccaugaugaggucguucaacc | 69[69,0,0] | 56[56,0,0] | 13[13,0,0] | yes | 127 | AK250387 |
| Tae-2156a | contig958892_9544 | ugaagcugccagcaugaucua | aucaugacugacagccucauu | ugaagcugccagcaugaucuaucuuugauucgcuucugcgugugaauccuuguuagaucaugacugacagccucauu | 60[47,0,13] | 58[46,0,12] | 2[1,0,1] | yes | 134 | AK248901 |
| Tae-2156b | contig1803535_12570 | ugaagcugccagcaugaucuac | aucaugacugacagccucauu | ugaagcugccagcaugaucuaccuuugauucgcuucugcgugugaauccuuguuagaucaugacugacagccucauu | 1025[1012,0,13] | 997[985,0,12] | 28[27,0,1] | yes | 216 | AK248901 |
| Tae-2157 | contig1941326_12977 | auagcaucauccauccugcca | uagaauggcuggugcuaugga | auagcaucauccauccugccauucugcugagguagcugcugcccuuuugcacgauggguagaauggcuggugcuaugga | 60[57,0,3] | 50[47,0,3] | 10[10,0,0] | yes | 23 | AK248342 |
| Tae-2158a | contig5111788_19161 | gccaucauacguccaaccgug | cgguugggcuguaugauggcga | gccaucauacguccaaccgugcauuugauaugcauauauaugcaucacgagccacgguugggcuguaugauggcga | 56[45,0,11] | 39[28,0,11] | 17[17,0,0] | yes | 14 | AK250015 |
| Tae-2158b | contig3048700_15424 | cgguugggcuguaugauggcga | ccaucauacguccaaccgug | ccaucauacguccaaccgugcauuugauaugcauauauaugcaucacgagccacgguugggcuguaugauggcga | 12[11,0,1] | 12[11,0,1] | 0[0,0,0] | yes | 64 | AK250015 |
| Tae-2158c | contig4603203_18346 | cgguugggcuguaugauggcga | gccaucauacauccaaccgug | gccaucauacauccaaccgugcauuugauaugcauauauaugcaucacgagcaacgguugggcuguaugauggcga | 12[11,0,1] | 12[11,0,1] | 0[0,0,0] | yes | 64 | AK250015 |
| Tae-2159a | contig98625_2582 | uggagaagcagggcacgugcu | cacgugcgcuccuucuccagc | uggagaagcagggcacgugcucgauccauggacccaaagcgguugcagcacgugcgcuccuucuccagc | 46[31,0,15] | 42[29,0,13] | 4[2,0,2] | yes | 509 | AK250475 |
| Tae-2159b | contig169127_3668 | uggagaagcagggcacgugcu | cacgugcgcuccuucuccagc | uggagaagcagggcacgugcucgauccaucgacccaaagcgguugcagcacgugcgcuccuucuccagc | 46[31,0,15] | 42[29,0,13] | 4[2,0,2] | yes | 509 | AK250475 |
| Tae-2159c | contig1173624_10491 | uggagaagcagggcacgugcu | cacgugcgcuccuucuccagc | uggagaagcagggcacgugcucgauccauauggacccaaagcgguugcagcacgugcgcuccuucuccagc | 46[31,0,15] | 42[29,0,13] | 4[2,0,2] | yes | 509 | AK250475 |
| Tae-2160 | contig3273262_15798 | uuaugacuucucuuagacagu | gugugagagaagucauaagug | gugugagagaagucauaagugugaaacacucuacgaugaugaugacgaagcugcaaacacuuaugacuucucuuagacagu | 47[38,0,9] | 43[34,0,9] | 4[4,0,0] | yes | 5 | AK250213 |
| Tae-2161 | contig379598_5894 | ugggcaagucauccuggcuacc | uagccaaggaugauuugc | uagccaaggaugauuugccugugaaccucccccgggagcccagcucucgggaaccugguuccaugggcaagucauccuggcuacc | 46[33,0,13] | 44[32,0,12] | 2[1,0,1] | yes | 104 | AY568306_1 (***WHAP12***) |
| Tae-2162a | contig59907_1840 | uugagugcagcguugaugaac | ucaccggcgcugcacacaaug | uugagugcagcguugaugaaccguccggccuccuccgaggccggagcgguucaccggcgcugcacacaaug | 44[23,0,21] | 28[22,0,6] | 16[1,0,15] | yes | 38 | RFL_Contig3899, AK249126 |
| Tae-2162b | contig1047595_9935 | uugagugcagcguugaugaac | ucaccggcgcugcacacaaug | uugagugcagcguugaugaaccguccuacuccuccgaggccggagcgguucaccggcgcugcacacaaug | 44[23,0,21] | 28[22,0,6] | 16[1,0,15] | yes | 38 | RFL_Contig3899, AK249126 |
| Tae-2163 | contig2984958_15313 | ccauuaguaccgguuggagcc | caccaaccgguacuaaaggcc | caccaaccgguacuaaaggcccucccauuaguaccgguuggagcc | 39[30,0,9] | 11[10,0,1] | 28[20,0,8] | yes | 6 | AK250580 |
| Tae-2164 | contig1364498_11207 | uucugucccgguucguguaagaac | uucgaaaaccgggacaaaaggccc | uucugucccgguucguguaagaaccggaacuaaaggguuaggcuuuaguaacgacccuuuagucccaguucgaaaaccgggacaaaaggcc | 32[25,3,4] | 26[20,3,3] | 6[5,0,1] | yes | 43 | AK248287 |
| Tae-2165a | contig4877233_18823 | gccaucauacgcccaaccgug | cgguagggcuguaugauggcga | gccaucauacgcccaaccgugcauuugauaugcauauauaugcaucacgagccacgguagggcuguaugauggcga | 27[22,0,5] | 13[8,0,5] | 14[14,0,0] | yes | 13 | RFL_Contig3008 |
| Tae-2165b | contig4238746_17655 | gccaucauacgcccaaccgug | cgguagggcuguaugauggcga | gccaucauacgcccaaccgugcauuugaaaugcauauauaugcaucacgagccacgguagggcuguaugauggcga | 27[22,0,5] | 13[8,0,5] | 14[14,0,0] | yes | 13 | RFL_Contig3008 |
| Tae-2165c | contig2287307_13867 | cgguagggcuguaugauggcga | gccaucauacgcccaaccau | gccaucauacgcccaaccauacauucgauaugcauauauaugcaucacgagccacgguagggcuguaugauggcga | 9[5,0,4] | 6[5,0,1] | 3[0,0,3] | yes | 62 | RFL_Contig3008 |
| Tae-2166 | contig1769557_12487 | uccgugaacuaauauaagagc | uuuuauauuaguuuacagagg | uccgugaacuaauauaagagcguucuaacgcuuuuauauuaguuuacagagg | 26[19,0,7] | 17[16,0,1] | 9[3,0,6] | yes | 29 | RFL_Contig2491 |
| Tae-2167a | contig2312229_13918 | agaaucuugaugaugcugcau | gcagcaccaccaagauucaca | gcagcaccaccaagauucacaucggauccgucguaaaaucaaauuuaugcgacgcgccaggugaugagaaucuugaugaugcugcau | 18[15,0,3] | 7[7,0,0] | 11[8,0,3] | yes | 52 | AY714342_1 (***floral homeotic protein (Q) gene***) |
| Tae-2167b | contig2566467_14516 | agaaucuugaugaugcugcau | gcagcaccaccaagauucaca | gcagcaccaccaagauucacaucgcauccgucguaaauuaaauuuaugcgacgcgccagaugaugagaaucuugaugaugcugcau | 18[15,0,3] | 7[7,0,0] | 11[8,0,3] | yes | 52 | AY714342_1 ***(floral homeotic protein (Q) gene)*** |
| Tae-2167c | contig1109643_10191 | agaaucuugaugaugcugcau | gcagcaccaccaagauucaca | gcagcaccaccaagauucacaucggauccgucgucguaaauuaaauuuaugcgacgcgccaggugaugagaaucuugaugaugcugcau | 18[15,0,3] | 7[7,0,0] | 11[8,0,3] | yes | 52 | AY714342_1 ***(floral homeotic protein (Q) gene)*** |
| Tae-2168 | contig102619_2645 | uugcgagcaacggaugaauc | uuucuccuuugcucgaguagaga | uugcgagcaacggaugaaucagccuuguuauccaauccagagguggcugauuucuccuuugcucgaguagaga | 16[15,0,1] | 16[15,0,1] | 0[0,0,0] | yes | 7 | AK250481 |
| Tae-2169 | contig2088896_13400 | acgagauaccuuaagcuucugacc | ucggaaguuugagguaucu | ucggaaguuugagguaucucgugaauacaaggccgacuugguauauuacguuggcauuguuauacgagauaccuuaagcuucugacc | 4[3,0,1] | 4[3,0,1] | 0[0,0,0] | yes | 10 | RFL_Contig5759 |
| Tae-2170a | contig112667_2846 | ugacagaagagagcgagcac | ucucccuccucuuccgucggc | ugacagaagagagcgagcacacggcggaggccggcgucguggagacgccgacccugacgcguucucccuccucuuccgucggc | 16[16,0,0] | 16[16,0,0] | 0[0,0,0] | yes | 24 | RFL_Contig1264,  RFL_Contig3928,AK250248, RFL_Contig539 |
| Tae-2170b | contig1885144_12836 | ugacagaagagagcgagcac | gaucccuccucuuuccgucggc | ugacagaagagagcgagcacacgguggaggcccgcgucauggagacgccacccccgacgcgugaucccuccucuuuccgucggc | 16[16,0,0] | 16[16,0,0] | 0[0,0,0] | no | 24 | RFL_Contig1264,RFL_Contig3928,AK250248, RFL_Contig539 |
| Tae-2170c | contig112667_2845 | ugacagaagagagcgagcac | gcugguuccauccgccgug | gcugguuccauccgccgugcccgcgggaggcugacagaagagagcgagcac | 16[16,0,0] | 16[16,0,0] | 0[0,0,0] | no | 24 | RFL_Contig1264,RFL_Contig3928,AK250248, RFL_Contig539 |
| Tae-2170d | contig1885144_12835 | ugacagaagagagcgagcac | guuuguucuacuccggu | guuuguucuacuccgguacguggugugcugguuccaucugccgugcccgcgggaggcugacagaagagagcgagcac | 16[16,0,0] | 16[16,0,0] | 0[0,0,0] | no | 24 | RFL_Contig1264,RFL_Contig3928,AK250248, RFL_Contig539 |
| Tae-2171 | contig474865_6755 | uuccaaagggaucgcauugau | cagugcaauccucuggaauu | uuccaaagggaucgcauugaucgaucucuccgucaucggcgugcaaggucgauggaucagugcaauccucuggaauu | 1324[1323,1,0] | 1279[1278,1,0] | 45[45,0,0] | yes | 61 | AY485643_5 |
| Tae-2172 | contig533924_7175 | uuccgauuuacucgucguggu | cacgacgaguaaauuggaacg | uuccgauuuacucgucgugguaaaaccacgacgaguaaauuggaacg | 21[21,0,0] | 20[20,0,0] | 1[1,0,0] | yes | 10 | RFL_Contig761 |
| Tae-2173a | contig370756_5808 | cuuuggucccgauucguguugaac | uccuuaccaaucgggacuaaaggu | uccuuaccaaucgggacuaaaggucccccagaccacggcgcgccucgugccacguggugggccuuuggucccgauucguguugaac | 22[19,3,0] | 12[9,3,0] | 10[10,0,0] | yes | 91 | RFL_Contig4702, AK249139 |
| Tae-2173b | contig370756_5808 | cuuuggucccgauucguguugaac | ucuggaaccgguacuaaaagu | cuuuggucccgauucguguugaaccaguacuaaagggggaccuuuagucccacucuuuaguaccgguucuggaaccgguacuaaaagu | 22[19,3,0] | 12[9,3,0] | 10[10,0,0] | yes | 91 | RFL_Contig4702, AK249139 |
| Tae-2174a | contig176099_3752 | ccuguuugucauuaaguuucuu | uuaaugagaacaugugacaaa | ccuguuugucauuaaguuucuuagugccuuugccaucuuagugaagggucaaaagguacuuagaagcuuaaugagaacaugugacaaa | 85[82,0,3] | 84[81,0,3] | 1[1,0,0] | yes | 68 | AK252415, RFL_Contig2254 |
| Tae-2174b | contig1239786_10728 | ccuguuugucauuaaguuucuu | gaagcuuaaugagaacaugug | ccuguuugucauuaaguuucuuagcgccuuugccauccuagugacuagugaaaggucaaaagguacuuagaagcuuaaugagaacaugug | 82[82,0,0] | 81[81,0,0] | 1[1,0,0] | yes | 68 | AK252415, RFL_Contig2254 |
| Tae-2175a | contig773303_8615 | auuuguaguguucggauugaguuu | acuuaguccuggcacuauaaauuu | acuuaguccuggcacuauaaauuuaaaaaugugcaugugaaaauaugcucauuauaaauuuguaguguucggauugaguuu | 15[15,0,0] | 15[15,0,0] | 0[0,0,0] | yes | 149 | AK251439, AF532601_1 (***MRP2*** ) |
| Tae-2175b | contig1928562_12930 | auuuguaguguucggauugaguuu | acuuagucuuggcacuauaaauuu | acuuagucuuggcacuauaaauuuaaaaugugcaugugaaauaugcucauuauaaauuuguaguguucggauugaguuu | 15[15,0,0] | 15[15,0,0] | 0[0,0,0] | yes | 149 | AK251439, AF532601_1 (***MRP2*** ) |
| Tae-2176 | contig143877_3348 | cuuguuugucauuaagcuucug | gaagcuuaaugcgaacaugug | cuuguuugucauuaagcuucugagucucuuugccauccuauagcggugaaaggucaaaguuacucugaagcuuaaugcgaacaugug | 34[34,0,0] | 32[32,0,0] | 2[2,0,0] | yes | 99 | RFL_Contig1506 |
| Tae-2177 | contig346461_5614 | auaucucauuuucaacuauccc | aacaguugaagaugagauauug | aacaguugaagaugagauauugaacgaagauuuuauuucuuuuuguccaauaucucauuuucaacuauccc | 10[10,0,0] | 7[7,0,0] | 3[3,0,0] | yes | 21 | AK249411 |
| Tae-2178 | contig106441_2711 | ucagaaaucucgugugga | cacaugauucauucugcaa | ucagaaaucucguguggaauugagauguaaugcacaugauucauucugcaa | 963[963,0,0] | 116[116,0,0] | 847[847,0,0] | yes | 33 | AK248318 |
| Tae-2179 | contig360034_5734 | gggaaauuccuugucgggga | ucugucacuuccuca | gggaaauuccuugucggggauaacugucucugucacuuccuca | 42[42,0,0] | 7[7,0,0] | 35[35,0,0] | yes | 10 | AK249118 |
| Tae-2180a | contig3827810_16742 | cuugaggaauaguguagacugcac | gcagucuacccacucccucaaggc | cuugaggaauaguguagacugcacggagagcuauguguacuuuucguccgaaaccuuauagcuccgugcagucuacccacucccucaaggc | 8[8,0,0] | 1[1,0,0] | 7[7,0,0] | yes | 197 | RFL_Contig55, AF207745_1 (***TaAKT1***) |
| Tae-2180b | contig3576723_16244 | cuugaggaauaguguagacugcac | gcaguugacacuauucugaaagac | cuugaggaauaguguagacugcacggagcuaugugcagugcaguugacacuauucugaaagac | 8[8,0,0] | 1[1,0,0] | 7[7,0,0] | yes | 197 | RFL_Contig55, AF207745_1 (***TaAKT1***) |
| Tae-2181 | contig63933_1966 | uuagagauuucaauacggaca | uccguauguuucuaaau | uccguauguuucuaaauauaagccauuuuagagauuucaauacggaca | 22[22,0,0] | 22[22,0,0] | 0[0,0,0] | yes | 35 | RFL_Contig3639 |
| Tae-2182 | contig1368635_11233 | acgcuggucagacauguagcgccu | gcgcugcauaguguaaggugucg | acgcuggucagacauguagcgccuaucagccaggcgcugcauaguguaaggugucg | 10[10,0,0] | 10[10,0,0] | 0[0,0,0] | yes | 93 | AK252997 |
| Tae-2183 | contig1860742_12747 | uuuuuuucagugagagcuccu | gaccucucacuaaaua | gaccucucacuaaauaaauucaaugcucaaauaguggacaaaacuuugaacaacgacauuauuuuuuucagugagagcuccu | 10[10,0,0] | 10[10,0,0] | 0[0,0,0] | yes | 127 | AK249628 |
| Tae-2184 | contig1160517_10417 | agccauaacacguacauucagc | cgaauguauuuuuuauggcu | agccauaacacguacauucagcucuggcauccacgcacgcuguggcuuguguagccgaauguauuuuuuauggcu | 4[3,0,1] | 4[3,0,1] | 0[0,0,0] | yes | 8 | RFL_Contig4246 |
| Tae-2185 | contig580941_7454 | auacaugcgcgagcggcuggccu | guccgcuagcgcauguaucu | auacaugcgcgagcggcuggccuaaaauagacaugcauguguguaauuuuuguccgcuagcgcauguaucu | 9[9,0,0] | 5[5,0,0] | 4[4,0,0] | yes | 54 | AF023472_1 (***ptr1***) |
| Tae-2186 | contig3128711_15582 | augccauguuguucugaagaa | cuuuagaauagcaugaaaauuu | cuuuagaauagcaugaaaauuucugagaugauagucuagaaaugccauguuguucugaagaa | 7[7,0,0] | 7[7,0,0] | 0[0,0,0] | yes | 337 | AK248578 |
| Tae-2187 | contig4844488_18781 | cugguccccucccacacuuggccc | gccuugguggggggcggcaccagcc | gccuugguggggggcggcaccagcccaccuagggcugguccccucccacacuuggccc | 6[6,0,0] | 5[5,0,0] | 1[1,0,0] | yes | 28 | RFL_Contig1154 |
| Tae-2188 | contig542503_7224 | cgaaaaguacuacuuuggccu | gccgaaaaguacugcuucggc | cgaaaaguacuacuuuggccuagcucuggccaaaaaguacuacuucgggcuagcuaaggccgaaaaguacugcuucggc | 6[5,1,0] | 2[2,0,0] | 4[3,1,0] | yes | 12 | AK249459 |
| Tae-2189a | contig82452_2298 | uauuauggugcggagggagua | ccuccucuguaucacaauaua | ccuccucuguaucacaauauauguuguuuuagcaacuuauaaauuaaauugcuaaagcuacauaauuauuauggugcggagggagua | 7[7,0,0] | 6[6,0,0] | 1[1,0,0] | yes | 62 | RFL_Contig4637, AK248893 |
| Tae-2189b | contig701555_8210 | uauuauggugcggagggagua | cuuccuuucguuacuuauauu | uauuauggugcggagggaguacuugucaaggaucgucauaugucuuucguuacuugauuuccuucacuacuuccuuucguuacuuauauu | 13[13,0,0] | 7[7,0,0] | 6[6,0,0] | yes | 62 | RFL_Contig4637, AK248893 |
| Tae-2190a | contig477975_6789 | ugugcucuuuccuucuuaccc | guaagaagcaaauagcacaug | guaagaagcaaauagcacaugcaggagguaagaaagaagcaagcgaaugcgugugcucuuuccuucuuaccc | 6[6,0,0] | 6[6,0,0] | 0[0,0,0] | yes | 40 | AK251284, AK248476 |
| Tae-2190b | contig3001237_15345 | ugugcucuuuccuucuuaccc | guaagaagcaaauagcacaug | guaagaagcaaauagcacaugcaggagguaagaagcaaacgaaugugugugcucuuuccuucuuaccc | 6[6,0,0] | 6[6,0,0] | 0[0,0,0] | yes | 40 | AK251284, AK248476 |
| Tae-2190c | contig3010976_15363 | ugugcucuuuccuucuuaccc | guaagaagaaaauagcacaug | guaagaagaaaauagcacaugcaggagguaagaagcaaacgaaugugugugcucuuuccuucuuaccc | 6[6,0,0] | 6[6,0,0] | 0[0,0,0] | yes | 40 | AK251284, AK248476 |
| Tae-2191a | contig205503_4134 | uucgucggacgagcgugccu | gcgcgcuccuccgucgaacg | uucgucggacgagcgugccuaggcuaucggccgcgcgcuccuccgucgaacg | 51[51,0,0] | 50[50,0,0] | 1[1,0,0] | yes | 101 | RFL_Contig4524,RFL_Contig2591 |
| Tae-2191b | contig1552523_11876 | uucgucggacgagcgugccu | gcgcgcuccuccgucgaacg | uucgucggacgagcgugccuaggcuaucggccgcgcgcuccuccgucgaacg | 51[51,0,0] | 50[50,0,0] | 1[1,0,0] | yes | 101 | RFL_Contig4524,RFL_Contig2591 |
| Tae-2191c | contig3589520_16257 | uucgucggacgagcgugccu | gcgcgcuccuccgucgaacg | uucgucggacgagcgugccuaggccaucggccgcgcgcuccuccgucgaacg | 51[51,0,0] | 50[50,0,0] | 1[1,0,0] | yes | 101 | RFL_Contig4524,RFL_Contig2591 |
| Tae-2191d | contig537825_7191 | uucgucggacgagcgugccu | gcgcacuccuccgucgaacg | uucgucggacgagcgugccuaggcaucagccgcgcacuccuccgucgaacg | 51[51,0,0] | 50[50,0,0] | 1[1,0,0] | yes | 101 | RFL_Contig4524,RFL_Contig2591 |
| Tae-2191e | contig205503_4134 | uucgucggacgagcgugccu | gcgcgcuccuccgucgaacg | uucgucggacgagcgugccuaggcuaucggccgcgcgcuccuccgucgaacg | 51[51,0,0] | 50[50,0,0] | 1[1,0,0] | yes | 101 | RFL_Contig4524,RFL_Contig2591 |
| Tae-2192a | contig444816_6492 | uucgccggucgcgcguucccu | agcgcgccgccgucgaaagg | uucgccggucgcgcguucccuuacggugagcgcgccgccgucgaaagg | 29[26,0,3] | 25[25,0,0] | 4[1,0,3] | yes | 506 | RFL_Contig1486 |
| Tae-2192b | contig3141743_15597 | uucgccggucgcgcguucccu | agcgcgccgccgucgaaagg | uucgccggucgcgcguucccuuacggugagcgcgccgccgucgaaagg | 29[26,0,3] | 25[25,0,0] | 4[1,0,3] | yes | 506 | RFL_Contig1486 |
| Tae-2192c | contig95434_2517 | uucgccggucgcgcguucccu | gugagcgcgccgccgucgaaac | uucgccggucgcgcguucccuuacggugagcgcgccgccgucgaaac | 26[26,0,0] | 25[25,0,0] | 1[1,0,0] | yes | 506 | RFL_Contig1486 |
| Tae-2192d | contig1282657_10869 | uucgccggucgcgcguucccu | gugagcgcgccgccgucgaaac | uucgccggucgcgcguucccuuacggugagcgcgccgccgucgaaac | 26[26,0,0] | 25[25,0,0] | 1[1,0,0] | yes | 506 | RFL_Contig1486 |
| Tae-2193 | contig3874161_16862 | cagaaccagaaugaguagcuc | gcugcucauucugguuuuaga | cagaaccagaaugaguagcucaugcaccuacucaacauagugaaugugagcugcucauucugguuuuaga | 38[38,0,0] | 38[38,0,0] | 0[0,0,0] | yes | 72 | M90664_1 (***E1*)** |
| Tae-2194a | contig1236006_10709 | cucgccggucgcgcguucucccu | agagcgcaccgccgucgaggg | cucgccggucgcgcguucucccucagcggugagagcgcaccgccgucgaggg | 41[32,0,9] | 22[14,0,8] | 19[18,0,1] | yes | 731 | RFL_Contig1486 |
| Tae-2194b | contig599141_7583 | cucgccggucgcgcguucucccu | ugagcgcaccgccgucgaggg | cucgccggucgcgcguucucccucagcggugagcgcaccgccgucgaggg | 39[32,0,7] | 20[14,0,6] | 19[18,0,1] | yes | 731 | RFL_Contig1486 |
| Tae-2194c | contig426428_6298 | cucgccggucgcgcguucucccu | ugagcgcaccgccgucgaggg | cucgccggucgcgcguucucccucagcggugagcgcaccgccgucgaggg | 41[32,0,9] | 22[14,0,8] | 19[18,0,1] | yes | 731 | RFL_Contig1486 |
| Tae-2195 | contig00619_67 | uccgauccagauuaauugacg | aauuaauauggaucggaggga | uccgauccagauuaauugacgcuaguuuaguacaaaguguacaacuuuguacuagaccagcgucaauuaauauggaucggaggga | 39[18,0,21] | 35[15,0,20] | 4[3,0,1] | yes | 12 | AK249471 |
| Tae-2196 | contig2297694_13883 | uggacgaggaugugcaacugc | gcacauccauguccaagcucu | uggacgaggaugugcaacugcgguacgguaggaccuucagcuacgaccggcagcugcacauccauguccaagcucu | 342[333,0,9] | 333[329,0,4] | 9[4,0,5] | yes | 287 | AK248421 |
| Tae-2197 | contig1369263_11240 | cggccgcgccggcggcca | gcugccagucacggcaggg | gcugccagucacggcagggcggcggcgcccggccgcgccggcggcca | 17[17,0,0] | 16[16,0,0] | 1[1,0,0] | yes | 478 | RFL_Contig6074 |
| Tae-2198 | contig2985567_15315 | cucgccggagcagcaugccg | gcgugcuccuucgucgagcg | cucgccggagcagcaugccguggacgauguccggcgugcuccuucgucgagcg | 13[13,0,0] | 11[11,0,0] | 2[2,0,0] | yes | 415 | AY483152_1 (***CesA2***) |
| Tae-2199 | contig496321_6931 | cucuccuguagauauaggcaccgg | uuauuuguacaagguagacgcuu | uuauuuguacaagguagacgcuuaacuaagcgucucuccuguagauauaggcaccgg | 236[224,0,12] | 133[128,0,5] | 103[96,0,7] | yes | 85 | RFL_Contig4208 |
| Tae-2200 | contig3619587_16325 | ugccaaaggagaguugcccug | gggcuccucuuauuggcagg | gggcuccucuuauuggcagggagcgugugaggccauguagcuccauucagcgcucugccaaaggagaguugcccug | 34[34,0,0] | 33[33,0,0] | 1[1,0,0] | yes | 34 | AK251663 |
| Tae-2201 | contig389713_5984 | acgaaacggacagcgggcggacgc | gucaccccguugggccaacuuuugug | acgaaacggacagcgggcggacgcuguguccguuuaggucaccccguugggccaacuuuugugu | 30[30,0,0] | 27[27,0,0] | 3[3,0,0] | yes | 33 | RFL_Contig2110 |
| Tae-2202 | contig1067062_10017 | cgaagacaaagagaacuuuu | auguuuaucccuuaucuuugca | cgaagacaaagagaacuuuucagauggaagugcuauaguaugucaugcuauuaugaauugucugagauguuuaucccuuaucuuugca | 60[60,0,0] | 8[8,0,0] | 52[52,0,0] | yes | 39 | RFL_Contig4734 |
| Tae-2203 | contig3150844_15617 | uucgccggucgcgcguuccccu | gugagcgcgccgccguugaaug | uucgccggucgcgcguuccccuugcggugagcgcgccgccguugaaug | 39[39,0,0] | 36[36,0,0] | 3[3,0,0] | yes | 568 | RFL_Contig1486 |
| Tae-2204 | contig1095979_10124 | aaaugcggacagcaaagacuuugc | aaagagucuuugccgucugcaauuuu | aaagagucuuugccgucugcaauuuuauuuacagacggcaaagagcucacuuugcccucuguaaauauaaaugcggacagcaaagacuuug | 14[5,9,0] | 10[5,5,0] | 4[0,4,0] | yes | 9 | AK249681 |
| Tae-2205a | contig1884136_12833 | cugcauuugcaccugcaccua | ggugcaguggcacaugcagcc | cugcauuugcaccugcaccuacgaugaagaacaaggaaacgccuuuguuaucaucaaguugcuaggugcaguggcacaugcagcc | 71[71,0,0] | 71[71,0,0] | 0[0,0,0] | yes | 49 | AK248722, AK251439 |
| Tae-2205b | contig4624450_18391 | cugcauuugcaccugcaccua | ggugcaguggcacaugcagcc | cugcauuugcaccugcaccuacgacgaagaacaaggaaacgccuuuugucauccucaaguugcuaggugcaguggcacaugcagcc | 71[71,0,0] | 71[71,0,0] | 0[0,0,0] | yes | 49 | AK248722, AK251439 |
| Tae-2206a | contig346286_5611 | cucuccgguagaaauaggcaccgg | ggugcuuauuguacaggauagau | ggugcuuauuguacaggauagaugcuuaacuaagcgucucuccgguagaaauaggcaccgg | 228[228,0,0] | 128[128,0,0] | 100[100,0,0] | yes | 58 | AK250208, AK250846 |
| Tae-2206b | contig814930_8846 | cucuccgguagaaauaggcaccgg | ggugcuuauuuguacaugauagac | ggugcuuauuuguacaugauagacguuuaacuaagcgucucuccgguagaaauaggcaccgg | 228[228,0,0] | 128[128,0,0] | 100[100,0,0] | yes | 58 | AK250208, AK250846 |
| Tae-2207 | contig4476999_18095 | uugauuuuccucuauaagcgcauc | ugcccuuauauagggaaauuagcc | ugcccuuauauagggaaauuagccuaaauucauacggauuucgauugaaauucguuaugaauuuaaguugauuuuccucuauaagcgcau | 19[19,0,0] | 19[19,0,0] | 0[0,0,0] | yes | 67 | AB042240_15 (***chloroplast DNA***) |
| Tae-2208 | contig1563490_11906 | ugcaucauuuggaacucgccg | gcaaguuccagacgaugcagg | ugcaucauuuggaacucgccggugcgauagcacgcagcaugaccuggcguucaucaccggcaaguuccagacgaugcagg | 21[21,0,0] | 21[21,0,0] | 0[0,0,0] | yes | 40 | RFL_Contig1389 |
| Tae-2209 | contig172807_3715 | uacggccaagccgucggcaua | ugccgacggcuugccauagg | uacggccaagccgucggcauagauuuauaucuaugccgacggcuugccauagg | 177[177,0,0] | 176[176,0,0] | 1[1,0,0] | yes | 111 | AK252326 |
| Tae-2210 | contig4606075_18348 | ugagccgaaccaauaucacuc | uauuguuucggcucauguccu | uauuguuucggcucauguccucgucgagcacaucgucggaugagaugugagccgaaccaauaucacuc | 183[182,0,1] | 183[182,0,1] | 0[0,0,0] | yes | 7 | RFL_Contig2448 |
| Tae-2211 | contig3013472_15368 | cgguggggaguuuggcug | gcucgggcgcgcgcgcguc | gcucgggcgcgcgcgcgucgacacgccucugccuacugguagagguugaagacgguggggaguuuggcug | 152[152,0,0] | 38[38,0,0] | 114[114,0,0] | yes | 82 | AK248318 |
| Tae-2212 | contig608257_7658 | acacuuauuuccgaucggagg | uccgaucagaaauaaguuucg | uccgaucagaaauaaguuucgugguuuuaguucaaauuuuuaaacuaaaaccaugacacuuauuuccgaucggagg | 14[13,1,0] | 7[6,1,0] | 7[7,0,0] | yes | 9 | AK252407 |
| Tae-2213 | contig3205398_15694 | gugggcccgcucugguuuag | agggcgugggccuaggu | gugggcccgcucugguuuaguuaauccuaaacagauauuaaacugggguuaauuagggcgugggccuaggu | 12[12,0,0] | 6[6,0,0] | 6[6,0,0] | yes | 8 | AY663392_4 |
| Tae-2214 | contig1569657_11932 | auagcaucauccauccuaccc | guaagauggcugaugcuaugg | auagcaucauccauccuacccuucaacggugguugccgauucccacguuggguaagauggcugaugcuaugg | 2761[2761,0,0] | 2717[2717,0,0] | 44[44,0,0] | yes | 16 | RFL_Contig2142 |
| Tae-2215 | contig154805_3489 | ccucaaacgcucggucugaccggc | ccggucaggccgaccggucacuggcc | ccggucaggccgaccggucacuggccuguuacaaaauaacgaucuaaucggagcuuucaaacgggccucaaacgcucggucugaccggc | 14[14,0,0] | 6[6,0,0] | 8[8,0,0] | yes | 71 | AK250882 |
| Tae-2216 | contig2955072_15264 | uauucuggugugcuaggcg | ccaggagggcaccccaaguagg | uauucuggugugcuaggcgcccccucccacauauauauaggugggggaggagggagcagccaggagggcaccccaaguagg | 1044[1044,0,0] | 892[892,0,0] | 152[152,0,0] | yes | 13 | RFL_Contig5742 |
| Tae-2217 | contig11878_590 | cggcggauugcuggagcu | cggcggauuuccgccgau | cggcggauugcuggagcucacgcaaaucgugagcggcggauuuccgccgau | 178[178,0,0] | 104[104,0,0] | 74[74,0,0] | yes | 41 | AK250317 |
| Tae-2218 | contig768112_8578 | gaugggggccggcgaugc | gugccugcugcagcgcg | gaugggggccggcgaugccgauggcgugccugcugcagcgcg | 35[35,0,0] | 16[16,0,0] | 19[19,0,0] | no | 8 | RFL_Contig4025 |
| Tae-2219 | contig244456_4590 | gggauaucuucaaucgguuuggau | gccugccgguuguggcaguccugu | gauaucuucaaucgguuuggauggcggucaacuaaauagauagguguuuaguuccuaucuguuuuuugccugccgguuguggcaguccu | 12[12,0,0] | 10[10,0,0] | 2[2,0,0] | yes | 53 | AK250170 |
| Tae-2220 | contig271167_4875 | ucguuccuuucucgcaaacgag | cguucguggagaggaacgagc | ucguuccuuucucgcaaacgagcuagaaaccagcuucccuuguggccgugcugguuacuauuucguucguggagaggaacgagc | 4[4,0,0] | 4[4,0,0] | 0[0,0,0] | yes | 68 | AK250613 |
| Tae-2221 | contig2996054_15340 | caaaaaaugguccugggggc | ccccggccgccggca | caaaaaaugguccugggggcgaguugguccccggccgccggca | 4[4,0,0] | 0[0,0,0] | 4[4,0,0] | yes | 31 | RFL_Contig5776 |
| Tae-2222 | contig632315_7817 | gcccuggcccggcgcacccca | ggguggcccaucacaguag | ggguggcccaucacaguagcaugcgucgucugggcguugcacugcugcccuggcccggcgcacccca | 29[27,2,0] | 20[18,2,0] | 9[9,0,0] | yes | 69 | RFL_Contig4234 |
| Tae-2223 | contig1644676_12166 | cugaaagcauauaaguagua | guacuauaugcugcagua | cugaaagcauauaaguaguaauacguuuucagaaaagugaaagcguacuauaugcugcagua | 134[134,0,0] | 8[8,0,0] | 126[126,0,0] | yes | 30 | RFL_Contig2812 |
| Tae-2224 | contig1816694_12607 | gaagagcaccgcacgucga | ggugugcggucggacguacua | ggugugcggucggacguacuagggaucugcuagagcaggcggcuguccuagaagagcaccgcacgucga | 33[33,0,0] | 9[9,0,0] | 24[24,0,0] | no | 141 | AK248318 |
| Tae-2225 | contig172799_3713 | aggcaguggcuugguuaaggga | cuuuaccggaucacaaaaggg | cuuuaccggaucacaaaagggacuaaucaggcaguggcuugguuaaggga | 199[199,0,0] | 24[24,0,0] | 175[175,0,0] | yes | 140 | AK251284 |
| Tae-2226 | contig881186_9199 | gagggagggcagaggcuu | cccuuucccaacuuccuucuc | gagggagggcagaggcuugcacgcucucccucgcuccacccuuucccaacuuccuucuc | 28[28,0,0] | 0[0,0,0] | 28[28,0,0] | no | 58 | AK248810 |
| Tae-2227 | contig172691_3710 | cugaggacugggcucggg | cuaggauagccguuggcc | cugaggacugggcucgggacuauugacgauggucucuaggauagccguuggcc | 427[427,0,0] | 176[176,0,0] | 251[251,0,0] | no | 25 | AK248318 |
| Tae-2228 | contig1047394_9933 | ugacagaagagagggagcau | guuuaucugaucuggaaaug | ugacagaagagagggagcaugcuuagguagguuuaucugaucuggaaaug | 20[20,0,0] | 19[19,0,0] | 1[1,0,0] | no | 23 | RFL_Contig4144 |
| Tae-2229a | contig370930_5810 | aaggaucaucauacaugu | gugauguugaugaaauuuag | gugauguugaugaaauuuaggugaguauuaugugaguuguaggugcucauaaaggaucaucauacaugu | 16[16,0,0] | 3[3,0,0] | 13[13,0,0] | yes | 15 | RFL_Contig5558 |
| Tae-2229b | contig423007_6272 | aaggaucaucauacaugu | gugauguugaugaaauuuag | gugauguugaugaaauuuaggugaguauuaugugaguuguaggugcucauaaaggaucaucauacaugu | 16[16,0,0] | 3[3,0,0] | 13[13,0,0] | yes | 15 | RFL_Contig5558 |
| Tae-2230 | contig3530906_16194 | ccaacuugauagcuuugaccggu | cggugcagcccgucgguggug | cggugcagcccgucgguggugauaauaaugaugguggaccggucauaauugucaaccaacuugauagcuuugaccggu | 19[18,1,0] | 8[7,1,0] | 11[11,0,0] | yes | 108 | AK248936 |
| Tae-2231 | contig2071105_13361 | aaggaggugggggugcaaaaa | guacuaccgcuaccuccucac | guacuaccgcuaccuccucacaaucuccacuuucgucgaaaugacaaacucccaggagaggaaaggaggugggggugcaaaaa | 32[32,0,0] | 4[4,0,0] | 28[28,0,0] | yes | 445 | AK253043 |
| Tae-2232 | contig412239_6168 | aacuacaaucugaggcuu | gccaaaucugauuguagaugc | aacuacaaucugaggcuugaucggucaguauccgcggaguuugaguggucgaauaugccaaaucugauuguagaugc | 15[15,0,0] | 2[2,0,0] | 13[13,0,0] | yes | 17 | AK249854 |
| Tae-2233 | contig66765_2007 | gaagaaugagccggcgac | cacggccacauuugccugg | cacggccacauuugccugguuaagccgaagaaugagccggcgac | 166[166,0,0] | 29[29,0,0] | 137[137,0,0] | no | 55 | RFL_Contig2459 |
| Tae-2234 | contig293089_5094 | uuccgagaggcacggcuuugacuc | agcacaaccgugccucuugcggaagc | uuccgagaggcacggcuuugacucucgugaagcacaaccgugccucuugcggaagc | 4[4,0,0] | 3[3,0,0] | 1[1,0,0] | yes | 58 | RFL_Contig4830 |
| Tae-2235 | contig2716339_14848 | guguuguaacaugcuggcuug | agccugcgugccuuaugacucgg | guguuguaacaugcuggcuugcauacccauguaauuaggaucacaagccugcgugccuuaugacucgg | 21[21,0,0] | 10[10,0,0] | 11[11,0,0] | no | 74 | RFL_Contig5030 |
| Tae-2236 | contig2766119_14941 | gccgaugaguccggaaguuugggc | acuagccggacagcccguugggac | gccgaugaguccggaaguuugggccggcuacgagaguccacuugaguggcuuuuuuuuuguucgggcacuagccggacagcccguuggga | 8[7,0,1] | 6[6,0,0] | 2[1,0,1] | no | 157 | AK248714 |
| Tae-2237 | contig605321_7636 | caagcagugggaggggaaa | gccucacccaucgcuagagcc | gccucacccaucgcuagagccgagagcgacuggaucgcuugcaacggcaagcagugggaggggaaa | 28[28,0,0] | 4[4,0,0] | 24[24,0,0] | no | 69 | AK250784 |
| Tae-2238 | contig1379187_11263 | auuaguaacucgccacacagaa | augucugggaguuucgcugauag | augucugggaguuucgcugauagcgccuucuuuacuaguggaggugcuucauuaguaacucgccacacagaa | 23[23,0,0] | 4[4,0,0] | 19[19,0,0] | yes | 4 | RFL_Contig4970 |
| Tae-2239 | contig3076104_15496 | ucgagaucguugcaagcugagcag | gccacuuuguacaacuugaucuugag | ucgagaucguugcaagcugagcagagccacuuuguacaacuugaucuugagc | 14[14,0,0] | 8[8,0,0] | 6[6,0,0] | yes | 165 | RFL_Contig5033 |
| Tae-2240 | contig2851415_15106 | guugagauauggcauucugagugac | cgaaggaaccaauguuuccacac | cgaaggaaccaauguuuccacacgugcacuguugagauauggcauucugagugac | 20[20,0,0] | 2[2,0,0] | 18[18,0,0] | no | 156 | AK248902 |
| Tae-2241 | contig4578333_18289 | agucgcgguuggccagaagaaccg | guugggcaggcgaccgcgacuaa | cgcgguuggccagaagaaccgcgacuaaaggugggggcgcgggaacgcaaaaaacccgccaaaaacccuuagucgcgguugggcaggcgac | 1[1,0,0] | 1[1,0,0] | 0[0,0,0] | yes | 242 | RFL_Contig3557 |
| Tae-2242 | contig187007_3905 | gggccgggucggccggug | ccggccgucccugcaucg | ccggccgucccugcaucgggccgugcccggaaguucgcccggggggccgggucggccggug | 8[8,0,0] | 8[8,0,0] | 0[0,0,0] | yes | 155 | AK251731 |
| Tae-2243 | contig24155_977 | auggauggcgcugaagcu | cugaagaugcuaccaaua | auggauggcgcugaagcugccaccggcugaagaugcuaccaaua | 138[138,0,0] | 15[15,0,0] | 123[123,0,0] | no | 86 | AK248508 |
| Tae-2244 | contig2515702_14400 | gccucucucaccccacca | agugauccugaggucgagagu | agugauccugaggucgagaguucgagccucucucaccccacca | 153[149,0,4] | 51[49,0,2] | 102[100,0,2] | no | 11 | RFL_Contig661 |
| Tae-2245 | contig60031_1846 | gcuacucuccucauaggau | cugcaugauuucuuagucc | cugcaugauuucuuagucccagcuacucuccucauaggau | 30[30,0,0] | 11[11,0,0] | 19[19,0,0] | yes | 13 | AF164405_1 |
| Tae-2246 | contig476910_6775 | cgaagaauugaaucccggcg | caaggguucuucuccucgca | caaggguucuucuccucgcacagcagaggaacaaaucuuggauggcuuguuucucuuuugugcgaagaauugaaucccggcg | 9[8,1,0] | 9[8,1,0] | 0[0,0,0] | yes | 26 | RFL_Contig4865 |
| Tae-2247 | contig1491442_11671 | auuaguaacucgccacacagau | augucugggaguuucgcugauag | augucugggaguuucgcugauagcgccuucuuaacgguggaggugcuucauuaguaacucgccacacagau | 211[211,0,0] | 27[27,0,0] | 184[184,0,0] | yes | 4 | AK251382 |
| Tae-2248 | contig1855197_12728 | caaccgguacuaaagggcucccug | ccuuuuaauaccgguuggugacac | ccuuuuaauaccgguuggugacaccaaccgguacuaaagggcucccug | 15[14,0,1] | 10[9,0,1] | 5[5,0,0] | yes | 6 | AK249896 |
| Tae-2249 | contig4115927_17337 | cagagauuguaguugaugaguucuu | gaacuucucagcugauguuauauc | cagagauuguaguugaugaguucuugcggaacuucucagcugauguuauauc | 3[3,0,0] | 0[0,0,0] | 3[3,0,0] | yes | 285 | RFL_Contig5299 |
| Tae-2250 | contig94531_2504 | uuccagagggauugcacugauc | ucaaugcgaucccuuggaauc | uuccagagggauugcacugauccaucgaccuugcacggagagaucgaucaaugcgaucccuuggaauc | 8[8,0,0] | 7[7,0,0] | 1[1,0,0] | yes | 351 | AY485643_5 |
| Tae-2251 | contig4199570_17569 | gacucuguggcccaaugga | uguuggguaacuagugcc | uguuggguaacuagugccugcuggugugauguuaacguuugauuacauucguuagugacucuguggcccaaugga | 30[30,0,0] | 29[29,0,0] | 1[1,0,0] | no | 1 | AY093954_1 (***Wph***) |
| Tae-2252 | contig39550_1389 | auugaggucuaauuggag | ucaauuagauuucaguaa | auugaggucuaauuggagagauggucaauuagauuucaguaa | 3[3,0,0] | 3[3,0,0] | 0[0,0,0] | yes | 10 | AK248182 |
| Tae-2253 | contig2110861_13447 | ucuacaguccggacgaug | uaguccggcuguccagaua | ucuacaguccggacgaugcauauaguccggcuguccagaua | 18[18,0,0] | 1[1,0,0] | 17[17,0,0] | yes | 0 | AK252858 |
| Tae-2254 | contig4857495_18798 | guccauuugagucgggccgacuuu | uguccgccgcgauucaaacagacgc | guccauuugagucgggccgacuuuucuguccgccgcgauucaaacagacgc | 8[8,0,0] | 2[2,0,0] | 6[6,0,0] | yes | 73 | AK249198 |
| Tae-2255 | contig61492_1872 | auauaaggucucacccucuagccu | gaauagaggguagucuguuuag | auauaaggucucacccucuagccucaauaugcauugugagcggcaccaccgauaaggcaaaggaauagaggguagucuguuuag | 10[5,5,0] | 9[5,4,0] | 1[0,1,0] | yes | 1 | RFL_Contig2834 |
| Tae-2256 | contig1618727_12094 | augucgaagguaguagcucga | uagcugcugccuucaacauuu | augucgaagguaguagcucgacugguuaugcugcgcguguggcacaucgucggcgcgaguucgaauccuagcugcugccuucaacauuu | 9[9,0,0] | 8[8,0,0] | 1[1,0,0] | yes | 28 | RFL_Contig4833 |

The genome sequence and the EST sequence of wheat were used as reference. * Targets prediction in EST database and the detailed information is listed in Table S3. The bold and italic indicated the annotation gene.
